# Discovery of New Nanomolar Selective IRAP Inhibitors

**DOI:** 10.1021/acs.jmedchem.4c01744

**Published:** 2025-02-07

**Authors:** Ben He, Nour Bou Karroum, Ronan Gealageas, François-Xavier Mauvais, Sandrine Warenghem, Matthieu Roignant, Nicolas Kraupner, Bao Vy Lam, Nathalie Azaroual, Vincent Ultré, Alexandre Rech, Laetitia Lesire, Cyril Couturier, Florence Leroux, Peter van Endert, Benoit Deprez, Rebecca Deprez-Poulain

**Affiliations:** † Univ. Lille, Inserm, Institut Pasteur de Lille, U1177 - Drugs and Molecules for Living Systems, F-59000 Lille, France; ‡ European Genomic Institute for Diabetes, EGID, University of Lille, Lille F-59000, France; § Institut Necker Enfants Malades, Université Paris Cité, INSERM, CNRS, Paris F-75015, France; ∥ Service de Physiologie—Explorations Fonctionnelles, AP-HP, Hôpital Robert-Debré, Paris F-75019, France; ⊥ University Lille, CHU Lille, ULR 7365—GRITA—Groupe de Recherche Sur Les Formes Injectables Et Les Technologies Associées, Lille F-59000, France; # University Lille, Plateau RMN Pharmacie, UFR3S-Pharmacie, Lille F-59000, France; ¶ Service Immunologie Biologique, AP-HP, Hôpital Universitaire Necker-Enfants Malades, Paris F-75015, France

## Abstract

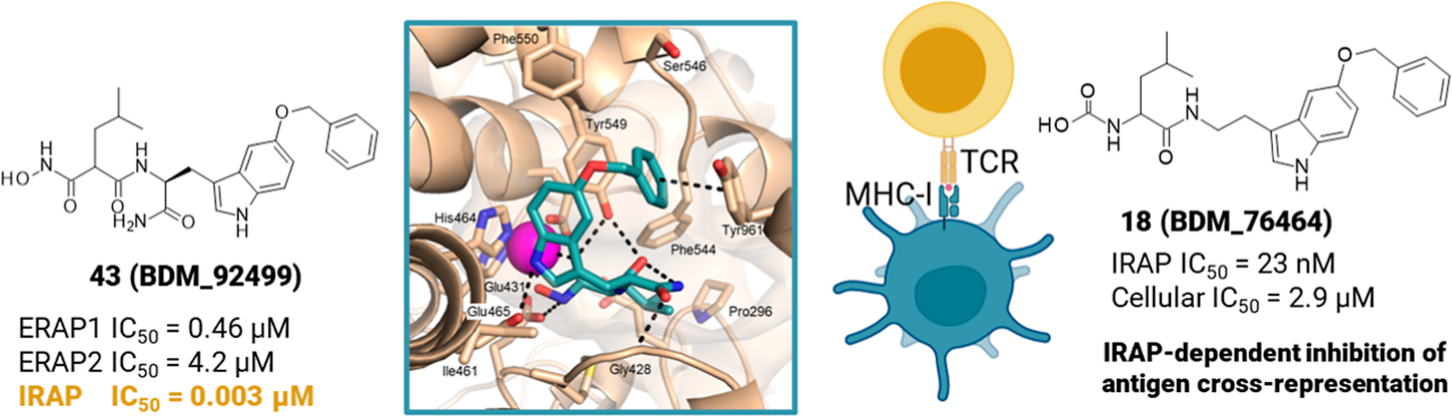

Among the M1 family of oxytocinase aminopeptidases, insulin-regulated aminopeptidase IRAP, is an emerging drug target implicated in various biological pathways and particularly in MHC-I antigen presentation through amino-terminal trimming of exogenous cross-presented peptides. A few series of inhibitors inspired either by angiotensin IV, one of IRAP substrates, or by bestatin a pan aminopeptidase inhibitor, have been disclosed. However, the variety and number of chemotypes remains relatively limited. Here we disclose the design and optimization of a series of hydroxamic acids IRAP inhibitors bearing a 5-substituted indole. Docking studies of the best compound **43** (**BDM_92499**), a single-digit nanomolar and selective inhibitor of IRAP, suggest an original binding mode and highlight the substituent on the indole and a primary amide as groups driving selectivity. Several inhibitors in the series displayed IRAP-dependent inhibition of antigen cross-presentation. These results pave the way to the development of novel therapeutic agents targeting IRAP.

## Introduction

**Figure 1:**
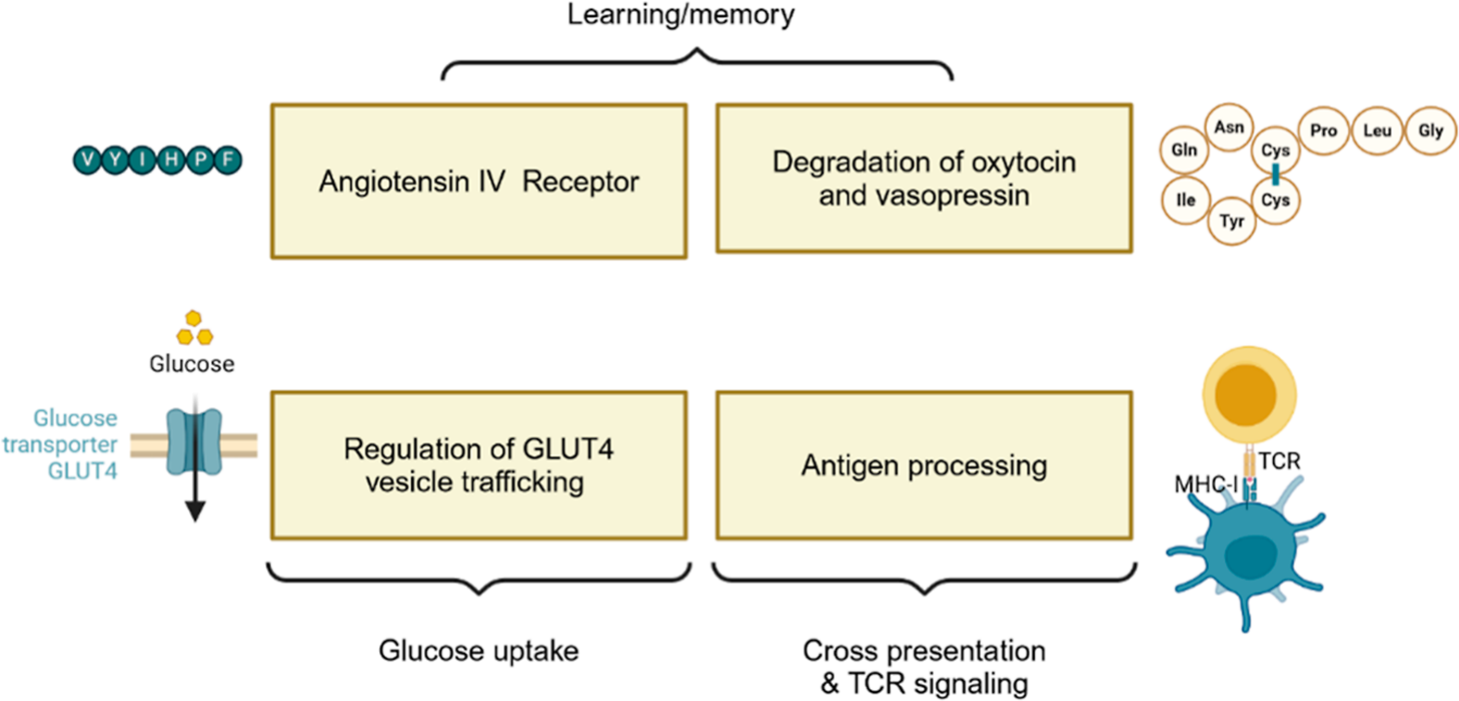
Physiological roles of insulin-regulated aminopeptidase. Created with Biorender.com.

Insulin-regulated aminopeptidase (IRAP) is a type II transmembrane Zn protease belonging to the M1 family of aminopeptidases. It exerts diverse functions in various biological pathways (Figure 1). It colocalizes with the glucose transporter GLUT4 in vesicles that move to the plasma membrane upon activation of the insulin receptor to facilitate glucose uptake, hence its name.^1,2^ As an aminopeptidase, it degrades several peptidic substrates including the cyclic peptides oxytocin^3^ and vasopressin^4^ and angiotensin III.^5^ It has also been proposed as the membrane receptor for angiotensin IV (AngIV), an inhibitor of IRAP.^6^ Because of its effect on AngIV,^7^ oxytocin, and vasopressin which have implications for cognition, IRAP is considered to be a potential target for memory and learning disorders.^8,9^ Finally, IRAP has been implicated in the progression of both cardiac and renal fibrosis^10,11^

Like the closely related M1-aminopeptidases ER aminopeptidase 1 and 2 (ERAP1 and ERAP2), IRAP is also involved in the generation of major histocompatibility complex (MHC) class I antigens. More specifically, while ERAPs are involved in the presentation of endogenously produced antigens, IRAP is involved in the presentation of exogenous peptides through a process called cross-presentation and in the trafficking of the T-cell receptors (TCR).^12,13^

The IRAP structure shows three distinct domains: a cytoplasmic N-terminal domain of 109 amino acids, a transmembrane domain of 23 amino acids, and an extracellular domain of 893 amino acids. The large C-terminal intraendosomal domain contains a Zn-binding motif HEXXH(X)18E and the exopeptidase motif GAMEN.^14^ IRAP shares a high percentage of protein sequence identity with ERAP1 and ERAP2 (43% and 49%, respectively).^15^ The substrate specificity of IRAP and its closest homologues, ERAP1 and ERAP2, is largely due to differences in their S1 pockets. ERAP1 favors hydrophobic and aromatic side chains, whereas ERAP2 prefers chains with positively charged amino acids. IRAP shares specificity features with both ERAPs.^16^

**Figure 2:**
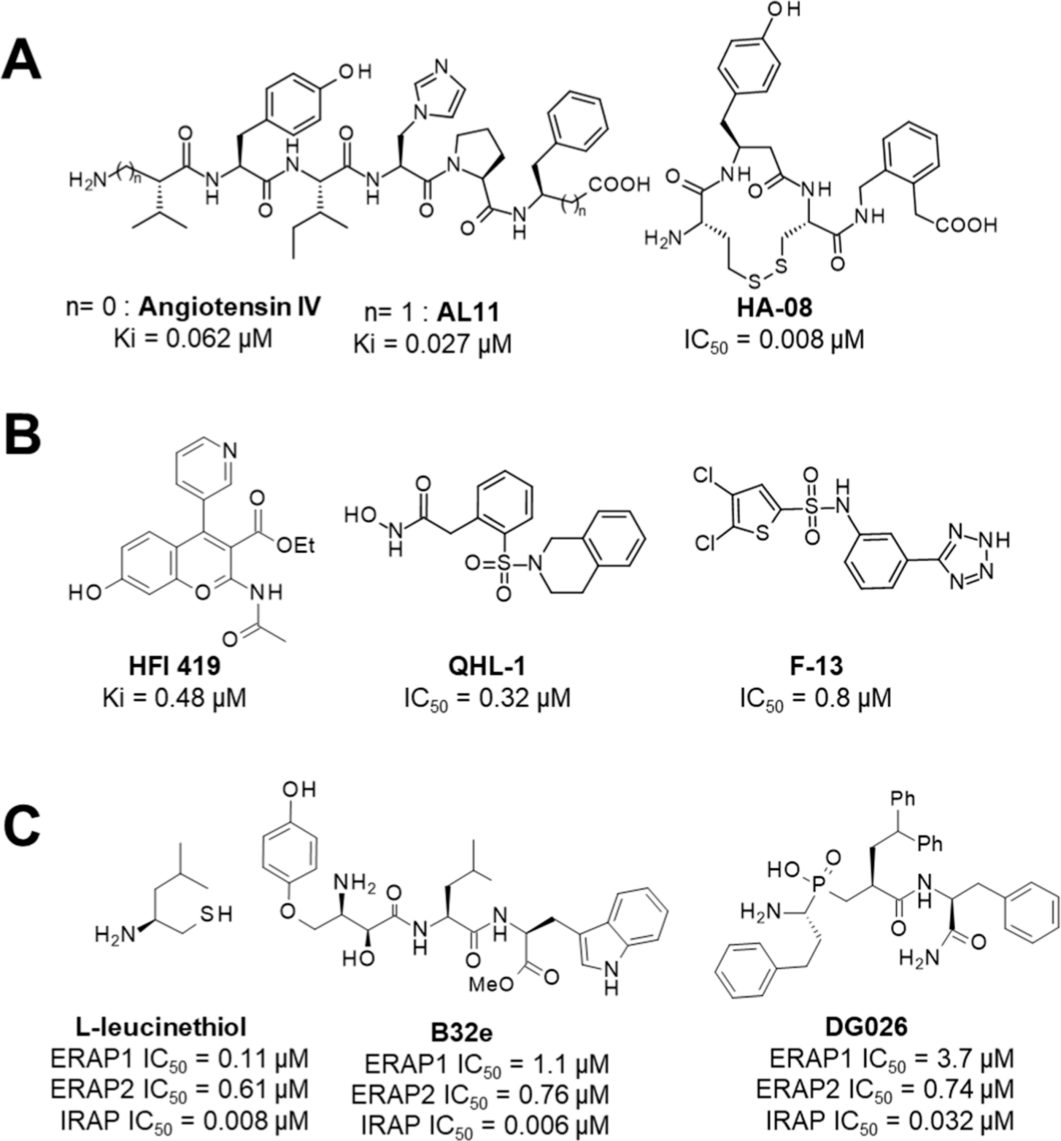
IRAP inhibitors from literature. (A) AngIV-based inhibitors: peptidic **AL-11** or macrocyclic **HA-08**; (B) inhibitors derived from either virtual or wet screening: coumarin **HFI419**, sulfonamides **QHL-1** and **F-13**; (C) M1 aminopeptidase inhibitors: leucinethiol,; transition state analogues **B32e** bestatin-derived and phosphinic pseudopeptide **DG026**.

Several families of IRAP inhibitors have been disclosed.^17,18^ The first IRAP inhibitors were derived from **AngIV** (Figure 2A) and were developed for therapeutic intervention in neurological and cognitive disorders. **AngIV** is a potent IRAP inhibitor (*K*_i_ = 62 nM), however, it is poorly stable in plasma.^19^ Close analogue **AL-11** shows improved stability and greater potency (*K*_i_ = 27 nM).^20^ Macrocyclization of pseudopeptides provided the low nanomolar inhibitor **HA-08** (*K*_i_ = 3.3 nM) which was cocrystallized with IRAP.^21,22^

Additional inhibitors were identified by screening (Figure 2B). For example, **HFI419** was optimized from a hit discovered by virtual screening.^23^**QHL-1** is a recently published IRAP inhibitor discovered by screening of a large 400,000 compounds.^24^ Another screening campaign led to the discovery of sulfonamide IRAP inhibitors such as **F-13**.^25,26^

As a member of the M1-aminopeptidase family, and closely related to ERAP enzymes, IRAP is inhibited by inhibitors targeting the protein family (Figure 2C). In particular, the nonselective aminopeptidase inhibitor **leucinethiol** inhibits IRAP at the low nanomolar level.^27^ Transition-state phosphinic pseudotripeptides have been reported as nanomolar inhibitors of ERAP/IRAP.^28^ Optimization of these compounds led to selective IRAP inhibitors such as **DG026**.^29^ Recently, α-hydroxy-β-amino acid derivatives of bestatin, a nonselective inhibitor of zinc aminopeptidases, were disclosed.^30^ The most potent inhibitor **B32e** was cocrystallized with IRAP.

**Figure 3:**
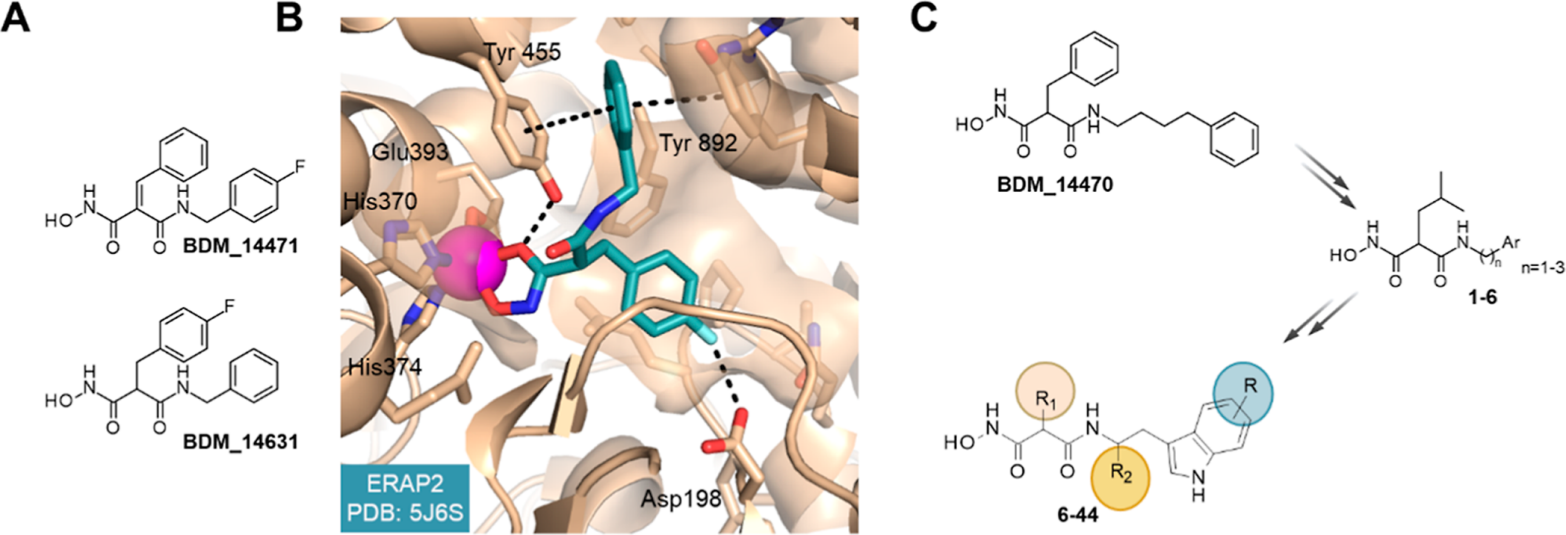
Malonic acid hydroxamates designed to target M1-aminopeptidases. (A) Inhibitors developed to inhibit *Pf*AM1 and/or *p*APN, 2 aminopeptidases of the M1 family (B) BDM_14631 (*S*-isomer) cocrystallized in hERAP2 (PDB: 5J6S). Oxygens, nitrogens are in red, blue, respectively. Carbons are colored beige and teal for hERAP2 and compound, respectively. Zinc ion is represented as a magenta sphere. Polar contacts and interactions are represented as black dashed lines. The structures were rendered using PyMOL Molecular Graphics System v2.5. (C) Two-step optimization of malonic acid hydroxamates providing inhibitors 1–44, highlighted positions of pharmacomodulation.

In the past, our research group developed a library of malonic acid hydroxamates to target the M1-aminopeptidase family.^31^ Subsequently, we discovered and optimized the first drug-like nanomolar inhibitors of *Plasmodium falciparum* aminopeptidase (*Pf*AM1) for the treatment of malaria, in particular **BDM_14471** and **BDM_14631** (Figure 3A, and Supporting Information Table S1).^32,33^ Although **BDM_14631** is a very weak inhibitor of ERAP enzymes (ERAP2 IC_50_ about 100 μM), it was found to cocrystallize with ERAP2 (Figure 3B).^34^ In this series, the selectivity within the M1 family, was shown to be dependent on the substituent on the malonic acid carbon (Supporting Information Table S1). Initial unpublished results indicate measurable activity against ERAP and IRAP particularly with analogues of **BDM_14843** and **BDM_14821**, which displayed an extended chain of 1 to 3 carbon atoms ending in an aromatic group (Ar) **1**–**6** (Figure 3C). This prompted us to explore the activity a larger set of analogues **6**–**44**.

Our initial goal was to develop families of protease inhibitors that modulate antigen presentation. Given the sequence and mechanism homology between the 3 main proteases involved in the trimming of peptide antigens, we set out to systematically profile all of our compounds against the three enzymes. This strategy allowed us to incorporate not only potency but also selectivity into the optimization process, and to better understand which of the three enzymes is/are involved in the antigen presentation. Thus, starting from ERAP1 inhibitors, we identified selective IRAP inhibitors with low nanomolar inhibition. A docking study allowed to explain the binding of the most potent compound **43** (**BDM_92499**) by a set of interactions different from those observed with bestatin analogues. Some selected inhibitors, such as **18** (**BDM_76464**) show a dose-dependent reduction of antigen cross-presentation in the low micromolar range and completely inhibit IRAP in cells.

## Results

### Chemistry

**Scheme 1:**
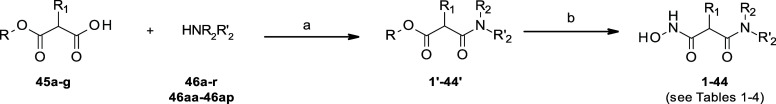
Synthetic Route of Inhibitors **1–44**, from Carboxylic Acids **45a**–**g** and Amines **46a–r**, **46aa–ap** Reagents and conditions: (a) HBTU or EDCI/HOBt, Et_3_N, DMF, rt, overnight; (b) hydroxylamine (50% aq solution), cat. KCN or DBU, rt, overnight.

Inhibitors **1**–**44** (Scheme 1) were obtained via coupling malonic acid monoester derivatives (**45a**–**g**) and commercially available (**46a**–**r**) or in-house synthesized amines (**46aa–ap**), followed by aminolysis of the ester to provide the hydroxamic acid, using either catalytic DBU^35^ or preferably catalytic KCN^36^ that avoids carboxylic acid or amide byproducts (Scheme 1).

**Scheme 2:**
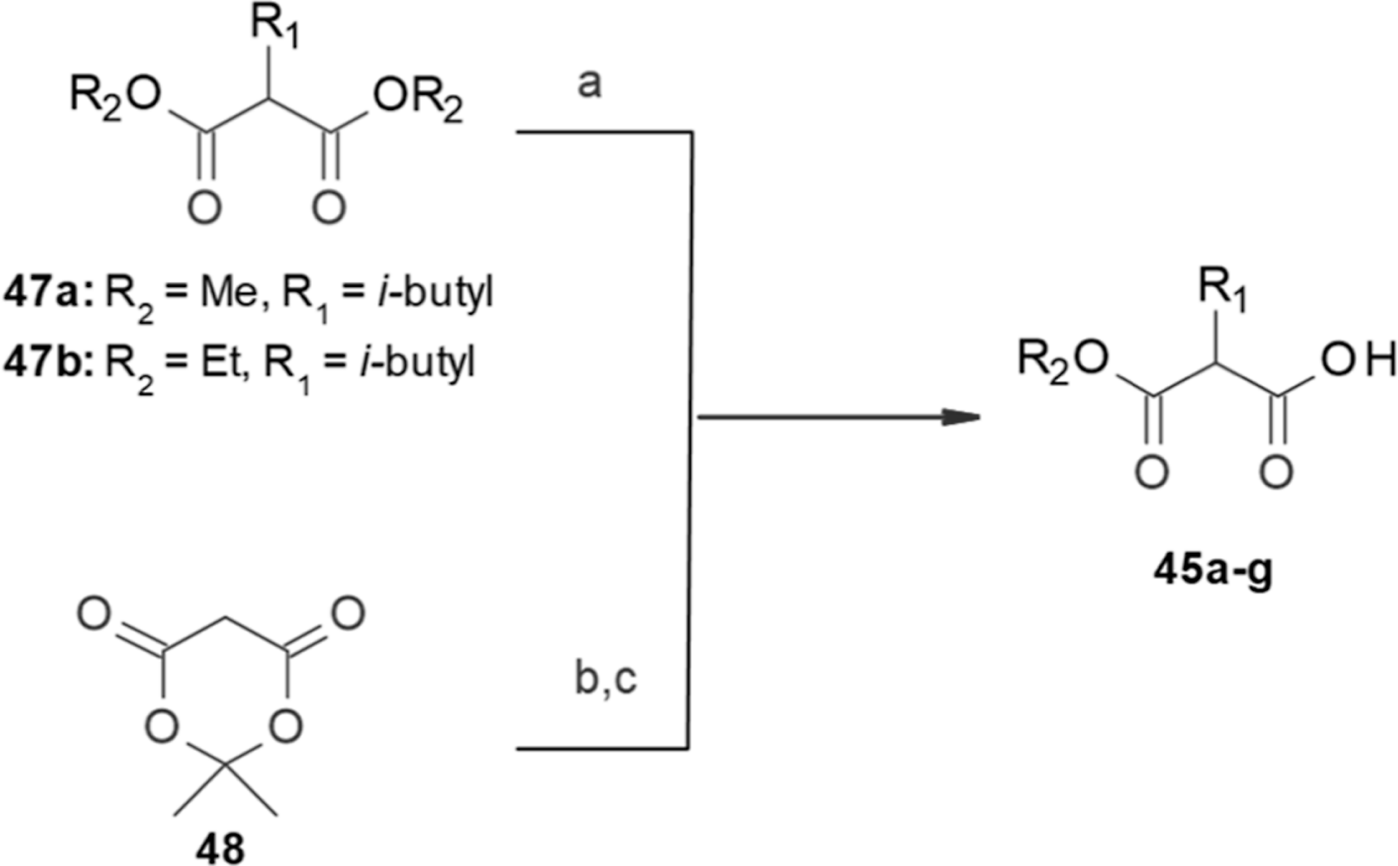
Synthetic Route of Carboxylic Acids **45a**–**g** Reagents and conditions: (a) KOH, MeOH, H_2_O, rt, 72 h or NaOH, EtOH, rt, overnight; (b) l-proline, EtOH, rt, 3 h, CH_3_COOH, NaBH_4_, 20 min, rt or ACN, rt, 1 h, Hantzsch ester, l-proline, EtOH, overnight, rt; (c) MW (100 °C, 1 h), MeOH.

Carboxylic acids (**45a**–**g**) were synthesized as depicted in Scheme 2 as racemates. Carboxylic acids **45a**–**b** were synthesized via a monosaponification of commercially available malonic diesters (**47a**–**b**). Carboxylic acids **45c**–**g** were obtained via a Knoevenagel reaction between Meldrum’s acid **48** and the corresponding aldehydes, followed by ring opening to afford desired compounds (Scheme 2) as described by Ramachary et al.^37^

**Scheme 3:**
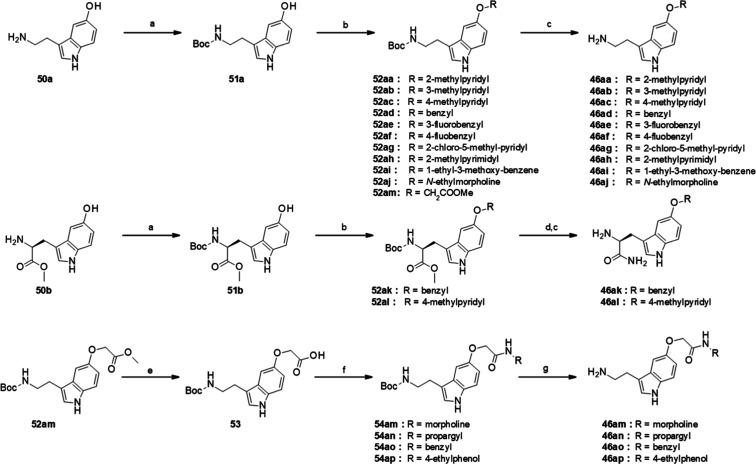
Synthetic Route of Amines **46aa–ap** Reagents and conditions: (a) Boc_2_O, CHCl_3_, NaHCO_3_, H_2_O, NaCl, Boc_2_O, reflux, 3 h, for **50b**: SOCl_2_, MeOH, overnight, then Boc_2_O, DCM, Et_3_N, overnight; (b) O-alkylation: R_2_-X, K_2_CO_3_ or Cs_2_CO_3_, ACN or DMF, rt; or Mitsunobu reaction: R_2_-OH, DIAD, PPh_3_, rt; (c) 4 N HCl in dioxane, MeOH, rt; Or TFA, 0 °C, 4 h; (d) 7 M NH_3_ in MeOH, 50–60 °C, 120 h, reflux, then 4 M HCl in dioxane; (e) MeOH, NaOH, H_2_O,overnight, rt; (f) HBTU or EDCI/HOBt, amine R_3_NH_2_, Et_3_N, DMF, rt, overnight; (g) 4 N HCl in dioxane, MeOH, rt.

While the amines **46a**–**r** were commercially available, the amines **46aa–aq** were synthesized as shown in Scheme 3. Serotonin **50a** was first N-protected by a Boc group and then substituted on the hydroxyl function either by nucleophilic substitution using a variety of halogenated derivatives (**52aa–af**; **52ai–aj**, **52am**),^38^ or via a Mitsunobu reaction (**52ag–ah**). Deprotection of the Boc group afforded the amines **46aa–aj**.

Similarly, l-5-hydroxy-tryptophan methyl ester **50b** was N-protected by a Boc group (**51b**) and then substituted on the hydroxyl function by nucleophilic substitution followed by conversion to primary amides **52ak–al** using 7 M ammonia in methanol at 60 °C and N-deprotection to give amines **46ak–al**.

Finally, the intermediate **52am**, bearing a methyl ester at position R_2_, was saponified to give the corresponding carboxylic acid **53**, which was reacted with various amines to give amides **54am–ap**, which were then deprotected to give amines **46aa–ap**.

### Structure–Activity Relationships

Early SAR around **BDM_14471** showed that the presence of an alkyl group on the malonic carbon and an extended chain on the amide allowed the first activities on ERAP1 in the series. Compounds in this series carry a chiral malonic carbon, and have been tested as racemic mixtures. It is challenging to separate this type of enantiomers and therefore malonamides with a single substituent on the malonic carbon are evaluated as racemic mixtures.^31−33,39,40^ Early analogues **1**–**9** (Table 1) bearing an *iso*-butyl group showed micromolar inhibition of ERAP1. *meta*-Biphenyl analogue **1** showed equivalent activity on all three enzymes while its *para*-analogue **2** is not active on ERAP2. Imidazole analogues **4**–**5** were more potent than **BDM_14471**, with the shorter chain length more selective for ERAP1 and IRAP and the longer chain length more selective for IRAP. Indole analogue **6** was selective for IRAP with a submicromolar activity (IC_50_ = 0.65 μM). Cyclization into 2,3,4,5-tetrahydro-1*H*-pyrido[4,3-*b*]indole (**3**) was detrimental to activity on ERAP1 and IRAP but provided low micromolar inhibition of ERAP2 (IC_50_ = 1.3 μM). In an effort to improve the potency of **6**, we replaced the *iso*-butyl moiety with other alkyl chains. The introduction of an *iso*-pentyl group (**7**) had no strong effect on activity (IC_50_ = 0.61 μM) and selectivity. Similar results were observed with the phenethyl substituent (**9**). However, substitution of *iso*-butyl with 2,2-dimethylbutane (**8**) improved activity on IRAP (IC_50_ = 0.34 μM) and selectivity toward ERAP1 (>2-log).

**Table 1:**
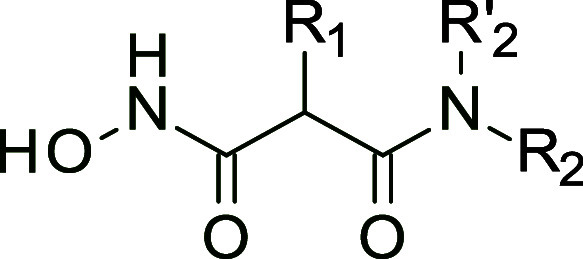

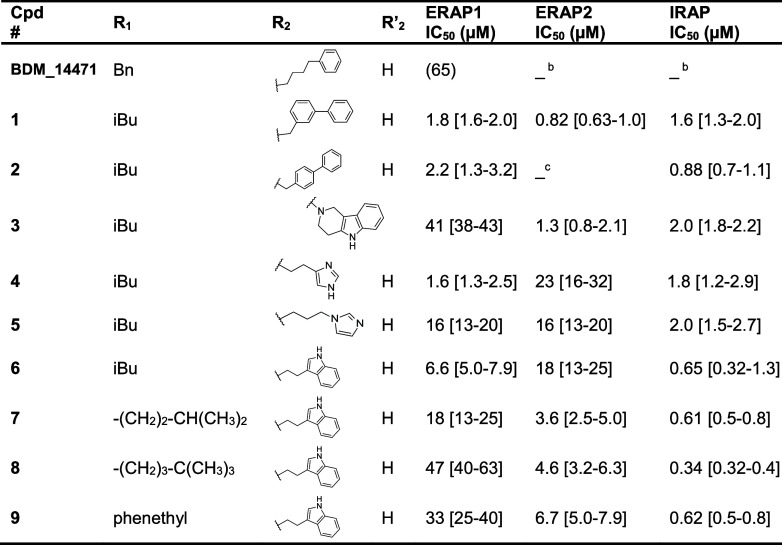
Activities of Analogues **1–9**^a^

a All inhibitors were tested as racemic mixtures. IC_50_ values were calculated from dose–response inhibition curves and represent the mean value of three independent measurements. Substrates l-leucine-7-amido-4-methylcoumarin (L-AMC) for ERAP1 and IRAP; l-arginine-7-amido-4-methylcoumarin (R-AMC) for ERAP2.

b Not determined.

c % inhibition@100 μM < 30%.

These results indicate that an indole ring at the R_2_ position, combined with an alkyl chain at the R_1_ position, confers submicromolar activity against IRAP with good selectivity against ERAP1 and ERAP2. To further improve the binding affinity and selectivity of the indole series, several optimizations were performed. We chose to optimize compounds **6** and **7**, bearing an *iso*-butyl and an *iso*-pentyl group respectively, as these compounds showed favorable lipohilic ligand efficiency (LLE) values compared to **8** and **9** (Supporting Information Table 2).

First, the activities of analogues of **6** (Table 2) were studied. Incorporation of halogens (F, Cl, Br) or methyl, methoxy and hydroxy groups at positions 5 or 6 of the indole ring (**10**–**17**) maintains low micromolar (**16**–**17**) or even submicromolar (**10**–**15**) activity against IRAP. Inhibitor (**11**) with a fluorine in position 6, is the best inhibitor (IC_50_ = 0.23 μM). Brominated analogue **13** also has submicromolar activity against ERAP1. All analogues in this subseries are nonselective for all three enzymes.

**Table 2:**
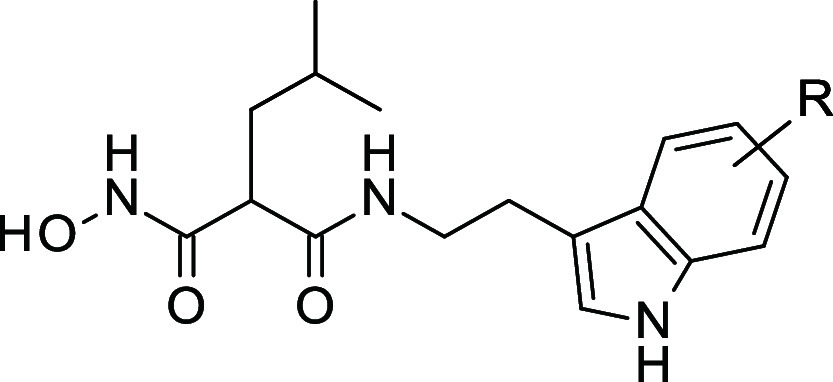

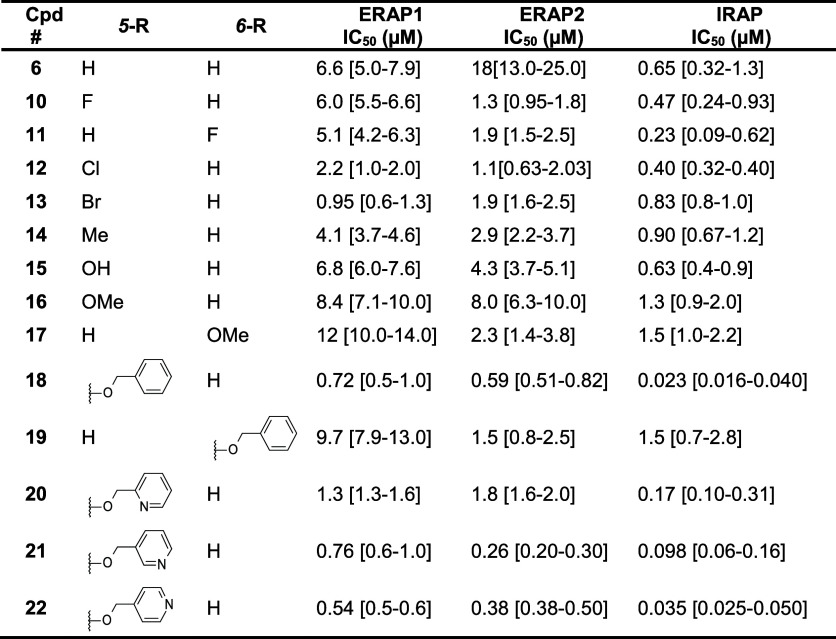
Activities of *iso*-Butyl Analogues **10–22**^a^

a All inhibitors were tested as racemic mixtures. IC_50_ values were calculated from dose–response inhibition curves and represent the mean value of three independent measurements. Substrates l-leucine-7-amido-4-methylcoumarin (L-AMC) for ERAP1 and IRAP; l-arginine-7-amido-4-methylcoumarin (R-AMC) for ERAP2.

Introduction of a benzyloxy group at position 5 (**18**) resulted in submicromolar activity on ERAP1, and ERAP2, and nanomolar activity on IRAP (IC_50_ = 23 nM). However, introduction of a benzyloxy group at position 6 (**19**) resulted in loss of activity against all three enzymes.

Isosteric replacement of the phenyl by a pyridine (**20**–**22**) at position 5 selectively improved the potency against IRAP. In particular, **22**, bearing a 4-pyridylmethoxy group, showed nanomolar activity (IC_50_ = 35 nM) with moderate selectivity against ERAP1 (8-fold) and ERAP2 (6-fold).

We investigated the effects of substitution on the 5-hydroxyindole group of the isopentyl inhibitor **7** with compounds **23**–**36** (Table 3). While introduction of a benzyl group to **6** gave a submicromolar inhibitor of all three enzymes **18**, the introduction of a benzyl group to **7** gave a submicromolar selective ERAP2 inhibitor (**23**). In this series, substitution of the phenyl (**24**–**25**) or isosteric replacement by a pyridine (**26**–**39**) afforded submicromolar dual ERAP2/IRAP inhibitors. In the iso-pentyl series, the best pyridine inhibitor is the ortho-substituted analogue **26**, whereas in the iso-butyl series it is the para-substituted analogue (**22**). Both the introduction of a pyrimidine (**30**) and the extension of the benzyl chain to a phenethyl chain (**31**) led to a 1-log loss of activity on ERAP2, while the activity on IRAP was retained. Analogues **32**–**36** show good to excellent activity on both ERAP2 and IRAP, demonstrating that substitution at this position by groups of various sizes and polarities is tolerated by both enzymes but is detrimental to activity on ERAP1.

**Table 3:**
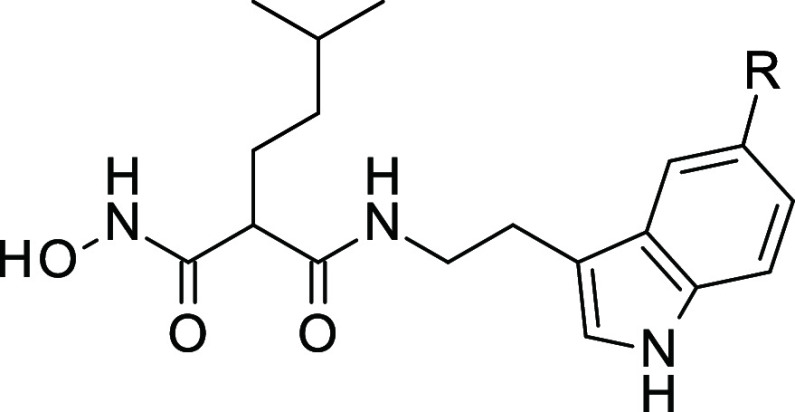

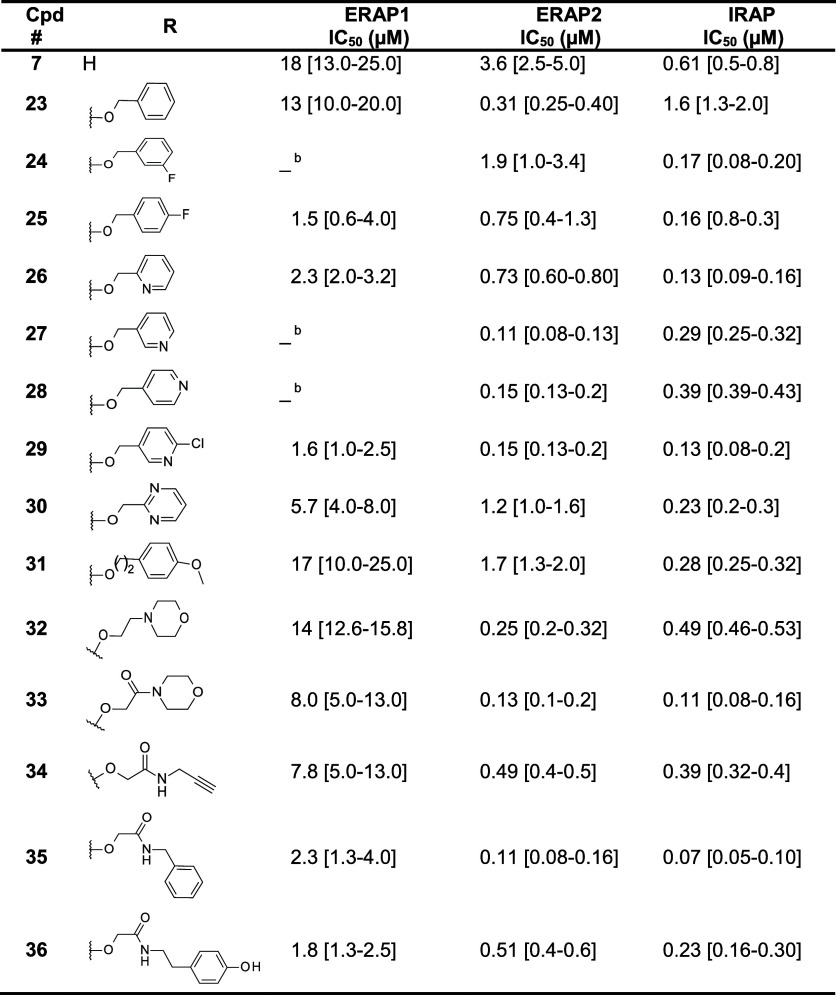
Activities of *iso*-Pentyle Analogues **23–36**^a^

a All inhibitors were tested as racemic mixtures. IC_50_ values were calculated from dose–response inhibition curves and represent the mean value of three independent measurements. Substrates l-leucine-7-amido-4-methylcoumarin (L-AMC) for ERAP1 and IRAP; l-arginine-7-amido-4-methylcoumarin (R-AMC) for ERAP2.

b % inhibition@100 μM < 30%.

Since *iso*-butyl derivatives gave the best activities and selectivities, we further explored this subseries (Table 4). First, considering that some inhibitors of ERAP enzymes and/or IRAP from the phosphinic or bestatin series, contain a terminal α-amino amide or ester such as Trp-OMe or Phe-NH_2_, we studied the effect of a substituent at position R_2_ in **6**. Analogues (**37**–**40**) bearing either a methyl, a hydroxymethylor a carbamoyl group, retain activity on IRAP. **38**, bearing an alcohol side chain in S-configuration, shows an IC_50_ of 0.16 μM on IRAP and is selective versus ERAP1 and ERAP2. We then evaluated cyclized analogues, changing the isobutyl to a tetrahydropyrane or a cyclohexyl group (**41**–**42**). This greatly improves both potency and selectivity for IRAP. In particular **42** is active in the low nanomolar range (IC_50_ = 10 nM) and is highly and moderately selective against ERAP1 (2 log) and ERAP2 (1 log) respectively. The cyclohexyl analogue **41** is highly active on both ERAP2 (IC_50_ = 34 nM) and IRAP (IC_50_ = 28 nM). Finally, we studied the combination of the substitution on the 5-hydroxy-indole and the substitution at position R_2_ (**43**–**44**). In particular, for the analogues with a benzyloxy group at position 5 of the indole ring, the introduction of the (S)-carbamoyl moiety improved the potency by 2-log on IRAP (**43** vs **18**). Compound **43** exhibited the highest IRAP inhibitory activity, with an IC_50_ of 3.4 nM, and remarkable selectivity against ERAP1 (>2 log) and ERAP2 (>3 log). Taken together, these structure–activity relationship studies enabled the discovery of a highly potent and selective IRAP inhibitor within the malonic acid chemical series.

**Table 4:**
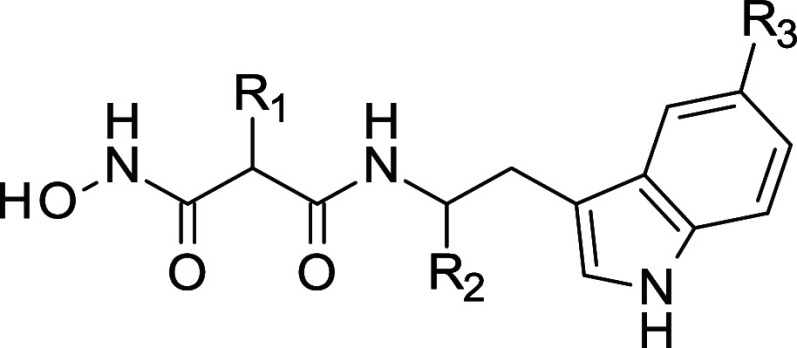

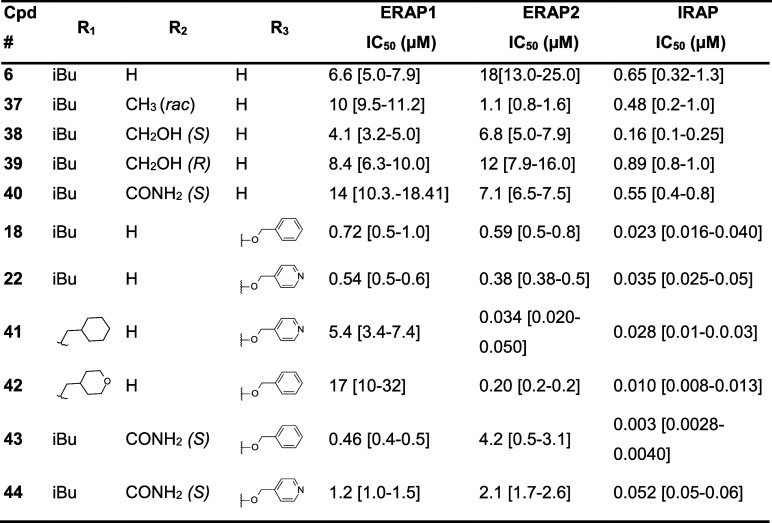
Activities of Analogues **37–44**

a IC_50_ values were calculated from dose–response inhibition curves and represent the mean value of three independent measurements. Substrates l-leucine-7-amido-4-methylcoumarin (L-AMC) for ERAP1 and IRAP; l-arginine-7-amido-4-methylcoumarin (R-AMC) for ERAP2. **6**, **18**, **22**, **37**, **41–42** were tested as racemic mixtures. **38–40**, **43–44** were tested as 50/50 mixture of 2 diasteroisomers.

### Docking Studies

**Figure 4:**
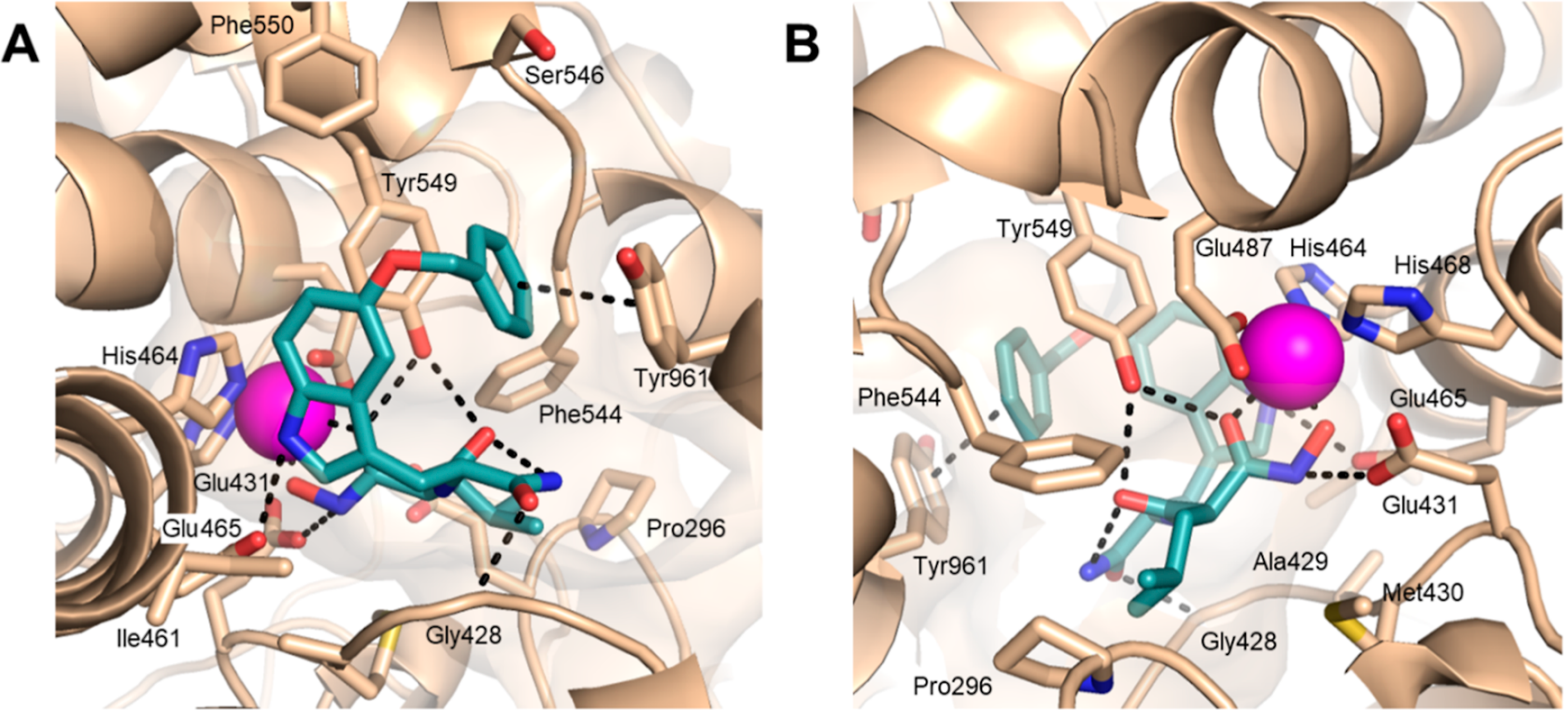
Putative binding of **43** (*S*,*S* isomer) in hIRAP. (A,B) Two different views of the docking pose of **43** (*S*,*S* isomer) in hIRAP (PDB: 7ZYF); oxygens, nitrogens are in red, blue, respectively. Carbons are colored beige and teal for hIRAP and **43** respectively. Zinc ion is represented as a magenta sphere. Polar contacts and interactions are represented as black dashed lines. The structures were rendered using PyMOL Molecular Graphics System v2.5.

**Figure 5:**
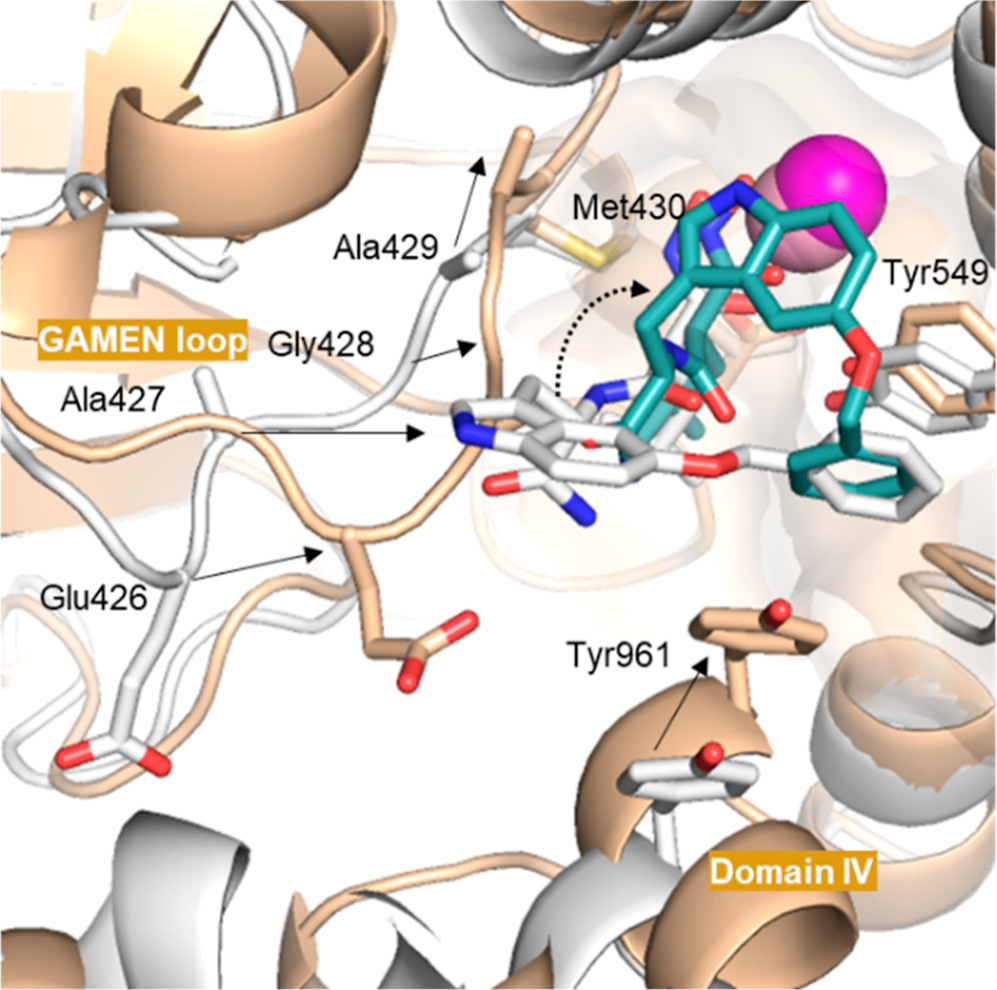
Comparison of binding of **43** (*S*,*S*) in hIRAP semiopen and closed conformation. Superimposition of the docking pose of **43** (*S*,*S* isomer) in closed hIRAP (PDB: 7ZYF) or semiopen (PDB: 4Z7I) conformation. IRAP is colored light orange (closed) or light gray (semiopen). Oxygens, nitrogens, sulfurs are in red, blue and yellow, respectively. Carbons are colored white and teal for **43** in semiopen and closed conformations, respectively. Zinc ions are represented as pink/magenta spheres. Shifts of GAMEN loop and Domain IV are represented by black arrows. Reorientation of the indole group is depicted by a dotted black arrow. The structures were rendered using PyMOL Molecular Graphics System v2.5.

To further rationalize the excellent inhibition of IRAP by **43**, a docking study of this inhibitor was performed. **43** contains equal amounts of both *RS* and *SS* diastereoisomers. In hIRAP, the hydroxamate function of **43** (*SS*-isomer) chelates the zinc ion and forms hydrogen bonds with Tyr549 and Glu431 (Figure 4). The isobutyl group is adjacent to the hydrophobic residues Phe544 and Pro296. The carbonyl group of the primary amide forms a hydrogen bond with the NH of Gly428 of the GAMEN loop while the NH_2_ is involved in a network of hydrogen bonds with both the carbonyl group of the malonic moiety and the phenol group of Tyr549. The indole allows an optimal orientation of **43**. The indole NH forms a hydrogen bond with Glu465 and the 5-benzyloxy substituent pi-stacks with both Tyr961 and Tyr549. The multiple interactions explain the high potency of **43** and are consistent with the structure–activity relationships that emphasized the importance of the primary amide and the substituent at the 5-position of the indole as key determinants of potency. This binding also explains the selectivity of **43** for IRAP over ERAP1 and ERAP2. While many amino-acids interacting with **43** are conserved in the other two enzymes, Tyr961, replaced by Ser869 in ERAP1 and Arg895 in ERAP2, is unique to IRAP. **43** (*SS*-isomer) was also docked in the semiopen conformation of IRAP (Supporting Information Figure S1).^41^ In this conformation, the hydroxamic acid function interacts with the zinc ion, Tyr549 and Glu431. The NH of the malonamide forms a hydrogen bond with the backbone of Ala429. The stacking between Tyr549 and the phenyl group is conserved. The indole is close to the GAMEN loop but is not involved in specific interactions. The primary amide no longer interacts with the GAMEN loop. No interaction is observed between **43** (*SS*-isomer) and domain IV of the enzyme, specifically with Tyr 961. Comparison of the binding of **43** (*SS*-isomer) in the semiopen and closed forms of IRAP (Figure 5) suggests that the movement of the GAMEN loop and domain IV upon closure forces the indole to reorient itself, allowing the insertion of the phenyl group between Tyr549 and Tyr961, thus stabilizing the closed conformation.

In strong contrast to the *SS* isomer, the *RS* isomer adopts a different binding orientation in IRAP (Supporting Information Figure S2). The hydroxamic acid still chelates the zinc ion and interacts with Tyr5749, and Gly428 forms a hydrogen bond with the primary amide group. The carbonyl of the malonamide forms a hydrogen bond with the Ala429 backbone (instead of Tyr549) and the indole nitrogen interacts with Glu426 (instead of Glu465). Importantly, due to the orientation of this isomer, Tyr549 does not interact with the primary amide group and all pi-stacking interactions are lost. This suggests that the *RS* isomer is the least active isomer of **43**.

### Cellular Activities

Potent compounds **18**, **22**, **41**–**43**, and early analogue **12** did not show cytotoxicity at the highest concentration tested (Supporting Information Table 3). Their potency and efficacy were measured in a cellular model of antigen cross-presentation. The HEK-K^b^ cells used express an F_c_γ receptor for internalization and cross-presentation of ovalbumin. The ovalbumin epitope SIINFEKL presented on the cell surface is recognized by the B3Z T-cell hybridoma. Upon recognition of the cognate peptide, the T-cells are activated and produce IL-2. In this system, cross-presentation is primarily dependent on the enzymatic activity of IRAP; IRAP−/− cells were used as controls.

**Figure 6:**
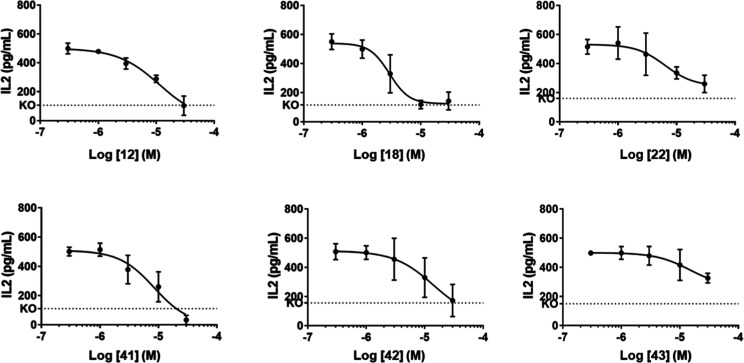
Inhibition of antigen cross-presentation by selected inhibitors. Dose-dependent reduction of IL-2 dosed from the supernatants of cultures of wt (solid line) and IRAP−/− HEK293T FcR-EGFP.Kb (KO, dotted line) cells exposed to various compounds or vehicle and cross-presenting OVA/anti-OVA immune complexes to B3Z. Data are mean of four independent experiments ± SD.

In this assay, all inhibitors show dose-dependent micromolar inhibition of the cross-presentation of immune complexes by IRAP+/+ cells (Table 5, Figure 6). In particular compounds **18**, **22** and **41** are the most potent inhibitors in cells with single digit micromolar IC_50_s. No effect is observed on IRAP−/− cells, confirming that this effect is IRAP dependent. At the highest concentration, all inhibitors except **43** block cross-presentation with the same efficacy as IRAP knockout, i.e. they fully inhibit IRAP, at the highest concentration (30 μM).

**Table 5:**
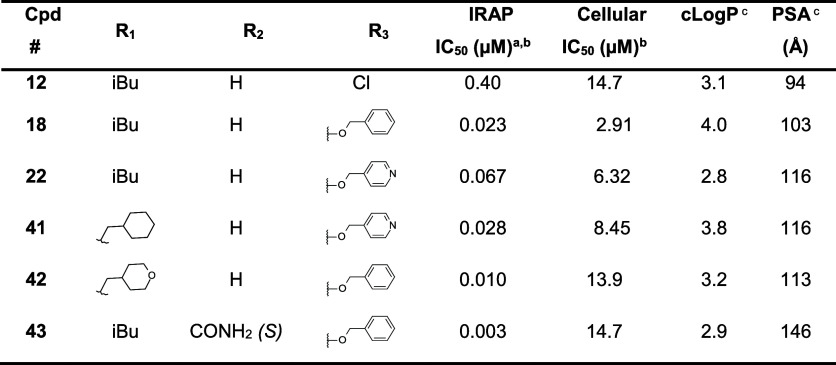
Effect of Inhibitors **12**, **18**, **22** and **41–43** on Antigen Cross-Presentation^a^

a IC_50_ values were calculated from dose–response inhibition curves and represent the mean value of three independent measurements with l-leucine-7-amido-4-methylcoumarin (L-AMC) as substrate.

b IC_50_ values were calculated from dose–response inhibition curves of the antigen cross-presentation measured as the decrease in production of IL-2 by B3Z hybridoma. Data represent the mean value of four independent measurements.

c ALog *P* and PSA (polar surface area) were calculated using embedded functions of BioviaDraw.

## Discussion and Conclusions

We have successfully optimized compounds inspired by previous M1-aminopeptidase inhibitors for their activity against IRAP. In particular, in the *iso*-butyl series, the introduction of a 5-hydroxytryptamine further substituted by benzyl groups or isosters allowed to obtain low nanomolar activities. Combining this modification with substitution on the alkyl linker with a carbamoyl group allowed **43** (**BDM_92499**, IC_50_ = 3.4 nM) with optimal selectivity toward ERAP1 and ERAP2. The binding study rationalized the potency and selectivity of this compound and suggested that the binding mode of **43** is different from the bestatin or phosphinic analogues (Supporting Information Figure S3), illustrating the originality of the series. Our inhibitors were tested as racemates (**1–36**, **41–42**) or as a binary mixture of 2 diastereoisomers in the same amount (**38–40**, **43–44**) due to the presence of exchangeable hydrogen on the malonic carbon (Supporting Information Figure S4). However, the docking study on **43** suggests that the preferred configuration of this carbon would be S, which is consistent with the configuration of the isomer of precursor **BDM_14631**, which was found to cocrystallize with the closely related enzyme ERAP2 from a racemic mixture.^34^ Docking within the semiopen conformation of IRAP also suggested a strong influence of the GAMEN loop and the Tyr961 position in the binding of **43**. The separation of enantiomers or diastereomers in malonamides that are monosubstituted on the malonic carbon is still challenging and this carbon is stereochemically unstable. Therefore, the next round of medicinal chemistry should investigate controlling the geometry of the carbon vicinal to the hydroxamic acid by quaternarization or homologation. Several inhibitors in the series showed a significant dose-dependent inhibition of antigen cross-presentation in cells to the level of a complete genetic IRAP inhibition. However, the cellular potency of some inhibitors may be limited by their moderate membrane permeability (Caco-2 and BBB clog *P*_app_, Supporting Information Table 4). For example, although **43** is the most potent inhibitor in the series, its higher polarity (clog *P* = 2.9; PSA = 146 Å; HBD = 5; HBA = 5) reduces its ability to enter cells compared to **18** (**BDM_76464**), which has the highest clog *P* (4.0) and one of the lowest PSA (103 Å) and shows the best compromise between potency on IRAP (IC_50_ = 23 nM) and on the cross-presentation assay (IC_50_ = 2.91 μM). Further optimization in the series will therefore also focus on improving cell penetration by fine-tuning physicochemical properties (HBA, HBD, clog *P*).

Overall, these potent and selective malonic acid hydroxamate IRAP inhibitors, are the first series of M1-aminopeptidase-derived inhibitors to completely inhibit IRAP in cells and complement the arsenal of selective ERAP2^42^ or ERAP1^43,44^ inhibitors for exploring the respective roles of these 3 enzymes in antigen processing and presentation.

## Experimental Section

### Biology

#### Dose Response Experiments on Enzymes

The enzymatic activity was assayed using L-AMC (l-leucine 7-amido-4-methylcoumarin, Sigma) for ERAP1 and IRAP and R-AMC (l-arginine-7-amido-4-methylcoumarin hydrochloride, Sigma) for ERAP2. Hepes at 50 mM with 100 mM NaCl at pH 7 was used as buffer. Recombinant hERAP1 (PILS/ARTS1 from R&D Systems, ref 2334-ZN-010), recombinant hERAP2 (prepared as previously described^45^) and recombinant hIRAP (Gift from E. Stratikos, Greece) were used at final concentrations of 0.8, 1 and 0.2 μg/mL respectively. Briefly, 60 nL of test compounds were added in 384-wells plates (dark, nonbinding surface) by acoustic dispensing with Echo (Labcyte) and preincubated 30 min at ambient temperature with 10 μL of enzyme at the above final concentration or vehicle. The reaction was then started with the addition of 10 μL of substrate at 10 μM. The final concentration of ERAP2, substrate and DMSO was 0.5 μg/mL, 5 μM and 0.4% respectively. For the kinetic readout a Victor 3 V (PerkinElmer) was used with excitation at 380 nm and emission at 450 nm. The fluorescence was measured each 3 min during 1 h. Percentages of inhibition or activation at different concentrations were obtained as for screening. All measurements were carried out as 8-point dose response curves and reported as the average of at least three independent measurements. Bestatin was used as a reference inhibitor (100% inhibition at 2 mM). Data analysis was performed using Xlfit v 5.0 or GraphPad Prism v 4.0. Nonlinear curve fitting and statistical analysis was done using built-in functions.

#### Cell Lines

HEK293T cells expressing the mouse MHC class I molecule H-2K^b^ and the human FcγRIIA receptor fused to an eGFP tag (293T FcR-EGFP.K^*b*^), were kindly provided by Cresswell (Yale University).^46^*IRAP*^*−/−*^ 293T FcR-EGFP.K^*b*^ were generated by knocking-out IRAP gene using CRISPR-Cas9 technology. WT and *IRAP2*^*−/−*^ 293T FcR-EGFP.K^*b*^ cells were cultured in Gibco DMEM high-glucose Glutamax medium (Fisher Scientific) + 10% heat-inactivated fetal bovine serum (Eurobio) + 1% penicillin/streptomycin (Sigma-Aldrich), 5% CO_2_, 37 °C, in T75 Nunc flasks. B3Z hybridoma (a gift from Shastri^47^) were cultured in RPMI-1640 medium (Merck) + 10% heat-inactivated fetal bovine serum (Eurobio) + 2 mM l-glutamine (Merck) + 1% penicillin/streptomycin, 50 μM β-mercaptoethanol (Gibco) + 25 mM HEPES (Thermo Fisher Scientific) + 1× non-essential amino acids (Gibco), 1 mM sodium pyruvate (Thermo Fisher Scientific), 5% CO_2_, 37 °C.

#### In Vitro Antigen Cross-Presentation Assay

Ovalbumin (OVA, Worthington) was complexed to anti-OVA polyclonal antibodies (Polyscience) according to published procedure^46^ and precipitated OVA/anti-OVA complexes were used as antigen for cross-presentation assays. WT and *IRAP*^*−/−*^ 293T FcR-EGFP.K^*b*^ were plated into a 48-well culture plate (40,000–50,000 cells/well) with flat bottoms (Nunc) in culture medium. After 2 h, serial dilutions (30, 10, 3, 1, 0.1 μmol/L) of compounds or DMSO (vehicle, Merck) were added for 60 min to the wells. Then precipitated OVA/anti-OVA complexes diluted into Gibco DMEM high-glucose Glutamax medium (Fisher Scientific) + 1% heat-inactivated fetal bovine serum (Eurobio) + 1% penicillin/streptomycin (Sigma-Aldrich), 5% CO_2_, 37 °C were provided to the cells, in the presence of the inhibitor or vehicle (DMSO) for 17–19 h. As a positive control, serial dilutions of synthetic SIINFEKL peptide were provided to the cells in the presence of compounds (30 μmol/L) or vehicle. At the end of incubation, cells were washed two times in PBS before the addition of B3Z hybridoma (60,000–75,000 cells/well, ration 1.5:1) in culture medium for another 24 h. Culture supernatants were frozen at −20 °C for up to 7 days before dosing IL-2 by a sandwich ELISA technique. In brief, Nunc Maxisorp ELISA plates (Nunc) were coated overnight with purified anti-IL2 antibody (clone JES6-1A12, Biolegend, 2 μg/mL) diluted in PBS (50 μL/well). Coating solution was discarded, and plates were blocked with a PBS 10% fetal bovine serum (200 μL/well) for 30 min at room temperature. After discarding blocking solution, supernatants or serial dilutions of mouse IL-2 recombinant protein (Peprotech, Thermo Fisher Scientific) (100 μL/well) were added for 60–90 min to the plates at room temperature, with gentle shaking (300 rpm). Plates were then washed twice with PBS Tween20 0.05% (300 μL/well), and biotin-conjugated anti-IL2 antibody (clone JES6-5H4, Biolegend, 2 μg/mL) diluted in PBS (50 μL/well) was incubated for 45 min at room temperature with gentle shaking (300 rpm). After 5 washes in PBS Tween20 0.05% (300 μL/well), HRP-streptavidin (BD Biosciences, 1:2000) diluted in PBS was added for 30 min (500 μL/well) at room temperature with gentle shaking (300 rpm). After 5 washes in PBS Tween20 0.05% (300 μL/well), TMB substrate (Cell Signaling Technology) reagent was added (100 μL/well) for 5–10 min at room temperature to reveal bound cytokine and reaction was blocked by adding HCl 2 N (Merck) solution (20 μ L/well). The absorbance at 450 nm was read using a Mithras LB940 reader (Berthold Technologies). Each condition was performed in duplicate. IL-2 OD readings for OVA-incubated cells were corrected for background values obtained with BSA-incubated cells, before their conversion into IL2-concentrations based on the linear curve of mouse recombinant IL-2 standards. The results are presented as the means of 4 independent experiments.

#### Cytotoxicity Assays

Cytotoxicity of compounds was measured at 30 μM on HEK293T FcR-EGFP.Kb and HEK293 by several methods. (1) IRAP−/− or WT HEK293T FcR-EGFP.Kb cells were cultured with compounds or DMSO for 16 h in culture medium. After detaching and extensive washing in PBS bovine serum albumin 2%, cells were stained for 7-aminoactinomycine D (7-AAD) (Biolegend) and fluorescence was recorded on a BD LSR Fortessa flow cytometer equipped with 5 lasers. Data were analyzed on FlowJo v10 software and dead cells were identified and quantified by gating on cells positive for 7-AAD. (2) HEK-293 cells were seeded in a μClear black 384-well plate (Greiner) at 10,000 cells per wells in 30 μL for 24 h at 37 °C, 5% CO_2_. The next day, compounds were added to the plate using an Echo550 liquid handler (Labcyte), then 20 μL of medium supplemented with Hoescht 33342 (nuclei staining), Nucview 488 (apoptosis staining) and propidium iodide (PI, necrosis staining) were dispensed into the wells to obtain a final concentration of 40 ng/mL, 0.8 μM and 1 μg/mL respectively. The final concentration of the compounds is 30 μM with 0.3% of DMSO as the vehicle. Images were acquired the INCell 6000 high-resolution automated microscope. Six fields per well were read with the 20× objective after 24 h of incubation at 37 °C, 5% CO_2_. Images were analyzed with Columbus software (PerkinElmer). Hoechst 33342 staining was used to detect and quantify nuclei, in which the mean fluorescence intensity of NucView 488 and propidium iodide was quantified. A threshold based on the negative control is determined to separate positive from negative cells. For each well, a percentage of propidium iodide (necrosis) and Nucview (apoptosis) positive cells in the total cell population is calculated. Values represent the mean and standard deviation of 3 technical replicates.

#### Docking and Physicochemical Calculations

Modeling and simulations were performed using GOLD 2021.3.0 (Cambridge Crystallographic Data Centre (CCDC)) on X-ray crystal structure of hIRAP obtained from the Protein Data Bank (http://www.rcsb.org): PDB: 7ZYF; closed protein conformation in complex with bestatin analogue B32e or PDB: 4Z7I semi open conformation in complex with DG025. Before the analysis, ligands, solvents and proteins were protonated according to default settings in GOLD. The binding site radius was set to default 10 Å (around B32e). The built-in scoring function ChemPLP was applied for docking. The predicted binding were selected based on the docking score and the binding to zinc PLP.Chemscore.Metal and visual inspection. The structures were rendered in PyMOL (Delano, W. L. The PyMOL Molecular Graphics System. DeLano Scientific LLC: San Carlos, CA, 2002). clog *P* and PSA were calculated using OSIRIS Datawarrior. Caco-2 clog *P*_app_ and BBB clog *P*_app_ were calculated using the EnalosSuite (Supporting Information Methods).

### Chemistry

#### General Information

Solvents for synthesis, analysis and purification were purchased as analytical grade from commercial suppliers and used directly without further purification. Chemical reagents were purchased as reagent grade and used without further purification. LC–MS Waters system was equipped with a 2747 sample manager, a 2695 separations module, a 2996 photodiode array detector (200–400 nm) and a Micromass ZQ2000 detector (scan 100–800). XBridge C18 column (50 mm × 4.6 mm, 3.5 μm, Waters) was used. The injection volume was 20 μL. A mixture of water and acetonitrile was used as mobile phase in gradient-elution. The pH of the mobile phase was adjusted with HCOOH and NH_4_OH to form a buffer solution at pH 3.8. The analysis time was 5 min (at a flow rate at 2 mL/min), 10 min (at a flow rate at 1 mL/min) or 30 min (at a flow rate at 1 mL/min). Purity (%) was determined by reversed phase HPLC, using UV detection (215 nm), and all isolated compounds showed purity greater than 95%. HRMS analysis was performed on a LC–MS system equipped with a LCT Premier XE mass spectrometer (Waters), using a XBridge C18 column (50 mm × 4.6 mm, 3.5 μm, Waters). A gradient starting from 98% H_2_O 5 mM ammonium formate pH 3.8 and reaching 100% CH_3_CN 5 mM ammonium formate pH 3.8 within 3 min at a flow rate of 1 mL/min was used. NMR spectra were recorded on a Bruker DRX-300 spectrometer and Bruker NMR Spectrometer DRX-500. The results were calibrated to signals from the solvent as an internal reference [e.g., 2.50 (residual DMSO-*d*_6_) and 39.52 (DMSO-*d*_6_) ppm for ^1^H and ^13^C NMR spectra respectively]. Chemical shifts (δ) are in parts per million (ppm) downfield from tetramethylsilane (TMS). The assignments were made using one-dimensional (1D) ^1^H and ^13^C spectra and two-dimensional (2D) HSQC-DEPT, COSY and HMBC spectra. NMR coupling constants (*J*) are reported in Hertz (Hz), and splitting patterns are indicated as follows: s for singlet, br s for broad singlet, d for doublet, t for triplet, q for quartet, quin for quintet, dd for doublet of doublet, ddd for doublet of doublet of doublet, dt for doublet of triplet, qd for quartet of doublet, m for multiplet, δ for chemical shift, *J* for coupling constant. Flash chromatography was performed using a Puriflash430 with silica columns. UV and ELSD detection were used to collect the desired product. Reverse flash chromatography was performed using a CombiFlash C_18_ Rf200 with C_18_ silica gel cartridges. UV detection (215 and 254 nm) was used to collect the desired product.

Preparative HPLC was performed using a Varian PRoStar system with an OmniSphere 10 μm column C_18_ Dynamax (250 mm × 41.4 mm) from Agilent Technologies. A gradient starting from 20% MeCN/80% H_2_O/0.1% formic acid and reaching 100% MeCN/0.1% formic acid at a flow rate of 80 mL/min was used. UV detection (215 and 254 nm) was used to collect the desired product. Purification yields were not optimized.

The specific optical rotation of compound **43** was measured using an Anton Paar MCP 5100 polarimeter (Anton Paar GmbH, Graz, Austria). Measurements were performed at 20 °C using the sodium d-line wavelength (589 nm) and a UV wavelength (365 nm). The compound was dissolved in DMSO-*d*_6_ at a concentration of 5.0 g/L. The polarimeter was calibrated according to the manufacturer’s specifications, ensuring valid measurements. Specific rotation values were recorded and reported under dry conditions.

#### Procedure A: General Procedure for Knoevenagel Reaction

Meldrum’s acid (1.0 equiv), Aldehyde (1.0 equiv) and l-proline (0.3 equiv) were mixed in EtOH and stirred at rt for 3 h. Acetic acid was then added, followed by sodium borohydride (3.0 equiv). The resulting mixture was stirred for 20 min and solvents were evaporated under reduced pressure. The resulting crude product was purified by column chromatography.

#### Procedure B: General Procedure for Ring Opening

Substituted Meldrum’s acid (1.0 equiv) was dissolved in MeOH in a microwave-adapted vial. The reaction was submitted to microwave irradiations during 1 h at 100 °C. The solvent was removed under reduced pressure.

#### Procedure C: General Procedure for Monosaponification

To the solution of compound **48** (1.0 equiv) in MeOH or EtOH was added KOH or NaOH (1.04 equiv) in water. The resulting solution was stirred at rt. Solvents were evaporated under reduced pressure and the residue was dissolved in 0.1 M Na_2_CO_3_ (aq) solution. The aqueous layer was washed with DCM or EtOAc and acidified by HCl solution (1 M) until pH = 1. The aqueous solution was then extracted twice with EtOAc. The combined organic layers were dried with MgSO_4_, filtered and concentrated under reduced pressure to afford the desired product.

#### Procedure D: General Procedure for O-Alkylation

*N*-Boc-Serotonin (1.0 equiv), halogenated derivatives (1.05 equiv) and K_2_CO_3_ or Cs_2_CO_3_ (3.0 equiv) were dissolved in ACN or DMF. The resulting suspension was stirred overnight at rt and solvent was evaporated under reduced pressure. The residue was partitioned between water and EtOAc, and the aqueous layer was extracted twice with EtOAc. The combined organic layers were dried with MgSO_4_, filtered and concentrated under reduced pressure. The resulting crude product was purified by column chromatography.

#### Procedure E: General Procedure for Mitsunobu Reaction

DIAD (1.2 equiv) was added slowly to a solution of *N*-Boc-serotonin (1.0 equiv), corresponding alcohols (1.2 equiv) and PPh_3_ (1.2 equiv). The mixture was stirred at rt for 48 h. The mixture was then diluted in water and extracted twice with DCM. The combined organic layers were dried with MgSO_4_, filtered and concentrated under reduced pressure. The resulting crude product was purified by column chromatography.

#### Procedure F: General Procedure for Boc Deprotection

HCl 4 M in dioxane or TFA was added to the *O*-alkylated-*N*-Boc-serotonin (1.0 equiv) in MeOH or DCM at 0 °C and stirred at rt overnight. The reaction mixture was concentrated under reduced pressure to afford the desired product as chlorhydrate or TFA salt.

#### Procedure G: General Procedure for Amide Synthesis

To a solution of corresponding malonic acid monoester (1.0 equiv) in DMF was added EDCI/HOBt or HBTU and Et_3_N. The resulting solution was stirred at rt fo 15 min. The amine derivative was added slowly and the mixture was stirred overnight at rt. The reaction mixture was then diluted with water and extracted twice with EtOAc. The combined organic layers were dried with MgSO_4_, filtered and concentrated under reduced pressure. The resulting crude product was purified by column chromatography.

##### 2-(Hydroxycarbamoyl)-4-methyl-*N*-[(3-phenylphenyl)methyl]pentanamide (**1**)

To a solution of **1′** (42.6 mg, 0.126 mmol, 1.0 equiv) in methanol (2 mL) was added aqueous hydroxylamine (50% in water, 2 mL) and KCN (1.634 mg, 0.025 mmol, 0.2 equiv). The mixture was stirred overnight at rt. The solvent was removed under reduced pressure and the crude was purified by flash chromatography on silica gel (DCM/MeOH, 100:0 to 90:10) to give the product as a yellowish powder (27 mg, 61%). Purity: 100%. LC tr = 1.12 min, MS (ESI+) *m*/*z*: 283 [M + H]^+^. HRMS *m*/*z*: calcd for C_20_H_25_N_2_O_3_ [M + H]^+^, 341.1865; found, 341.1863. ^1^H NMR (300 MHz, MeOD-*d*_4_): δ (ppm) 7.61–7.24 (m, 10H), 4.45 (dd, *J* = 15.0, 19.0 Hz, 2H), 3.16 (t, *J* = 7.7 Hz, 1H), 1.79–1.74 (m, 2H), 1.57 (sept, *J* = 6.6 Hz, 1H), 0.92 (d, *J* = 6.6 Hz, 6H). ^13^C NMR (75 MHz, MeOD-*d*_4_): δ (ppm) 172.0, 169.7, 142.8, 142.2, 140.4, 130.0, 129.8, 128.4, 127.9, 127.3, 127.0, 126.8, 51.0, 44.1, 40.8, 27.3, 22.7, 22.5.

##### 2-(Hydroxycarbamoyl)-4-methyl-*N*-[(4-phenylphenyl)methyl]pentanamide (**2**)

To a of compound **2′** (125 mg, 0.35 mmol, 1.0 equiv) in MeOH (1 mL) was added aqueous hydroxylamine (50% in water, 1 mL) followed by DBU (25 μL). The mixture was stirred overnight at rt, then solvents were evaporated in vacuo. The residue was washed with water and DCM, affording the desired product as a white solid (82 mg, 68%). Purity: 100%. LC tr = 2.53 min, MS (ESI+) *m*/*z*: 341 [M + H]^+^. HRMS *m*/*z*: calcd for C_20_H_24_N_2_O_3_ [M + H]^+^, 341.1865; found, 341.1852. ^1^H NMR (300 MHz, MeOD-*d*_4_): δ (ppm) 7.60–7.54 (m, 4H), 7.44–7.28 (m, 5H), 4.42 (s, 2H), 3.15 (t, *J* = 7.7 Hz, 2H), 1.85–1.69 (m, 2H), 1.57 (sept, *J* = 6.6 Hz, 1H), 0.94 (d, *J* = 6.6 Hz, 6H). ^13^C NMR (75 MHz, MeOD-*d*_4_): δ (ppm) 172.0, 169.8, 142.1, 141.5, 138.9, 129.9, 129.0, 128.3, 128.1, 127.9, 51.1, 43.9, 40.7, 27.3, 22.9, 22.5.

##### 4-Methyl-2-(1,3,4,5-tetrahydropyrido[4,3-*b*]indole-2-carbonyl)pentanehydroxamic Acid (**3**)

To a solution of **3′** (109 mg, 0.319 mmol, 1.0 equiv) in DMF (1 mL) was added aqueous hydroxylamine (50% in water,1 mL) followed by KCN (1 mg, 0.013 mmol, 0.4 equiv). The mixture was stirred overnight at rt, then the solvent was removed under reduced pressure. The residue was purified by reverse flash chromatography on C18 silica gel (H_2_O/MeOH, 90:10 to 0:100) to afford the desired product as a white solid (10 mg, 10%) and mixture of 4 rotamers. Purity: >95%. LC tr = 1.76 min, MS (ESI+) *m*/*z*: 330 [M + H]^+^. HRMS *m*/*z*: calcd for C_18_H_24_N_3_O_3_ [M + H]^+^, 330.1804; found, 330.1818. ^1^H NMR (500 MHz, MeOD-*d*_4_): δ (ppm) 7.40–7.19 (m, 2H), 7.13–6.89 (m, 2H), 4.85–4.60 (m, 2H), 3.98–3.89 (m, 1H + 0.4H), 3.76–3.61 (m, 0.8H), 3.39–3.33 (m, 0.3H), 3.05–2.67 (m, 2H + 0.2H), 2.13–1.51 (m, 3H + 0.4H), 0.98–0.85 (m, 6H)·^13^C NMR (125 MHz, MeOD-*d*_4_): δ (ppm) 171.9, 171.8, 171.7, 170.7, 170.3, 169.4, 169.2, 143.5, 138.1, 138.0, 131.5, 131.3, 130.8, 130.7, 130.6, 129.2, 129.1, 128.1, 125.4, 125.3, 124.6, 124.4, 123.4, 122.4, 122.2, 121.4, 120.0, 119.9, 118.8, 118.6, 118.5, 113.7, 112.4, 111.9, 111.4, 110.9, 110.9, 109.3, 108.1, 52.3, 52.2, 49.6, 49.5, 49.3, 47.9, 47.2, 45.5, 45.0, 42.5, 42.2, 40.2, 39.9, 39.0, 38.8, 37.8, 37.6, 31.4, 31.2, 27.5, 27.4, 27.3, 27.2, 23.1, 23.0, 22.8, 22.8, 22.7, 22.4, 22.4.

##### 2-(Hydroxycarbamoyl)-*N*-[2-(1*H*-imidazole-4-yl)ethyl]-4-methyl-pentanamide (**4**)

To a solution of compound **4′** (200 mg, 0.748 mmol, 1.0 equiv) in MeOH (2 mL) was added aqueous hydroxylamine (50% in water, 2 mL) followed by KCN (9.743 mg, 0.150 mmol, 0.2 equiv). The mixture was stirred overnight at rt and the solvent was then removed under vacuum. The resulting residue was purified by reverse phase chromatography on C18 silica gel (H_2_O/MeOH 90:10 to 0:100) to afford the desired product as a yellowish powder (26 mg, 13%). Purity: 100%. LC tr = 1.31 min, MS (ESI+) *m*/*z*: 269 [M + H]^+^. HRMS *m*/*z*: calcd for C_17_H_21_N_4_O_3_ [M + H]^+^, 269.1614; found, 269.1611. ^1^H NMR (300 MHz, MeOD-*d*_4_): δ (ppm) 7.67 (br d, *J* = 0.9 Hz, 1H), 6.87 (br s, 1H), 3.44 (t, *J* = 6.9 Hz, 2H), 3.04 (t, *J* = 7.7 Hz, 1H), 2.78 (t, *J* = 6.9 Hz, 2H), 1.75–1.59 (m, 2H), 1.48 (sept, *J* = 6.6 Hz, 1H), 0.90 (d, *J* = 6.6 Hz, 6H). ^13^C NMR (75 MHz, MeOD-*d*_4_): δ (ppm) 172.0, 169.8, 136.1, 135.7, 118.0, 51.0, 40.7, 40.4, 27.5, 27.3, 22.8, 22.6.

##### 2-(Hydroxycarbamoyl)-*N*-(3-imidazole-1-ylpropyl)-4-methyl-pentanamide (**5**)

To a solution of **5′** (209 mg, 0.596 mmol, 1.0 equiv) in MeOH (2 mL) was added aqueous hydroxylamine (50% in water, 2 mL) and KCN (7.758 mg, 0.119 mmol, 0.2 equiv). The mixture was stirred overnight at rt and solvents were then evaporated under vacuum. The residue was purified by flash chromatography on silica gel (DCM/MeOH, 100:0 to 90:10) to give the product as a white powder. Purity: 100%. LC tr = 1.32 min, MS (ESI+) = 283 [M + H]^+^. HRMS *m*/*z*: calcd for C_13_H_23_N_4_O_3_ [M + H]^+^, 283.1770; found, 283.1772. ^1^H NMR (300 MHz, MeOD-*d*_4_): δ (ppm) 7.87 (s, 1H), 7.23 (s, 1H), 7.06 (s, 1H), 4.07 (t, *J* = 6.8 Hz, 2H), 3.20 (t, *J* = 6.5 Hz, 2H), 3.09 (t, *J* = 7.8 Hz, 1H), 2.04–1.95 (m, 2H), 1.76–1.67 (m, 2H), 1.56–1.52 (m, 1H), 0.94 (d, *J* = 6.6 Hz, 6H). ^13^C NMR (75 MHz, MeOD-*d*_4_): δ (ppm) 172.2, 169.8, 138.2, 127.8, 121.1, 51.1, 45.8, 40.3, 37.4, 31.7, 27.4, 22.8, 22.5.

##### 2-(Hydroxycarbamoyl)-*N*-[2-(1*H*-indol-3-yl)ethyl]-4-methyl-pentanamide (**6**)

To a solution of **6′** (175 mg, 0.55 mmol, 1.0 equiv) in MeOH (2 mL) was added aqueous hydroxylamine (50% in water, 1.0 mL), followed by KCN (7.2 mg, 0.11 mmol, 0.2 equiv). The resulting solution was stirred for 16 h at rt and solvents were then evaporated in vacuo. The residue was purified by flash chromatography on silica gel (DCM/MeOH 99:1 to 95:5). The product was then washed with MeCN, affording the desired compound as a white solid (136 mg, 76%). Purity: 100%. LC tr = 2.12 min, MS (ESI+) *m*/*z*: 318 [M + H]^+^. HRMS *m*/*z*: calcd for C_17_H_24_N_3_O_3_ [M + H]^+^, 318.1818; found, 318.1803. ^1^H NMR (300 MHz, MeOD-*d*_4_): δ (ppm) 7.56 (br d, *J* = 8.0 Hz, 1H), 7.33 (br d, *J* = 8.0 Hz, 1H), 7.10–6.97 (m, 3H), 3.50 (t, *J* = 7.1 Hz, 2H), 3.02 (t, *J* = 7.7 Hz, 1H), 2.94 (t, *J* = 7.1 Hz, 2H), 1.73–1.55 (m, 2H), 1.46 (sept, *J* = 6.6 Hz, 1H), 0.87 (d, *J* = 6.6 Hz, 6H). ^13^C NMR (75 MHz, MeOD-*d*_4_): δ (ppm) 168.9, 166.9, 136.3, 127.2, 122.7, 122.0, 118.3, 118.2, 111.6, 111.4, 49.0, 39.6, 38.7, 25.6, 25.1, 22.5, 22.1.

##### 2-(Hydroxycarbamoyl)-*N*-[2-(1*H*-indol-3-yl)ethyl]-5-methyl-hexanamide (**7**)

To a solution of **7′** (155 mg, 0.469 mmol, 1.0 equiv) in MeOH (2 mL) was added aqueous hydroxylamine (50% in water, 2 mL) and KCN (6.1 mg, 0.094 mmol, 0.2 equiv). The mixture was stirred overnight at rt, and then filtered under vacuum. The resulting residue was dissolved in MeOH, and after the removal of MeOH under vacuum, the product was obtained as a white powder (109 mg, 69%). Purity: 100%. LC tr = 2.38 min, MS (ESI+) *m*/*z*: 332 [M + H]^+^. HRMS *m*/*z*: calcd for C_18_H_26_N_3_O_3_ [M + H]^+^, 332.1974; found, 332.1961. ^1^H NMR (300 MHz, MeOD-*d*_4_): δ (ppm) 7.57–7.54 (m, 1H), 7.34–7.30 (td, *J* = 8.0, 1.0 Hz, 1H), 7.10–6.97 (m, 3H), 3.50 (td, *J* = 7.1, 1.9 Hz, 2H), 2.94 (t, *J* = 7.0 Hz, 2H), 2.85 (t, *J* = 7.6 Hz, 1H), 1.80–1.72 (m, 2H), 1.50 (sept, *J* = 6.6 Hz, 1H), 1.16–1.08 (m, 2H), 0.86 (d, *J* = 6.6 Hz, 6H). ^13^C NMR (75 MHz, MeOD-*d*_4_): δ (ppm) 171.9, 169.9, 138.2, 128.6, 123.5, 122.3, 119.6, 119.2, 112.9, 112.2, 52.8, 41.3, 37.5, 30.0, 28.9, 26.1, 22.8, 22.7.

##### 2-(Hydroxycarbamoyl)-*N*-[2-(1*H*-indol-3-yl)ethyl]-5,5-dimethyl-hexanamide (**8**)

To a solution of **8′** (281.2 mg, 0.816 mmol, 1.0 equiv) in MeOH (2 mL) was added aqueous hydroxylamine (50% in water, 2 mL) and KCN (10.6 mg, 0.163 mmol, 0.2 equiv). The mixture was stirred overnight at rt and then filtered under vacuum. The resulting residue was dissolved in MeOH, and after the removal of MeOH under vacuum, the product was obtained as a white powder (169 mg, 57%). Purity: 100%. LC tr = 2.48 min, MS (ESI+) *m*/*z*: 346 [M + H]^+^. HRMS *m*/*z*: calcd for C_19_H_28_N_3_O_3_ [M + H]^+^, 346.2131; found, 346.2119. ^1^H NMR (300 MHz, MeOD-*d*_4_): δ (ppm) 7.56 (d, *J* = 7.6 Hz, 1H), 7.32 (d, *J* = 8.0 Hz, 1H), 7.10–6.97 (m, 3H), 3.50 (td, *J* = 7.1, 2.7 Hz, 2H), 2.94 (t, *J* = 7.1 Hz, 1H), 2.81 (t, *J* = 7.5 Hz, 1H), 1.80–1.70 (m, 2H), 1.16–1.09 (m, 2H), 0.85 (s, 9H). ^13^C NMR (75 MHz, MeOD-*d*_4_): δ (ppm) 26.1, 27.4, 29.6, 30.9, 41.3, 42.5, 53.2, 112.2, 112.9, 119.2, 119.6, 122.3, 123.6, 128.7, 138.2, 169.9, 171.9.

##### 2-(Hydroxycarbamoyl)-*N*-[2-(1*H*-indol-3-yl)ethyl]-4-phenyl-butanamide (**9**)

To a solution of **9′** (215 mg, 0.59 mmol, 1.0 equiv) in MeOH (2 mL) was added aqueous hydroxylamine (50% in water, 1.6 mL), followed by KCN (7.8 mg, 0.12 mmol, 0.2 equiv). The resulting solution was stirred for 16 h at rt, and solvents were then evaporated in vacuo. The residue was purified by flash chromatography on silica gel (DCM/MeOH 99:1 to 95:5) to afford the desired product as a white solid (114 mg, 52%). Purity: 100%. LC tr = 2.30 min, MS (ESI+) *m*/*z*: 366 [M + H]^+^. HRMS *m*/*z*: calcd for C_21_H_24_N_3_O_3_ [M + H]^+^, 366.1818; found, 366.1805. ^1^H NMR (300 MHz, MeOD-*d*_4_): δ (ppm) 7.56 (ddd, *J* = 7.9, 1.0, 0.8 Hz, 1H), 7.31 (ddd, *J* = 8.2, 1.0, 0.8 Hz, 1H), 7.26–7.20 (m, 2H), 7.18–7.05 (m, 5H), 7.01–6.96 (m, 1H), 3.51 (t, *J* = 7.2 Hz, 2H), 2.94 (td, *J* = 7.2, 5.8 Hz, 3H), 2.59–2.44 (m, 2H), 2.10–2.01 (m, 2H). ^13^C NMR (300 MHz, MeOD-*d*_4_): δ (ppm) 171.5, 169.5, 142.2, 138.2, 129.5, 128.7, 127.1, 123.6, 122.3, 119.6, 119.3, 112.9, 112.3, 52.1, 41.4, 34.4, 33.5, 26.1.

##### *N*-[2-(5-Fluoro-1*H*-indol-3-yl)ethyl]-2-(hydroxycarbamoyl)-4-methyl-pentanamide (**10**)

To a solution of **10′** (111 mg, 0.319 mmol, 1.0 equiv) in DMF (1 mL) was added aqueous hydroxylamine (50% in water, 1 mL) and KCN (1 mg, 0.013 mmol, 0.4 equiv). The mixture was stirred overnight at rt, and the solvent was removed under reduced pressure. The residue was purified by reverse flash chromatography on C18 silica gel (H_2_O/MeOH, 90:10 to 0:100) to afford the desired product as a white solid (37 mg, 34%). Purity: >95%. LC tr = 2.04 min, MS (ESI+) *m*/*z*: 750 [M + H]^+^. HRMS *m*/*z*: calcd for C_17_H_23_FN_3_O_3_ [M + H]^+^, 336.1723; found, 336.1718. ^1^H NMR (300 MHz, DMSO-*d*_6_): δ (ppm) 10.93 (s, 1H), 10.49 (br s, 1H), 8.94 (br s, 1H), 7.70 (t, *J* = 5.5 Hz, 1H), 7.36–7.27 (m, 2H), 7.21 (br d, *J* = 2.1 Hz, 1H), 6.90 (td, *J* = 9.2, 2.4 Hz, 1H), 3.30 (q, *J* = 7.3 Hz, 2H), 2.98–2.93 (m, 1H), 2.76 (t, *J* = 7.3 Hz, 2H), 1.66–1.44 (m, 2H), 1.43–1.31 (m, 1H), 0.82 (d, *J* = 6.5 Hz, 6H). ^13^C NMR (75 MHz, DMSO-*d*_6_): δ (ppm) 169.0, 166.9, 156.6 (d, *J* = 230.0 Hz), 132.9, 127.5 (d, *J* = 9.4 Hz), 124.9, 112.3 (d, *J* = 9.5 Hz), 112.0 (d, *J* = 4.8 Hz), 109.0 (d, *J* = 26.2 Hz), 103.0 (d, *J* = 22.7 Hz), 49.0, 39.5, 38.7, 25.5, 25.0, 22.5, 22.1.

##### *N*-[2-(6-Fluoro-1*H*-indol-3-yl)ethyl]-2-(hydroxycarbamoyl)-4-methyl-pentanamide (**11**)

To a solution of **11′** (111 mg, 0.319 mmol, 1.0 equiv) in DMF (1 mL) was added aqueous hydroxylamine (50% in water,1 mL) and KCN (1 mg, 0.013 mmol, 0.4 equiv). The mixture was stirred overnight at rt, and the solvent was removed under reduced pressure. The residue was purified by reverse flash chromatography on C18 silica gel (H_2_O/MeOH, 90:10 to 0:100) to afford the desired product as a white solid (42 mg, 39%). Purity: >95%. LC tr = 1.68 min, MS (ESI+) *m*/*z*: 336 [M + H]^+^. HRMS *m*/*z*: calcd for C_17_H_23_FN_3_O_3_ [M + H]^+^, 336.1723; found, 336.1721. ^1^H NMR (300 MHz, DMSO-*d*_6_): δ (ppm) 10.90 (s, 1H), 7.72 (br t, *J* = 5.5 Hz, 1H), 7.52 (dd, *J* = 8.5, 5.5 Hz, 1H), 7.13 (s, 1H), 7.10 (dd, *J* = 10.4, 2.5 Hz, 1H), 6.87–6.80 (m, 1H), 3.41–3.26 (m, 3H), 2.96 (t, *J* = 7.5 Hz, 1H), 2.78 (t, *J* = 7.5 Hz, 2H), 1.65–1.46 (m, 2H), 1.44–1.32 (m, 1H), 0.83 (d, *J* = 6.4 Hz, 6H). ^13^C NMR (75 MHz, DMSO-*d*_6_): δ (ppm) 169.0, 168.9, 158.8 (d, *J* = 233.4 Hz), 136.0 (d, *J* = 12.8 Hz), 124.1, 123.3 (d, *J* = 3.2 Hz), 119.3 (d, *J* = 10.2 Hz), 111.9, 106.7 (d, *J* = 24.7 Hz), 97.3 (d, *J* = 25.4 Hz), 49.0, 39.4, 38.7, 25.5, 25.0, 22.5, 22.1.

##### *N*-[2-(5-Chloro-1*H*-indol-3-yl)ethyl]-2-(hydroxycarbamoyl)-4-methyl-pentanamide (**12**)

To a solution of **12′** (209 mg, 0.596 mmol, 1.0 equiv) in MeOH (2 mL) was added aqueous hydroxylamine (50% in water, 2 mL) and KCN (7.758 mg, 0.119 mmol, 0.2 equiv). The mixture was stirred overnight at rt, and then filtered under vacuum. The resulting residue was dissolved in MeOH, and after the removal of MeOH under vacuum, the product was obtained as a white powder (122 mg, 58%). Purity: 100%. LC tr = 2.42 min, MS (ESI+) *m*/*z*: 352 [M + H]^+^. HRMS *m*/*z*: calcd for C_17_H_23_N_3_O_3_Cl [M + H]^+^, 352.1428; found, 352.1408. ^1^H NMR (300 MHz, MeOD-*d*_4_): δ (ppm) 7.55 (d, *J* = 1.9 Hz, 1H), 7.29 (d, *J* = 8.6 Hz, 1H), 7.13 (s, 1H), 7.04 (dd, *J* = 8.6, 1.9 Hz, 1H), 3.47 (t, *J* = 7.2 Hz, 2H), 3.02 (dd, *J* = 8.2, 8.1 Hz, 1H), 2.90 (t, *J* = 7.0 Hz, 2H), 1.68–1.56 (m, 2H), 1.47–1.42 (m, 1H), 0.87 (d, *J* = 6.4 Hz, 6H). ^13^C NMR (75 MHz, MeOD-*d*_4_): δ (ppm) 172.0, 169.9, 136.5, 129.9, 125.4, 122.4, 118.7, 113.4, 112.9, 51.0, 41.3, 40.8, 27.2, 25.8, 22.8, 22.5.

##### *N*-[2-(5-Bromo-1*H*-indol-3-yl)ethyl]-2-(hydroxycarbamoyl)-4-methyl-pentanamide (**13**)

To a solution of **13′** (339.5 mg, 0.829 mmol, 1.0 equiv) in MeOH (2 mL) was added aqueous hydroxylamine (50% in water, 2 mL) and KCN (10.8 mg, 0.166 mmol, 0.2 equiv). The mixture was stirred overnight at rt, and then filtered under vacuum. The resulting residue was dissolved in MeOH, and after the removal of MeOH under vacuum, the residue was purified by flash chromatography on silica gel (DCM/MeOH, 100:0 to 90:10). The desired product was obtained as a white powder (209 mg, 63%). Purity: 100%. LC tr = 2.45 min, MS (ESI+) *m*/*z*: 398 [M + H]^+^. HRMS *m*/*z*: calcd for C_17_H_23_N_3_O_3_Br [M + H]^+^, 396.0923; found, 96.0931. ^1^H NMR (300 MHz, MeOD-*d*_4_): δ (ppm) 7.69 (d, *J* = 1.4 Hz, 1H), 7.25 (dd, *J* = 0.4, 8.6 Hz, 1H), 7.16 (dd, *J* = 1.8, 8.6 Hz, 1H), 7.11 (s, 1H), 3.47 (t, *J* = 7.1 Hz, 2H), 3.03 (dd, *J* = 7.2, 8.2 Hz, 1H), 2.89 (t, *J* = 7.0 Hz, 2H), 1.72–1.54 (m, 2H), 1.49–1.39 (m, 1H), 0.87 (d, *J* = 6.5 Hz, 6H). ^13^C NMR (75 MHz, MeOD-*d*_4_): δ (ppm) 172.0, 169.9, 136.7, 130.5, 125.3, 125.0, 121.9, 113.9, 112.8, 112.7 51.0, 41.3, 40.8, 27.2, 25.8, 22.8, 22.5.

##### 2-(Hydroxycarbamoyl)-4-methyl-*N*-[2-(5-methyl-1*H*-indol-3-yl)ethyl]pentanamide (**14**)

To a solution of **14′** (110 mg, 0.319 mmol, 1.0 equiv) in DMF (1 mL) was added aqueous hydroxylamine (50% in water, 1 mL) and KCN (1 mg, 0.013 mmol, 0.4 equiv). The mixture was stirred overnight at rt, and the solvent was removed under reduced pressure. The residue was then purified by reverse flash chromatography on C18 silica gel (H_2_O/MeOH, 90:10 to 0:100) to afford the desired product as a white solid (30 mg, 28%). Purity: >95%. LC tr = 1.74 min, MS (ESI+) *m*/*z*: 332 [M + H]^+^. HRMS *m*/*z*: calcd for C_18_H_26_N_3_O_3_ [M + H]^+^, 332.1974; found, 332.1966. ^1^H NMR (300 MHz, MeOD-*d*_4_): δ (ppm) 10.67 (s, 1H), 10.47 (br s, 1H), 8.94 (br s, 1H), 7.70 (br t, *J* = 5.6 Hz, 1H), 7.32 (s, 1H), 7.21 (d, *J* = 8.1 Hz, 1H), 7.07 (d, *J* = 2.2 Hz, 1H), 6.88 (dd, *J* = 8.2,1.1 Hz, 1H), 4.31 (q, *J* = 6.7 Hz, 2H), 2.97 (t, *J* = 7.7 Hz, 1H), 2.77 (t, *J* = 7.7 Hz, 2H), 2.37 (s, 3H), 1.66–1.49 (m, 2H), 1.47–1.34 (m, 1H), 0.84 (d, *J* = 6.6 Hz, 6H). ^13^C NMR (75 MHz, MeOD-*d*_4_): δ (ppm) 168.9, 166.9, 134.6, 127.4, 126.6, 122.8, 122.5, 117.9, 111.1, 49.0, 39.7, 38.7, 25.6, 25.1, 22.6, 22.1, 21.3.

##### 2-(Hydroxycarbamoyl)-*N*-[2-(5-hydroxy-1*H*-indol-3-yl)ethyl]-4-methyl-pentanamide (**15**)

Toa solution of **15′** (111 mg, 0.319 mmol, 1.0 equiv) in DMF (1 mL) was added aqueous hydroxylamine (50% in water, 1 mL) and KCN (1 mg, 0.13 mmol, 0.4 equiv). The mixture was stirred 2 days at rt and the solvent was removed under reduced pressure. The residue was purified by reverse flash chromatography on C18 silica gel (H_2_O/MeOH, 90:10 to 0:100) to afford the desired product as a white solid (37 mg, 34%). Purity: >95%. LC tr = 1.38 min, MS (ESI+) *m*/*z*: 334 [M + H]^+^. HRMS *m*/*z*: calcd for C_17_H_24_N_3_O_4_ [M + H]^+^, 334.1767; found, 334.1758.^1^H NMR (300 MHz, DMSO-*d*_6_): δ (ppm) 10.49 (s, 1H), 8.10 (t, *J* = 5.5 Hz, 1H), 7.11 (d, *J* = 8.6 Hz, 1H), 7.01 (s, 1H), 6.83 (d, *J* = 2.1 Hz, 1H), 6.58 (dd, *J* = 8.3, 1.5 Hz, 1H), 3.31–3.22 (m, 2H), 2.87–2.79 (m, 1H), 2.69 (t, *J* = 7.5 Hz, 2H), 1.64–1.53 (m, 1H), 1.52–1.39 (m, 2H), 0.87–0.79 (m, 6H). ^13^C NMR (75 MHz, DMSO-*d*_6_): δ (ppm) 170.7, 166.4, 156.3, 130.8, 127.9, 123.1, 111.6, 111.3, 110.8, 49.0, 39.44, 39.38, 25.5, 25.4, 22.8, 22.1.

##### 2-(Hydroxycarbamoyl)-*N*-[2-(5-methoxy-1*H*-indol-3-yl)ethyl]-4-methyl-pentanamide (**16**)

To a solution of **16′** (111 mg, 0.319 mmol, 1.0 equiv) in DMF (1 mL) was added aqueous hydroxylamine (50% in water, 1 mL) and KCN (1 mg, 0.13 mmol, 0.4 equiv). The mixture was stirred 2 days at rt. The solvent was removed under reduced pressure and the residue was purified by reverse flash chromatography on C18 silica gel (H_2_O/MeOH, 90:10 to 0:100) to afford the desired product as a white solid (58 mg, 52%). Purity: >95%. LC tr = 1.59 min, MS (ESI+) *m*/*z*: 348 [M + H]^+^. HRMS *m*/*z*: calcd for C_18_H_26_N_3_O_4_ [M + H]^+^, 348.1923; found, 348.1920. ^1^H NMR (300 MHz, DMSO-*d*_6_): δ (ppm) 10.67 (s, 1H), 8.07 (t, *J* = 5.3 Hz, 1H), 7.22 (d, *J* = 9.0 Hz, 1H), 7.08 (s, 1H), 7.03 (d, *J* = 2.4 Hz, 1H), 6.70 (dd, *J* = 8.6, 2.4 Hz, 1H), 3.76 (s, 3H), 3.31 (q, *J* = 6.4 Hz, 2H), 2.89–2.84 (m, 1H), 2.75 (t, *J* = 7.5 Hz, 2H), 1.66–1.54 (m, 1H), 1.51–1.36 (m, 2H), 0.86–0.79 (m, 6H). ^13^C NMR (75 MHz, DMSO-*d*_6_): δ (ppm) 170.5, 166.5, 153.0, 131.4, 127.5, 123.4, 112.0, 111.5, 111.1, 100.1, 55.4, 49.0, 39.35, 39.29, 25.5, 25.2, 22.8, 22.1.

##### 2-(Hydroxycarbamoyl)-*N*-[2-(6-methoxy-1*H*-indol-3-yl)ethyl]-4-methyl-pentanamide (**17**)

To a solution of **17′** (115 mg, 0.319 mmol, 1.0 equiv) in DMF (1 mL) was added aqueous hydroxylamine (50% in water,1 mL) followed by KCN (8 mg, 0.13 mmol, 0.4 equiv). The mixture was stirred for 2 days at rt and the solvent was removed under reduced pressure. The residue was purified by reverse flash chromatography on C18 silica gel (H_2_O/MeOH, 90:10 to 0:100) to afford the desired product as a white solid (52 mg, 46%). Purity: >95%. LC tr = 1.61 min, MS (ESI+) *m*/*z*: 348 [M + H]^+^. HRMS *m*/*z*: calcd for C_18_H_26_N_3_O_4_ [M + H]^+^, 348.1923; found, 348.1910. ^1^H NMR (300 MHz, DMSO-*d*_6_): δ (ppm) 10.64 (s, 1H), 8.13 (t, *J* = 6.1 Hz, 1H), 7.40 (d, *J* = 8.6 Hz, 1H), 6.98 (s, 1H), 6.83 (d, *J* = 2.2 Hz, 1H), 6.62 (dd, *J* = 8.6, 2.2 Hz, 1H), 3.74 (s, 3H), 3.29 (q, *J* = 6.6 Hz, 2H), 2.89–2.81 (m, 1H), 2.73 (t, *J* = 7.8 Hz, 2H), 1.66–1.53 (m, 1H), 1.51–1.36 (m, 2H), 0.83 (d, *J* = 1.8 Hz, 3H), 0.81 (d, *J* = 1.8 Hz, 3H). ^13^C NMR (75 MHz, DMSO-*d*_6_): δ (ppm) 170.6, 166.4, 155.5, 136.9, 121.6, 121.2, 118.9, 111.7, 108.4, 95.5, 55.1, 48.9, 39.6, 39.4, 25.5, 25.3, 22.8, 22.1.

##### *N*-[2-(5-Benzyloxy-1*H*-indol-3-yl)ethyl]-2-(hydroxycarbamoyl)-4-methyl-pentanamide (**18**)

To a solution of **18′** (169 mg, 0.387 mmol, 1.0 equiv) in MeOH (2 mL) was added aqueous hydroxylamine (50% in water, 2 mL), followed by KCN (5.042 mg, 0.077 mmol, 0.2 equiv). The mixture was stirred overnight at rt and then filtered under vacuum. The resulting residue was dissolved in MeOH, and after the removal of MeOH under vacuum, the product was obtained as a white powder (127 mg, 75%). Purity: 98.4%. LC tr = 2.63 min, MS (ESI+) *m*/*z*: 424 [M + H]^+^. HRMS *m*/*z*: calcd for C_24_H_30_N_3_O_4_ [M + H]^+^, 424.2243; found, 424.2236. ^1^H NMR (300 MHz, DMSO-*d*_6_): δ (ppm) 10.7 (br s, 1H), 10.5 (br s, 1H), 8.93 (br s, 1H), 7.69 (t, *J* = 5.7 Hz, 1H), 7.09–7.50 (m, 9H), 6.79 (dd, *J* = 2.4, 8.7 Hz, 1H), 5.09 (s, 2H), 3.32 (q, *J* = 7.2 Hz, 2H), 2.98 (dd, *J* = 7.0, 8.3 Hz, 1H), 2.76 (t, *J* = 7.2 Hz, 2H), 1.66–1.34 (m, 3H), 0.84 (d, *J* = 6.5 Hz, 6H). ^13^C NMR (75 MHz, DMSO-*d*_6_): δ (ppm) 168.9, 166.9, 152.0, 137.8, 131.6, 128.3, 127.7, 127.6, 127.5, 123.5, 112.0, 111.6, 111.4, 101.8, 69.8, 49.1, 39.6, 39.0, 25.6, 25.1, 22.5, 22.1.

##### *N*-[2-(6-Benzyloxy-1*H*-indol-3-yl)ethyl]-2-(hydroxycarbamoyl)-4-methyl-pentanamide (**19**)

To a solution of **19′** (155 mg, 0.36 mmol, 1.0 equiv) in MeOH (2 mL) was added aqueous hydroxylamine (50% in water, 2 mL), followed by KCN (5 mg, 0.07 mmol, 0.2 equiv). The mixture was stirred overnight at rt, then solvents were evaporated in vacuo. The residue was purified by flash chromatography on silica gel (DCM/MeOH, 100:0 to 90:10), affording the desired product as an off-white solid (37 mg, 27%). Purity: >95%. LC tr = 2.52 min, MS (ESI+) *m*/*z*: 424 [M + H]^+^. HRMS *m*/*z*: calcd for C_24_H_30_N_3_O_4_ [M + H]^+^, 424.2236; found, 424.2227. ^1^H NMR (300 MHz, DMSO-*d*_6_): δ (ppm) 10.61 (br s, 1H), 10.47 (br s, 1H), 8.92 (br s, 1H), 7.68 (br t, *J* = 5.8 Hz, 1H), 7.48–7.36 (m, 5H), 7.34–7.28 (m, 1H), 6.98 (d, *J* = 2.0 Hz, 1H), 6.90 (d, *J* = 2.0 Hz, 1H), 6.72 (dd, *J* = 8.6, 2.3 Hz, 1H), 5.10 (s, 2H), 4.04 (s, 1H), 2.96 (dd, *J* = 8.1, 7.2 Hz, 1H), 2.74 (t, *J* = 7.4 Hz, 2H), 1.64–1.35 (m, 4H), 0.83 (d, *J* = 6.5 Hz, 6H). ^13^C NMR (75 MHz, DMSO-*d*_6_): δ (ppm) 168.9, 166.9, 154.5, 137.7, 136.8, 128.4, 127.6, 127.5, 121.8, 121.4, 118.9, 111.6, 109.1, 96.0, 69.5, 49.0, 39.6, 38.6, 25.6, 25.2, 22.5, 22.1.

##### 2-(Hydroxycarbamoyl)-4-methyl∼{*N*}-[2-[5-(2-pyridylmethoxy)-1∼{*H*}-indol-3-yl]ethyl]pentan-amide (**20**)

To a solution of **20′** (85 mg, 0.194 mmol, 1.0 equiv) in MeOH (1 mL), was added aqueous hydroxylamine (50% in water, 1 mL), followed by DBU (25 μL). The mixture was stirred overnight at rt, and solvents were then evaporated under vacuum. The crude product was purified by flash chromatography on silica gel (DCM/MeOH, 100:0 to 90:10), affording the desired product as an off white solid (49 mg, 59%). Purity: >95%. LC tr = 2.12 min, MS (ESI+) *m*/*z*: 425 [M + H]^+^. HRMS *m*/*z*: calcd for C_23_H_29_N_4_O_4_ [M + H]^+^, 425.2189; found, 425.2191. ^1^H NMR (300 MHz, DMSO-*d*_6_): δ (ppm) 10.69 (br d, *J* = 2.0 Hz, 1H), 10.50 (br s, 1H), 8.9 (br s, 1H), 8.58 (ddd, *J* = 4.8, 1.6, 0.8 Hz, 1H), 7.83 (dt, *J* = 7.7, 1.9 Hz, 1H), 7.69 (br t, *J* = 5.7 Hz, 1H), 7.57 (br d, *J* = 7.8 Hz, 1H), 7.33 (ddd, *J* = 7.5, 4.8, 1.0 Hz, 1H), 7.24 (d, *J* = 8.7 Hz, 1H), 7.16 (d, *J* = 2.3 Hz, 1H), 7.10 (d, *J* = 2.3 Hz, 1H), 6.82 (dd, *J* = 8.7, 2.3 Hz, 1H), 5.17 (s, 2H), 3.31 (br q, *J* = 7.1 Hz, 2H), 2.97 (dd, *J* = 8.3, 7.1 Hz, 1H), 2.75 (br t, *J* = 7.4 Hz, 2H), 1.65–1.47 (m, 2H), 1.40 (sept, *J* = 6.5 Hz, 1H), 0.83 (d, *J* = 6.5 Hz, 6H). ^13^C NMR (75 MHz, DMSO-*d*_6_): δ (ppm) 169.9, 168.9, 157.5, 151.7, 149.0, 136.9, 131.7, 127.5, 123.6, 122.8, 121.6, 112.0, 111.4, 101.9, 70.9, 49.0, 39.4, 38.6, 25.5, 25.1, 22.5, 22.1.

##### 2-(Hydroxycarbamoyl)-4-methyl-*N*-[2-[5-(3-pyridylmethoxy)-1*H*-indol-3-yl]ethyl]pentanamide (**21**)

To a solution of **21′** (85 mg, 0.194 mmol, 1.0 equiv) in MeOH (1 mL), was added aqueous hydroxylamine (50% in water, 1 mL), followed by DBU (25 μL). The mixture was stirred overnight at rt, and solvents were then evaporated under vacuum. The crude product was purified by flash chromatography on silica gel (DCM/MeOH, 100:0 to 90:10), affording the product as an off white solid (56 mg, 68% yield). Purity: >95%. LC tr = 2.01 min, MS (ESI+) *m*/*z*: 425 [M + H]^+^. HRMS *m*/*z*: calcd for C_23_H_29_N_4_O_4_ [M + H]^+^, 425.2189; found, 425.2171. ^1^H NMR (300 MHz, DMSO-*d*_6_): δ (ppm) 10.69 (br d, *J* = 2.0 Hz, 1H), 10.51 (br s, 1H), 8.95 (br s, 1H), 8.70 (d, *J* = 1.6 Hz, 1H), 8.54 (dd, *J* = 4.7, 1.6 Hz, 1H), 7.91 (dt, *J* = 7.8, 1.9 Hz, 1H), 7.71 (br t, *J* = 5.7 Hz, 1H), 7.43 (ddd, *J* = 7.8, 4.7, 0.7 Hz, 1H), 7.24 (d, *J* = 8.8 Hz, 1H), 7.19 (d, *J* = 2.4 Hz, 1H), 7.11 (d, *J* = 1.9 Hz, 1H), 6.80 (dd, *J* = 8.8, 2.4 Hz, 1H), 5.15 (s, 2H), 3.32 (br q, *J* = 6.9 Hz, 2H), 2.98 (dd, *J* = 8.2, 6.9 Hz, 1H), 2.77 (br t, *J* = 7.4 Hz, 2H), 1.66–1.47 (m, 2H), 1.41 (sept, *J* = 6.5 Hz, 1H), 0.83 (d, *J* = 6.5 Hz, 6H). ^13^C NMR (75 MHz, DMSO-*d*_6_): δ (ppm) 168.9, 167.0, 151.7, 148.9, 135.7, 133.4, 131.7, 127.5, 123.6, 112.0, 111.6, 111.4, 102.0, 102.0, 49.0, 39.5, 38.7, 25.6, 25.1, 22.5, 22.1.

##### 2-(Hydroxycarbamoyl)-4-methyl-*N*-[2-[5-(4-pyridylmethoxy)-1*H*-indol-3-yl]ethyl]pentanamide (**22**)

To a solution of **22′** (75.0 mg, 0.171 mmol, 1.0 equiv) in MeOH (2 mL) was added aqueous hydroxylamine (50% in water, 2 mL) followed by DBU (25 μL). The solution was stirred overnight at rt, and solvents were then evaporated under vacuum. The residue was purified by flash chromatography on silica gel (DCM/MeOH, 100:0 to 90:10), affording the desired product as a white powder (26 mg, 36%). Purity: >95%. LC tr = 1.95 min, MS (ESI+) *m*/*z*: 425 [M + H]^+^. HRMS *m*/*z*: calcd for C_23_H_29_N_4_O_4_ [M + H]^+^, 425.2189; found, 425.2167. ^1^H NMR (300 MHz, MeOD-*d*_4_): δ (ppm) 8.51 (br s, 2H), 7.55 (br d, *J* = 4.2 Hz, 2H), 7.25 (d, *J* = 8.7 Hz, 1H), 7.15 (d, *J* = 2.1 Hz, 1H), 7.05 (s, 1H), 6.86 (dd, *J* = 8.6, 2.2 Hz, 1H), 5.19 (s, 2H), 3.46 (t, *J* = 7.1 Hz, 2H), 3.03 (t, *J* = 7.5 Hz, 1H), 2.89 (t, *J* = 7.1 Hz, 2H), 1.72–1.53 (m, 2H), 1.45 (sept, *J* = 6.5 Hz, 1H), 0.86 (d, *J* = 6.5 Hz, 6H). ^13^C NMR (75 MHz, MeOD-*d*_4_): δ (ppm) 172.0, 169.9, 153.5, 150.5, 150.0, 133.8, 129.0, 124.7, 123.5, 113.1, 112.8, 103.1, 69.9, 51.0, 41.3, 40.9, 27.2, 26.1, 22.8, 22.5.

##### *N*-[2-(5-Benzyloxy-1*H*-indol-3-yl)ethyl]-2-(hydroxycarbamoyl)-5-methyl-hexanamide (**23**)

To a solution of **23′** (175 mg, 0.40 mmol, 1.0 equiv) in MeOH (2 mL) was added aqueous hydroxylamine (50% in water, 2 mL), followed by KCN (5 mg, 0.08m mol, 0.2 equiv). The solution was stirred overnight at rt, and solvents were then evaporated under vacuum. The residue was purified by flash chromatography on silica gel (DCM/MeOH, 100:0 to 90:10), affording the desired product as a white solid (121 mg, 69%). Purity: >95%. LC tr = 2.62 min, MS (ESI+) *m*/*z*: 438 [M + H]^+^. HRMS *m*/*z*: calcd for C_25_H_32_N_3_O_4_ [M + H]^+^, 438.2393; found, 438.2393. ^1^H NMR (300 MHz, MeOD-*d*_4_): δ (ppm) 7.48–7.44 (m, 2H), 7.38–7.25 (m, 3H), 7.23 (d, *J* = 8.7 Hz, 1H), 7.16 (d, *J* = 2.3 Hz, 1H), 7.04 (s, 1H), 6.83 (dd, *J* = 8.7, 2.3 Hz, 1H), 5.08 (s, 2H), 3.47 (td, *J* = 7.1, 2.2 Hz, 2H), 2.88 (q, *J* = 7.1 Hz, 2 + 1H), 1.82–1.70 (m, 2H), 1.49 (sept, *J* = 6.6 Hz, 1H), 1.16–1.08 (m, 2H), 0.85 (d, *J* = 6.6 Hz, 6H). ^13^C NMR (75 MHz, MeOD-*d*_4_): δ (ppm) 171.9, 169.9, 154.0, 139.4, 133.6, 129.4, 129.0, 128.8, 128.7, 124.5, 113.3, 112.9, 112.7, 103.2, 72.1, 52.8, 41.2, 37.5, 30.0, 28.9, 26.1, 22.8, 22.8.

##### *N*-[2-[5-[(3-Fluorophenyl)methoxy]-1*H*-indol-3-yl]ethyl]-2-(hydroxycarbamoyl)-5-methyl-hexanamide (**24**)

To a solution of **24′** (212 mg, 0.452 mmol, 1.0 equiv) in MeOH (1 mL) was added aqueous hydroxylamine (50% in water, 1 mL), followed by DBU (25 μL). The solution was stirred overnight at rt, and solvents were then evaporated under vacuum. The residue was purified by flash chromatography on silica gel (DCM/MeOH, 100:0 to 90:10), affording the desired product as a pink solid (151 mg, 73%). Purity: >95%. LC tr = 2.68 min, MS (ESI+) *m*/*z*: 456 [M + H]^+^. HRMS *m*/*z*: calcd for C_25_H_31_N_3_O_4_ [M + H]^+^, 456.2699; found, 456.2296. ^1^H NMR (300 MHz, MeOD-*d*_4_): δ (ppm) 7.34 (td, *J* = 11.8, 5.8 Hz, 1H), 7.25–7.17 (m, 3H), 7.14 (d, *J* = 2.3 Hz, 1H), 7.04 (s, 1H), 7.01–6.95 (m, 1H), 6.83 (dd, *J* = 8.7, 2.3 Hz, 1H), 5.05 (s, 2H), 3.49–3.44 (m, 2H), 2.92–2.86 (m, 3H), 1.86–1.68 (m, 2H), 1.48 (sept, *J* = 6.6 Hz, 1H), 1.16–1.08 (m, 2H), 0.83 (d, *J* = 6.6 Hz, 6H). ^13^C NMR (75 MHz, MeOD-*d*_4_): δ (ppm) 171.9, 169.9, 164.2 (d, *J* = 244.2 Hz), 153.7, 142.3 (d, *J* = 7.3 Hz), 133.6, 131.1 (d, *J* = 8.2 Hz), 128.9, 124.5, 124.1 (d, *J* = 2.9 Hz), 115.2 (d, *J* = 21.0 Hz), 115.1 (d, *J* = 22.2 Hz), 113.2, 113.0, 112.7, 103.1, 71.1, 52.7, 41.2, 37.5, 29.9, 28.9, 26.1, 22.8, 22.7.

##### *N*-[2-[5-[(4-Fluorophenyl)methoxy]-1*H*-indol-3-yl]ethyl]-2-(hydroxycarbamoyl)-5-methyl-hexanamide (**25**)

To a solution of **25′** (268 mg, 0.572, 1.0 equiv) in MeOH (1 mL) was added aqueous hydroxylamine (50% in water, 1 mL), followed by DBU (25 μL). The mixture was stirred overnight at rt, and solvents were then evaporated under vacuum. The residue was purified by flash chromatography on silica gel (DCM/MeOH, 100:0 to 90:10), affording the desired product as a brown solid (195 mg, 75%). Purity: >95%. LC tr = 2.68 min, MS (ESI+) *m*/*z*: 456 [M + H]^+^. HRMS *m*/*z*: calcd for C_25_H_31_N_3_O_4_ [M + H]^+^, 456.2699; found, 456.2297. ^1^H NMR (300 MHz, MeOD-*d*_4_): δ (ppm) 7.46 (dd, *J* = 8.6, 5.5 Hz, 2H), 7.23 (d, *J* = 8.7 Hz, 1H), 7.15 (d, *J* = 2.3 Hz, 1H), 7.08 (d, *J* = 8.7 Hz, 2H), 7.04 (d, *J* = 1.0 Hz, 1H), 6.81 (dd, *J* = 8.7, 2.3 Hz, 1H), 5.04 (s, 2H), 3.47 (td, *J* = 6.9, 2.4 Hz, 2H), 2.91–2.85 (m, 3H), 1.83–1.72 (m, 2H), 1.60–1.08 (m, 2H), 1.49 (d, *J* = 6.6 Hz, 6H), 1.49 (sept, *J* = 6.6 Hz, 1H). ^13^C NMR (75 MHz, MeOD-*d*_4_): δ (ppm) 171.9, 169.9, 163.7 (d, *J* = 244.1 Hz), 153.9, 135.4 (d, *J* = 3.0 Hz), 133.6, 130.7 (d, *J* = 8.1 Hz), 129.0, 124.5, 116.0 (d, *J* = 21.6 Hz), 113.3, 113.0, 112.7, 103.2, 71.3, 52.8, 41.2, 37.5, 30.0, 28.9, 26.1, 22.8, 22.7.

##### 2-(Hydroxycarbamoyl)-5-methyl-*N*-[2-[5-(2-pyridylmethoxy)-1*H*-indol-3-yl]ethyl]hexanamide (**26**)

To a solution of **26′** (100 mg, 0.221 mmol, 1.0 equiv) in MeOH (2 mL) was added aqueous hydroxylamine (50% in water, 2 mL), followed by DBU (40 μL). The mixture was stirred overnight at rt, and solvents were then evaporated under vacuum. The residue was purified by flash chromatography on silica gel (DCM/MeOH, 100:0 to 90:10), affording the desired product as a colorless solid (48 mg, 49%). Purity: >95%. LC tr = 2.25 min, MS (ESI+) *m*/*z*: 439 [M + H]^+^. HRMS *m*/*z*: calcd for C_24_H_31_N_4_O_4_ [M + H]^+^, 439.2345; found, 439.2319. ^1^H NMR (300 MHz, DMSO-*d*_6_): δ (ppm) 10.68 (br s, 1H), 10.47 (br s, 1H), 8.93 (br s, 1H), 8.57 (br d, *J* = 4.8 Hz, 1H), 7.83 (td, *J* = 7.8, 1.7 Hz, 1H), 7.72 (t, *J* = 5.7 Hz, 1H), 7.57 (d, *J* = 7.8 Hz, 1H), 7.33 (ddd, *J* = 7.3, 4.9, 1.1 Hz, 1H), 7.24 (d, *J* = 8.7 Hz, 1H), 7.16 (d, *J* = 2.3 Hz, 1H), 7.10 (d, *J* = 1.6 Hz, 1H), 6.82 (dd, *J* = 8.7, 2.3 Hz, 1H), 5.17 (s, 2H), 3.34–3.28 (m, 4H), 2.84–2.73 (m, 3H), 1.74–1.60 (m, 2H), 1.47 (sept, *J* = 6.6 Hz, 1H), 1.04 (dd, *J* = 15.6, 7.1 Hz, 2H), 0.82 (d, *J* = 6.6 Hz, 6H). ^13^C NMR (75 MHz, DMSO-*d*_6_): δ (ppm) 168.9, 167.0, 157.5, 151.8, 149.0, 136.9, 131.7, 127.5, 123.6, 123.4, 122.8, 121.6, 112.0, 111.4, 101.9, 71.0, 51.0, 39.4, 36.1, 27.6, 27.3, 25.1, 22.4, 22.4.

##### 2-(Hydroxycarbamoyl)-5-methyl-*N*-[2-[5-(3-pyridylmethoxy)-1*H*-indol-3-yl]ethyl]hexanamide (**27**)

To a solution of **27′** (80 mg, 0.177 mmol, 1.0 equiv) in MeOH (2 mL) was added aqueous hydroxylamine (50% in water, 2 mL), followed by KCN (5 mg, 0.07 mmol, 0.2 equiv). The mixture was stirred overnight at rt, and solvents were then evaporated under vacuum. The residue was purified by flash chromatography on silica gel (DCM/MeOH, 100:0 to 90:10), affording the desired product as a white solid (28 mg, 36%). Purity: >95%. LC tr = 2.13 min, MS (ESI+) *m*/*z*: 439 [M + H]^+^. HRMS *m*/*z*: calcd for C_24_H_31_N_4_O_4_ [M + H]^+^, 439.2345; found, 439.2351. ^1^H NMR (300 MHz, DMSO-*d*_6_): δ (ppm) 10.69 (d, *J* = 1.9 Hz, 1H), 10.48 (br s, 1H), 8.94 (br s, 1H), 8.69 (dd, *J* = 2.2, 0.7 Hz, 1H), 8.53 (dd, *J* = 4.8, 1.7 Hz, 1H), 7.90 (ddd, *J* = 7.8, 2.2, 1.8 Hz, 1H), 7.73 (br t, *J* = 5.8 Hz, 1H), 7.42 (ddd, *J* = 7.8, 4.8, 0.8 Hz, 1H), 7.24 (d, *J* = 8.7 Hz, 1H), 7.18 (d, *J* = 2.4 Hz, 1H), 7.11 (d, *J* = 1.8 Hz, 1H), 6.80 (dd, *J* = 8.7, 2.4 Hz, 1H), 5.14 (s, 2H), 3.28–3.32 (m, 2H), 2.74–2.84 (m, 3H), 1.60–1.73 (m, 2H), 1.47 (sept, *J* = 6.6 Hz, 1H), 1.00–1.08 (m, 2H), 0.82 (dd, *J* = 6.6, 0.6 Hz, 6H). ^13^C NMR (75 MHz, DMSO-*d*_6_): δ (ppm) 168.9, 167.0, 151.7, 149.1, 149.0, 135.7, 133.3, 131.7, 127.5, 123.6, 112.0, 111.6, 111.5, 102.0, 67.6, 51.0, 39.4, 36.1, 27.7, 27.3, 25.1, 22.5, 22.4.

##### 2-(Hydroxycarbamoyl)-5-methyl-*N*-[2-[5-(4-pyridylmethoxy)-1*H*-indol-3-yl]ethyl]hexanamide (**28**)

To a solution of **28′** (95 mg, 0.210 mmol, 1.0 equiv) in MeOH (2 mL) was added aqueous hydroxylamine (50% in water, 2 mL) followed by KCN (5 mg, 0.07 mmol, 0.2 equiv). The mixture was stirred overnight at rt, and solvents were then evaporated under vacuum. The residue was purified by flash chromatography on silica gel (DCM/MeOH, 100:0 to 90:10), affording the desired product as a white solid (21 mg, 23%). Purity: >95%. LC tr = 2.07 min, MS (ESI+) *m*/*z*: 439 [M + H]^+^. HRMS *m*/*z*: calcd for C_24_H_31_N_4_O_4_ [M + H]^+^, 439.2345; found, 439.2343. ^1^H NMR (300 MHz, MeOD-*d*_4_): δ (ppm) 8.51 (br d, *J* = 5.0 Hz, 2H), 7.56 (br d, *J* = 5.9 Hz, 2H), 7.25 (d, *J* = 8.8 Hz, 1H), 7.16 (d, *J* = 2.4 Hz, 1H), 7.06 (s, 1H), 6.86 (dd, *J* = 8.8, 2.4 Hz, 1H), 5.20 (s, 2H), 3.54–3.42 (m, 2H), 2.94–2.83 (m, 3H), 1.84–1.68 (2H), 1.49 (sept, *J* = 6.6 Hz, 1H), 1.15–1.07 (m, 2H), 0.84 (d, *J* = 6.6 Hz, 6H). ^13^C NMR (75 MHz, MeOD-*d*_4_): δ (ppm) 172.0, 169.9, 153.5, 150.5, 150.0, 133.8, 129.0, 124.7, 123.4, 113.1, 112.8, 103.1, 69.9, 52.9, 41.2, 37.6, 30.0, 29.0, 26.1, 22.8, 22.7.

##### *N*-[2-[5-[(6-Chloro-3-pyridyl)methoxy]-1*H*-indol-3-yl]ethyl]-2-(hydroxycarbamoyl)-5-methyl-hexanamide (**29**)

To a solution of **29′** (125 mg, 0.257 mmol, 1.0 equiv) in MeOH (2 mL) was added aqueous hydroxylamine (50% in water, 2 mL), followed by DBU (40 μL). The mixture was stirred overnight at rt, and solvents were then evaporated under vacuum. The residue was purified by flash chromatography on silica gel (DCM/MeOH, 100:0 to 90:10) affording the desired product as an off white solid (45 mg, 37%). Purity: >95%. LC tr = 2.48 min, MS (ESI+) *m*/*z*: 473 [M + H]^+^. HRMS *m*/*z*: calcd for C_24_H_30_N_4_O_4_ [M + H]^+^, 473.1956; found, 473.1938. ^1^H NMR (300 MHz, MeOD-*d*_4_): δ (ppm) 8.46 (dd, *J* = 2.4, 0.5 Hz, 1H), 7.92 (dd, *J* = 8.3, 2.4 Hz, 1H), 7.45 (dd, *J* = 8.3, 0.5 Hz, 1H), 7.23 (d, *J* = 8.8 Hz, 1H), 7.18 (d, *J* = 2.3 Hz, 1H), 7.06 (s, 1H), 6.83 (dd, *J* = 8.7, 2.4 Hz, 1H), 5.13 (s, 2H), 3.48 (td, *J* = 7.1, 3.3 Hz, 2H), 2.90 (t, *J* = 7.2 Hz, 2H), 2.87 (t, *J* = 7.7 Hz, 1H), 1.80–1.71 (m, 2H), 1.49 (sept, *J* = 6.6 Hz, 1H), 1.15–1.07 (m, 2H), 0.84 (d, *J* = 6.6 Hz, 6H). ^13^C NMR (75 MHz, MeOD-*d*_4_): δ (ppm) 172.0, 169.9, 153.5, 151.4, 149.8, 140.4, 134.9, 133.8, 129.0, 125.4, 124.7, 113.3, 113.1, 112.8, 103.4, 68.6, 52.8, 41.3, 37.6, 30.0, 29.0, 26.1, 22.8, 22.8.

##### 2-(Hydroxycarbamoyl)-5-methyl-*N*-[2-[5-(pyrimidin-2-ylmethoxy)-1*H*-indol-3-yl]ethyl]hexanamide (**30**)

To a solution of **30′** (35 mg, 0.077 mmol, 1.0 equiv) in MeOH (1 mL) was added aqueous hydroxylamine (50% water, 1 mL), followed by DBU (12 μL). The mixture was stirred overnight at rt, and solvents were then evaporated under vacuum. The crude product was purified by flash chromatography on silica gel (DCM/MeOH, 99:1 to 95:5), affording the desired product as an off white solid (22 mg, 65%). Purity: >95%. LC tr = 2.03 min, MS (ESI+) *m*/*z*: 440 [M + H]^+^. HRMS *m*/*z*: calcd for C_23_H_30_N_5_O_4_ [M + H]^+^, 440.2298; found, 440.2297. ^1^H NMR (300 MHz, MeOD-*d*_4_): δ (ppm) 8.83 (d, *J* = 5.0 Hz, 2H), 7.44 (t, *J* = 5.0 Hz, 1H), 7.23 (d, *J* = 8.8 Hz, 1H), 7.14 (d, *J* = 2.4 Hz, 1H), 7.05 (s, 1H), 6.88 (dd, *J* = 8.8, 2.4 Hz, 1H), 5.30 (s, 2H), 3.46 (td, *J* = 7.1, 1.7 Hz, 1H), 2.87 (br q, *J* = 7.6 Hz, 3H), 1.81–1.71 (m, 2H), 1.50 (sept, *J* = 6.5 Hz, 1H), 1.16–1.08 (m, 2H), 0.85 (d, *J* = 6.6 Hz, 6H). ^13^C NMR (75 MHz, MeOD-*d*_4_): δ (ppm) 171.9, 169.9, 167.8, 158.8, 153.6, 133.8, 128.9, 124.6, 121.7, 113.2, 113.0, 112.8, 103.2, 72.6, 52.9, 41.2, 37.6, 30.0, 29.0, 26.1, 22.84, 22.75.

##### 2-(Hydroxycarbamoyl)-*N*-[2-[5-[2-(3-methoxyphenyl)ethoxy]-1*H*-indol-3-yl]ethyl]-5-methyl-hexanamide (**31**)

To a solution of compound **31′** (130 mg, 0.263 mmol, 1.0 equiv) in MeOH (2 mL) was added aqueous hydroxylamine (50% in water, 2 mL), followed by DBU (40 μL). The mixture was stirred overnight at rt, and solvents were then evaporated under vacuum. The residue was purified by flash chromatography on silica gel (DCM/MeOH, 100:0 to 90:10) affording the desired product as an off white solid (41 mg, 32%). Purity: >95%. LC tr = 2.72 min, MS (ESI+) *m*/*z*: 482 [M + H]^+^. HRMS *m*/*z*: calcd for C_27_H_36_N_3_O_5_ [M + H]^+^, 482.2655; found, 482.2628. ^1^H NMR (300 MHz, MeOD-*d*_4_): δ (ppm) 7.84 (br t, *J* = 5.7 Hz, 1H), 7.21 (d, *J* = 8.9 Hz, 1H), 7.18 (d, *J* = 8.1 Hz, 1H), 7.05 (d, *J* = 2.4 Hz, 1H), 7.04 (s, 1H), 6.90–6.87 (m, 2H), 6.78–6.73 (m, 2H), 4.21 (t, *J* = 6.9 Hz, 2H), 3.77 (s, 3H), 3.54–3.44 (m, 2H), 3.04 (t, *J* = 6.8 Hz, 2H), 2.89 (t, *J* = 7.2 Hz, 2H), 2.86 (t, *J* = 7.8 Hz, 1H), 1.79–1.70 (m, 2H), 1.49 (sept, *J* = 6.6 Hz, 1H), 1.15–1.07 (m, 2H), 0.84 (d, *J* = 6.6 Hz, 6H). ^13^C NMR (75 MHz, MeOD-*d*_4_): δ (ppm) 170.6, 168.5, 159.8, 152.6, 140.4, 132.2, 129.0, 127.6, 123.0, 121.0, 114.3, 111.9, 111.5, 111.4, 111.3, 101.4, 69.5, 54.2, 51.5, 39.8, 36.2, 35.7, 28.6, 27.6, 24.7, 21.4, 21.3.

##### 2-(Hydroxycarbamoyl)-5-methyl-*N*-[2-[5-(2-morpholinoethoxy)-1*H*-indol-3-yl]ethyl]hexanamide (**32**)

To a solution of **32′** (140 mg, 0.296 mmol, 1.0 equiv) in MeOH (2 mL) was added aqueous hydroxylamine (50% in water, 2 mL), followed by KCN (5 mg, 0.07 mmol, 0.2 equiv). The mixture was stirred overnight at rt, and solvents were then evaporated under vacuum. The residue was purified by flash chromatography on silica gel (DCM/MeOH, 100:0 to 90:10), affording the desired product as a white solid (83 mg, 61%). Purity: >95%. LC tr = 1.80 min, MS (ESI+) *m*/*z*: 461 [M + H]^+^. HRMS *m*/*z*: calcd for C_24_H_37_N_4_O_5_ [M + H]^+^, 461.2764; found, 461.2763. ^1^H NMR (300 MHz, MeOD-*d*_4_): δ (ppm) 7.22 (d, *J* = 8.8 Hz, 1H), 7.09 (d, *J* = 2.3 Hz, 1H), 7.05 (s, 1H), 6.78 (dd, *J* = 8.8, 2.3 Hz, 1H), 4.18 (t, *J* = 5.5 Hz, 2H), 3.75–3.72 (m, 4H), 3.56–3.40 (m, 2H), 2.93–2.84 (m, 5H), 2.68–2.65 (m, 4H), 1.80–1.71 (m, 2H), 1.50 (sept, *J* = 6.6 Hz, 1H), 1.16–1.08 (m, 2H), 0.85 (d, *J* = 6.6 Hz, 6H). ^13^C NMR (75 MHz, MeOD-*d*_4_): δ (ppm) 172.0, 169.9, 153.9, 133.6, 129.0, 124.5, 113.1, 113.0, 112.8, 102.6, 67.4, 67.0, 59.0, 55.1, 52.8, 41.3, 37.6, 30.1, 29.0, 26.1, 22.8, 22.8.

##### 2-(Hydroxycarbamoyl)-5-methyl-*N*-[2-[5-(2-morpholino-2-oxo-ethoxy)-1*H*-indol-3-yl]ethyl]hexanamide (**33**)

To a solution of **33′** (99 mg, 0.203 mmol, 1.0 equiv) in MeOH (1 mL) was added aqueous hydroxylamine (50% in water, 1 mL) followed by DBU (10 μL). The mixture was stirred for 7 h at rt, and solvents were then evaporated under vacuum. The residue was purified by flash chromatography on silica gel (DCM/MeOH, 100:0 to 90:10) to afford the desired product (16 mg, 17%). Purity: 100%. LC tr = 2.08 min, MS (ESI+) *m*/*z*: 475 [M + H]^+^. HRMS *m*/*z*: calcd for C_24_H_35_N_4_O_6_ [M + H]^+^, 475.2557; found, 475.2550. ^1^H NMR (300 MHz, MeOD-*d*_4_): δ (ppm) 7.24 (d, *J* = 8.8 Hz, 1H), 7.13 (d, *J* = 2.3 Hz, 1H), 7.06 (s, 1H), 6.83 (dd, *J* = 8.8, 2.3 Hz, 1H), 4.79 (s, 2H), 3.66–3.61 (m, 8H), 3.51–3.46 (m, 2H), 2.93–2.84 (m, 3H), 1.80–1.71 (m, 2H), 1.50 (s, *J* = 6.6 Hz, 1H), 1.20–1.08 (m, 2H), 0.85 (d, *J* = 6.6 Hz, 6H). ^13^C NMR (75 MHz, MeOD-*d*_4_): δ (ppm) 172.0, 169.9, 169.8, 153.2, 133.9, 128.9, 124.7, 113.1, 113.0, 112.9, 103.0, 68.8, 67.8, 67.7, 52.8, 46.9, 43.5, 41.3, 37.6, 30.0, 29.0, 26.1, 22.8.

##### 2-(Hydroxycarbamoyl)-5-methyl-*N*-[2-[5-[2-oxo-2-(prop-2-ynylamino)ethoxy]-1*H*-indol-3-yl]ethyl]hexanamide (**34**)

To a solution of **34′** (70 mg, 0.154 mmol, 1.0 equiv) in MeOH (1 mL) was added aqueous hydroxylamine (50% in water, 1 mL), followed by DBU (10 μL). The mixture was stirred for 7 h at rt, and solvents were then evaporated under vacuum. The residue was purified by flash chromatography on silica gel (DCM/MeOH, 100:0 to 90:10) to afford the desired product (21 mg, 30%). Purity: 96%, LC tr = 2.18 min, MS (ESI+) *m*/*z*: 443 [M + H]^+^. HRMS *m*/*z*: calcd for C_23_H_31_N_4_O_5_ [M + H]^+^, 443.2294; found, 443.2299. ^1^H NMR (300 MHz, MeOD-*d*_4_): δ (ppm) 7.26 (d, *J* = 8.7 Hz, 1H), 7.12 (d, *J* = 2.2 Hz, 1H), 7.07 (s, 1H), 6.87 (dd, *J* = 8.7, 2.2 Hz, 1H), 4.54 (s, 2H), 4.07 (d, *J* = 2.5 Hz, 2H), 3.51–3.46 (m, 2H), 2.93–2.84 (m, 3H), 2.58 (t, *J* = 2.5 Hz, 1H), 1.80–1.71 (m, 2H), 1.50 (s, *J* = 6.6 Hz, 1H), 1.16–1.08 (m, 2H), 0.85 (d, *J* = 6.6 Hz, 6H). ^13^C NMR (75 MHz, MeOD-*d*_4_): δ (ppm) 172.0, 171.7, 169.9, 153.0, 134.0, 129.0, 124.8, 113.1, 112.9, 103.4, 80.5, 72.1, 69.5, 52.8, 41.2, 37.5, 30.0, 29.1, 28.9, 26.1, 22.8, 22.7.

##### *N*-[2-[5-[2-(Benzylamino)-2-oxo-ethoxy]-1*H*-indol-3-yl]ethyl]-2-(hydroxycarbamoyl)-5-methyl-hexanamide (**35**)

To a solution of **35′** (105 mg, 0.207 mmol, 1.0 equiv) in MeOH (1 mL) was added aqueous hydroxylamine (50% in water, 1 mL) followed by DBU (31 μL). The mixture was stirred overnight at rt, and the reaction mixture was concentrated under reduced pressure. The residue was purified by flash chromatography on silica gel (DCM/MeOH, 100:0 to 95:5) affording the product as a white solid (67 mg, 66%). Purity: 96%. LC tr = 2.37 min, MS (ESI+) *m*/*z*: 495 [M + H]^+^. HRMS *m*/*z*: calcd for C_27_H_35_N_4_O_5_ [M + H]^+^, 495.2607; found, 495.2601. ^1^H NMR (300 MHz, MeOD-*d*_4_): δ (ppm) 7.26–7.17 (m, 6H), 7.12 (d, *J* = 2.3 Hz, 1H), 7.08 (s, 1H), 6.87 (dd, *J* = 8.9, 2.3 Hz, 1H), 4.60 (s, 2H), 4.48 (s, 2H), 3.62–3.52 (m, 1H), 3.50–3.45 (m, 2H), 2.92–2.88 (m, 3H), 1.79–1.73 (m, 3H), 1.14–1.12 (m, 1H), 0.85 (d, *J* = 6.7 Hz, 6H). ^13^C NMR (300 MHz, MeOD-*d*_4_): δ (ppm) 172.0, 171.9, 169.9, 153.0, 129.5, 129.0, 128.4, 128.2, 124.8, 113.1, 112.9, 103.3, 69.5, 52.8, 43.6, 41.2, 37.6, 30.1, 29.9, 29.0, 27.4, 26.1, 24.9, 22.8, 22.7.

##### 2-(Hydroxycarbamoyl)-*N*-[2-[5-[2-[2-(4-hydroxyphenyl)ethylamino]-2-oxo-ethoxy]-1*H*-indol-3-yl]ethyl]-5-methyl-hexanamide (**36**)

To a solution of **36′** (57 mg, 0.106 mmol, 1.0 equiv) in MeOH (1 mL) was added aqueous hydroxylamine (50% in water, 1 mL), followed by DBU (25 μL). The reaction mixture was stirred overnight at rt, and solvents were then evaporated under vacuum. The residue was purified by flash chromatography on silica gel (DCM/MeOH, 100:0 to 90:10), affording the desired product as a light-brownish solid (40 mg, 71.9%). Purity: 96%. LC tr = 2.18 min, MS (ESI+) *m*/*z*: 525 [M + H]^+^. HRMS *m*/*z*: calcd for C_28_H_37_N_4_O_6_ [M + H]^+^, 525.2713; found, 525.2704. ^1^H NMR (300 MHz, MeOD-*d*_4_): δ (ppm) 7.26 (d, *J* = 8.8 Hz, 1H), 7.10 (d, *J* = 2.4 Hz, 1H), 7.08 (s, 1H), 6.97–7.02 (m, 2H), 6.82 (dd, *J* = 8.8, 2.4 Hz, 1H), 6.64–6.69 (m, 2H), 4.50 (s, 2H), 3.52–3.43 (m, 4H), 2.91 (t, *J* = 7.0 Hz, 2H), 2.86 (t, *J* = 7.6 Hz, 1H), 2.72 (t, *J* = 7.5 Hz, 2H), 1.80–1.70 (m, 2H), 1.49 (sept, *J* = 6.6 Hz, 1H), 1.15–1.07 (m, 2H), 0.85 (d, *J* = 6.6 Hz, 6H). ^13^C NMR (75 MHz, MeOD-*d*_4_): δ (ppm) 172.0, 171.9, 169.9, 156.9, 153.0, 134.0, 131.0, 130.8, 129.0, 124.9, 116.3, 113.1, 112.9, 112.7, 103.4, 69.4, 52.9, 41.9, 41.2, 37.6, 35.7, 30.1, 29.0, 26.1, 22.8, 22.8.

##### 2-(Hydroxycarbamoyl)-*N*-[2-(1*H*-indol-3-yl)-1-methyl-ethyl]-4-methyl-pentanamide (**37**)

To a solution of **37′** (110 mg, 0.319 mmol, 1.0 equiv) in DMF (1 mL) was added aqueous hydroxylamine (50% in water, 1 mL) and KCN (1 mg, 0.013 mmol, 0.4 equiv). The reaction mixture was stirred overnight at rt, and the solvent was then removed under reduced pressure. The residue was purified by reverse flash chromatography on C18 silica gel (H_2_O/MeOH, 90:10 to 0:100) to afford the desired product as a white solid (37 mg, 35%) and mixture of 4 diastereoisomers (ratio: 45/55). Purity: >95%. LC tr = 1.71 min, MS (ESI+) *m*/*z*: 332 [M + H]^+^. HRMS *m*/*z*: calcd for C_18_H_26_N_3_O_3_ [M + H]^+^, 332.1974; found, 332.1972. ^1^H NMR (300 MHz, DMSO-*d*_6_): δ (ppm) 7.61–7.50 (m, 2H), 7.33 (s, 0.5H), 7.31 (s, 0.5H), 7.13–7.09 (m, 1H), 7.08–6.93 (m, 2H), 4.10–3.90 (m, 1H), 2.98–2.80 (m, 2H), 2.74–2.63 (m, 1H), 1.61–1.43 (m, 2H), 1.42–1.30 (m, 1H), 1.06–0.97 (m, 3H), 0.86–8.78 (m, 6H). ^13^C NMR (75 MHz, DMSO-*d*_6_): δ (ppm) 168.40, 168.37, 167.1, 166.9, 136.1, 127.5, 123.3, 120.8, 118.5, 118.2, 111.3, 111.1, 49.1, 49.0, 45.4, 37.1, 31.7, 31.6, 25.55, 25.50, 22.51, 22.46, 22.2, 22.1, 11.9, 19.8.

##### 2-(Hydroxycarbamoyl)-*N*-[(1*S*)-1-(hydroxymethyl)-2-(1*H*-indol-3-yl)ethyl]-4-methyl-pentanamide (**38**)

To a solution of **38′** (262 mg, 0.727 mmol, 1.0 equiv) in MeOH (2 mL) was added aqueous hydroxylamine (50% in water, 2 mL) and KCN (9.466 mg, 0.145 mmol, 0.2 equiv). The mixture was stirred overnight at rt and solvents were then removed under vacuum. The residue was purified by flash chromatography on silica gel (DCM/MeOH, 100:0 to 90:10) to give the product as a white powder (112 mg, 83%) and mixture of 2 diastereoisomers (ratio: 56/44). Purity: 100%. LC tr = 2.03 min, MS (ESI+) *m*/*z*: 348 [M + H]^+^. HRMS *m*/*z*: calcd for C_24_H_30_N_3_O_4_ [M + H]^+^, 424.2243; found, 424.2236. ^1^H NMR (300 MHz, MeOD-*d*_4_): δ (ppm) 7.65–7.60 (m, 1H), 7.34–7.30 (m, 1H), 7.10–6.97 (m, 3H), 4.28–4.18 (m, 1H), 3.57 (dd, *J* = 5.3, 6.4 Hz, 2H), 3.08–2.86 (m, 3H), 1.70–1.30 (m, 3H), 0.86–0.81 (m, 6H). ^13^C NMR (75 MHz, MeOD-*d*_4_): δ (ppm) 171.9, 171.8, 170.1, 170.0, 138.1, 129.0, 128.9, 124.3, 124.2, 122.3, 122.2, 119.7, 119.6, 119.5, 119.4, 112.2, 112.1, 112.0, 111.9, 64.1, 64.0, 53.4, 53.3, 51.0, 50.9, 41.2, 40.8, 27.6, 27.5, 27.2, 27.1, 22.9, 22.7, 22.6, 22.4.

##### 2-(Hydroxycarbamoyl)-*N*-[(1*R*)-1-(hydroxymethyl)-2-(1*H*-indol-3-yl)ethyl]-4-methyl-pentanamide (**39**)

To a solution of **39′** (262 mg, 0.727 mmol, 1.0 equiv) in MeOH (2 mL) was added aqueous hydroxylamine (50% in water, 2 mL) and KCN (9.466 mg, 0.145 mmol, 0.2 equiv). The mixture was stirred overnight at rt and solvents were removed under vacuum. The crude was purified by flash chromatography on silica gel (DCM/MeOH, 100:0 to 90:10) to give the product as a yellowish powder (193 mg, 75%) and mixture of 2 diastereoisomers (ratio: 41/59). Purity: 100%. LC tr = 2.03 min, MS (ESI+) *m*/*z*: 348 [M + H]^+^. HRMS *m*/*z*: calcd for C_18_H_26_N_3_O_4_ [M + H]^+^, 248.1923; found, 348.1917. ^1^H NMR (500 MHz, MeOD-*d*_4_): δ (ppm) 7.63–7.60 (m, 1H), 7.32 (s, 0.5H), 7.30 (s, 0.5H), 7.08–7.06 (m, 2H), 7.01–6.97 (m, 1H), 4.25–4.19 (m, 1H), 3.58–3.55 (m, 2H), 3.06–2.87 (m, 3H), 1.67–1.30 (m, 3H), 0.85–0.79 (m, 6H). ^13^C NMR (125 MHz, MeOD-*d*_4_): δ (ppm) 170.5, 170.4, 168.7, 168.6, 136.7, 127.6, 127.5, 122.9, 122.8, 120.9, 120.8, 118.3, 118.2, 118.1, 118.0, 110.8, 110.7, 110.7, 110.6, 62.8, 62.7, 52.0, 51.9, 49.6, 49.5, 39.8, 39.4, 26.2, 26.1, 25.8, 25.7, 21.5, 21.3, 21.2, 21.0.

##### *N*-[(1*S*)-2-Amino-1-(1*H*-indol-3-ylmethyl)-2-oxo-ethyl]-2-(hydroxycarbamoyl)-4-methyl-pentanamide (**40**)

To a solution of **40′** (200 mg, 0.536 mmol, 1.0 equiv) in 95% Ethanol (1.1 mL) was added aqueous hydroxylamine (50% in water, 164 μL) followed by cat. amount of KCN. The mixture was stirred at rt for 1.5 h and the solution was directly injected to C18 flash chromatography (H_2_O/ACN, 90:10 to 0:100). The product was obtained as a white powder (86 mg, 44.6%) and mixture of 2 diastereoisomers (ratio: 46/54). Purity = 100%. LC tr = 1.54 min, MS (ESI+) *m*/*z*: 361 [M + H]^+^, MS (ESI−) *m*/*z*: 359 [M − H]^−^. HRMS *m*/*z*: calcd for C_18_H_25_N_4_O_4_ [M + H]^+^, 361.1876; found, 361.1888.^1^H NMR (300 MHz, DMSO-*d*_6_): δ (ppm) 10.80 (s, 1H), 8.12 (d, *J* = 8.3 Hz, 0.5H), 8.04 (s, 0.5H), 7.92 (m, 1H), 7.74 (s, 0.5H), 7.55 (d, *J* = 7.7 Hz, 1H), 7.30 (d, *J* = 8.0 Hz, 1H), 7.12–6.89 (m, 4H), 4.39 (td, *J* = 8.6, 4.7 Hz, 1H), 3.24–3.06 (m, 1H), 3.05–2.74 (m, 2H), 1.45 (m, 2H), 1.28 (m, 1H), 0.74 (dt, *J* = 6.3, 2.7 Hz, 6H). ^13^C NMR (75 MHz, DMSO-*d*_6_): δ (ppm) 174.3, 174.0, 171.1, 167.0, 166.8, 136.5, 127.9, 127.8, 124.0, 123.8, 121.2, 111.6, 110.9, 110.5, 53.7, 53.6, 49.9, 49.1, 38.2, 27.9, 27.8, 26.0, 25.9, 23.0, 22.9, 22.7.

##### *N*-[2-(5-Benzyloxy-1*H*-indol-3-yl)ethyl]-3-(hydroxyamino)-3-oxo-2-(tetrahydropyran-4-ylmethyl)propenamide (**41**)

To a solution of **41′** (35 mg, 0.064 mmol, 1.0 equiv) in MeOH (4 mL) was added aqueous hydroxylamine (50 in water, 155 μL) and KCN (0.0154 mmol, 1 mg, 0.07 equiv). The reaction mixture was stirred at overnight at rt, and solvents were then evaporated under vacuum. The crude was purified by flash chromatography on silica gel (cyclohexane/ethyl acetate, 100:0 to 0:100) to afford the desired product (12.0 mg, 39%) as a white solid. Purity: 96%. LC tr = 2.43 min, MS (ESI+) *m*/*z*: 466 [M + H]^+^. HRMS *m*/*z*: calcd for C_26_H_31_N_3_O_5_ [M + H]^+^, 466.2331; found, 466.2342. ^1^H NMR (300 MHz, DMSO-*d*_6_): δ (ppm) 10.67 (s, 1H), 10.49 (s, 1H), 8.94 (s, 1H), 7.71 (t, *J* = 5.3 Hz, 1H), 7.49 (d, *J* = 7.5 Hz, 2H), 7.42–7.27 (m, 3H), 7.22 (d, *J* = 8.7 Hz, 1H), 7.15 (d, *J* = 2.3 Hz, 1H), 7.09 (d, *J* = 2.1 Hz, 1H), 6.46 (dd, *J* = 8.7, 2.4 Hz, 1H), 5.09 (s, 2H), 3.78 (d, *J* = 10.8 Hz, 2H), 3.16 (t, *J* = 11.5 Hz, 2H), 3.00 (t, *J* = 7.2 Hz, 1H), 2.76 (t, *J* = 7.2 Hz, 2H), 1.69–1.43 (m, 4H), 1.38–1.26 (m, 1H), 1.19–0.97 (m, 2H). ^13^C NMR (75 MHz, DMSO-*d*_6_): δ (ppm) 169.2, 167.3, 152.4, 138.3, 132.0, 128.8, 128.2, 128.1, 127.9, 129.9, 112.4, 112.0, 111.8, 102.3, 70.3, 67.4, 48.3, 37.0, 33.0, 32.8, 32.7, 25.5.

##### 2-(Cyclohexylmethyl)-3-(hydroxyamino)-3-oxo-*N*-[2-[5-(4-pyridylmethoxy)-1*H*-indol-3-yl]ethyl]propenamide (**42**)

To a solution of **42′** (91 mg, 0.196 mmol, 1.0 equiv) in MeOH (2 mL) was added aqueous hydroxylamine (50% in water, 1 mL), followed by DBU (25 μL). The solution was stirred overnight at rt, and solvents were then evaporated under vacuum. The residue was purified by flash chromatography on silica gel (DCM/MeOH 100:0 to 90:10), affording the desired product as a yellow solid (46 mg, 50%). Purity: 100%, LC tr = 2.21 min, MS (ESI+) *m*/*z*: 465 [M + H]^+^. HRMS *m*/*z*: calcd for C_26_H_33_N_4_O_4_ [M + H]^+^, 465.2502; found, 465.2503. ^1^H NMR (300 MHz, MeOD-*d*_4_): δ (ppm) 8.51 (br s, 2H), 7.56 (d, *J* = 4.9 Hz, 2H), 7.25 (d, *J* = 8.7 Hz, 1H), 7.15 (d, *J* = 2.3 Hz, 1H), 7.06 (s, 1H), 6.86 (dd, *J* = 8.7, 2.3 Hz, 1H), 5.19 (s, 2H), 3.46 (td, *J* = 7.1, 2.7 Hz, 2H), 3.05 (dd, *J* = 8.3, 7.2 Hz, 1H), 2.89 (t, *J* = 7.1 Hz, 2H), 1.71–1.52 (m, 7H), 1.24–1.09 (m, 4H), 0.93–0.76 (m, 2H). ^13^C NMR (75 MHz, MeOD-*d*_4_): δ (ppm) 153.5, 150.5, 150.0, 133.8, 129.0, 124.6, 123.5, 113.09, 113.06, 112.8, 103.1, 69.9, 50.3, 41.2, 39.5, 36.7, 34.3, 33.9, 27.5, 27.2, 26.

##### *N*-[(1*S*)-2-Amino-1-[(5-benzyloxy-1*H*-indol-3-yl)methyl]-2-oxo-ethyl]-2-(hydroxycarbamoyl)-4-methyl-pentanamide (**43**)

To the solution of **43′** (100 mg, 0.209 mmol, 1.0 equiv) in MeOH (1.2 mL) was added aqueous hydroxylamine (50% in water, 600 μL) and KCN (5 mg). The mixture was stirred at rt for 3 h and the solution was directly injected to C18 flash chromatography (H_2_O/ACN, 90:10 to 0:100). The desired product was obtained as white powder (50 mg, 50%) and mixture of 2 diastereoisomers (ratio: 45/55). Purity 100%. [α]_365_^20^ −12.6, [α]_D_^20^ −3.2. LC tr = 9.22 min, MS (ESI+) *m*/*z*: 467 [M + H]^+^. HRMS *m*/*z*: calcd for C_25_H_31_N_4_O_5_ [M + H]^+^, 467.2294; found, 467.2293. ^1^H NMR (500 MHz, DMSO-*d*_6_): δ (ppm) 10.68 (br s, 1.0H), 9.6 (br s, 1H), 8.06 (d, *J* = 7.0 Hz, 0.5H), 7.85 (d, *J* = 8.4 Hz, 0.4H), 7.81 (br s, 0.5H), 7.63 (br s, 0.4H), 7.50–7.48 (m, 2H), 7.40 (t, *J* = 7.3 Hz, 2H), 7.34–7.31 (m, 1H), 7.21–7.20 (m, 2H), 710 (d, *J* = 3.5 Hz, 1H), 7.06 (br s, 1H), 6.77 (dd, *J* = 8.7, 1.8 Hz, 1H), 5.09 (s, 2H), 4.45–4.42 (m, 1H), 3.14–3.07 (m, 1H), 3.01–2.96 (m, 1H), 2.91–2.86 (m, 1H), 1.49–1.39 (m, 2H), 1.31–1.23 (m, 1H), 0.77–0.73 (m, 6H). ^13^C NMR (125 MHz, DMSO-*d*_6_): δ (ppm) 173.7, 173.4, 169.7, 169.5, 166.8, 166.6, 152.1, 152.1, 137.9, 131.4, 131.3, 128.4, 127.8, 127.7, 127.6, 124.4, 124.2, 111.8, 111.4, 110.0, 109.7, 102.0, 101.9, 69.9, 69.8, 53.2, 53.1, 49.1, 48.6, 39.0, 37.9, 27.7, 25.5, 25.4, 22.5, 22.4, 22.2, 22.1.

##### *N*-[(1*S*)-2-Amino-2-oxo-1-[[5-(4-pyridylmethoxy)-1*H*-indol-3-yl]methyl]ethyl]-2-(hydroxycarbamoyl)-4-methyl-pentanamide (**44**)

To the solution of **44′** (20 mg, 0.041 mmol, 1.0 equiv) in MeOH (0.5 mL) was added aqueous hydroxylamine (50% in water, 50 μL) and KCN (5 mg). The reaction mixture was stirred for 3 h at rt and the solution was directly injected to C18 flash chromatography (H_2_O/ACN, 90:10 to 0:100). The desired product was obtained as a white powder (7 mg, 36%) and mixture of 2 diastereoisomers (ratio: 49/51). Purity 100%. LC tr = 6.24 min, MS (ESI+) *m*/*z*: 468 [M + H]^+^. HRMS *m*/*z*: calcd for C_24_H_30_N_5_O_5_ [M + H]^+^, 468.2247; found, 468.2238. ^1^H NMR (500 MHz, DMSO-*d*_6_): δ (ppm) 10.69 (d, *J* = 11.7 Hz, 1H), 8.57 (td, *J* = 5.1, 2.2 Hz, 2H), 8.25 (s, 1H), 8.08 (s, 0.3H), 7.89 (s, 0.5H),7.49–7.48 (m, 2H), 7.22–7.17 (m, 1.8H), 7.15–7.06 (m, 1.6H), 6.95 (d, *J* = 17.1 Hz, 0.6H), 6.79 (dt, *J* = 8.7, 2.2 Hz, 1H), 5.17–5.15 (m, 2H), 4.36 (s, 1H), 3.19–3.05 (m, 1.3H), 2.95–2.90 (m, 0.6H), 2.84–2.77 (s, 0.9H), 2.68 (s, 0.3H), 1.61–1.37 (m, 1.5H), 1.36–1.19 (m, 1.5H), 0.81–0.60 (m, 6H). ^13^C NMR (125 MHz, DMSO-*d*_6_): δ (ppm) 174.7, 174.5, 166.5, 161.7, 161.4, 152.0, 150.0, 147.4, 131.9, 131.9, 128.2, 128.1, 128.1, 124.9, 124.6, 122.3, 112.3, 111.7, 111.0, 110.8, 110.7, 102.5, 102.3, 68.6, 53.7, 53.6, 50.3, 49.2, 38.1, 27.8, 27.5, 26.1, 25.8, 25.6, 23.1, 22.9, 22.7, 22.4, 22.3, 22.2.

##### Methyl 4-Methyl-2-[(3-phenylphenyl)methylcarbamoyl]pentanoate (**1′**)

Compound **1′** was prepared from **45a** (200 mg, 1.15 mmol, 1.0 equiv), **46a** (231.4 mg, 1.26 mmol, 1.2 equiv), DMF (4 mL), HBTU (478 mg, 1.26 mmol, 1.1 equiv) and Et_3_N (471 μL, 3.44 mmol, 3.0 equiv) using general procedure G. After flash chromatography on silica gel (DCM/MeOH 100:0 to 90:10), the desired compound was obtained as an orange oil (42.9 mg, 10%). LC tr = 3.15 min, MS (ESI+) *m*/*z*: 340 [M + H]^+^. ^1^H NMR (300 MHz, CDCl_3_): δ (ppm) 7.59–7.32 (m, 9H), 7.26–7.22 (m, 1H), 4.51 (qd, *J* = 5.8, 14.7 Hz, 2H), 3.71–3.70 (m, 3H), 3.47 (s, 2H), 3.38 (t, *J* = 7.6 Hz, 1H), 1.82 (t, *J* = 7.2 Hz, 2H), 1.59 (sept, *J* = 6.5 Hz, 1H), 0.92 (d, *J* = 6.5 Hz, 6H). ^13^C NMR (75 MHz, CDCl_3_): δ (ppm) 172.8, 168.4, 141.7, 140.8, 138.5, 129.1, 128.8, 127.4, 127.1, 126.5, 126.4, 126.3, 52.4, 51.6, 43.6, 40.1, 26.4, 22.4, 22.1.

##### Ethyl 4-Methyl-2-[(4-phenylphenyl)methylcarbamoyl]pentanoate (**2′**)

Compound **2′** was prepared from **45b** (150 mg, 0.59 mmol, 1.0 equiv), **46b** (131 mg, 0.72 mmol, 1.2 equiv), DMF (4 mL), EDCI (154.7 mg, 0.81 mmol, 1.35 equiv) HOBT (88 mg, 0.66 mmol, 1.1 equiv) and Et_3_N (245 μL, 1.79 mmol, 3.0 equiv) using general procedure G. After flash chromatography on silica gel (DCM/MeOH 100:0 to 90:10), the desired compound was obtained as a yellowish solid (125 mg, 59%). LC tr = 3.22 min, MS (ESI+) *m*/*z*: 354 [M + H]^+^.^1^H NMR (300 MHz, CDCl_3_): δ (ppm) 7.58–7.53 (m, 4H), 7.46–7.40 (m, 2H), 7.37–7.30 (m, 6H), 6.89 (br t, 1H), 4.53 (dd, *J* = 6.0, 14.9 Hz, 1H), 4.43 (dd, *J* = 5.6, 14.9 Hz, 1H), 4.18 (q, *J* = 7.0 Hz, 2H), 3.37 (t, *J* = 7.7 Hz, 1H), 1.85–1.79 (m, 2H), 1.60 (sept, *J* = 6.7 Hz, 1H), 1.26 (t, *J* = 7.1 Hz, 3H), 0.94 (dd, *J* = 2.0, 6.6 Hz, 6H). ^13^C NMR (75 MHz, CDCl_3_) δ: 172.5, 168.7, 140.7, 140.4, 137.1, 128.8, 128.0, 127.4, 127.3, 127.0, 61.5, 51.7, 43.3, 40.1, 26.3, 22.5, 22.1, 14.1.

##### Ethyl 4-Methyl-2-(1,3,4,5-tetrahydropyrido[4,3-*b*]indole-2-carbonyl)pentanoate (**3′**)

Compound **3′** was prepared from **45b** (60 mg, 0.319 mmol, 1.0 equiv), **46c** (55 mg, 0.319 mmol, 1.0 equiv), DMF (1 mL), HBTU (121 mg, 0.319 mmol, 1.0 equiv), Et_3_N (163 μL, 0.95 mmol, 3.0 equiv) using general procedure G. The desired product as a colorless oil (109 mg, quant). LC tr = 2.21 min, MS (ESI+) *m*/*z*: 343 [M + H]^+^. Mixture of 2 rotamers: ^1^H NMR (300 MHz, MeOD-*d*_4_): δ (ppm) 7.37 (br d, *J* = 7.7 Hz, 1H), 7.28 (br d, *J* = 7.7 Hz, 1H), 7.05 (br t, *J* = 7.1 Hz, 1H), 6.97 (br t, *J* = 7.4 Hz, 1H), 4.75 (br s, 2H), 4.18–4.07 (m, 2H), 4.02–3.73 (m, 3H), 2.87–2.67 (m, 2H), 1.76–1.73 (m, 2H), 1.63–1.48 (m, 1H), 1.23–1.14 (m, 2H), 0.99–0.84 (m, 7H). ^13^C NMR (75 MHz, MeOD-*d*_4_): δ (ppm) 171.6, 171.4, 170.6, 170.4, 137.9, 131.1, 130.7, 128.04, 128.01, 122.3, 122.2, 119.9, 118.6, 118.5, 111.9, 109.1, 108.0, 62.4, 62.3, 48.8, 48.3, 45.7, 45.3, 42.6, 42.1, 39.1, 38.8, 27.34, 27.32, 22.94, 22.88, 22.7, 21.7, 14.4, 14.0.

##### Methyl 2-[2-(1*H*-Imidazole-4-yl)ethylcarbamoyl]-4-methyl-pentanoate (**4′**)

Compound **4′** was prepared from **45b** (300 mg, 1.722 mmol, 1.0 equiv), **46d** (210.5 mg, 1.89 mmol, 1.2 equiv), DCM (5 mL), HBTU (718.5 mg, 1.89 mmol, 1.1 equiv), Et_3_N (707 μL, 5.167 mmol, 3.0 equiv) using general procedure G. After flash chromatography on silica gel (DCM/MeOH 100:0 to 90:10), the desired compound was obtained as a colorless oil (452 mg, 98%). LC tr = 1.63 min, MS (ESI+) *m*/*z*: 268 [M + H]^+^. ^1^H NMR (300 MHz, CDCl_3_): δ (ppm) 7.73 (br s, 1H), 7.54 (s, 1H), 7.42–7.39 (m, 1H), 6.8 (s, 1H), 3.68 (s, 3H), 3.53–3.51 (m, 2H), 3.34 (t, *J* = 7.6 Hz, 1H), 2.81 (t, *J* = 6.4 Hz, 2H), 1.74 (t, *J* = 7.1 Hz, 2H), 1.50 (sept, *J* = 6.6 Hz, 1H), 0.88 (d, *J* = 6.6 Hz, 6H). ^13^C NMR (75 MHz, CDCl_3_): δ (ppm) 172.2, 169.1, 134.9, 117.1, 52.4, 51.4, 39.6, 39.1, 26.6, 26.2, 22.3, 22.2.

##### Methyl 2-(3-Imidazole-1-ylpropylcarbamoyl)-4-methyl-pentanoate (**5′**)

Compound **5′** was prepared from **45b** (200 mg, 1.148 mmol, 1.0 equiv), **46e** (245.9 mg, 1.26 mmol, 1.2 equiv), DMF (4 mL), HBTU (478.9 mg, 1.26 mmol, 1.1 equiv), Et_3_N (471 μL, 3.44 mmol, 3.0 equiv) using general procedure G. After flash chromatography on silica gel (cyclohexane/EtOAc 90:10 to 50:50), the desired compound was obtained as a yellow oil (323 mg, 79%). LC tr = 1.63 min, MS (ESI+) *m*/*z*: 282 [M + H]^+^. ^1^H NMR (300 MHz, CDCl_3_): δ (ppm) 9.36 (s, 1H), 8.25 (s, 1H), 7.35–7.22 (m, 3H), 4.17 (t, *J* = 6.8 Hz, 2H), 3.70 (s, 3H), 3.43 (dd, *J* = 6.0, 7.5 Hz, 1H), 2.08 (quint, *J* = 6.7 Hz, 2H), 1.76 (t, *J* = 7.3 Hz, 2H), 1.57 (sept, *J* = 6.7 Hz, 1H), 0.90 (d, *J* = 6.7 Hz, 6H). ^13^C NMR (75 MHz, CDCl_3_): δ (ppm) 171.7, 170.2, 135.9, 123.6, 121.0, 52.4, 50.9, 45.7, 38.5, 36.2, 30.2, 26.2, 22.4, 22.1.

##### Methyl 2-[2-(1*H*-Indol-3-yl)ethylcarbamoyl]-4-methyl-pentanoate (**6′**)

Compound **6′** was prepared from **45b** (130 mg, 0.75 mmol, 1.0 equiv), **46f** (143 mg, 0.90 mmol, 1.2 equiv), DMF (4 mL), HBTU (379 mg, 0.82 mmol, 1.1 equiv), Et_3_N (306 μL, 2.24 mmol, 3.0 equiv) using general procedure G. After flash chromatography on silica gel (cyclohexane/EtOAc 90:10 to 50:50), the desired compound was obtained as a thick wax. LC tr = 3.05 min, MS (ESI+) *m*/*z*: 317 [M + H]^+^. ^1^H NMR (300 MHz, CDCl_3_): δ (ppm) 8.85 (br s, 1H), 7.57 (br d, *J* = 7.8 Hz, 1H), 7.33 (ddd, *J* = 8.0, 1.0, 0.9 Hz), 7.16 (ddd, *J* = 8.0, 7.0, 1.2 Hz, 1H), 7.07 (ddd, *J* = 7.8, 7.0, 1.2 Hz, 1H), 6.95 (d, *J* = 2.3 Hz, 1H), 6.59 (t, *J* = 5.7 Hz, 1H), 3.61 (s, 3H), 3.57 (td, *J* = 6.9, 5.8 Hz, 2H), 3.27 (t, *J* = 7.7 Hz, 1H), 2.94 (t, *J* = 6.9 Hz, 2H), 1.75–1.70 (m, 2H), 1.50 (sept, *J* = 6.6 Hz, 1H), 0.85 (dd, *J* = 6.6, 1.9 Hz, 6H). ^13^C NMR (75 MHz, CDCl_3_): δ (ppm) 172.3, 168.7, 136.5, 127.3, 122.4, 121.8, 119.1, 118.5, 112.2, 111.4, 52.3, 51.5, 40.1, 39.4, 26.2, 25.1, 22.3, 22.2.

##### Methyl 2-[2-(1*H*-Indol-3-yl)ethylcarbamoyl]-5-methyl-hexanoate (**7′**)

Compound **7′** was prepared from **45c** (150 mg, 0.79 mmol, 1.0 equiv), **46f** (153.2 mg, 0.95 mmol, 1.2 equiv), DMF (4 mL), HBTU (332.5, 0.88 mmol, 1.1 equiv), Et_3_N (327 μL, 2.391 mmol, 3.0 equiv) using general procedure G. After flash chromatography on silica gel (cyclohexane/EtOAc 90:10 to 50:50), the desired compound was obtained as a colorless oil (201 mg, 76%). LC tr = 2.87 min, MS (ESI+) *m*/*z*: 331 [M + H]^+^. ^1^H NMR (300 MHz, CDCl_3_): δ (ppm) 8.46 (br s, 1H), 7.59 (d, *J* = 7.8 Hz, 1H), 7.34 (d, *J* = 8.0 Hz, 1H), 7.18 (t, *J* = 7.2 Hz, 1H), 7.10 (t, *J* = 7.2 Hz, 1H), 6.99 (s, 1H), 6.52 (br s, 1H), 3.64 (s, 3H), 3.56–3.60 (m, 2H), 3.11 (t, *J* = 7.4 Hz, 1H), 2.96 (t, *J* = 6.8 Hz, 2H), 1.87–1.79 (m, 2H), 1.49 (sept, *J* = 6.7 Hz, 1H), 1.07–1.17 (m, 2H), 0.84 (d, *J* = 6.6 Hz, 6H). ^13^C NMR (75 MHz, CDCl_3_): δ (ppm) 172.4, 168.5, 136.5, 127.3, 122.1, 122.0, 119.3, 118.6, 112.6, 111.3, 53.4, 52.3, 40.0, 36.3, 28.8, 27.8, 25.2, 22.4, 22.3.

##### Methyl 2-[2-(1*H*-Indol-3-yl)ethylcarbamoyl]-5,5-dimethyl-hexanoate (**8′**)

Compound **8′** was prepared from **45d** (160.7 mg, 0.795 mmol, 1.0 equiv) and **46f** (152.8 mg, 0.953 mmol, 1.2 equiv), DMF (4 mL), HBTU (331.5, 0.874 mmol, 1.1 equiv), Et_3_N (326 μL, 2.384 mmol, 3.0 equiv) using general procedure G. After flash chromatography on silica gel (cyclohexane/EtOAc 90:10 to 50:50), the desired compound was obtained as a colorless oil (281 mg, quant.). LC tr = 2.97 min, MS (ESI+) *m*/*z*: 345 [M + H]^+^. ^1^H NMR (300 MHz, CDCl_3_): δ (ppm) 8.60 (s, 1H), 7.58 (d, *J* = 7.8 Hz, 1H), 7.35–7.33 (m, 1H), 7.17 (td, *J* = 1.2, 7.0 Hz, 1H), 7.09 (td, *J* = 1.2, 8.0 Hz, 1H), 6.97 (t, *J* = 2.3 Hz, 1H), 6.54 (t, *J* = 5.5 Hz, 1H), 3.63–3.56 (m, 5H), 3.07 (t, *J* = 7.4 Hz, 2H), 2.96 (t, *J* = 6.8 Hz, 2H), 1.78–1.86 (m, 2H), 1.07–1.16 (m, 2H), 0.84 (s, 9H). ^13^C NMR (75 MHz, CDCl_3_): δ (ppm) 172.3, 168.6, 136.5, 127.3, 122.3, 122.0, 119.3, 118.6, 112.4, 111.1, 53.8, 52.3, 41.3, 40.1, 30.2, 29.1, 26.2, 25.2.

##### Methyl 2-[2-(1*H*-Indol-3-yl)ethylcarbamoyl]-4-phenyl-butanoate (**9′**)

Compound **9′** was prepared from **45e** (160 mg, 0.72 mmol, 1.0 equiv), **46f** (138 mg, 0.86 mmol, 1.2 equiv), DMF (4 mL), HBTU (300 mg, 0.79 mmol, 1.1 equiv), Et_3_N (295 μL, 2.16 mmol, 3.0 equiv) using general procedure G. After flash chromatography on silica gel (cyclohexane/EtOAc 90:10 to 50:50), the desired compound was obtained as a light brown wax (221 mg, 84%). LC tr = 2.70 min, MS (ESI+) *m*/*z*: 365 [M + H]^+^. ^1^H NMR (300 MHz, CDCl_3_): δ (ppm) 8.60 (br s, 1H), 7.56 (d, *J* = 7.8 Hz, 1H), 7.31 (dq, *J* = 8.0, 0.9 Hz, 1H), 7.24–7.12 (m, 4H), 7.10–7.05 (m, 3H), 6.93 (br s, 1H), 6.45 (br t, *J* = 5.4 Hz, 1H), 3.57 (q, *J* = 6.8 Hz, 2H), 3.57 (s, 3H), 3.13 (t, *J* = 7.4 Hz, 1H), 2.94 (t, *J* = 6.8 Hz, 2H), 2.47–2.63 (m, 2H), 2.15 (q, *J* = 7.6 Hz, 2H). ^13^C NMR (75 MHz, CDCl_3_): δ (ppm) 171.7, 168.2, 140.5, 136.4, 128.4, 127.2, 126.1, 122.3, 119.2, 118.5, 112.3, 111.4, 52.33, 52.30, 40.0, 33.2, 31.6, 25.1.

##### Ethyl 2-[2-(5-Fluoro-1*H*-indol-3-yl)ethylcarbamoyl]-4-methyl-pentanoate (**10′**)

Compound **10′** was prepared from **45b** (60 mg, 0.319 mmol, 1.0 equiv), Et_3_N (163 μL, 0.95 mmol, 3.0 equiv), **46g** (68 mg, 0.319 mmol, 1.0 equiv), HBTU (121 mg, 0.319 mmol, 1. equiv), DMF (1 mL) using general procedure G. The desired product was obtained as a colorless oil (102 mg, quant). LC tr = 2.06 min, MS (ESI+) *m*/*z*: 349 [M + H]^+^. ^1^H NMR (300 MHz, MeOD-*d*_4_): δ (ppm) 7.28 (dd, *J* = 8.9, 4.7 Hz, 1H), 7.23 (dd, *J* = 9.9, 2.5 Hz, 1H), 7.12 (s, 1H), 6.85 (td, *J* = 9.0, 2.4 Hz, 1H), 4.11 (br q, *J* = 6.7 Hz, 2H), 3.57–3.42 (m, 2H), 3.35–3.30 (m, 1H), 2.91 (t, *J* = 6.9 Hz, 2H), 1.74–1.60 (m, 2H), 1.48–1.35 (m, 1H), 1.20 (t, *J* = 7.1 Hz, 3H), 0.85 (t, *J* = 6.2 Hz, 6H). ^13^C NMR (75 MHz, MeOD-*d*_4_): δ (ppm) 172.0, 171.5, 158.8 (d, *J* = 232.0 Hz), 134.6, 129.1 (d, *J* = 9.3 Hz), 125.6, 113.3 (d, *J* = 4.9 Hz), 112.9 (d, *J* = 9.5 Hz), 110.3 (d, *J* = 26.8 Hz), 103.9 (d, *J* = 23.4 Hz), 62.2, 52.2, 41.3, 39.1, 27.1, 25.9, 23.0, 22.3, 14.4.

##### Ethyl 2-[2-(6-Fluoro-1*H*-indol-3-yl)ethylcarbamoyl]-4-methyl-pentanoate (**11′**)

Compound **11′** was prepared from **45b** (60 mg, 0.319 mmol, 1.0 equiv), Et_3_N (163 μL, 0.95 mmol, 3.0 equiv), **46h** (68 mg, 0.319 mmol, 1.0 equiv), HBTU (121 mg, 0.319 mmol, 1.0 equiv), DMF (1 mL) using general procedure G. The desired product was obtained as a colorless oil (102 mg, quant). LC tr = 2.06 min, MS (ESI+) *m*/*z*: 349 [M + H]^+^. ^1^H NMR (300 MHz, MeOD-*d*_4_): δ (ppm) 7.50 (dd, *J* = 8.7, 5.3 Hz, 1H), 7.05 (s, 1H), 7.01 (dd, *J* = 10.1, 2.3 Hz, 1H), 6.82–6.75 (m, 1H), 4.10 (qd, *J* = 7.1, 1.4 Hz, 2H), 3.58–3.43 (m, 2H), 3.34–3.29 (m, 1H), 2.93 (t, *J* = 7.1 Hz, 2H), 1.71–1.59 (m, 2H), 1.48–1.35 (m, 1H), 1.20 (t, *J* = 7.1 Hz, 3H), 0.85 (t, *J* = 6.3 Hz, 6H). ^13^C NMR (75 MHz, MeOD-*d*_4_): δ (ppm) 172.0, 171.6, 161.1 (d, *J* = 235.3 Hz), 138.0 (d, *J* = 12.4 Hz), 125.5, 124.0 (d, *J* = 3.5 Hz), 120.1 (d, *J* = 10.1 Hz), 113.3, 108.0 (d, *J* = 24.7 Hz), 98.1 (d, *J* = 26.4 Hz), 62.2, 52.2, 41.3, 39.0, 27.1, 26.0, 23.0, 22.3, 14.4.

##### Methyl 2-[2-(5-Chloro-1*H*-indol-3-yl)ethylcarbamoyl]-4-methyl-pentanoate (**12′**)

Compound **12′** was prepared from **45b** (200 mg, 1.148 mmol, 1.0 equiv), **46i** (245.9 mg, 1.263 mmol, 1.2 equiv), DMF (4 mL), HBTU (478.9 mg, 1.263 mmol, 1.1 equiv), Et_3_N (471 μL, 3.444 mmol, 3.0 equiv) using general procedure G. After flash chromatography on silica gel (cyclohexane/EtOAc 90:10 to 50:50), the desired compound was obtained as a colorless oil (209 mg, 52%). LC tr = 2.87 min, MS (ESI+) *m*/*z*: 351 [M + H]^+^. ^1^H NMR (300 MHz, CDCl_3_): δ (ppm) 9.12 (s, 1H), 7.52 (s, 1H), 7.27–7.24 (m, 1H), 7.10–7.07 (m, 1H), 6.99 (s, 1H), 6.69 (t, *J* = 5.2 Hz, 1H), 3.62 (s, 3H), 3.55 (q, *J* = 6.5, 12.6 Hz, 2H), 3.30 (t, *J* = 7.6 Hz, 1H), 2.92–2.84 (m, 2H), 1.78–1.68 (m, 2H), 1.57–1.45 (m, 1H), 0.85 (d, *J* = 6.6 Hz, 6H). ^13^C NMR (75 MHz, CDCl_3_): δ (ppm) 172.3, 168.9, 134.8, 128.4, 124.8, 123.8, 122.0, 117.9, 112.5, 112.0, 52.4, 51.5, 40.2, 39.5, 26.2, 24.9, 22.3, 22.1.

##### Ethyl 2-[2-(5-Bromo-1*H*-indol-3-yl)ethylcarbamoyl]-4-methyl-pentanoate (**13′**)

Compound **13′** was prepared from **45b** (250 mg, 0.797 mmol, 1.0 equiv), **46J** (228.7 mg, 0.956 mmol, 1.2 equiv), DMF (4 mL), HBTU (332.5 mg, 0.877 mmol, 1.1 equiv), Et_3_N (327 μL, 2.291 mmol, 3.0 equiv) using general procedure G. After flash chromatography on silica gel (cyclohexane/EtOAc 90:10 to 50:50), the desired compound was obtained a colorless oil (340 mg, quant.). LC tr = 3.05 min, MS (ESI+) *m*/*z*: 411 [M + H]^+^. ^1^H NMR (300 MHz, CDCl_3_): δ (ppm) 8.90 (s, 1H), 7.69 (s, 1H), 7.26–7.22 (m, 2H), 7.23 (d, *J* = 0.8 Hz, 1H), 6.63 (t, *J* = 5.6 Hz, 1H), 4.16–4.05 (m, 2H), 3.59–3.53 (m, 2H), 3.27 (dd, *J* = 7.2, 9.1 Hz, 1H), 2.90 (t, *J* = 6.9 Hz, 2H), 1.78–1.67 (m, 2H), 1.51 (sept, *J* = 6.7 Hz, 1H), 1.20 (t, *J* = 7.1 Hz, 3H), 0.87 (d, *J* = 6.6 Hz, 6H). ^13^C NMR (75 MHz, CDCl_3_): δ (ppm) 172.2, 168.9, 135.1, 129.1, 124.7, 123.5, 121.1, 112.9, 112.5, 112.1, 51.7, 40.0, 39.9, 38.6, 26.9, 26.3, 25.0, 22.4, 22.1.

##### Ethyl 4-Methyl-2-[2-(5-methyl-1*H*-indol-3-yl)ethylcarbamoyl]pentanoate (**14′**)

Compound **14′** was prepared from **45b** (60 mg, 0.319 mmol, 1.0 equiv), Et_3_N (163 μL, 0.95 mmol, 3.0 equiv), **47k** (67 mg, 0.319 mmol, 1.0 equiv), HBTU (121 mg, 0.319 mmol, 1.1 equiv), DMF (1 mL) using general procedure G. The desired product was obtained as a colorless oil (110 mg, quant). LC tr = 2.13 min, MS (ESI+) *m*/*z*: 345 [M + H]^+^. ^1^H NMR (300 MHz, MeOD-*d*_4_): δ (ppm) 7.50 (br quint, *J* = 0.7 Hz, 1H), 7.20 (d, *J* = 8.1 Hz, 1H), 7.01 (s, 1H), 6.91 (dd, *J* = 8.4, 1.6 Hz, 1H), 4.10 (qd, *J* = 7.2, 1.5 Hz, 2H), 3.58–3.44 (m, 2H), 3.35–3.29 (m, 2H), 2.92 (t, *J* = 7.1 Hz, 2H), 2.41 (s, 3H), 1.74–1.57 (m, 2H), 1.50–1.36 (m, 1H), 1.20 (t, *J* = 7.1 Hz, 3H), 0.86 (t, *J* = 6.3 Hz, 6H). ^13^C NMR (75 MHz, MeOD-*d*_4_): δ (ppm) 172.0, 171.5, 136.5, 129.0, 128.6, 123.9, 123.6, 118.9, 112.5, 111.9, 62.2, 52.2, 41.4, 39.1, 27.1, 26.0, 23.0, 22.3, 21.7, 14.4.

##### Ethyl 2-[2-(5-Hydroxy-1*H*-indol-3-yl)ethylcarbamoyl]-4-methyl-pentanoate (**15′**)

Compound **15′** was prepared from **45b** (60 mg, 0.319 mmol, 1.0 equiv), Et_3_N (163 μL, 0.95 mmol, 3.0 equiv), **46l** (68 mg, 0.319 mmol, 1.0 equiv), HBTU (121 mg, 0.319 mmol, 1.1 equiv), DMF (1 mL) using general procedure G. The desired product was obtained as a colorless oil (110 mg, quant). LC tr = 2.77 min, MS (ESI+) *m*/*z*: 347 [M + H]^+^. ^1^H NMR (300 MHz, MeOD-*d*_4_): δ (ppm) 7.16–7.14 (m, 1H), 7.00 (s, 1H), 6.66 (d, *J* = 8.6 Hz, 1H), 4.11 (qd, *J* = 7.2, 1.5 Hz, 2H), 3.53–3.45 (m, 2H), 3.33–3.29 (m, 1H), 2.87 (t, *J* = 6.7 Hz, 2H), 1.71–1.63 (m, 2H), 1.50–1.37 (m, 1H), 1.20 (t, *J* = 7.2 Hz, 3H), 0.86 (dd, *J* = 6.6, 5.7 Hz, 6H). ^13^C NMR (75 MHz, MeOD-*d*_4_): δ (ppm) 172.0, 171.5, 151.1, 133.1, 129.3, 124.3, 112.6, 112.5, 112.3, 112.2, 62.2, 52.2, 41.3, 39.1, 27.1, 26.1, 23.0, 22.3, 14.4.

##### Ethyl 2-[2-(5-Methoxy-1*H*-indol-3-yl)ethylcarbamoyl]-4-methyl-pentanoate (**16′**)

Compound **16′** was prepared from **45b** (60 mg, 0.319 mmol, 1.0 equiv), Et_3_N (163 μL, 0.95 mmol, 3.0 equiv), **46m** (72 mg, 0.319 mmol, 1.0 equiv), HBTU (121 mg, 0.319 mmol, 1.1 equiv), DMF (1 mL) using general procedure G. The desired product was obtained as a colorless oil (45 mg, quant). LC tr = 3.10 min, MS (ESI+) *m*/*z*: 361 [M + H]^+^. ^1^H NMR (300 MHz, MeOD-*d*_4_): δ (ppm) 7.23 (d, *J* = 8.8 Hz, 1H), 7.10–7.06 (m, 1H), 7.05 (br s, 1H), 6.76 (d, *J* = 8.8 Hz, 1H), 4.12 (qd, *J* = 7.0, 2.0 Hz, 2H), 3.84 (s, 3H), 3.57–3.47 (m, 2H), 3.34–3.31 (m, 1H), 2.94 (t, *J* = 7.0 Hz, 2H), 1.72–1.65 (m, 2H), 1.51–1.38 (m, 1H), 1.22 (t, *J* = 7.0 Hz, 3H), 0.88 (d, *J* = 5.7 Hz, 3H), 0.86 (d, *J* = 5.6 Hz, 3H). ^13^C NMR (75 MHz, MeOD-*d*_4_): δ (ppm) 172.0, 171.5, 155.1, 133.3, 129.0, 124.3, 112.8, 112.7, 112.5, 101.3, 62.2, 56.3, 52.2, 41.3, 39.1, 27.1, 26.0, 23.0, 22.3, 14.4.

##### Ethyl 2-[2-(6-Methoxy-1*H*-indol-3-yl)ethylcarbamoyl]-4-methyl-pentanoate (**17′**)

Compound **17′** was prepared from **45b** (60 mg, 0.319 mmol, 1.0 equiv), Et_3_N (163 μL, 0.95 mmol, 3.0 equiv), **46n** (68 mg, 0.319 mmol, 1.0 equiv), HBTU (121 mg, 0.319 mmol, 1.1 equiv), DMF (1 mL) using general procedure G. The desired product was obtained as a colorless oil (115 mg, quant). LC tr = 3.10 min, MS (ESI+) *m*/*z*: 361 [M + H]^+^. ^1^H NMR (300 MHz, MeOD-*d*_4_): δ (ppm) 7.44–7.39 (m, 1H), 6.86 (br s, 1H), 6.69–6.65 (m, 1H), 4.11 (qd, *J* = 7.0, 1.2 Hz, 2H), 3.79 (s, 3H), 3.53–3.46 (m, 2H), 3.35–3.32 (m, 1H), 2.93–2.87 (m, 2H), 1.69–1.64 (m, 2H), 1.57–1.50 (m, 1H), 1.20 (t, *J* = 7.0 Hz, 3H), 0.87 (d, *J* = 5.7 Hz, 3H), 0.84 (d, *J* = 5.5 Hz, 3H). ^13^C NMR (75 MHz, MeOD-*d*_4_): δ (ppm) 172.0, 171.5, 157.6, 138.8, 123.3, 119.8, 119.7, 112.8, 109.8, 95.5, 62.2, 55.9, 52.2, 41.4, 39.1, 27.1, 26.1, 23.0, 22.3, 14.4.

##### Ethyl 2-[2-(5-Benzyloxy-1*H*-indol-3-yl)ethylcarbamoyl]-4-methyl-pentanoate (**18′**)

Compound **18′** was prepared from **45b** (130 mg, 0.414 mmol, 1.0 equiv), **46ad** (125.5 mg, 0.414 mmol, 1.0 equiv), DMF (4 mL), HBTU (172.9 mg, 0.456 mmol, 1.1 equiv) and Et_3_N (170 μL, 1.243 mmol, 3.0 equiv) using general procedure G. After flash chromatography on silica gel (DCM/MeOH 100:0 to 95:5), the desired compound was obtained as a colorless oil (169 mg, 93%). LC tr = 3.18 min, MS (ESI+) *m*/*z*: 437 [M + H]^+^. ^1^H NMR (300 MHz, CDCl_3_): δ (ppm) 8.63 (br s, 1H), 7.47–7.20 (m, 4H), 7.12 (d, *J* = 2.3 Hz, 1H), 6.94–6.88 (m, 2H), 6.60 (t, *J* = 5.6 Hz, 1H), 5.07 (s, 2H), 4.21–3.95 (m, 2H), 3.55 (q, *J* = 6.5 Hz, 2H), 3.26 (t, *J* = 7.6 Hz, 1H), 2.90 (t, *J* = 6.9 Hz, 2H), 1.75–1.69 (m, 2H), 1.20–1.15 (m, 3H), 1.51 (sept, *J* = 6.5 Hz, 1H), 0.86 (dd, *J* = 2.5, 6.5 Hz, 6H). ^13^C NMR (75 MHz, CDCl_3_): δ (ppm) 172.1, 168.8, 153.0, 137.6, 131.9, 128.5, 127.8, 127.7, 127.6, 123.1, 112.8, 112.1, 112.0, 102.1, 71.0, 61.4, 51.7, 39.9, 39.7, 26.2, 25.1, 22.4, 22.1, 14.0.

##### Ethyl 2-[2-(6-Benzyloxy-1*H*-indol-3-yl)ethylcarbamoyl]-4-methyl-pentanoate (**19′**)

Compound **19′** was prepared from **45b** (85 mg, 0.45 mmol, 1.0 equiv), HBTU (188 mg, 0.50 mmol, 1.1 equiv), Et_3_N (185 μL, 1.36 mmol, 3.0 equiv), DMF (4 mL), 2-methylindole-4-*N*-benzylpiperazine (120 mg, 0.45 mmol, 1.0 equiv) using general procedure G. After flash chromatography on silica gel (cyclohexane/EtOAc 9:1 to 5:5), the desired compound was obtained as a green-yellow wax (159 mg, 81%). LC tr = 3.03 min, MS (ESI+) *m*/*z*: 437 [M + H]^+^. ^1^H NMR (300 MHz, CDCl_3_): δ (ppm) 8.42 (d, br, *J* = 1.7 Hz, 1H), 7.45–7.40 (m, 3H), 7.38–7.26 (m, 3H), 6.88–6.83 (m, 3H), 5.66 (br t, *J* = 5.7 Hz, 1H), 5.03 (s, 2H), 4.21–4.01 (m, 2H), 3.55 (ddd, *J* = 12.7, 6.8,1.3 Hz, 2H), 3.25 (dd, *J* = 8.0, 7.3 Hz, 2H), 2.90 (t, *J* = 6.9 Hz, 2H), 1.75–1.69 (m, 1H), 1.51 (sept, *J* = 6.6 Hz, 1H), 1.18 (t, *J* = 7.1 Hz, 3H), 0.87 (dd, *J* = 6.6, 2.0 Hz, 6H). ^13^C NMR (75 MHz, CDCl_3_): δ (ppm) 172.1, 168.8, 155.6, 137.4, 137.1, 128.5, 127.8, 127.5, 122.0, 121.1, 119.2, 112.4, 110.0, 96.3, 70.6, 61.4, 51.7, 40.0, 39.7, 26.2, 25.2, 22.4, 22.1, 14.0.

##### Ethyl 4-Methyl-2-[2-[5-(2-pyridylmethoxy)-1*H*-indol-3-yl]ethylcarbamoyl]pentanoate (**20′**)

Compound **20′** was prepared from **45b** (108 mg, 0.58 mmol, 1.0 equiv), **46aa** (154 mg, 0.58 mmol, 1.0 equiv), DMF (4 mL), HBTU (240 mg, 0.63 mmol, 1.1 equiv), Et_3_N (394 μL, 2.88 mmol, 5.0 equiv) using general procedure G. After flash chromatography on silica gel (DCM/MeOH 100:0 to 90:10), the desired compound was obtained as a pale-yellow solid (85 mg, 34%). LC tr = 2.60 min, MS (ESI+) *m*/*z*: 438 [M + H]^+^. ^1^H NMR (300 MHz, CDCl_3_): δ (ppm) 8.60 (m, 1H), 8.34 (s, 1H), 7.69 (td, *J* = 11.5, 1.7 Hz, 1H), 7.59 (d, *J* = 7.8 Hz, 1H), 7.26 (m, 1H), 7.21 (m, 1H), 7.12 (d, *J* = 2.3 Hz, 1H), 6.99 (d, *J* = 2.1 Hz, 1H), 6.95 (dd, *J* = 8.7, 2.4 Hz, 1H), 6.52 (t, *J* = 5.36 Hz, 1H), 5.26 (s, 2H), 4.11 (m, 2H), 3.55 (q, *J* = 7.1 Hz, 2H), 3.25 (t, *J* = 7.7 Hz, 1H), 2.90 (t, *J* = 6.9 Hz, 2H), 1.72 (m, 2H), 1.52 (m, 1H), 1.20 (t, *J* = 7.1 Hz, 3H), 0.87 (dd, *J* = 6.6, 2.3 Hz, 6H). ^13^C NMR (75 MHz, CDCl_3_): δ (ppm) 172.3, 168.6, 157.9, 152.7, 149.1, 136.8, 131.9, 127.7, 123.1, 122.5, 121.5, 112.6, 112.4, 112.0, 102.3, 71.5, 61.3, 51.8, 39.9, 39.6, 26.3, 25.2, 22.5, 22.0, 14.0.

##### Ethyl 4-Methyl-2-[2-[5-(3-pyridylmethoxy)-1*H*-indol-3-yl]ethylcarbamoyl]pentanoate (**21′**)

Compound **21′** was prepared from **45b** (92 mg, 0.490 mmol, 1.0 equiv), **46ab** (131 mg, 0.490 mmol, 1.0 equiv), DMF (4 mL), HBTU (204 mg,0.539 mmol, 1.1 equiv), Et_3_N (335 μL, 2.4 5 mmol, 5.0 equiv) using general procedure G. After flash chromatography on silica gel (DCM/MeOH 99:1 to 95:5), the desired compound was obtained as a pale-yellow solid (89 mg, 42%). LC tr = 2.51 min, MS (ESI+) *m*/*z*: 438 [M + H]^+^.

##### Ethyl4-Methyl-2-[2-[5-(4-pyridylmethoxy)-1*H*-indol-3-yl]ethylcarbamoyl]pentanoate (**22′**)

Compound **22′** was prepared from **45b** (58 mg, 0.31 mmol, 1.0 equiv), HBTU (140 mg, 0.37 mmol, 1.2 equiv), Et_3_N (130 μL, 0.92 mmol, 3.0 equiv), DMF (4 mL), **46ac** (105 mg, 0.31 mmol, 1.0 equiv) using general procedure G. After flash chromatography on silica gel (DCM/MeOH 100:0 to 90:10), the product was obtained as a yellow-brown wax (78 mg, 58%). LC tr = 2.47 min, MS (ESI+) *m*/*z*: 438 [M + H]^+^. ^1^H NMR (300 MHz, CDCl_3_): δ (ppm) 8.72 (br s, 1H), 8.59 (br d, *J* = 4.9 Hz, 2H), 7.43 (br d, *J* = 5.9 Hz, 2H), 7.27 (d, *J* = 8.8 Hz, 1H), 7.10 (d, *J* = 2.4 Hz, 1H), 7.01 (d, *J* = 2.2 Hz, 1H), 6.90 (dd, *J* = 8.8, 2.4 Hz, 1H), 6.67 (br t, *J* = 5.6 Hz, 1H), 5.43 (br s, 1H), 5.13 (s, 2H), 4.16–4.05 (m, 2H), 3.60–3.53 (m, 2H), 3.28 (dd, *J* = 8.2, 7.1 Hz, 1H), 2.92 (br t, *J* = 6.9 Hz, 2H), 1.80–1.65 (m, 2H), 1.52 (sept, *J* = 6.6 Hz, 1H), 1.20 (t, *J* = 7.1 Hz, 3H), 0.87 (dd, *J* = 6.6, 2.0 Hz, 6H). ^13^C NMR (75 MHz, CDCl_3_): δ (ppm) 172.3, 168.8, 152.4, 149.1, 147.8, 132.1, 127.7, 123.3, 121.9, 112.5, 112.3, 112.2, 102.2, 69.0, 61.4, 51.7, 40.0, 39.9, 26.3, 25.2, 22.4, 22.0, 14.0.

##### Methyl 2-[2-(5-Benzyloxy-1*H*-indol-3-yl)ethylcarbamoyl]-5-methyl-hexanoate (**23′**)

Compound **23′** was prepared from **45c** (100 mg, 0.53 mmol, 1.0 equiv), EDCI (122 mg, 0.64 mmol, 1.2 equiv), Et_3_N (218 μL, 1.61 mmol, 3.0 equiv), DMF (4 mL), **46ad** (243 mg, 0.64 mmol, 1.2 equiv) using general procedure G. After flash chromatography on silica gel (cyclohexane/EtOAc 9:1 to 5:5), the product was obtained as a green-yellow wax (178 mg, 77%). LC tr = 3.02 min, MS (ESI+) *m*/*z*: 437 [M + H]^+^. ^1^H NMR (300 MHz, CDCl_3_): δ (ppm) 7.49–7.45 (m, 2H), 7.40–7.27 (m, 3H), 7.23 (dd, *J* = 8.7, 0.5 Hz, 1H), 7.12 (d, *J* = 2.4 Hz, 1H), 6.96 (d, *J* = 2.3 Hz, 1H), 6.92 (dd, *J* = 8.7, 2.3 Hz, 1H), 6.54 (br t, *J* = 5.5 Hz, 1H), 5.09 (s, 2H), 3.63 (s, 3H), 3.57 (br dd, *J* = 12.7, 6.8 Hz, 2H), 3.12 (t, *J* = 7.4 Hz, 1H), 2.92 (br t, *J* = 6.8 Hz, 2H), 1.84 (dd, *J* = 16.4, 7.5 Hz, 1H), 1.82 (dd, *J* = 11.9, 6.1 Hz, 1H), 1.50 (sept, *J* = 6.7 Hz, 1H), 1.21–1.03 (m, 2H), 0.84 (d, *J* = 6.7 Hz, 6H). ^13^C NMR (75 MHz, CDCl_3_): δ (ppm) 172.5, 168.4, 153.2, 137.6, 131.8, 128.5, 127.8, 127.4, 123.0, 112.9, 112.3, 112.0, 102.1, 71.0, 53.4, 52.3, 39.8, 36.3, 28.9, 27.8, 25.2, 22.4, 22.3.

##### Ethyl 2-[2-[5-[(3-Fluorophenyl)methoxy]-1*H*-indol-3-yl]ethylcarbamoyl]-5-methyl-hexanoate (**24′**)

Compound **24′** was prepared from **45c** (125 mg, 0.62 mmol, 1.2 equiv), HOBt (83 mg, 0.62 mmol, 1.2 equiv), DMF (2 mL), Et_3_N (211 μL, 1.54 mmol, 3.0 equiv), EDCI (118 mg, 0.62 mmol, 1.2 equiv), **46ae** (165 mg, 0.51 mmol, 1.0 equiv), using general procedure G. After flash chromatography on silica gel (DCM/MeOH 98:2), the desired product was obtained as a brownish wax (212 mg, 88%). LC tr = 3.17 min, MS (ESI+) *m*/*z*: 469 [M + H]^+^. ^1^H NMR (300 MHz, CDCl_3_): δ (ppm) 8.42 (br s, 1H), 7.32 (td, *J* = 8.0, 5.8 Hz, 1H), 7.25–7.17 (m, 3H), 7.11 (d, *J* = 2.3 Hz, 1H), 7.01–6.95 (m, 2H), 6.90 (dd, *J* = 8.8, 2.3 Hz, 1H), 6.64 (br t, *J* = 5.7 Hz, 1H), 5.07 (s, 2H), 4.21–4.02 (m, 2H), 3.61–3.54 (m, 2H), 3.12 (t, *J* = 7.5 Hz, 1H), 2.92 (t, *J* = 6.9 Hz, 2H), 1.84 (dt, *J* = 8.7, 7.6 Hz, 2H), 1.50 (sept, *J* = 6.6 Hz, 1H), 1.23–1.09 (m, 2H), 1.20 (t, *J* = 7.2 Hz, 3H), 0.84 (d, *J* = 6.6 Hz, 6H). ^13^C NMR (75 MHz, CDCl_3_): δ (ppm) 172.2, 168.7, 163.0 (d, *J* = 245.7 Hz), 152.8, 140.3 (d, *J* = 7.2 Hz), 131.9, 130.0 (d, *J* = 8.4 Hz), 127.7, 123.1, 122.9 (d, *J* = 3.1 Hz), 114.6 (d, *J* = 16.2 Hz), 114.3 (d, *J* = 17.4 Hz), 112.8, 112.3, 112.1, 102.1, 70.1 (d, *J* = 1.8 Hz), 61.4, 53.4, 39.8, 36.3, 29.0, 27.8, 25.2, 22.4, 22.3, 14.0.

##### Ethyl2-[2-[5-[(4-Fluorophenyl)methoxy]-1*H*-indol-3-yl]ethylcarbamoyl]-5-methyl-hexanoate (**25′**)

Compound **25′** was prepared from **45c** (151 mg, 0.75 mmol, 1.2 equiv), DMF (3 mL), Et_3_N (205 μL, 3.12 mmol, 2.4 equiv), HOBt (101 mg, 0.75 mmol, 1.2 equiv), EDCI (143 mg, 0.75 mmol, 1.2 equiv), **46af** (200 mg, 0.62 mmol, 1.0 equiv) using general procedure G. After flash chromatography on silica gel (DCM/MeOH 100:0 to 98:2), the desired product was obtained as a brownish wax (268 mg, 92%). LC tr = 3.16 min, MS (ESI+) *m*/*z*: 469 [M + H]^+^. ^1^H NMR (300 MHz, CDCl_3_): δ (ppm) 8.78 (br s, 1H), 7.41 (dd, *J* = 8.4, 5.5 Hz, 1H), 7.23 (d, *J* = 8.7 Hz, 1H), 7.12 (d, *J* = 2.3 Hz, 1H), 7.04 (t, *J* = 8.7 Hz, 2H), 6.97 (d, *J* = 2.2 Hz, 1H), 6.88 (dd, *J* = 8.8, 2.3 Hz, 1H), 6.72 (t, *J* = 5.9 Hz, 1H), 5.02 (s, 2H), 4.18–4.02 (m, 1H), 4.10 (dd, *J* = 7.3, 4.2 Hz, 1H), 3.57 (q, *J* = 6.6 Hz, 2H), 3.13 (t, *J* = 7.5 Hz, 1H), 2.93 (t, *J* = 7.1 Hz, 2H), 1.85 (q, *J* = 8.0 Hz, 2H), 1.50 (sept, *J* = 6.6 Hz, 1H), 1.25–1.09 (m, 2H), 1.19 (t, *J* = 7.1 Hz, 3H), 0.84 (d, *J* = 6.6 Hz, 6H). ^13^C NMR (75 MHz, CDCl_3_): δ (ppm) 172.0, 168.7, 162.4, 152.9, 133.4 (d, *J* = 3.2 Hz), 131.9, 129.5 (d, *J* = 8.1 Hz), 127.6, 123.2, 115.3 (d, *J* = 21.5 Hz), 112.7, 112.15, 112.11, 102.1, 70.3, 61.3, 53.4, 39.9, 36.3, 28.8, 27.8, 25.2, 22.4, 22.3, 14.0.

##### Ethyl 5-Methyl-2-[2-[5-(2-pyridylmethoxy)-1*H*-indol-3-yl]ethylcarbamoyl]hexanoate (**26′**)

Compound **26′** was prepared from **45c** (78 mg, 0.39 mmol, 1.0 equiv), HBTU (176 mg, 0.46 mmol, 1.2 equiv), Et_3_N (160 μL, 1.16 mmol, 3.0 equiv), DMF (4 mL), **46aa** (132 mg, 0.39 mmol, 1.0 equiv) using general procedure G. After flash chromatography on silica gel (DCM/MeOH 100:0 to 98:2), the desired product was obtained as a light-brown solid (100 mg, 57%). LC tr = 2.75 min, MS (ESI+) *m*/*z*: 452 [M + H]^+^.

##### Ethyl 5-Methyl-2-[2-[5-(3-pyridylmethoxy)-1*H*-indol-3-yl]ethylcarbamoyl]hexanoate (**27′**)

Compound **27′** was prepared from **45c** (50 mg, 0.25 mmol, 1.0 equiv), HOBt (40 mg, 0.30 mmol, 1.2 equiv), EDCI (57 mg, 0.30 mmol, 1.2 equiv), Et_3_N (101 μL, 0.74 mmol, 3.0 equiv), DMF (4 mL), **46ab** (84 mg, 0.25 mmol, 1.0 equiv) using general procedure G. After flash chromatography on silica gel (cyclohexane/EtOAc 90:10 to 50:50), the product was obtained as a green-yellow wax (82, 74 mg). LC tr = 2.67 min, MS (ESI+) *m*/*z*: 452 [M + H]^+^. ^1^H NMR (300 MHz, CDCl_3_): δ (ppm) 8.70 (br d, *J* = 1.5 Hz, 1H), 8.66 (br d, *J* = 1.5 Hz, 1H), 8.56 (dd, *J* = 4.8, 1.5 Hz, 1H), 7.83 (ddd, *J* = 7.8, 2.2, 1.5 Hz, 1H), 7.32 (ddd, *J* = 7.8, 4.8, 0.3 Hz, 1H), 7.26 (dd, *J* = 8.8, 0.3 Hz, 1H), 7.14 (d, *J* = 2.4 Hz, 1H), 7.02 (d, *J* = 2.4 Hz, 1H), 6.90 (dd, *J* = 8.8, 2.4 Hz, 1H), 6.75 (br t, *J* = 5.6 Hz, 1H), 5.11 (s, 2H), 4.17–4.06 (m, 2H), 3.62–3.55 (m, 2H), 3.14 (t, *J* = 7.4 Hz, 1H), 2.94 (t, *J* = 6.9 Hz, 2H), 1.88–1.81 (m, 2H), 1.51 (sept, *J* = 6.6 Hz, 1H), 1.21 (t, *J* = 7.1 Hz, 3H), 1.18–1.09 (m, 2H), 0.84 (d, *J* = 6.6 Hz, 6H). ^13^C NMR (75 MHz, CDCl_3_): δ (ppm) 172.2, 168.7, 152.7, 149.0, 148.9, 135.6, 133.3, 132.0, 127.7, 123.6, 123.2, 112.8, 112.4, 112.2, 102.2, 68.5, 61.3, 53.4, 39.9, 36.3, 29.0, 27.8, 25.3, 22.4, 22.3, 14.1.

##### Ethyl 5-Methyl-2-[2-[5-(4-pyridylmethoxy)-1*H*-indol-3-yl]ethylcarbamoyl]hexanoate (**28′**)

Compound **28′** was prepared from **45c** (52 mg, 0.26 mmol, 1.0 equiv), HOBt (42 mg, 0.31 mmol, 1.2 equiv), EDCI (59 mg, 0.31 mmol, 1.2 equiv), Et_3_N (105 μL, 0.77 mmol, 3.0 equiv), DMF (4 mL), **46ac** (88 mg, 0.26 mmol, 1.0 equiv) using general procedure G. After flash chromatography on silica gel (cyclohexane/EtOAc 90:10 to 50:50), the product was obtained as a green-yellow wax (95 mg, 82%). LC tr = 2.60 min, MS (ESI+) *m*/*z*: 452 [M + H]^+^.

##### Ethyl 2-[2-[5-[(6-Chloro-3-pyridyl)methoxy]-1*H*-indol-3-yl]ethylcarbamoyl]-5-methyl-hexanoate (**29′**)

Compound **29′** was prepared from **45c** (54 mg, 0.27 mmol, 1.0 equiv), HBTU (122 mg, 0.32 mmol, 1.2 equiv), Et_3_N (110 μL, 0.80 mmol, 3.0 equiv), DMF (4 mL), **46ag** (100 mg, 0.27 mmol, 1.0 equiv) using general procedure G. After flash chromatography on silica gel (DCM/MeOH 100:0 to 90:10), the product was obtained as a brownish solid (128 mg, 99%). LC tr = 2.98 min, MS (ESI+) *m*/*z*: 486 [M + H]^+^.

##### Ethyl 5-Methyl-2-[2-[5-(pyrimidin-2-ylmethoxy)-1*H*-indol-3-yl]ethylcarbamoyl]hexanoate (**30′**)

Compound **30′** was prepared from **45c** (68 mg, 0.34 mmol, 1.0 equiv), **46ah** (90 mg, 0.34 mmol, 1.0 equiv), DMF (4 mL), HBTU (140 mg, 0.37 mmol, 1.1 equiv), Et_3_N (229 μL, 1.68 mmol, 5.0 equiv) using general procedure G. After flash chromatography on silica gel (DCM/MeOH 99:1 to 95:5), the desired compound was obtained as a light brown wax (35 mg, 23%). LC tr = 2.53 min, MS (ESI+) *m*/*z*: 453 [M + H]^+^. ^1^H NMR (300 MHz, CDCl_3_): δ (ppm) 8.79 (d, *J* = 4.9 Hz, 2H), 8.28 (s, 1H), 7.26 (t, *J* = 2.6 Hz, 1H), 7.23 (d, *J* = 4.9 Hz, 1H), 7.15 (d, *J* = 2.4 Hz, 1H), 7.00 (dd, *J* = 8.8, 2.4 Hz, 2H), 6.58 (t, *J* = 5.5 Hz, 1H), 5.37 (s, 2H), 4.11 (m, 2H), 3.56 (q, *J* = 7.1 Hz, 2H), 3.11 (t, *J* = 7.4 Hz, 1H), 2.90 (t, *J* = 6.8 Hz, 2H), 1.83 (m, 2H), 1.51 (m, 1H), 1.21 (t, *J* = 7.1 Hz, 3H), 0.84 (d, *J* = 6.6 Hz, 6H). ^13^C NMR (75 MHz, CDCl_3_): δ (ppm) 172.2, 168.5, 166.7, 157.4, 152.7, 131.9, 127.6, 123.0, 119.9, 112.9, 112.5, 112.0, 102.4, 71.7, 61.3, 53.5, 39.5, 36.3, 29.0, 27.8, 25.2, 22.4, 22.3, 14.1.

##### Ethyl2-[2-[5-[2-(3-Methoxyphenyl)ethoxy]-1*H*-indol-3-yl]ethylcarbamoyl]-5-methyl-hexanoate (**31′**)

Compound **31′** was prepared from **45c** (58 mg, 0.29 mmol, 1.0 equiv), HBTU (131 mg, 0.34 mmol, 1.2 equiv), Et_3_N (120 μL, 0.86 mmol, 3.0 equiv), DMF (4 mL), **46ai** (99 mg, 0.29 mmol, 1.0 equiv) using general procedure G. After flash chromatography on silica gel (DCM/MeOH 99:1 to 90:10), the product was obtained as a brownish solid (130 mg, 92%). LC tr = 3.19 min, MS (ESI+) *m*/*z*: 495 [M + H]^+^.

##### Ethyl 5-Methyl-2-[2-[5-(2-morpholinoethoxy)-1*H*-indol-3-yl]ethylcarbamoyl]hexanoate (**32′**)

Compound **32′** was prepared from **45c** (100 mg, 0.49 mmol, 1.0 equiv), HOBt (80 mg, 0.59 mmol, 1.2 equiv), EDCI (114 mg, 0.59 mmol, 1.2 equiv), Et_3_N (203 μL, 1.48 mmol, 3.0 equiv), DMF (4 mL), **46aj** (179 mg, 0.49 mmol, 1.2 equiv) using general procedure G. After flash chromatography on silica gel (cyclohexane/EtOAc 9:1 to 5:5), the product was obtained as a green-yellow wax (142 mg, 61%). LC tr = 2.32 min, MS (ESI+) *m*/*z*: 474 [M + H]^+^. ^1^H NMR (300 MHz, CDCl_3_): δ (ppm) 8.73 (br s, 1H), 7.23 (d, *J* = 8.8 Hz, 1H), 7.04 (d, *J* = 2.1 Hz, 1H), 6.98 (d, *J* = 2.1 Hz, 1H), 6.83 (dd, *J* = 8.8, 2.2 Hz, 1H), 4.19–4.06 (m, 4H), 3.76–3.73 (m, 4H), 3.61–3.54 (m, 2H), 3.12 (t, *J* = 7.4 Hz, 1H), 2.92 (t, *J* = 6.9 Hz, 2H), 2.84 (t, *J* = 5.7 Hz, 2H), 2.64–2.61 (m, 4H), 1.88–1.80 (m, 2H), 1.51 (sept, *J* = 6.6 Hz, 1H), 1.21 (t, *J* = 7.1 Hz, 3H), 1.17–1.09 (m, 2H), 0.84 (d, *J* = 6.6 Hz, 6H). ^13^C NMR (75 MHz, CDCl_3_): δ (ppm) 172.1, 168.7, 152.9, 131.8, 127.6, 123.1, 112.6, 112.10, 112.05, 101.7, 66.7, 66.3, 61.3, 57.8, 53.9, 53.4, 39.8, 36.3, 28.9, 27.8, 25.2, 22.4, 22.3, 14.0.

##### Ethyl5-Methyl-2-[2-[5-(2-morpholino-2-oxo-ethoxy)-1*H*-indol-3-yl]ethylcarbamoyl]hexanoate (**33′**)

Compound **33′** was prepared from **45c** (89 mg, 0.44 mmol, 1.2 equiv), HOBt (60 mg, 0.44 mmol, 1.2 equiv), DMF (2 mL), Et_3_N (201 μL, 1.47 mmol, 4.0 equiv), EDCI (212 mg, 1.10 mmol, 3.0 equiv), **46am** (125 mg, 0.37 mmol, 1.0 equiv) using general procedure G. After flash chromatography on silica gel (cyclohexane/EtOAc 70:30 to 20:80), the product was obtained as a colorless waxy solid (99 mg, 55%). LC tr = 2.52 min, MS (ESI+) *m*/*z*: 488 [M + H]^+^. ^1^H NMR (300 MHz, CDCl_3_): δ (ppm) 8.52 (br s, 1H), 7.25 (d, *J* = 8.8 Hz, 1H), 7.10 (d, *J* = 2.4 Hz, 1H), 6.99 (d, *J* = 2.1 Hz, 1H), 6.86 (dd, *J* = 8.8, 2.4 Hz, 1H), 6.69 (br t, *J* = 5.6 Hz, 1H), 4.17–4.06 (m, 2H), 4.73 (s, 2H), 3.70–3.60 (m, 8H), 3.60–3.52 (m, 2H), 3.12 (t, *J* = 7.4 Hz, 1H), 2.91 (br t, *J* = 7.0 Hz, 2H), 1.88–1.80 (m, 2H), 1.51 (sept, *J* = 6.6 Hz, 1H), 1.22 (t, *J* = 7.1 Hz, 3H), 1.18–1.09 (m, 2H), 0.85 (d, *J* = 6.6 Hz, 6H). ^13^C NMR (75 MHz, CDCl_3_): δ (ppm) 172.2, 168.6, 167.2, 152.0, 132.1, 127.6, 123.3, 112.4, 112.2, 102.1, 68.5, 66.8, 61.3, 53.4, 45.9, 42.4, 39.7, 36.3, 29.0, 27.8, 25.3, 22.40, 22.35, 14.1.

##### Ethyl 5-Methyl-2-[2-[5-[2-oxo-2-(prop-2-ynylamino)ethoxy]-1*H*-indol-3-yl]ethylcarbamoyl] Hexanoate (**34′**)

Compound **34′** was prepared from **45c** (75 mg, 0.37 mmol, 1.2 equiv), HOBt (50 mg, 0.37 mmol, 1.2 equiv), DMF (2 mL), Et_3_N (170 μL, 1.24 mmol, 4.0 equiv), EDCI (178 mg, 93 mmol, 3.0 equiv), **46an** (95 mg, 0.31 mmol, 1.0 equiv) using general procedure G. After flash chromatography on silica gel (cyclohexane/EtOAc 70:30 to 20:80), the product was obtained as a colorless waxy solid (71 mg, 51%). LC tr = 2.62 min, MS (ESI+) *m*/*z*: 456 [M + H]^+^. ^1^H NMR (300 MHz, CDCl_3_): δ (ppm) 8.58 (br s, 1H), 7.29 (d, *J* = 8.6 Hz, 1H), 7.06 (d, *J* = 2.2 Hz, 1H), 7.04 (br s, 1H), 6.85 (dd, *J* = 8.6, 2.2 Hz, 1H), 6.71 (br t, *J* = 5.6 Hz, 1H), 4.20–4.05 (m, 4H), 4.55 (s, 2H), 3.66–3.50 (m, 2H), 3.14 (t, *J* = 7.3 Hz, 1H), 2.92 (t, *J* = 6.9 Hz, 2H), 2.27 (br s, 1H), 1.88–1.80 (m, 2H), 1.51 (sept, *J* = 6.7 Hz, 1H), 1.22 (t, *J* = 7.2 Hz, 3H), 1.18–1.10 (m, 2H), 0.85 (d, *J* = 6.7 Hz, 6H). ^13^C NMR (75 MHz, CDCl_3_): δ (ppm) 172.2, 168.8, 168.7, 151.5, 132.3, 127.7, 123.5, 112.5, 112.3, 111.9, 102.8, 79.2, 71.8, 68.5, 61.4, 53.4, 39.8, 36.3, 29.0, 28.7, 27.8, 25.2, 22.4, 22.3, 14.1.

##### Ethyl 2-[2-[5-[2-(Benzylamino)-2-oxo-ethoxy]-1*H*-indol-3-yl]ethylcarbamoyl]-5-methyl-hexanoate (**35′**)

Compound **35′** was prepared from **45c** (71 mg, 0.35 mmol, 1.2 equiv), HBTU (133 mg, 0.35 mmol, 1.2 equiv), Et_3_N (88.6 mg, 0.88 mmol, 2.5 equiv), **46ao** (105 mg, 0.292 mmol, 1.0 equiv), DMF (3 mL) using general procedure G. After flash chromatography on silica gel (cyclohexane/EtOAc 70:30 to 30:70), the product was obtained as a colorless solid (104 mg, 70%). LC tr = 2.82 min, MS (ESI+) *m*/*z*: 508 [M + H]^+^. ^1^H NMR (300 MHz, CDCl_3_): δ (ppm) 8.26 (s, 1H), 7.30–7.25 (m, 6H), 7.06 (m, 3H), 6.84 (d, *J* = 8.7 Hz, 1H), 6.67 (t, *J* = 6.1 Hz, 1H), 4.57 (d, *J* = 5.9 Hz, 2H), 4.16–4.08 (m, 2H), 3.60–3.53 (m, 2H), 3.13 (t, *J* = 7.4 Hz, 1H), 2.96–2.89 (m, 4H), 1.87–1.79 (m, 2H), 1.55–1.43 (m, 1H), 1.21 (t, *J* = 7.4 Hz, 3H), 1.18–1.10 (m, 2H), 0.85 (d, *J* = 6.5 Hz, 6H). ^13^C NMR (75 MHz, CDCl_3_): δ (ppm) 172.3, 169.0, 168.6, 151.6, 137.9, 132.2, 128.7, 127.8, 127.7, 127.6, 123.4, 112.7, 112.2, 112.0, 102.7, 68.5, 61.3, 53.4, 43.0, 39.7, 36.3, 29.2, 27.8, 25.2, 22.4, 22.3, 14.1.

##### Ethyl 2-[2-[5-[2-[2-(4-Hydroxyphenyl)ethylamino]-2-oxo-ethoxy]-1*H*-indol-3-yl]ethylcarbamoyl]-5-methyl-hexanoate (**36′**)

Compound **36′** was prepared from **45c** (54 mg, 0.27 mmol, 1.0 equiv), HBTU (122 mg, 0.32 mmol, 1.2 equiv), Et_3_N (110 μL, 0.80 mmol, 3.0 equiv), DMF (4 mL), **46ap** (114 mg, 0.32 mmol, 1.0 equiv) using general procedure G. After flash chromatography on silica gel (DCM/MeOH 100:0 to 90:10), the product was obtained as an off white solid (57 mg, 33%). LC tr = 2.62 min, MS (ESI+) *m*/*z*: 538 [M + H]^+^.

^1^H NMR (300 MHz, CDCl_3_): δ (ppm) 8.53 (br s, 1H), 7.05–6.94 (m, 5H), 6.88–6.80 (m, 3H), 6.72 (dd, *J* = 8.8, 2.3 Hz, 1H), 4.47 (s, 2H), 4.19–4.06 (m, 2H), 3.19 (t, *J* = 7.3 Hz, 1H), 2.92 (t, *J* = 7.3 Hz, 2H), 2.72 (t, *J* = 6.4 Hz, 2H), 1.89–1.81 (m, 2H), 1.48 (sept, *J* = 6.6 Hz, 1H), 1.25–1.22 (m, 1H), 1.21 (t, *J* = 8.8 Hz, 3H), 1.17–1.08 (m, 2H), 0.82 (d, *J* = 6.6 Hz, 6H). ^13^C NMR (75 MHz, CDCl_3_): δ (ppm) 172.3, 169.5, 169.1, 155.7, 151.4, 132.5, 129.9, 129.3, 127.9, 123.8, 115.8, 112.2, 112.0, 110.4, 103.8, 68.3, 61.6, 53.1, 40.4, 40.1, 36.2, 34.6, 29.2, 27.7, 25.2, 22.4, 22.3, 14.0.

##### Ethyl 2-[[2-(1*H*-Indol-3-yl)-1-methyl-ethyl]carbamoyl]-4-methyl-pentanoate (**37′**)

Compound **36′** was prepared from **45b** (60 mg, 0.319 mmol, 1.0 equiv), Et_3_N (163 μL, 0.95 mmol, 3.0 equiv), **46o** (67 mg, 0.319 mmol, 1.0 equiv), HBTU (121 mg, 0.319 mmol, 1.0 equiv), DMF (1 mL) using general procedure G. The desired product was obtained as a colorless oil (114 mg, quant). LC tr = 2.11 min, MS (ESI+) *m*/*z*: 345 [M + H]^+^. ^1^H NMR (300 MHz, MeOD-*d*_4_): δ (ppm) 7.61–7.55 (m, 1H), 7.34–7.29 (m, 1H), 7.10–6.95 (m, 3H), 4.34–4.19 (m, 1H), 4.14–4.02 (m, 2H), 3.39–3.24 (m, 1H), 3.04–2.77 (m, 2H), 1.78–1.63 (m, 1H), 1.61–1.44 (m, 1H), 1.23–1.11 (m, 6H), 0.90 (s, 1.5H), 0.88 (s, 1.5H), 0.77 (d, *J* = 9.8 Hz, 1.5H), 0.75 (d, *J* = 9.7 Hz, 1.5H). ^13^C NMR (75 MHz, MeOD-*d*_4_): δ (ppm) 172.0, 170.8, 138.0, 129.1, 129.0, 124.0, 122.2, 120.7, 119.6, 119.5, 112.7, 112.6, 112.1, 62.2, 62.1, 52.2, 52.1, 47.5, 39.8, 39.0, 27.2, 26.8, 23.06, 22.4, 22.2, 22.12, 20.7, 20.3, 14.4.

##### Ethyl 2-[[(1*S*)-1-(Hydroxymethyl)-2-(1*H*-indol-3-yl)ethyl]carbamoyl]-4-methyl-pentanoate (**38′**)

Compound **38′** was prepared from **45b** (250 mg, 0.797 mmol, 1.0 equiv) and **46p** (181.9 mg, 0.96 mmol, 1.2 equiv), DMF (4 mL), HBTU (332.5 mg, 0.878 mmol, 1.1 equiv), Et_3_N (327 μL, 2.39 mmol, 3.0 equiv) using general procedure G. The desired compound was obtained as a colorless oil (138 mg, 48%). LC tr = 2.55 min, MS (ESI+) *m*/*z*: 361 [M + H]^+^. ^1^H NMR (300 MHz, CDCl_3_): δ (ppm) 8.67 (br s, 1H), 7.63 (t, *J* = 7.3 Hz, 1H), 7.32 (d, *J* = 7.9 Hz, 1H), 7.17–6.99 (m, 3H), 6.79 (dd, *J* = 16.2, 7.8 Hz, 1H), 4.30–4.26 (m, 1H), 4.12–4.00 (m, 2H), 3.66–3.44 (m, H), 3.25 (q, *J* = 7.3 Hz, 1H), 3.01–2.96 (m, 2H), 1.71–1.56 (m, 2H), 1.49–1.40 (m, 1H), 1.17 (t, *J* = 7.1 Hz, 3H), 0.83–0.79 (m, 6H). ^13^C NMR (75 MHz, CDCl_3_): δ (ppm) 172.0, 169.7, 169.6, 136.3, 127.6, 123.1, 123.0, 121.9, 119.4, 118.7, 111.3, 111.1, 111.0, 64.2, 61.6, 61.5, 52.4, 52.3, 51.7, 51.6, 39.4, 39.2, 26.4, 26.2, 22.3, 22.2, 22.0, 13.9.

##### Ethyl 2-[[(1*R*)-1-(Hydroxymethyl)-2-(1*H*-indol-3-yl)ethyl]carbamoyl]-4-methyl-pentanoate (**39′**)

Compound **39′** was prepared from **45b** (250 mg, 0.79 mmol, 1.0 equiv), **46q** (181.9 mg, 0.956 mmol, 1.2 equiv), DMF (4 mL), HBTU (332.5 mg, 0.877 mmol, 1.1 equiv), Et_3_N (327 μL, 2.391 mmol, 3.0 equiv) using general procedure G. After flash chromatography on silica gel (cyclohexane/EtOAc 90:10 to 50:50), the desired compound was obtained as a colorless oil. LC tr = 2.55 min, MS (ESI+) *m*/*z*: 361 [M + H]^+^. ^1^H NMR (300 MHz, CDCl_3_): δ (ppm) 8.67 (br s, 1H), 7.63 (t, *J* = 7.3 Hz, 1H), 7.33–7.00 (m, 3H), 6.79 (dd, *J* = 16.8, 7.8 Hz, 1H), 4.29 (br s, 1H), 4.12–4.03 (m, 2H), 3.62–3.46 (m, 3H), 3.25 (q, *J* = 7.2 Hz, 1H), 2.97 (t, *J* = 6.1 Hz, 2H), 1.71–1.54 (m, 2H), 1.49–1.39 (m, 1H), 1.19–1.13 (m, 3H), 0.82–0.80 (m, 6H). ^13^C NMR (75 MHz, CDCl_3_): δ (ppm) 172.0, 169.8, 169.6, 136.3, 127.6, 123.2, 123.1, 121.9, 119.3, 118.7, 118.6, 111.3, 111.1, 110.9, 64.2, 61.6, 61.5, 52.4, 52.3, 51.7, 51.6, 39.4, 39.2, 26.4, 26.2, 22.3, 22.2, 22.0, 13.9.

##### Ethyl 2-[[(1*S*)-2-Amino-1-(1*H*-indol-3-ylmethyl)-2-oxo-ethyl]carbamoyl]-4-methyl-pentanoate (**40′**)

Compound **40′** was prepared from **45b** (1.5 g, 7.97 mmol, 1.0 equiv), DMF (50 mL), Et_3_N (10 mL), HBTU (5.29 g, 13.9 mmol, 1.75 equiv), **46r** (1.78 g, 8.77 mmol, 1.1 equiv) using general procedure G. After reverse flash chromatography on C18 silica gel (H_2_O/ACN,10:90 to 100:0), the desired product was obtained as white powder (1.2 g, 40.3%). LC tr = 1.84 min, MS (ESI+) *m*/*z*: 374 [M + H]^+^. ^1^H NMR (300 MHz, DMSO-*d*_6_): δ (ppm) 10.87–10.78 (m, 1H), 8.36 (d, *J* = 8.4 Hz, 0.5H), 8.24 (d, *J* = 8.1 Hz, 0.5H), 7.59 (d, *J* = 7.8 Hz, 1H), 7.41–7.26 (m, 2H), 7.19–6.91 (m, 4H), 4.53 (dtd, *J* = 11.9, 6.5, 3.7 Hz, 1H), 4.14–3.91 (m, 2H), 3.43 (ddd, *J* = 14.9, 8.3, 6.6 Hz, 1H), 3.14 (td, *J* = 14.1, 5.0 Hz, 1H), 3.06–2.81 (m, 1H), 1.69–1.30 (m, 2H), 1.19–0.95 (m, 3H), 0.84 (dd, *J* = 6.5, 5.3 Hz, 3H), 0.69 (dd, *J* = 6.5, 4.0 Hz, 3H).

##### Methyl 3-[2-(5-Benzyloxy-1*H*-indol-3-yl)ethylamino]-3-oxo-2-(tetrahydropyran-4-ylmethyl)propa-noate (**41′**)

Compound **41′** was prepared from **45g** (60 mg, 0.171 mmol, 1.0 equiv), DMF (1 mL), Et_3_N (130 μL, 0.933 mmol, 5.0 equiv), HBTU (86 mg, 0.226 mmol, 1.32 equiv), **46ad** (70 mg, 0.228 mmol, 1.32 equiv) using general procedure G. After flash chromatography on silica gel (DCM/MeOH 100:0 to 90:10), the desired product was obtained as a white solid (40 mg, 39% yield). LC tr = 2.77 min, MS (ESI+) *m*/*z*: 465 [M + H]^+^. ^1^H NMR (300 MHz, CDCl_3_): δ (ppm) 8.16 (br s, 1H), 7.48 (m, 2H), 7.38 (m, 2H), 7.31 (m, 1H), 7.25 (d, *J* = 8.8 Hz, 1H), 7.12 (d, *J* = 2.3 Hz, 1H), 6.99 (d, *J* = 2.3 Hz, 1H), 6.94 (dd, *J* = 8.8, 2.4 Hz, 1H), 6.43 (t, *J* = 5.5 Hz, 1H), 5.10 (s, 2H), 3.89 (m, 2H), 3.64 (s, 3H), 3.59 (m, 2H), 3.28 (m, 3H), 2.93 (t, *J* = 6.8 Hz, 2H), 1.76 (t, *J* = 7.0 Hz, 2H), 1.51 (m, 2H), 1.38 (m, 1H), 1.20 (m, 2H), 0.86 (m, 2H). ^13^C NMR (75 MHz, CDCl_3_): δ (ppm) 172.4, 168.1, 153.2, 137.6, 131.7, 128.5, 127.8, 127.7, 127.6, 122.9, 113.0, 112.4, 112.0, 102.2, 71.0, 67.7, 67.7, 52.5, 50.3, 39.8, 37.6, 33.0, 32.6, 32.6, 29.7, 25.1.

##### Methyl 2-(Cyclohexylmethyl)-3-oxo-3-[2-[5-(4-pyridylmethoxy)-1*H*-indol-3-yl]ethylamino]propanoate (**42′**)

Compound **42′** was prepared from **45f** (76 mg, 0.35 mmol, 1.2 equiv), HBTU (145 mg, 0.38 mmol, 1.3 equiv), Et_3_N (120 μL, 0.88 mmol, 3.0 equiv), DMF (2 mL), **46ac** (100 mg, 0.29 mmol, 1.0 equiv) using general procedure G. After flash chromatography on silica gel (DCM/MeOH 100:0 to 90:10), the product was obtained as a yellowish solid (91 mg, 67%). LC tr = 2.65 min, MS (ESI+) *m*/*z*: 464 [M + H]^+^. ^1^H NMR (300 MHz, MeOD-*d*_4_): δ (ppm) 8.45 (dd, *J* = 4.6, 1.6 Hz, 2H), 8.16 (br t, *J* = 5.7 Hz, 1H), 7.48 (dd, *J* = 4.6, 1.6 Hz, 2H), 7.25 (d, *J* = 8.7 Hz, 1H), 7.14 (d, *J* = 2.4 Hz, 1H), 7.04 (s, 1H), 6.85 (dd, *J* = 8.7, 2.4 Hz, 1H), 5.12 (s, 2H), 3.62 (s, 3H), 3.56–3.43 (m, 2H), 3.38 (t, *J* = 7.6 Hz, 1H), 2.90 (t, *J* = 7.5 Hz, 2H), 1.70–1.55 (m, 5H), 1.67 (t, *J* = 7.4 Hz, 2H), 1.17–1.05 (m, 4H), 0.90–0.77 (m, 2H). ^13^C NMR (75 MHz, MeOD-*d*_4_): δ (ppm) 172.5, 171.6, 171.5, 153.4, 150.3, 149.9, 133.7, 129.1, 124.5, 123.3, 113.1, 113.0, 112.8, 103.1, 69.7, 52.7, 51.3, 41.4, 41.3, 37.7, 36.6, 34.3, 33.7, 27.4, 27.2, 27.1, 26.0.

##### Ethyl 2-[[(1*S*)-2-Amino-1-[(5-benzyloxy-1*H*-indol-3-yl)methyl]-2-oxo-ethyl]carbamoyl]-4-methyl-pentanoate (**43′**)

Compound **43′** was prepared from **45b** (0.26 g, 1.36 mmol, 1.2 equiv), DMF (10 mL), Et_3_N (3 mL), HBTU (0.65 g, 1.70 mmol, 1.5 equiv), **46ak** (0.350 g, 1.13 mmol, 1.0 equiv) using general procedure G. After reverse phase chromatography (H_2_O/ACN, 90:10 to 0:100), the desired product was obtained as a yellow oil. (100 mg, 18.4%). LC tr = 2.10 min, MS (ESI+) *m*/*z*: 480 [M + H]^+^, MS (ESI−) *m*/*z*: 478 [M − H]^−^. ^1^H NMR (300 MHz, DMSO-*d*_6_): δ (ppm) 8.40 (d, *J* = 8.5 Hz, 0.5H), 8.20 (d, *J* = 8.5 Hz, 0.3H), 7.54–7.03 (m, 14.3H), 6.79 (ddd, *J* = 8.9, 4.1, 2.4 Hz, 1H), 5.31 (s, 2H), 5.10 (s, 2H), 4.53 (qd, *J* = 8.7, 5.0 Hz, 1H), 4.17–3.85 (m, 2H), 3.51–3.35 (m, 1H), 3.10 (m, 1H), 3.02–2.81 (m, 1H), 1.57 (tt, *J* = 8.5, 4.6 Hz, 1H), 1.50–1.30 (m, 2H), 1.23–0.96 (m, 3H), 0.93–0.73 (m, 3H), 0.64 (dd, *J* = 6.6, 2.6 Hz, 3H).

##### Ethyl 2-[[(1*S*)-2-Amino-2-oxo-1-[[5-(4-pyridylmethoxy)-1*H*-indol-3-yl]methyl]ethyl]carbamoyl]-4-methyl-pentanoate (**44′**)

Compound **43′** was prepared from **45b** (0.32 g, 1.36 mmol, 1.5 equiv), DMF (10 mL), Et_3_N (3 mL), HBTU (0.37 g, 0.97 mmol, 1.5 equiv), **46al** (0.350 g, 1.13 mmol, 1 equiv) using general procedure G. The reaction mixture was stirred for 4 h under Ar atmosphere. After reverse phase chromatography (H_2_O/ACN, 90:10 to 0:100), the desired product was obtained as a yellow oil (150 mg, 48.4%). LC tr = 1.77 min, MS (ESI+) *m*/*z*: 481 [M + H]^+^. ^1^H NMR (300 MHz, DMSO-*d*_6_): δ (ppm) 10.71 (dd, *J* = 7.4, 2.4 Hz, 1H), 8.59 (dt, *J* = 4.6, 1.0 Hz, 2H), 8.35 (d, *J* = 8.5 Hz, 0.5H), 8.24 (d, *J* = 8.1 Hz, 0.5H), 7.49 (d, *J* = 6.1 Hz, 2H), 7.40 (s, 0.5H), 7.32 (s, 0.5H), 7.28–7.18 (m, 2H), 7.16 (s, 0.5H), 7.12–7.02 (m, 1.5H), 6.81 (dt, *J* = 8.8, 2.6 Hz, 1H), 5.18 (s, 2H), 4.51 (tt, *J* = 8.6, 4.8 Hz, 1H), 4.01 (m, 2H), 3.42 (dt, *J* = 16.9, 7.4 Hz, 1H), 3.08 (td, *J* = 14.0, 4.8 Hz, 1H), 3.00–2.79 (m, 1H), 1.72–1.32 (m, 3H), 1.21–0.94 (m, 5H), 0.91–0.76 (m, 4H), 0.67 (dd, *J* = 6.6, 3.4 Hz, 3H).

##### 2-Methoxycarbonyl-4-methyl-pentanoic Acid (**45a**)

Compound **45a** was prepared from **47a** (988 μL, 5.31 mmol, 1.0 equiv), KOH (311 mg, 5.54 mmol, 1.04 equiv), water (2 mL), MeOH (8 mL) using general procedure C. The desired compound was obtained as a colorless clear liquid (920 mg, quant.). LC tr = 2.18 min, MS (ESI+) *m*/*z*: 175 [M + H]^+^. ^1^H NMR (300 MHz, CDCl_3_): δ (ppm) 9.48 (br s, 1H), 3.76 (s, 3H), 3.49 (t, *J* = 7.6 Hz, 1H), 1.85–1.77 (m, 2H), 1.60 (sept, *J* = 6.7 Hz, 1H), 0.93 (d, *J* = 6.6 Hz, 6H). ^13^C NMR (75 MHz, CDCl_3_): δ (ppm) 175.3, 170.1, 52.7, 50.0, 37.6, 26.1, 22.2.

##### 2-Ethoxycarbonyl-4-methyl-pentanoic Acid (**45b**)

Compound **45b** was prepared from **47b** (556 mg, 2.49 mmol, 1 equiv), NaOH (105 mg, 2.49 mmol, 1 equiv), EtOH (15 mL) using general procedure C to afford compound **45b** (460 mg, 93.1%). LC tr = 3.77 min, MS (ESI+) *m*/*z*: 341 [M + H]^+^. ^1^H NMR (300 MHz, CDCl_3_): δ (ppm) 7.27–6.96 (m, 4H), 4.14 (q, *J* = 7.13 Hz, 4H), 1.90–1.75 (m, 3H), 1.22 (t, *J* = 7.14 Hz, 6H), 0.90 (d, *J* = 6.2 Hz, 6H). ^13^C NMR (75 MHz, CDCl_3_): δ (ppm) 171.4, 138.6, 133.9, 130.2, 129.4, 128.2, 127.0, 61.2, 58.2, 40.9, 38.4, 24.1, 23.6, 13.9.

##### 2-Methoxycarbonyl-5-methyl-hexanoic Acid (**45c**)

Compound **45c** was prepared from 5-isopentyl-2,2-dimethyl-1,3-dioxane-4,6-dione (442.3 mg, 2.064 mmol, 1.0 equiv), MeOH (4 mL), using general procedure B to afford compound **45c** as a yellowish oil (362 mg, 88%). LC tr = 2.43 min, MS (ESI+) *m*/*z*: 189 [M + H]^+^. ^1^H NMR (300 MHz, CDCl_3_): δ (ppm) 10.56 (br s, 1H), 3.77 (s, 3H), 3.36 (t, *J* = 7.5 Hz, 1H), 1.97–1.89 (m, 2H), 1.57 (sept, *J* = 6.6 Hz, 1H), 1.27–1.19 (m, 2H), 0.89 (d, *J* = 6.6 Hz, 6H). ^13^C NMR (75 MHz, CDCl_3_): δ (ppm) 175.2, 169.9, 52.6, 51.8, 36.3, 27.8, 26.9, 22.3.

##### 2-Methoxycarbonyl-5,5-dimethyl-hexanoic Acid (**45d**)

Compound **45d** was prepared from compound **49b** (190.6 mg, 0.835 mmol, 1.0 equiv), MeOH (4 mL), using general procedure B to afford compound **45d** as a colorless oil (161 mg, 95%). LC tr = 2.50 min, MS (ESI+) *m*/*z*: 203 [M + H]^+^. ^1^H NMR (300 MHz, CDCl_3_): δ (ppm) 10.85 (br s, 1H), 3.77 (s, 3H), 3.33 (t, *J* = 7.44 Hz, 1H), 1.95–1.86 (m, 2H), 1.25–1.18 (m, 2H), 0.90 (s, 9H). ^13^C NMR (75 MHz, CDCl_3_): δ (ppm) 175.4, 169.8, 52.6, 52.3, 41.4, 30.3, 29.1, 24.3.

##### 2-Methoxycarbonyl-4-phenyl-butanoic Acid (**45e**)

Compound **45e** was prepared from compound **49c** (680 mg, 2.74 mmol, 1.0 equiv), MeOH (15 mL), using general procedure B to afford compound **45e** as a pale-yellow oil (612 mg, 98%). LC tr = 2.20 min, MS (ESI+) *m*/*z*: 221 [M + H]^+^. ^1^H NMR (300 MHz, CDCl_3_): δ (ppm) 7.32–7.17 (m, 5H), 3.76 (s, 3H), 3.42 (t, *J* = 7.4 Hz, 1H), 2.69 (dd, *J* = 8.0, 7.4 Hz, 2H), 2.29–2.22 (m, 2H). ^13^C NMR (75 MHz, CDCl_3_): δ (ppm) 174.7, 169.6, 140.3, 128.5, 126.3, 52.7, 50.7, 33.2, 30.4.

##### 2-(Cyclohexylmethyl)-3-methoxy-3-oxo-propanoic Acid (**45f**)

Compound **45f** was prepared from compound **49d** (352.8 mg, 1.468 mmol, 1.0 equiv), MeOH (4 mL), using general procedure B to afford compound **45f** as a colorless oil (288 mg, 92%). LC tr = 2.52 min, MS (ESI+) *m*/*z*: 215 [M + H]^+^.

##### 3-Methoxy-3-oxo-2-(tetrahydropyran-4-ylmethyl)propanoic Acid (**45g**)

Compound **45g** was prepared from compound **49e** (1.01 g, 4.15 mmol, 1.0 equiv), MeOH (10 mL), using general procedure B to afford compound **45g** as a white solid (76 mg, 96% yield). LC tr = 1.42 min, MS (ESI+) *m*/*z*: 217 [M + H]^+^. ^1^H NMR (300 MHz, MeOD-*d*_4_): δ (ppm) 9.50 (br s, 1H), 3.77 (s, 3H), 3.39 (td, *J* = 11.7, 1.7 Hz, 2H), 3.99 (dd, *J* = 11.3, 4.0 Hz, 2H), 1.89 (m, 2H), 1.63 (m, 2H), 1.54 (m, 1H), 1.33 (td, *J* = 12.0, 4.5 Hz, 2H). ^13^C NMR (75 MHz, MeOD-*d*_4_): δ (ppm) 173.5, 169.8, 67.6, 52.7, 48.6, 35.5, 35.4, 32.7, 32.4.

##### 2-[5-(2-Pyridylmethoxy)-1*H*-indol-3-yl]ethanamine;dihydrochloride (**46aa**)

Compound **46aa** was prepared from **52aa** (167 mg, 0.45 mmol, 1.0 equiv), MeOH (5 mL), 4 N HCl in dioxane (2.0 mL) using general procedure F. The desired product was obtained as a yellow solid (154 mg, quant.). LC tr = 1.84 min, MS (ESI+) *m*/*z*: 268 [M + H]^+^.

##### 2-[5-(3-Pyridylmethoxy)-1*H*-indol-3-yl]ethanamine;dihydrochloride (**46ab**)

Compound **46ab** was prepared from **52ab** (142 mg, 0.39 mmol, 1.0 equiv), MeOH (5 mL), 4 N HCl in dioxane (2.0 mL) using general procedure F. The desired product was obtained as a beige solid (131 mg, quant.). LC tr = 2.34 min, MS (ESI+) *m*/*z*: 268 [M + H]^+^.

##### 2-[5-(4-Pyridylmethoxy)-1*H*-indol-3-yl]ethanamine;dihydrochloride (**46ac**)

Compound **46ac** was prepared from **52ac** (230 mg, 0.63 mmol, 1.0 equiv), MeOH (5 mL), 4 N HCl in dioxane (2.0 mL) using general procedure F. The desired product was obtained as a brown solid (211 mg, 99%). LC tr = 1.57 min, MS (ESI+) *m*/*z*: 268 [M + H]^+^. ^1^H NMR (300 MHz, MeOD-*d*_4_): δ (ppm) 7.33 (d, *J* = 8.8 Hz, 1H), 7.29 (d, *J* = 2.4 Hz, 1H), 7.20 (s, 1H), 6.97 (dd, *J* = 8.8, 2.4 Hz, 1H), 5.51 (s, 2H), 3.25–3.20 (m, 2H), 3.14–3.08 (m, 2H). ^13^C NMR (75 MHz, MeOD-*d*_4_): δ (ppm) 162.0, 153.1, 142.4, 128.6, 125.8, 125.6, 113.6, 113.3, 110.4, 103.1, 69.4, 41.2, 24.4.

##### 2-(5-Benzyloxy-1*H*-indol-3-yl)ethanamine;2,2,2-trifluoroacetic Acid (**46ad**)

Compound **46ad** was prepared from **52ad** (250 mg, 0.68 mmol, 1.0 equiv), DCM (10 mL), TFA (1 mL) using general procedure F. After flash chromatography on silica gel (DCM/MeOH, 100:0 to 90:10), the desired product was obtained (255 mg, 98%). LC tr = 2.20 min, MS (ESI+) *m*/*z*: 267 [M + H]^+^.

##### 2-[5-[(3-Fluorophenyl)methoxy]-1*H*-indol-3-yl]ethanamine;hydrochloride (**46ae**)

Compound **46ae** was prepared from **52ae** (275 mg, 0.72 mmol, 1.0 equiv), MeOH (8 mL), HCl 4 M in dioxane (2 mL) using general procedure F. The residue was triturated in DCM and filtered, affording the desired product as an off-white solid (166 mg, 72%). LC tr = 2.13 min, MS (ESI+) *m*/*z*: 285 [M + H]^+^.

##### 2-[5-[(4-Fluorophenyl)methoxy]-1*H*-indol-3-yl]ethanamine;hydrochloride (**46af**)

Compound **46af** was prepared from **52af** (250 mg, 0.65 mmol, 1.0 equiv), MeOH (8 mL), HCl 4 M in dioxane (2 mL) using general procedure F. The residue was triturated in DCM and filtered, affording the desired product as a beige solid (200 mg, 96%). LC tr = 2.12 min, MS (ESI+) *m*/*z*: 285 [M + Na]^+^.

^1^H NMR (300 MHz, MeOD-*d*_4_): δ (ppm) 7.51–7.45 (m, 2H), 7.29 (dd, *J* = 8.8, 0.4 Hz, 1H), 7.16 (br s, 1H), 7.16 (d, *J* = 2.3 Hz, 1H), 7.13–7.05 (m, 2H), 6.87 (dd, *J* = 8.8, 2.3 Hz, 1H), 5.08 (s, 2H), 3.23–3.18 (m, 2H), 3.11–3.05 (m, 2H). ^13^C NMR (75 MHz, MeOD-*d*_4_): δ (ppm) 163.8, 154.1, 135.4, 133.8, 130.7, 128.5, 125.2, 116.1, 113.7, 113.3, 110.0, 103.0, 71.4, 41.1, 24.5.

##### 2-[5-[(6-Chloro-3-pyridyl)methoxy]-1*H*-indol-3-yl]ethanamine;dihydrochloride (**46ag**)

Compound **46ag** was prepared from **52ag** (200 mg, 0.50 mmol, 1.0 equiv), MeOH (5 mL), 4 N HCl in dioxane (2.0 mL) using general procedure F. After flash chromatography on silica gel (DCM/MeOH, 95:5 to 80:20), the desired product was obtained as an off-white powder (100 mg, 54%). LC tr = 2.03 min, MS (ESI+) *m*/*z*: 302 [M + H]^+^. ^1^H NMR (300 MHz, MeOD-*d*_4_): δ (ppm) 8.45 (dd, *J* = 2.4, 0.5 Hz, 1H), 7.93 (ddt, *J* = 8.2, 2.4, 0.5 Hz, 1H), 7.46 (dd, *J* = 8.2, 0.5 Hz, 1H), 7.29 (dd, *J* = 8.8, 0.4 Hz, 1H), 7.20 (d, *J* = 2.4 Hz, 1H), 7.17 (s, 1H), 6.88 (dd, *J* = 8.8, 2.4 Hz, 1H), 5.16 (s, 2H), 3.25–3.19 (m, 2H), 3.12–3.07 (m, 2H). ^13^C NMR (75 MHz, MeOD-*d*_4_): δ (ppm) 153.7, 151.4, 149.7, 140.3, 134.8, 133.9, 128.5, 125.8, 125.6, 113.6, 113.4, 110.4, 103.1, 69.4, 41.2, 24.4.

##### 2-[5-(Pyrimidin-2-ylmethoxy)-1*H*-indol-3-yl]ethanamine;hydrochloride (**46ah**)

Compound **46ah** was prepared from **52ah** (98 mg, 0.27 mmol, 1.0 equiv), MeOH (5 mL), 4 N HCl in dioxane (2.0 mL) using general procedure F. The residue was triturated in DCM and the desired product was filtered off and obtained as a yellow solid (91 mg, quant.). LC tr = 1.52 min, MS (ESI+) *m*/*z*: 269 [M + H]^+^.

##### 2-[5-[2-(3-Methoxyphenyl)ethoxy]-1*H*-indol-3-yl]ethanamine;hydrochloride (**46ai**)

Compound **46ai** was prepared from **52ai** (140 mg, 0.34 mmol, 1.0 equiv), MeOH (5 mL), 4 N HCl in dioxane (2.0 mL) using general procedure F. The residue was triturated in DCM and the desired product was filtered off and obtained as a brown solid (99 mg, 84%). LC tr = 2.32 min, MS (ESI+) *m*/*z*: 311 [M + H]^+^. ^1^H NMR (300 MHz, MeOD-*d*_4_): δ (ppm) 7.27 (dd, *J* = 8.8, 0.4 Hz, 1H), 7.17 (d, *J* = 8.1 Hz, 1H), 7.15 (br s, 1H), 7.07 (br d, *J* = 2.4 Hz, 1H), 6.88–6.86 (m, 2H), 6.79 (dd, *J* = 8.9, 2.4 Hz, 1H), 6.75 (ddd, *J* = 8.2, 2.4, 1.1 Hz, 1H), 4.20 (t, *J* = 6.9 Hz, 2H), 3.74 (s, 3H), 3.22–3.17 (m, 2H), 3.10–3.00 (m, 4H). ^13^C NMR (75 MHz, MeOD-*d*_4_): δ (ppm) 161.1, 154.3, 141.7, 133.6, 130.3, 128.5, 125.1, 122.3, 115.7, 113.5, 113.2, 112.6, 110.0, 102.4, 70.8, 55.6, 37.0, 24.4.

##### 2-[5-(2-Morpholinoethoxy)-1*H*-indol-3-yl]ethanamine;dihydrochloride (**46aj**)

Compound **46aj** was prepared from **52aj** (195 mg, 0.50 mmol, 1.0 equiv), DCM (10 mL), 4 N HCl in dioxane (3 mL) using general procedure F. The desired product was obtained as a green solid (180 mg, 99%). LC tr = 1.42 min, MS (ESI+) *m*/*z*: 290 [M + H]^+^. ^1^H NMR (300 MHz, MeOD-*d*_4_): δ (ppm) 8.00 (br s, 1H), 7.36 (d, *J* = 8.7 Hz, 1H), 7.31 (br s, 1H), 7.22 (s, 1H), 6.90 (br d, *J* = 8.7 Hz, 1H), 4.45 (br s, 2H), 4.04–3.88 (m, 4H), 3.60–3.56 (m, 4H), 3.26–3.13 (m, 6H). ^13^C NMR (75 MHz, MeOD-*d*_4_): δ (ppm) 153.0, 133.8, 128.5, 125.5, 113.5, 113.3, 110.4, 103.0, 64.7, 63.9, 57.5, 53.6, 41.3, 41.2, 24.3, 24.2.

##### *N*-[(1*S*)-2-Amino-1-[(5-benzyloxy-1*H*-indol-3-yl)methyl]-2-oxo-ethyl]-2-(hydroxycarbamoyl)-4-methyl-pentanamide (**46ak**)

To the solution of **52ak** (400 mg, 0.94 mmol, 1.0 equiv) in DMF (5 mL) was added ammonia (7 M in methanol, 20 mL) and the reaction mixture was stirred at 60 °C for 96 h. The reaction mixture was concentrated under reduced pressure to give the corresponding primary amide. This intermediate was then dissolved in 20 mL 4 M HCl in dioxane. The reaction mixture was stirred at rt for 8 h and concentrated under reduced pressure to give compound **46ak** as yellow oil (300 mg, 100%). LC tr = 1.62 min, MS (ESI+) *m*/*z*: 310 [M + H]^+^. ^1^H NMR (300 MHz, DMSO-*d*_6_): δ (ppm) 10.88 (d, *J* = 2.5 Hz, 1H), 8.04 (s, 3H), 7.53–7.14 (m, 9H), 6.83 (dd, *J* = 8.8, 2.4 Hz, 1H), 5.11 (s, 2H), 3.92 (s, 1H), 3.77–3.63 (m, 1H), 3.48 (dt, *J* = 8.6, 4.1 Hz, 1H), 3.23 (dd, *J* = 14.7, 5.4 Hz, 2H), 3.08 (dd, *J* = 14.8, 8.0 Hz, 1H).

##### (2*S*)-2-Amino-3-[5-(4-pyridylmethoxy)-1*H*-indol-3-yl]propenamide (**46al**)

To the solution of compound **52al** (640 mg, 1.5 mmol, 1.0 equiv) in DMF (5 mL) was added ammonia (7 M in methanol, 10 mL) and the reaction mixture was stirred at 50 °C for 4 days. Then the mixture was concentrated under reduced pressure to give the corresponding primary amide. This intermediate was dissolved in 10 mL of DMF and 10 mL of 4 M HCl in dioxane. The reaction mixture was stirred at rt for 5 days and concentrated under reduced pressure to give the desired compound **46al** (2.5 g, 84.3%). LC tr = 2.31 min, MS (ESI+) *m*/*z*: 425 [M + H]^+^. ^1^H NMR (300 MHz, DMSO-*d*_6_): δ (ppm) 7.47–7.44 (d, *J* = 6.57 Hz, 2H), 7.38–7.22 (m, 4H), 7.15 (s, 1H), 7.04 (s, 1H), 6.87–6.84 (d, *J* = 8.32 Hz, 1H), 5.07 (s, 2H), 4.83–4.44 (t, *J* = 6.0 Hz, 1H), 3.24–3.18 (dd, *J* = 5.7, 14.5 Hz,1H), 3.12–3.05 (dd, *J* = 5.7, 14.5 Hz, 1H), 1.39 (s, 9H).

##### 2-[[3-(2-Aminoethyl)-1*H*-indol-5-yl]oxy]-1-morpholino-ethanone;hydrochloride (**46am**)

Compound **46am** was prepared from **54am** (161 mg, 0.40 mmol, 1.0 equiv), MeOH (3 mL), 4 N HCl in dioxane (1.5 mL) using general procedure F. After reverse phase chromatography (H_2_O/ACN, 90:10 to 0:100), the desired product was obtained (125 mg, 92%). LC tr = 1.43 min, MS (ESI+) *m*/*z*: 304 [M + H]^+^.

##### 2-[[3-(2-Aminoethyl)-1*H*-indol-5-yl]oxy]-*N*-prop-2-ynyl-acetamide;hydrochloride (**46an**)

Compound **46an** was prepared from **54an** (116 mg, 0.31 mmol, 1.0 equiv), MeOH (3 mL), 4 N HCl in dioxane (1.5 mL) using general procedure F. After reverse phase chromatography (H_2_O/ACN, 90:10 to 0:100), the desired product was obtained (96 mg, quant.). LC tr = 1.53 min, MS (ESI+) *m*/*z*: 272 [M + H]^+^.

##### 2-[[3-(2-Aminoethyl)-1*H*-indol-5-yl]oxy]-*N*-benzyl-acetamide;hydrochloride (**46ao**)

Compound **46ao** was prepared from **54ao** (124 mg, 0.293 mmol, 1.0 equiv), DCM (4 mL), 4 N HCl in dioxane (2 mL) using general procedure F. The desired product was obtained as a dark solid (105 mg, quant.). LC tr = 1.87 min, MS (ESI+) *m*/*z*: 325 [M + H]^+^. ^1^H NMR (300 MHz, CDCl_3_): δ (ppm) 7.33 (s, 1H), 7.30 (s, 1H), 7.24–7.16 (m, 7H), 6.91 (dd, *J* = 8.8 and 2.3 Hz, 1H), 4.60 (s, 2H), 4.45 (s, 2H), 3.17 (m, 2H), 3.10 (m, 2H). ^13^C NMR (75 MHz, CDCl_3_): δ (ppm) 171.8, 153.1, 139.7, 134.0, 129.4, 128.5, 128.4, 125.5, 113.4, 113.3, 110.3, 102.9, 69.3, 43.6, 41.1, 24.4.

##### 2-[[3-(2-Aminoethyl)-1*H*-indol-5-yl]oxy]-*N*-[2-(4-hydroxyphenyl)ethyl]acetamide;hydrochloride (**46ap**)

Compound **46ap** was prepared from **54ap** (140 mg, 0.31 mmol, 1.0 equiv), MeOH (10 mL), 4 N HCl in dioxane (2 mL) using general procedure F. The desired product was obtained as a greenish waxy solid (117 mg, 97%). LC tr = 1.73 min, MS (ESI+) *m*/*z*: 354 [M + H]^+^. ^1^H NMR (300 MHz, MeOD-*d*_4_): δ (ppm) 7.31 (d, *J* = 8.8 Hz, 1H), 7.19 (s, 1H), 7.09 (d, *J* = 2.4 Hz, 1H), 6.95–7.00 (m, 2H), 6.86 (dd, *J* = 8.8, 2.4 Hz, 1H), 6.71–6.61 (m, 2H), 4.50 (s, 2H), 3.45 (dd, *J* = 7.8, 6.9 Hz, 2H), 3.24–3.19 (m, 2H), 3.11–3.07 (m, 2H), 2.73–2.68 (m, 2H). ^13^C NMR (75 MHz, MeOD-*d*_4_): δ (ppm) 171.7, 156.9, 153.2, 134.1, 131.0, 130.8, 128.5, 125.5, 116.2, 113.5, 113.3, 110.2, 102.7, 69.3, 41.9, 41.1, 35.7, 24.4.

##### 5-Isopentyl-2,2-dimethyl-1,3-dioxane-4,6-dione (**49c**)

Compound **49c** was prepared from Meldrum’s acid (1.0 g, 6.938 mmol, 1.0 equiv), 3-methylbutanal (575 μL, 6.938 mmol, 1.0 equiv), l-proline (159.77 mg, 1.388 mmol, 0.3 equiv), EtOH (10 mL), acetic acid (1.19 mL), sodium borohydride (787.5 mg, 20.82 mmol, 3.0 equiv) using general procedure A. After flash chromatography on silica gel (Cy/AcOEt, 90:10 to 50:50), the desired compound was obtained as a white solid (1.08 g, 72%). LC tr = 2.81 min, MS (ESI+) *m*/*z*: 215 [M + H]^+^.^1^H NMR (300 MHz, CDCl_3_): δ (ppm) 3.58 (t, *J* = 5.0 Hz, 1H), 2.12–2.05 (m, 2H), 1.80 (d, *J* = 0.6 Hz, 3H), 1.75 (d, *J* = 0.6 Hz, 3H), 1.66–1.53 (sep, *J* = 6.6 Hz, 1H), 1.37–1.28 (m, 2H), 0.92 (d, *J* = 5.0 Hz, 6H). ^13^C NMR (75 MHz, CDCl_3_): δ (ppm) 165.7, 104.8, 46.3, 35.3, 28.4, 28.2, 26.8, 24.6, 22.3.

##### 5-(3,3-Dimethylbutyl)-2,2-dimethyl-1,3-dioxane-4,6-dione (**49d**)

Compound **49d** was prepared from Meldrum’s acid (300 mg, 2.082 mmol, 1.0 equiv), 3,3-dimethylbutanal (261 μL, 1.388 mmol, 1.0 equiv), l-proline (47.93 mg, 0.416 mmol, 0.3 equiv), EtOH (5 mL), acetic acid (357 μL), sodium borohydride (236.25 mg, 6.245 mmol, 3.0 equiv) using general procedure A. After flash chromatography on silica gel (DCM/Cy, 70/30 to 100/0), the desired compound was obtained as a white solid (191 mg, 40%). LC tr = 2.98 min, MS (ESI+) *m*/*z*: 229 [M + H]^+^. ^1^H NMR (300 MHz, CDCl_3_): δ (ppm) 3.56 (t, *J* = 4.9 Hz, 1H), 2.13–2.05 (m, 2H), 1.79–1.76 (m, 6H), 1.34–1.28 (m, 2H), 0.92 (s, 9H). ^13^C NMR (75 MHz, CDCl_3_): δ (ppm) 165.6, 104.7, 46.4, 40.0, 30.3, 29.1, 28.4, 26.9, 21.9.

##### 2,2-Dimethyl-5-(2-phenylethyl)-1,3-dioxane-4,6-dione (**49e**)

Phenylacetaldehyde (463 mg, 3.47 mmol, 1.0 equiv) was dissolved in ACN (3.5 mL) and added to a solution of Meldrum’s acid (500 mg, 3.47 mmol, 1.0 equiv) in ACN (3.5 mL) and stirred at rt for 1 h. A suspension of Hantzsch’ s ester (879 mg, 3.47 mmol, 1.0 equiv) in EtOH (7 mL) was added, followed by l-proline (80 mg, 0.69 mmol, 0.2 equiv). The resulting solution was stirred overnight at rt. Solvents were then evaporated under reduced pressure and the residue was purified by column chromatography on silica gel (cyclohexane/EtOAc, 90:10) to afford the desired product as a white solid (699 mg, 81%). LC tr = 2.67 min, MS (ESI+) *m*/*z*: 247 [M + H]^+^. ^1^H NMR (300 MHz, CDCl_3_): δ (ppm) 7.33–7.18 (m, 5H), 3.49 (t, *J* = 5.4 Hz, 1H), 2.88–2.82 (m, 2H), 2.44–2.37 (m, 2H), 1.75 (s, 3H), 1.73 (s, 3H). ^13^C NMR (75 MHz, CDCl_3_): δ (ppm) 165.4, 140.4, 128.65, 128.61, 126.4, 104.9, 45.1, 32.4, 28.5, 28.0, 26.6.

##### 5-(Cyclohexylmethyl)-2,2-dimethyl-1,3-dioxane-4,6-dione (**49f**)

Compound **49f** was prepared from Meldrum acid (300 mg, 2.082 mmol, 1.0 equiv), cyclohexanecarbaldehyde (252 μL, 2.082 mmol, 1.0 equiv), l-proline (47.93 mg, 0.416 mmol, 0.3 equiv), EtOH (5 mL), acetic acid (357 μL), sodium borohydride (236.25 mg, 6.245 mmol, 3.0 equiv), using general procedure A. After flash chromatography on silica gel (cyclohexane/EtOAc, 90/10 to 50/50), the desired compound was obtained as a white solid (353 mg, 71%). LC tr = 2.92 min, MS (ESI+) *m*/*z*: 241 [M + H]^+^.

##### 2,2-Dimethyl-5-(tetrahydropyran-4-ylmethyl)-1,3-dioxane-4,6-dione (**49g**)

Compound **49g** was prepared from Meldrum’s acid (1.44 g, 10.0 mmol, 1.0 equiv), tetrahydropyrancarbaldehyde (1.14 g, 10.0 mmol, 1.0 equiv), l-proline (230 mg, 2.0 mmol, 0.2 equiv), EtOH (25 mL), acetic acid (1.95 mL), sodium borohydride (1.13 g, 30 mmol, 3.0 equiv) using general procedure A. After flash chromatography on silica gel (DCM/AcOEt, 100/0 to 80/20), the desired product was obtained as a white solid (1g, 41%). LC tr = 1.67 min, MS (ESI+) *m*/*z*: 243 [M + H]^+^. ^1^H NMR (300 MHz, CDCl_3_): δ (ppm) 3.86 (dd, *J* = 10.8, 4.1 Hz, 2H), 3.49 (t, *J* = 5.7 Hz, 1H), 3.38 (td, *J* = 11.7, 2.0 Hz, 2H), 2.07 (t, *J* = 6.4 Hz, 2H), 1.90 (m, 1H), 1.80 (s, 3H), 1.77 (s, 3H), 1.60 (d, *J* = 13.9 Hz, 2H), 1.38 (td, *J* = 12.1, 4.5 Hz, 2H). ^13^C NMR (75 MHz, CDCl_3_): δ (ppm) 165.78, 105.00, 67.71, 43.13, 33.40, 32.68, 32.65, 29.70, 28.56, 26.9.

##### *tert*-Butyl *N*-[2-(5-Hydroxy-1*H*-indol-3-yl)ethyl]carbamate (**51a**)

Serotonin hydrochloride **50a** (1.064 g, 5.01 mmol, 1.0 equiv) was dissolved in CHCl_3_ (10 mL), then NaHCO_3_ (446 mg, 5.31 mmol, 1.0 equiv) in water (7 mL), NaCl (1.170 g, 20 mmol, 4.0 equiv) and di*tert*-butyl dicarbonate (1.110 g, 5.01 mmol, 1.0 equiv) in CHCl_3_ (2.5 mL) were added. The mixture was refluxed for 3 h and filtered. The aqueous phase was extracted with CHCl_3_ and the combined organic layers were washed with water and brine, dried over MgSO_4_, filtered and concentrated under reduced pressure. The crude was purified by flash chromatography on silica gel (DCM/MeOH, 100:0 to 95:5), to afford the pure product as a reddish-brown oil (1.380 g, 85%). LC tr = 2.35 min, MS (ESI−) *m*/*z*: 275 [M − H]^−^. ^1^H NMR (300 MHz, CDCl_3_): δ (ppm) 8.16 (br s, 1H), 7.16 (d, *J* = 8.6 Hz, 1H), 6.99 (d, *J* = 2.2 Hz, 1H), 6.91 (br s, 1H), 6.78 (dd, *J* = 8.6, 2.2 Hz, 1H), 4.73 (br s, 1H), 3.90 (d, *J* = 5.6 Hz, 2H), 2.81 (t, *J* = 6.4 Hz, 2H), 1.44 (s, 9H). ^13^C NMR (75 MHz, CDCl_3_): δ (ppm) 156.6, 150.1, 147.1, 131.8, 128.3, 123.5, 112.4, 112.2, 103.6, 85.6, 41.0, 28.7, 27.7.

##### Methyl 2-(*tert*-Butoxycarbonylamino)-3-(5-hydroxy-1*H*-indol-3-yl)propanoate (**51b**)

Methyl (2*S*)-2-amino-3-(5-hydroxy-1*H*-indol-3-yl) propanoate **50b** (5.0 g, 21.3 mmol, 1.0 equiv) was dissolved in MeOH (250 mL). To the solution, SOCl_2_ (4 mL, 54.8 mmol, 2.5 equiv) was added dropwise while stirring in an ice bath. The mixture was stirred overnight at rt and concentrated under reduced pressure. The residue was dissolved in DCM (250 mL) and Et_3_N (30 mL) was added dropwise. Then, Boc_2_O (7.45 g, 34.2 mmol, 1.6 equiv) was added. The reaction mixture was stirred overnight, concentrated under reduced pressure and purified by reverse phase chromatography (H_2_O/ACN, 90:10 to 0:100) to give compound **51b** as a red solid (3.5 g, 49%). LC tr = 1.75 min, MS (ESI+) *m*/*z*: 335 [M + H]^+^. ^1^H NMR (300 MHz, MeOD-*d*_4_): δ (ppm) 7.18 (d, *J* = 8.6 Hz, 1H), 7.02 (s, 1H), 6.92 (dd, *J* = 2.4, 0.6 Hz, 1H), 6.68 (dd, *J* = 8.7, 2.4 Hz, 1H), 4.47–4.36 (m, 1H), 3.68 (s, 3H), 3.18 (dd, *J* = 14.6, 5.8 Hz, 1H), 3.06 (dd, *J* = 14.6, 7.6 Hz, 1H), 1.41 (s, 9H).

##### *tert*-Butyl *N*-[2-[5-(2-Pyridylmethoxy)-1*H*-indol-3-yl]ethyl]carbamate (**52aa**)

Compound **52aa** was prepared from **51a** (200 mg, 0.72 mmol, 1.0 equiv), K_2_CO_3_ (200 mg, 1.45 mmol, 2.0 equiv), ACN (4 mL), (3-methoxy)phenethyl bromide bromide (164 mg, 0.76 mmol, 1.05 equiv) using general procedure D. After flash chromatography on silica gel (cyclohexane/EtOAc, 90:10 to 70:30), the desired product was obtained as a light-yellow oil (130 mg, 49%). LC tr = 2.60 min, MS (ESI+) *m*/*z*: 368 [M + H]^+^. ^1^H NMR (300 MHz, CDCl_3_): δ (ppm) 8.60 (ddd, *J* = 4.9, 1.8, 0.9 Hz, 1H), 8.42 (br s, 1H), 7.70 (td, *J* = 7.7, 1.8 Hz, 1H), 7.59 (d, *J* = 7.7 Hz, 1H), 7.24 (dd, *J* = 8.8, 0.5 Hz, 1H), 7.23–7.18 (m, 1H), 7.11 (d, *J* = 2.4 Hz, 1H), 6.97 (br s, 1H), 6.94 (dd, *J* = 8.8, 2.4 Hz, 1H), 5.25 (s, 2H), 4.69 (br s, 1H), 3.41 (q, *J* = 6.0 Hz, 2H), 2.86 (t, *J* = 6.8 Hz, 2H), 1.43 (s, 9H). ^13^C NMR (75 MHz, CDCl_3_): δ (ppm) 157.9, 156.0, 152.6, 149.0, 136.8, 131.9, 127.7, 123.1, 122.5, 121.5, 112.7, 112.5, 112.0, 102.5, 79.2, 71.5, 40.6, 28.4, 25.7.

##### *tert*-Butyl *N*-[2-[5-(3-Pyridylmethoxy)-1*H*-indol-3-yl]ethyl]carbamate (**52ab**)

Compound **52ab** was prepared from **51a** (200 mg, 0.72 mmol, 1.0 equiv), 3-(bromomethyl)pyridine (192 mg, 0.76 mmol, 1.05 equiv), K_2_CO_3_ (300 mg, 2.17 mmol, 3.0 equiv), ACN (5 mL) using general procedure D. After flash chromatography on silica gel (cyclohexane/EtOAc, 90:10 to 70:30), the desired product was obtained as a beige solid (106 mg, 40%). LC tr = 2.48 min, MS (ESI+) *m*/*z*: 368 [M + H]^+^.

##### *tert*-Butyl *N*-[2-[5-(4-Pyridylmethoxy)-1*H*-indol-3-yl]ethyl]carbamate (**52ac**)

Compound **52ac** was prepared from **51a** (200 mg, 0.72 mmol, 1.0 equiv), 4-chloromethylpyridine hydrochloride (131 mg, 0.80 mmol, 1.1 equiv), K_2_CO_3_ (300 mg, 2.18 mmol, 3.0 equiv), DMF (4 mL) using general procedure D. After flash chromatography on silica gel (cyclohexane/EtOAc, 90:10 to 70:30), the desired product was obtained as a light-purplish oil (97 mg, 37%). LC tr = 2.42 min, MS (ESI+) *m*/*z*: 368 [M + H]^+^. ^1^H NMR (300 MHz, CDCl_3_): δ (ppm) 8.81 (br s, 1H), 8.61–8.59 (m, 2H), 7.41–7.39 (m, 2H), 7.25 (d, *J* = 8.8 Hz, 1H), 7.10 (d, *J* = 2.0 Hz, 1H), 6.99 (d, *J* = 2.0 Hz, 1H), 6.91 (dd, *J* = 8.8, 2.4 Hz, 1H), 5.11 (s, 2H), 4.79 (br s, 1H), 3.43 (br q, *J* = 6.5 Hz, 2H), 2.93–2.87 (m, 2H), 1.44 (s, 9H). ^13^C NMR (75 MHz, CDCl_3_): δ (ppm) 156.1, 152.5, 149.7, 147.1, 132.1, 127.8, 123.3, 121.7, 112.6, 112.5, 112.1, 102.4, 79.2, 69.1, 40.8, 28.4, 25.8.

##### *tert*-Butyl *N*-[2-(5-Benzyloxy-1*H*-indol-3-yl)ethyl]carbamate (**52ad**)

Compound **52ad** was prepared from **51a** (235 mg, 0.85 mmol, 1.0 equiv), K_2_CO_3_ (352 mg, 2.55 mmol, 3 equiv), benzyl bromide (202 μL, 1.70 mmol, 2.0 equiv), DMF (4 mL) using general procedure D. After flash chromatography on silica gel (cyclohexane/EtOAc, 90:10 to 70:30), the desired product was obtained as a colorless oil (251 mg, 81%). LC tr = 3.03 min, MS (ESI+) *m*/*z*: 367 [M + H]^+^.

##### *tert*-Butyl *N*-[2-[5-[(3-Fluorophenyl)methoxy]-1*H*-indol-3-yl]ethyl]carbamate (**52ae**)

Compound **52ae** was prepared from **51a** (200 mg, 0.72 mmol, 1.0 equiv), 3-fluorobenzyl bromide (149 mg, 0.76 mmol, 1.05 equiv), K_2_CO_3_ (300 mg, 2.17 mmol, 3.0 equiv), DMF (5 mL) using general procedure D. After flash chromatography on silica gel (cyclohexane/EtOAc, 90:10 to 60:40), the desired product was obtained as a beige solid (279, quant.). LC tr = 3.08 min, MS (ESI+) *m*/*z*: 385 [M + H]^+^.

^1^H NMR (300 MHz, CDCl_3_): δ (ppm) 8.44 (br s, 1H), 7.30 (td, *J* = 7.9, 5.7 Hz, 1H), 7.22–7.16 (m, 3H), 7.09 (d, *J* = 2.2 Hz, 1H), 6.97 (tdd, *J* = 8.5, 2.5, 1.0 Hz, 1H), 6.90 (dd, *J* = 8.6, 2.5 Hz, 1H), 6.90 (br s, 1H), 5.04 (s, 2H), 4.74 (br s, 1H), 3.42 (q, *J* = 6.6 Hz, 2H), 2.87 (t, *J* = 6.6 Hz, 2H), 1.44 (s, 9H). ^13^C NMR (75 MHz, CDCl_3_): δ (ppm) 162.9 (d, *J* = 245.8 Hz), 156.1, 152.8, 140.3 (d, *J* = 7.3 Hz), 131.9, 130.0 (d, *J* = 8.4 Hz), 127.7, 123.1, 122.9 (d, *J* = 2.4 Hz), 114.6 (d, *J* = 21.6 Hz), 114.3 (d, *J* = 21.7 Hz), 112.7, 112.5, 112.0, 102.4, 79.2, 70.2, 40.8, 28.4, 25.8.

##### *tert*-Butyl *N*-[2-[5-[(4-Fluorophenyl)methoxy]-1*H*-indol-3-yl]ethyl]carbamate (**52af**)

Compound **52af** was prepared from **51a** (200 mg, 0.72 mmol, 1.0 equiv), 4-fluorobenzyl bromide (149 mg, 0.76 mmol, 1.05 equiv), K_2_CO_3_ (300 mg, 2.17 mmol, 3.0 equiv), DMF (5 mL) using general procedure D. After flash chromatography on silica gel (cyclohexane/EtOAc, 90:10 to 60:40), the desired product was obtained as a beige solid (253 mg, 91%). LC tr = 3.07 min, MS (ESI+) *m*/*z*: 385 [M + Na]^+^.

^1^H NMR (CDCl_3_, 300 MHz): δ (ppm) 8.47 (br s, 1H), 7.39 (dd, *J* = 8.7, 5.5 Hz, 2H), 7.19 (d, *J* = 8.8 Hz, 1H), 7.10 (d, *J* = 2.0 Hz, 1H), 7.03 (t, *J* = 8.7 Hz, 2H), 6.88 (dd, *J* = 8.8, 2.3 Hz, 1H), 6.89 (br s, 1H), 5.01 (s, 2H), 4.75 (br s, 1H), 3.42 (q, *J* = 6.6 Hz, 2H), 2.87 (t, *J* = 6.6 Hz, 2H), 1.44 (s, 9H). ^13^C NMR (75 MHz, CDCl_3_): δ (ppm) 162.4 (d, *J* = 245.7 Hz), 156.2, 152.9, 133.4 (d, *J* = 3.6 Hz), 131.9, 129.5 (d, *J* = 7.9 Hz), 127.7, 123.1, 115.3 (d, *J* = 21.5 Hz), 112.7, 112.5, 112.0, 102.4, 79.2, 70.3, 40.9, 28.4, 25.8.

##### *tert*-Butyl *N*-[2-[5-[(6-Chloro-3-pyridyl)methoxy]-1*H*-indol-3-yl]ethyl]carbamate (**52ag**)

Compound **52ag** was prepared from **51a** (200 mg, 0.72 mmol, 1.0 equiv), DIAD (171 μL, 0.87 mmol, 1.2 equiv), (6-chloro-3-pyridyl)methanol (125 mg, 0.87 mmol, 1.2 equiv), PPh_3_ (228 mg, 0.87 mmol, 1.2 equiv) using general procedure E. After flash chromatography on silica gel (cyclohexane/EtOAc, 90:10 to 50:50), the desired product was obtained (266 mg, 55%). LC tr = 2.47 min, MS (ESI+) *m*/*z*: 402 [M + H]^+^.

##### *tert*-Butyl *N*-[2-[5-(Pyrimidin-2-ylmethoxy)-1*H*-indol-3-yl]ethyl]carbamate (**52ah**)

Compound **52ah** was prepared from **51a** (212 mg, 0.77 mmol, 1.0 equiv), pyrimidin-2-ylmethanol (101 mg, 0.92 mmol, 1.2 equiv), PPh_3_ (241 mg, 0.92 mmol, 1.2 equiv), DIAD (181 μL, 0.92 mmol, 1.2 equiv) using general procedure E. After flash chromatography on silica gel (DCM/MeOH, 95:5), the desired product was obtained (98 mg, 35%). LC tr = 2.34 min, MS (ESI+) *m*/*z*: 369 [M + H]^+^. ^1^H NMR (300 MHz, CDCl_3_): δ (ppm) 9.23 (s, 1H), 8.91 (s, 2H), 7.30 (d, *J* = 8.8 Hz, 1H), 7.27 (s, 3H), 7.17 (d, *J* = 2.3 Hz, 1H), 7.06 (br s, 1H), 7.01 (dd, *J* = 8.7, 2.4 Hz, 1H), 5.37 (s, 2H), 3.42 (m, 1H), 2.88 (t, *J* = 6.7 Hz, 2H), 1.43 (s, 9H).

##### *tert*-Butyl *N*-[2-[5-[2-(3-Methoxyphenyl)ethoxy]-1*H*-indol-3-yl]ethyl]carbamate (**52ai**)

Compound **52ai** was prepared from **51a** (200 mg, 0.72 mmol, 1.0 equiv), Cs_2_CO_3_ (700 mg, 2.16 mmol, 3.0 equiv), (3-Methoxy)phenethyl bromide (164 mg, 0.76 mmol, 1.05 equiv), ACN (4 mL) using general procedure D. After flash chromatography on silica gel (cyclohexane/EtOAc, 90:10 to 70:30), the desired product was obtained as a light-brownish oil (141 mg, 48%). LC tr = 3.08 min, MS (ESI+) *m*/*z*: 411 [M + H]^+^.

##### *tert*-Butyl *N*-[2-[5-(2-Morpholinoethoxy)-1*H*-indol-3-yl]ethyl]carbamate (**52aj**)

Compound **52aj** was prepared from **51a** (200 mg, 0.72 mmol, 1.0 equiv), K_2_CO_3_ (300 mg, 2.18 mmol, 3.0 equiv), *N*-(2-chloroethyl)morpholine hydrochloride (148 mg, 0.80 mmol, 1.1 equiv), DMF (4 mL) using general procedure D. After flash chromatography on silica gel (cyclohexane/EtOAc, 90:10 to 70:30), the desired product was obtained as a colorless oil (197 mg, 70%). LC tr = 2.08 min, MS (ESI+) *m*/*z*: 390 [M + H]^+^.^1^H NMR (300 MHz, CDCl_3_): δ (ppm) 8.73 (br s, 1H), 7.21 (d, *J* = 8.7 Hz, 1H), 7.04 (d, *J* = 2.3 Hz, 1H), 6.93 (d, *J* = 1.6 Hz, 1H), 6.82 (dd, *J* = 8.7, 2.3 Hz, 1H), 4.83 (br s, 1H), 4.15 (t, *J* = 5.7 Hz, 2H), 3.75–3.71 (m, 4H), 3.45–3.37 (m, 2H), 2.91–2.86 (m, 2H), 2.81 (t, *J* = 5.7 Hz, 2H), 2.61–2.58 (m, 4H), 1.44 (s, 9H). ^13^C NMR (75 MHz, CDCl_3_): δ (ppm) 156.1, 152.8, 131.8, 127.7, 123.1, 112.6, 112.4, 112.0, 101.9, 79.1, 66.8, 66.4, 57.8, 54.0, 40.8, 28.4, 25.8.

##### Methyl 3-(5-Benzyloxy-1*H*-indol-3-yl)-2-(*tert*-butoxycarbonylamino)propanoate (**52ak**)

Compound **52a***k* was prepared from **51b** (2.0 g, 4.49 mmol, 1.0 equiv) Cs_2_CO_3_ (4.4 g, 13.5 mmol, 3.0 equiv), bromomethylbenzene (1.02 g, 5.98 mmol, 1.3 equiv), DMF (15 mL) using general procedure D. After reverse phase chromatography (H_2_O/ACN, 90:10 to 0:100), the desired product was obtained as a white solid (800 mg, 42%). LC tr = 2.31 min, MS (ESI+) *m*/*z*: 464 [M + K]^+^. ^1^H NMR (300 MHz, DMSO-*d*_6_): δ (ppm) 10.71 (s, 1H), 7.54–7.45 (m, 2H), 7.45–7.18 (m, 5H), 7.13 (dd, *J* = 4.6, 2.4 Hz, 2H), 6.80 (dd, *J* = 8.7, 2.4 Hz, 1H), 5.10 (s, 2H), 4.27–4.04 (m, 1H), 3.61 (s, 3H), 3.13–2.89 (m, 2H), 1.33 (s, 9H).

##### Methyl (2*S*)-2-(*tert*-Butoxycarbonylamino)-3-[5-(4-pyridylmethoxy)-1*H*-indol-3-yl]propanoate (**52al**)

Compound **52al** was prepared from **51b** (1.5 g, 3.36 mmol, 1.0 equiv), Cs_2_CO_3_ (3.3 g, 10.1 mmol, 3.0 equiv), 4-(chloromethyl)pyridine (0.750 g, 5.88 mmol, 1.75 equiv), DMF (40 mL) using general procedure D. After reverse phase chromatography (H_2_O/ACN, 90:10 to 0:100), the desired product was obtained as a pale-yellow solid (600 mg, 42%). LC tr = 1.92 min, MS (ESI+) *m*/*z*: [M + K]^+^ 464. ^1^H NMR (300 MHz, DMSO-*d*_6_): δ (ppm) 10.74 (s, 1H), 8.62–8.54 (m, 2H), 7.52–7.44 (m, 2H), 7.24 (t, *J* = 8.2, 8.2 Hz, 2H), 7.13 (d, *J* = 2.4 Hz, 2H), 6.83 (dd, *J* = 8.7, 2.4 Hz, 1H), 5.18 (s, 2H), 4.26–4.04 (m, 2H), 3.61 (s, 3H), 3.18 (d, *J* = 5.2 Hz, 1H), 3.13–2.88 (m, 2H), 1.32 (s, 10H).

##### Methyl 2-[[3-[2-(*tert*-Butoxycarbonylamino)ethyl]-1*H*-indol-5-yl]oxy]acetate (**52am**)

Compound **52am** was prepared from **51a** (200 mg, 0.72 mmol, 1.0 equiv), Cs_2_CO_3_ (700 mg, 2.16 mmol, 3.0 equiv), methyl 2-bromoacetate (75 μL, 0.80 mmol, 1.1 equiv), ACN (4 mL) using general procedure D. After flash chromatography on silica gel (DCM/MeOH, 100:0 to 98:2), the desired product was obtained as a colorless waxy solid (95 mg, 38%). LC tr = 2.58 min, MS (ESI+) *m*/*z*: 349 [M + H]^+^.

^1^H NMR (300 MHz, CDCl_3_): δ (ppm) 8.49 (br s, 1H), 7.23 (d, *J* = 8.8 Hz, 1H), 7.03 (d, *J* = 2.4 Hz, 1H), 6.94 (d, *J* = 1.9 Hz, 1H), 6.89 (dd, *J* = 8.8, 2.4 Hz, 1H), 4.70 (br s, 1H), 4.68 (s, 2H), 3.80 (s, 3H), 3.41 (q, *J* = 6.2 Hz, 2H), 2.86 (t, *J* = 6.8 Hz, 2H), 1.44 (s, 9H). ^13^C NMR (75 MHz, CDCl_3_): δ (ppm) 170.1, 156.1, 152.1, 132.3, 127.6, 123.3, 112.6, 112.5, 112.2, 102.6, 79.2, 66.7, 52.2, 40.7, 28.4, 25.8.

##### 2-[[3-[2-(*tert*-Butoxycarbonylamino)ethyl]-1*H*-indol-5-yl]oxy]acetic Acid (**53**)

To the solution of compound **52am′** (680 mg, 1.95 mmol, 1.0 equiv) in MeOH (4 mL) was added NaOH (101 mg, 2.53 mmol, 1.3 equiv) in water (1 mL). The reaction mixture was stirred at rt for overnight. Solvents were evaporated under reduced pressure and the residue was partitioned between 5% NaHCO_3_ (aq) and DCM. The aqueous layer was carefully acidified to pH ∼ 3–4 with HCl (1 M) and was extracted three times with DCM. Organic layers were combined and the solvent was evaporated under vacuum, affording the desired product as a yellow residue (613 mg, 94%). LC tr = 2.08 min, MS (ESI+) *m*/*z*: 335 [M + H]^+^.

##### *tert*-Butyl *N*-[2-[5-(2-Morpholino-2-oxo-ethoxy)-1*H*-indol-3-yl]ethyl]carbamate (**54am**)

Compound **54am** was prepared from **53** (150 mg, 0.45 mmol, 1.0 equiv), HOBT (91 mg, 0.67 mmol, 1.5 equiv), EDCI (112 mg, 0.58 mmol, 1.3 equiv), Et_3_N (313 μL, 2.24 mmol, 5.0 equiv), morpholine (78 μL, 0.90 mmol, 2.0 equiv), DMF (4 mL) using general procedure G. After flash chromatography on silica gel (cyclohexane/EtOAc, 70:30 to 20:80), the desired product was obtained as a brown solid (161 mg, 89%). LC tr = 2.31 min, MS (ESI+) *m*/*z*: 404 [M + H]^+^. ^1^H NMR (300 MHz, CDCl_3_): δ (ppm) 8.95 (br s, 1H), 7.24 (d, *J* = 8.8 Hz, 1H), 7.08 (d, *J* = 2.3 Hz, 1H), 6.96 (d, *J* = 2.3 Hz, 1H), 6.85 (dd, *J* = 8.8, 2.3 Hz, 1H), 4.82 (br s, 1H), 4.72 (s, 1H), 3.64 (br s, 8H), 3.44–3.37 (m, 2H), 2.87 (br t, *J* = 6.8 Hz, 2H), 1.43 (s, 9H). ^13^C NMR (75 MHz, CDCl_3_): δ (ppm) 167.3, 156.1, 151.9, 132.3, 127.7, 123.5, 112.4, 112.2, 112.0, 102.2, 79.1, 68.6, 66.8, 45.9, 42.4, 40.7, 28.4, 25.8.

##### *tert*-Butyl *N*-[2-[5-[2-Oxo-2-(prop-2-ynylamino)ethoxy]-1*H*-indol-3-yl]ethyl]carbamate (**54an**)

Compound **54an** was prepared from **53** (150 mg, 0.45 mmol, 1.0 equiv), HOBT (91 mg, 0.67 mmol, 1.5 equiv) and EDCI (112 mg, 0.58 mmol, 1.3 equiv), Et_3_N (313 μL, 2.24 mmol, 5.0 equiv), propargylamine (57 μL, 0.90 mmol, 2.0 equiv), DMF (4 mL) using general procedure G. After flash chromatography on silica gel (cyclohexane/EtOAc, 70:30 to 20:80), the desired product was obtained as a light brown solid (116 mg, 70%). LC tr = 2.41 min, MS (ESI+) *m*/*z*: 372 [M + H]^+^. ^1^H NMR (300 MHz, CDCl_3_): δ (ppm) 8.69 (br s, 1H), 7.27 (d, *J* = 8.8 Hz, 1H), 7.08–7.04 (m, 2H), 7.04 (d, *J* = 2.4 Hz, 1H), 7.01 (d, *J* = 2.0 Hz, 1H), 6.84 (dd, *J* = 8.8, 2.4 Hz, 1H), 4.77 (br s, 1H), 4.54 (s, 1H), 4.15 (dd, *J* = 5.6, 2.6 Hz, 2H), 3.46–3.39 (m, 2H), 2.88 (br t, *J* = 6.8 Hz, 2H), 2.27 (t, *J* = 2.6 Hz, 1H), 1.44 (s, 9H). ^13^C NMR (75 MHz, CDCl_3_): δ (ppm) 168.9, 156.1, 151.4, 132.0, 127.8, 123.6, 112.7, 112.3, 111.9, 102.8, 79.3, 79.2, 71.8, 68.5, 40.8, 28.7, 28.4, 25.8.

##### *tert*-Butyl *N*-[2-[5-[2-(Benzylamino)-2-oxo-ethoxy]-1*H*-indol-3-yl]ethyl]carbamate (**54ao**)

Compound **54ao** was prepared from **53** (150 mg, 0.45 mmol,1.0 equiv), HOBt (61 mg, 0.45 mmol, 1.0 equiv), EDCI (260 mg, 1.35 mmol, 3.0 equiv), Et_3_N (0.3 mL, 2.24 mmol, 5.0 equiv), benzylamine (100 μL, 0.90 mmol, 2.0 equiv), DMF (4 mL) using general procedure G. After flash chromatography on silica gel (cyclohexane/EtOAc, 90:10 to 70:30), the desired product was obtained as a colorless solid (91 mg, 48%). LC tr = 2.70 min, MS (ESI+) *m*/*z*: 424 [M + H]^+^. ^1^H NMR (300 MHz, CDCl_3_): δ (ppm) 7.29–7.23 (m, 6H), 7.12 (br s, 1H), 7.04 (br s, 1H), 7.00 (br s, 1H), 6.84–6.80 (dd, *J* = 8.7, 2.4 Hz, 1H), 4.71 (s, 1H), 4.59 (s, 2H), 4.55 (d, *J* = 5.9 Hz, 2H), 3.42–3.40 (m, 2H), 2.87–2.85 (m, 2H), 1.43 (s, 9H). ^13^C NMR (75 MHz, CDCl_3_): δ (ppm) 169.0, 156.0, 151.5, 137.8, 132.3, 128.7, 127.8, 127.7, 127.5, 123.5, 112.8, 112.2, 111.9, 102.8, 79.2, 68.6, 42.9, 40.8, 28.4, 25.8.

##### *tert*-Butyl *N*-[2-[5-[2-[2-(4-Hydroxyphenyl)ethylamino]-2-oxo-ethoxy]-1*H*-indol-3-yl]ethyl]carba-mate (**54ap**)

Compound **54ap** was prepared from **53** (120 mg, 0.36 mmol, 1.0 equiv), HBTU (150 mg, 0.40 mmol, 1.1 equiv), Et_3_N (150 μL, 1.08 mmol, 3.0 equiv), tyramine (54 mg, 0.4 mmol, 1.1 equiv), DMF (4 mL) using general procedure G. After flash chromatography on silica gel (DCM/MeOH, 100:0 to 95:5), the desired product was obtained as a brown waxy solid (147 mg, 90%). LC tr = 2.42 min, MS (ESI+) *m*/*z*: 454 [M + H]^+^. ^1^H NMR (300 MHz, CDCl_3_): δ (ppm) 8.71 (br s, 1H), 8.15 (br s, 1H), 7.22 (d, *J* = 8.8 Hz, 1H), 7.00–6.89 (m, 4H), 6.85 (br t, *J* = 5.8 Hz, 1H), 6.79 (br d, *J* = 8.2 Hz, 2H), 6.72 (dd, *J* = 8.8, 2.4 Hz, 1H), 4.91 (br t, *J* = 5.5 Hz, 1H), 4.48 (s, 2H), 3.55 (q, *J* = 6.4 Hz, 2H), 3.42 (q, *J* = 6.4 Hz, 2H), 2.87 (t, *J* = 6.7 Hz, 2H), 2.72 (t, *J* = 6.7 Hz, 2H), 1.43 (s, 9H). ^13^C NMR (75 MHz, CDCl_3_): δ (ppm) 169.2, 156.4, 155.6, 151.4, 132.4, 129.8, 129.4, 127.8, 123.7, 115.7, 112.4, 112.1, 110.0, 103.4, 79.7, 68.4, 40.9, 40.2, 34.6, 28.4, 25.8.

## Supporting Information Available

Supplementary figures: docking of **43** (*S*,*S* isomer) in semi open IRAP, **43** (*R*,*S* isomer) in closed IRAP, comparison of binding of **43** and **B32e**; NMR experiment on malonic proton exchange; supplementary tables: activities of inhibitors of PfAM1; LE and LLE for selected inhibitors; calculated physicochemical properties for selected inhibitors; cellular toxicities; supplementary methods for physicochemical properties calculations; NMR spectra for final compounds (PDF)
Molecular formula strings and associated biological data (CSV)
Coordinates of modeled structures of 43 (*SS* isomer and *RS* isomer) in IRAP (closed or semiopen conformations) (ZIP)

## Abbreviations

7-AAD: 7-aminoactinomycine D
ACN: acetonitrile
BBB: blood brain barrier
DBU: 1,8-diazabicyclo[5.4.0]undec-7-ene
DCM: dichloromethane
DIAD: diisopropyl azodicarboxylate
DMSO: dimethyl sulfoxide
EDCI: EDCI
ERAP1: endoplasmic reticulum aminopeptidase 1
ERAP2: endoplasmic reticulum aminopeptidase 2
HBTU: hexafluorophosphate benzotriazole tetramethyl uronium
HOBt: hydroxybenzotriazole
IRAP: insulin-regulated aminopeptidase
l-AMC: l-leucine-7-amido-4-methylcoumarin
LC: liquid chromatography
LE: ligand efficiency
LLE: lipophilic ligand efficiency
log *P*: partition coefficient (*P*)
MHC class-I: major histocompatibility complex, class-I
MS: mass spectrometry
NMR: nuclear magnetic resonance
PDB: Protein data bank
*Pf*: *Plasmodium falciparum*
PSA: polar surface area
R-AMC: l-arginine-7-amido-4-methylcoumarin
rt: room temperature
TCR: T-cell receptor
