# Supplementary material for: Discovery of New Nanomolar Selective IRAP Inhibitors
Source: J Med Chem. 2025 Feb 7;68(4):4168–95. doi: 10.1021/acs.jmedchem.4c01744 (PMC11874008; doi:10.1021/acs.jmedchem.4c01744)
Supplement: Supplementary file 1 — jm4c01744_si_001.pdf [file jm4c01744_si_001.pdf]

Supporting Information for

Discovery of new nanomolar selective IRAP inhibitors

Ben He;[1,‡] Nour Bou Karroum; [1,‡] Ronan Gealageas[1] ; François-Xavier Mauvais;[3,4] Sandrine Warenghem;[1] Matthieu Roignant;[1] Nicolas Kraupner;[1] Bao Vy Lam;[1] Nathalie Azaroual;[5] Vincent Ultré,[6] Alexandre Rech;[6] Laetitia Lesire;[1] Cyril Couturier;[1] Florence Leroux;[1,2] Peter van Endert ; [3,7] Benoit Deprez; [1,2] Rebecca Deprez-Poulain[1,2,*]

1. Univ. Lille, Inserm, Institut Pasteur de Lille, U1177 - Drugs and Molecules for Living Systems, F-59000 Lille, France;
2. European Genomic Institute for Diabetes, EGID, University of Lille, F-59000, France.
3. Université Paris Cité, INSERM, CNRS, Institut Necker Enfants Malades, F-75015 Paris, France
4. Service de Physiologie – Explorations Fonctionnelles, AP-HP, Hôpital Robert-Debré, F-75019 Paris, France
5. University Lille, CHU Lille, ULR 7365—GRITA—Groupe de Recherche Sur Les Formes Injectables Et Les Technologies Associées, F-59000 Lille, France
6. University Lille, Plateau RMN Pharmacie, UFR3S-Pharmacie, F-59000 Lille, France
7. Service Immunologie Biologique, AP-HP, Hôpital Universitaire Necker-Enfants Malades, F- 75015 Paris, France

Supplementary figures 2

Supplementary Figure S1: Putative Binding of 43 (*S,S isomer*) in hIRAP semi-open[a] 2

Supplementary Figure S2: Putative Binding of 43 (*R,S isomer*) in hIRAP [a] 3

Supplementary Figure S3: Compared binding of 43 (*S,S isomer*) & bestatin-derived inhibitor B32e[a] 4

Supplementary Figure S4: Kinetics of the proton deuterium exchange on the malonic carbon in 22 [a] 5

Supplementary Tables 6

Supplementary Table S1: Activities of inhibitors of *Pf*AM1[a] 6

Supplementary Table S2: LE and LLE for selected inhibitors[a] 6

Supplementary Table S3: Cellular toxicity for selected inhibitors at 30µM. 6

Supplementary Table S4: Calculated physicochemical properties for selected inhibitors[a] 6

Supplementary Methods 7

Caco-2 clogPapp and BBB clogPapp (EnalosSuite®) 7

cLogP and PSA (OSIRIS Datawarrior®) 7

HPLC chromatograms of inhibitors 12, 18, 22, 41,43. 8

NMR spectra for final compounds 11

‡ Contributed equally

* Corresponding author: rebecca.deprez@univ-lille.fr

Supplementary figures

Supplementary Figure S1: Putative Binding of **43** (*S,S isomer*) in hIRAP semi-open[a]

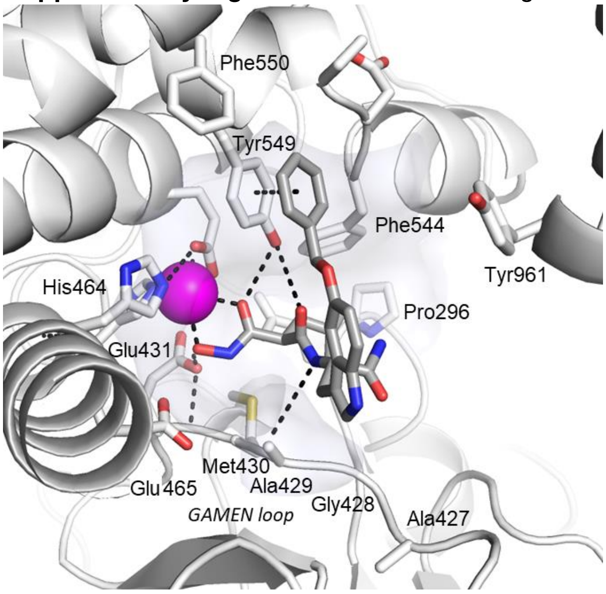

[a]Docking pose of **43** (*S,S isomer*) in hIRAP (PDB 4ZSI). Carbons are colored grey for hIRAP and **43** respectively. Oxygens, Nitrogens are in red, blue respectively. Zinc ion is represented as a magenta sphere. Polar contacts and interactions are represented as black dashed lines. The structures were rendered using PyMOL™ Molecular Graphics System v2.5.

Supplementary Figure S2: Putative Binding of **43** (*R,S isomer*) in hIRAP [a]

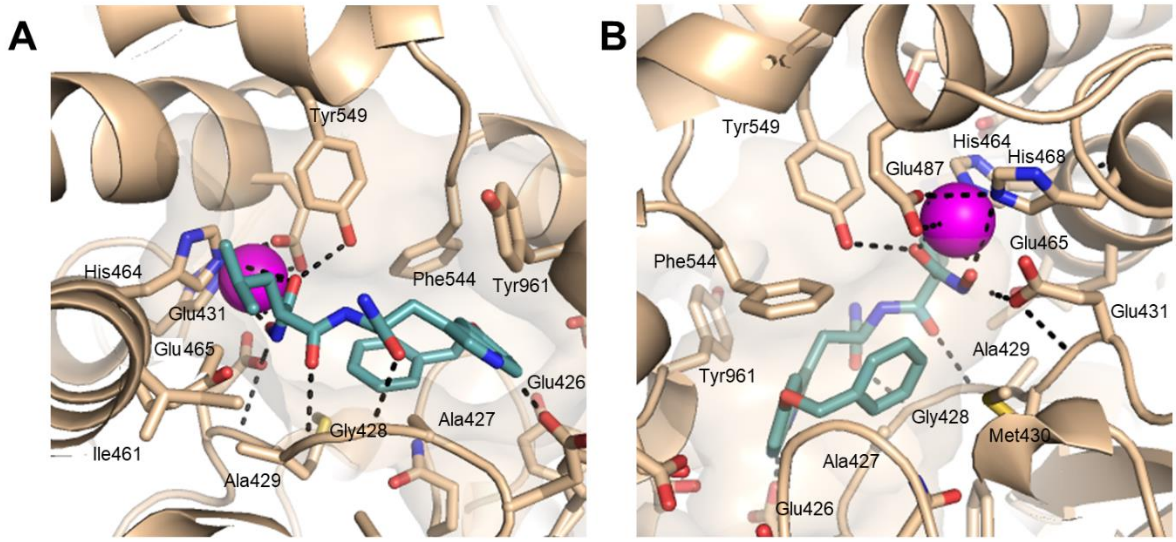

[a](A-B) two views of the docking pose of **43** (*R*,*S*) in hIRAP (PDB 7ZYF). Carbons are colored beige and teal for hIRAP and **43** respectively. Oxygens, Nitrogens are in red, blue respectively. Zinc ion is represented as a magenta sphere. Polar contacts and interactions are represented as black dashed lines. The structures were rendered using PyMOL^{TM} Molecular Graphics System v2.5.

Supplementary Figure S3: Compared binding of **43** (*S,S isomer*) & bestatin-derived inhibitor **B32e**[a]

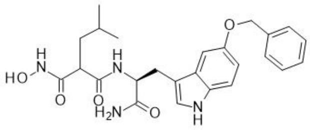

43

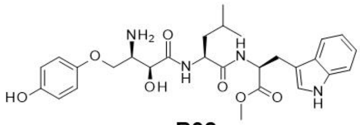

B32e

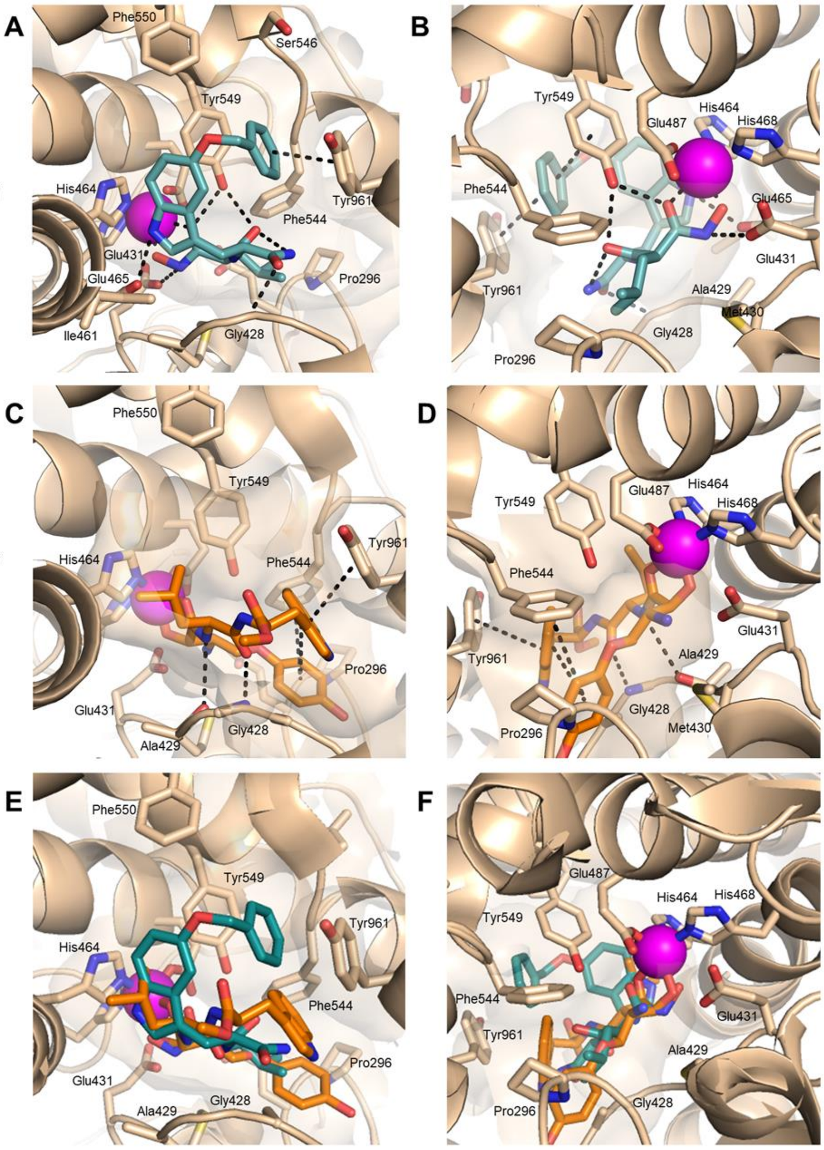

[a] (A-B) two views of the docking pose of **43** (S,S) in hIRAP Carbons are colored beige and teal for hIRAP and **43** respectively. (C-D) two views of the X-Ray structure of hIRAP in complex with **B32e** (PDB 7ZYF). Carbons are colored beige and orange for hIRAP and **B32e** respectively. (E-F) superimposition of **43** (S,S) and **B32e** in hIRAP (PDB 7ZYF) showing that the 2 inhibitors explore different pockets of the enzyme. Carbons are colored beige, teal and orange for hIRAP, **43** (S,S) and **B32e** respectively..Oxygens, Nitrogens are in red, blue respectively. Zinc ion is represented as a magenta sphere. Polar contacts and interactions are represented as black dashed lines. The structures were rendered using PyMOL^{TM} Molecular Graphics System v2.5.

Supplementary Figure S4: Kinetics of the proton deuterium exchange on the malonic carbon in **22** [a]

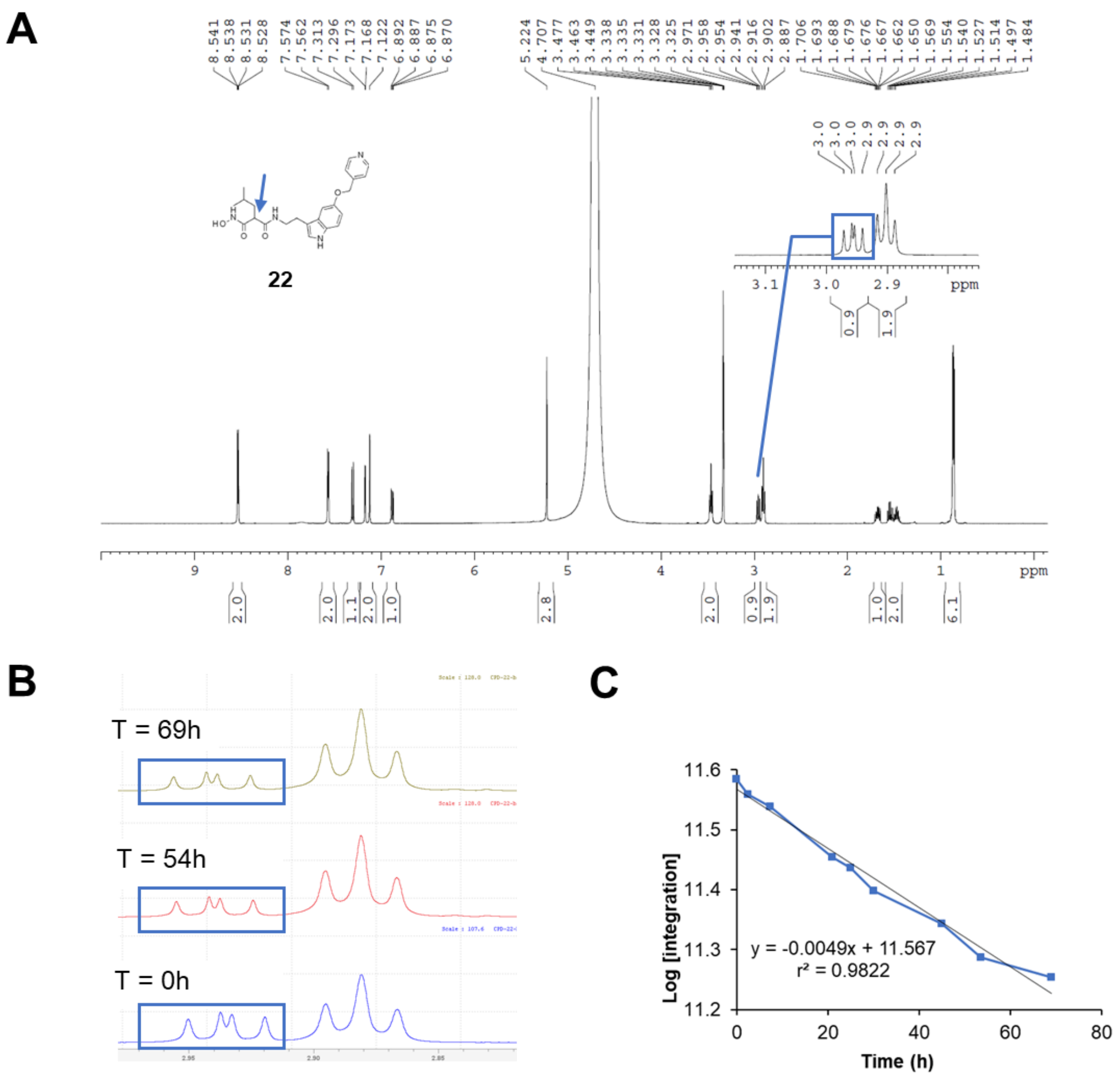

[a] (A-B) ^{1}H NMR spectra (500 MHz) at 37°C of **22** in MeOD-*d*$_4$ containing 5% D_2O and 1.0 eq. of Na_2CO_3 (A) Spectrum at t=0h, the proton on the malonic carbon is pointed by blue arrow and respective NMR signal is enlarged. (B) examples of ^{1}H NMR spectra at different time points, zoomed on the proton on the malonic carbon. (C) Log of the integration of the signal intensity of the proton on the malonic carbon in NMR at 37°C in function of time. Regression analysis provided a $T_{1/2}$ of 54 h.

Supplementary Tables

Supplementary Table S1: Activities of inhibitors of *Pf*AM1[a]

	*Pf*AM1 IC_{50} (nM)	*m*APN IC_{50} (nM)	*h*ERAP1 IC_{50} (nM)
**BDM_14471**	6	1372	nd
**BDM_14631**	310	1324	nd
**BDM_14470**	46	>10000	(65)[b]

[a] substrates : L-AMC. [b] % of inhibition at 300 µM; nd: not determined.

Supplementary Table S2: LE and LLE for selected inhibitors[a]

Cpd #	LE	LLE	IRAP IC_{50} (µM)
**6**	0.37	4.63	0.65
**7**	0.36	4.21	0.61
**8**	0.36	3.90	0.34
**9**	0.32	3.89	0.62
**12**	0.37	3.34	0.40
**18**	0.27	2.25	0.62
**22**	0.32	4.37	0.067
**41**	0.30	3.74	0.028
**42**	0.32	4.81	0.01
**43**	0.34	5.57	0.003

[a]:LE:LigandEfficiency;LLE:Lipophilic ligand efficiency

Supplementary Table S3: Cellular toxicity for selected inhibitors at 30µM.

Cpd #	HEK293T FcR-EGFP.K[b]		*wt* HEK293	
	IRAP-/-	*wt*		
	% 7-AAD+[a]	% 7-AAD+[a]	% PI+[b]	% Nucview+[c]
**12**	7.2 ± 2.0	7.7 ± 2.5	1.5 ± 0.25	3.7 ± 0.4
**18**	6.5 ± 1.4	7.6 ± 0.6	2.3 ± 0.4	5.6 ± 0.6
**22**	5.8 ± 1.9	6.6 ± 0.1	1.1 ± 0.2	2.7 ± 0.3
**41**	6.4 ± 0.1	4.8 ± 0.1	1.0 ± 0.1	2.4 ± 0.4
**42**	6.6 ± 3.1	7.4 ± 2.5	1.1 ± 0.2	2.6 ± 0.3
**43**	5 ± 0.2	4.1 ± 0.7	0.8 ± 0.3	2.1 ± 0.2

[a] % of cells positive to 7-aminoactinomycine D staining at 16h (late apoptotic or necrotic cells); [b] % of cells positive to propidium iodide (necrosis) at 24 h; [c] % of cells positive to Nucview (early apoptosis) at 24 h.

Supplementary Table S4: Calculated physicochemical properties for selected inhibitors[a]

Cpd #	HBA	HBD	cLogP (DW)	PSA[b] (DW)	Caco2 cLog Papp (ES)	BBB[c] cLog Papp (ES)
**12**	3	4	3.1	94	0.57	0.54
**18**	4	4	4	103	0.32	0.20
**22**	5	4	2.8	116	0.45	0.19
**41**	5	4	3.8	116	0.40	0.18
**42**	5	4	3.2	113	0.17	0.20
**43**	5	5	2.9	146	0.03	0.17

[a]in silico values calculated by DW: DataWarrior™ ; or ES: Enalos Suite™; [b] Polar surface area in Å^2 ; [c] BBB : Blood Brain Barrier.

Supplementary Methods

Caco-2 clogPapp and BBB clogPapp (EnalosSuite®)

Caco-2 clogPapp and BBB clogPapp were calculated using the EnalosSuite® from Novamechanics https://enalossuite.novamechanics.com/. The predictive models combine contrastive learning (CL) and atom-attention message-passing neural networks (MPNN). With CL, the models yielded significant improvements in predictive accuracy, particularly for BBB permeability and human intestinal permeability prediction. By pretraining the atom-attention MPNN on a large, unlabeled dataset, the model has been able to learn robust and comprehensive molecular representations. To train the predictive model for the desired properties, a comprehensive dataset of 7,807 compounds, categorized based on their BBB permeability (BBB+ or BBB-), was compiled from the literature[†]. A significant literature dataset[‡] was utilized to train the Caco-2 cell line permeability predictive model. Any compounds with unclear SMILES codes or permeability values outside the range of $10^{-3.5}\ cm \cdot s^{-1}$ to $10^{-8}\ cm\ \cdot s^{-1}$, which are considered potential unreliable[§], were excluded. The remaining compounds were then processed using the k-Means clustering algorithm to categorize them into two groups: permeable and non-permeable, based on their permeability values.[**] Compounds with permeability values less than or equal to $10^{-5.5}\ cm \cdot s^{-1}$ were classified as non-permeable, while those with values greater than $10^{-5.5}\ cm \cdot s^{-1}$ were considered permeable. The final modeling dataset comprised 1,827 compounds, of which 1,127 (62%) were classified as permeable.

cLogP and PSA (OSIRIS Datawarrior®)

The partition coefficient between n-octanol and water log(octanol/water) (cLogP) and the polar surface area (PSA) were calculated using Datawarrior®,[††] with protocols described in the DataWarrior User Manual accessible at https://openmolecules.org/properties/properties.html.

[†] F. Meng, Y. Xi, J. Huang, and P. W. Ayers, "A curated diverse molecular database of blood-brain barrier permeability with chemical descriptors," *Sci. Data*, vol. 8, no. 1, p. 289, Oct. **2021**.
[‡] Y. Wang and X. Chen, "QSPR model for Caco-2 cell permeability prediction using a combination of HQPSO and dual-RBF neural network," *RSC Adv.*, vol. 10, no. 70, pp. 42938–42952, 2020, doi: 10.1039/D0RA08209K.
[§] N.-N. Wang *et al.*, "ADME Properties Evaluation in Drug Discovery: Prediction of Caco-2 Cell Permeability Using a Combination of NSGA-II and Boosting," *J. Chem. Inf. Model.*, vol. 56, no. 4, pp. 763–773, Apr. 2016, doi: 10.1021/acs.jcim.5b00642.
[**] J. MacQueen, "Some methods for classification and analysis of multivariate observations.," in *In Proceedings of the Fifth Berkeley Symposium on Mathematical Statistics and Probability*, vol. 1, Oakland, CA, USA, pp. 281–297.
[††] T.Sander, J. Freyss, M. von Korff, C. Rufener. DataWarrior: An Open-Source Program For Chemistry Aware Data Visualization And Analysis. *J Chem Inf Model* **2015**, *55*, 460-473.

HPLC chromatograms of inhibitors 12, 18, 22, 41,43.

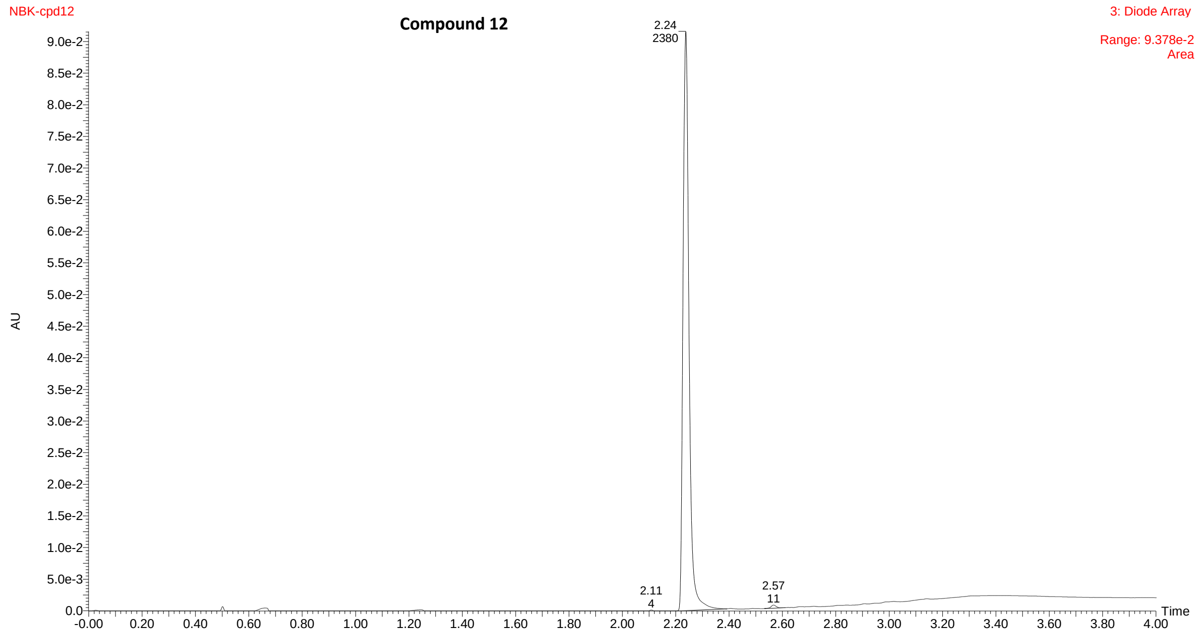

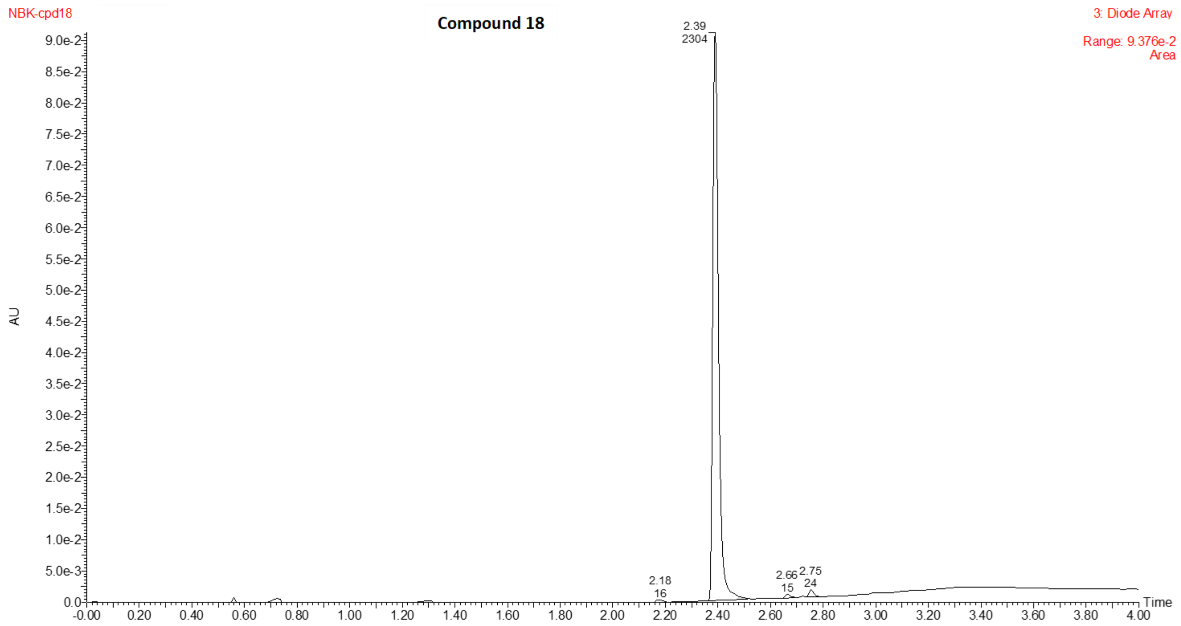

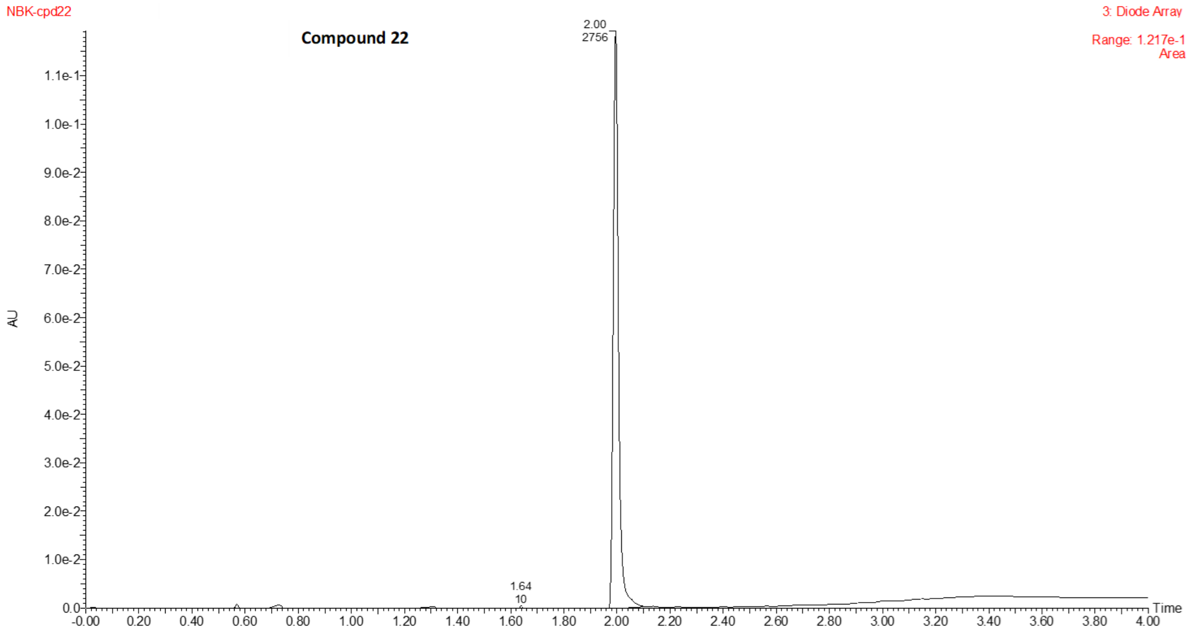
NBK-cpd22
Compound 22
3: Diode Array
Range: 1.217e-1
Area
2.00
2756
1.64
10
AU
Time

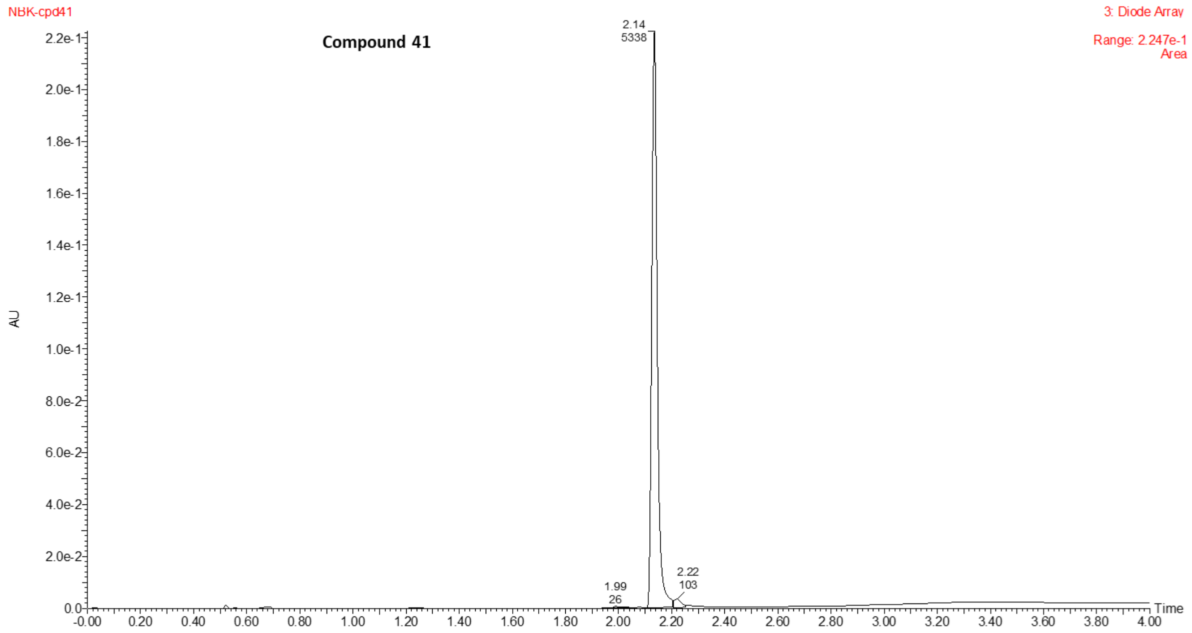
NBK-cpd41
Compound 41
3: Diode Array
Range: 2.247e-1
Area
2.14
5338
1.99
26
2.22
103
AU
Time

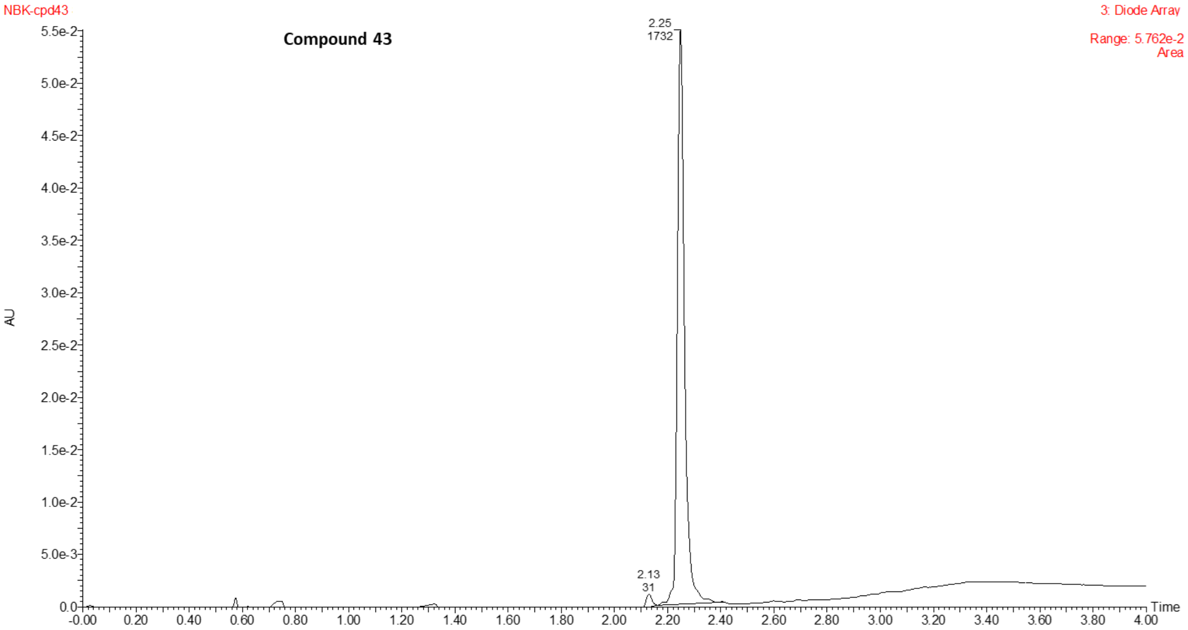
NBK-cpd43
Compound 43
3: Diode Array
Range: 5.762e-2
Area
2.25
1732
2.13
31
AU
Time

NMR spectra for final compounds

Compound 1

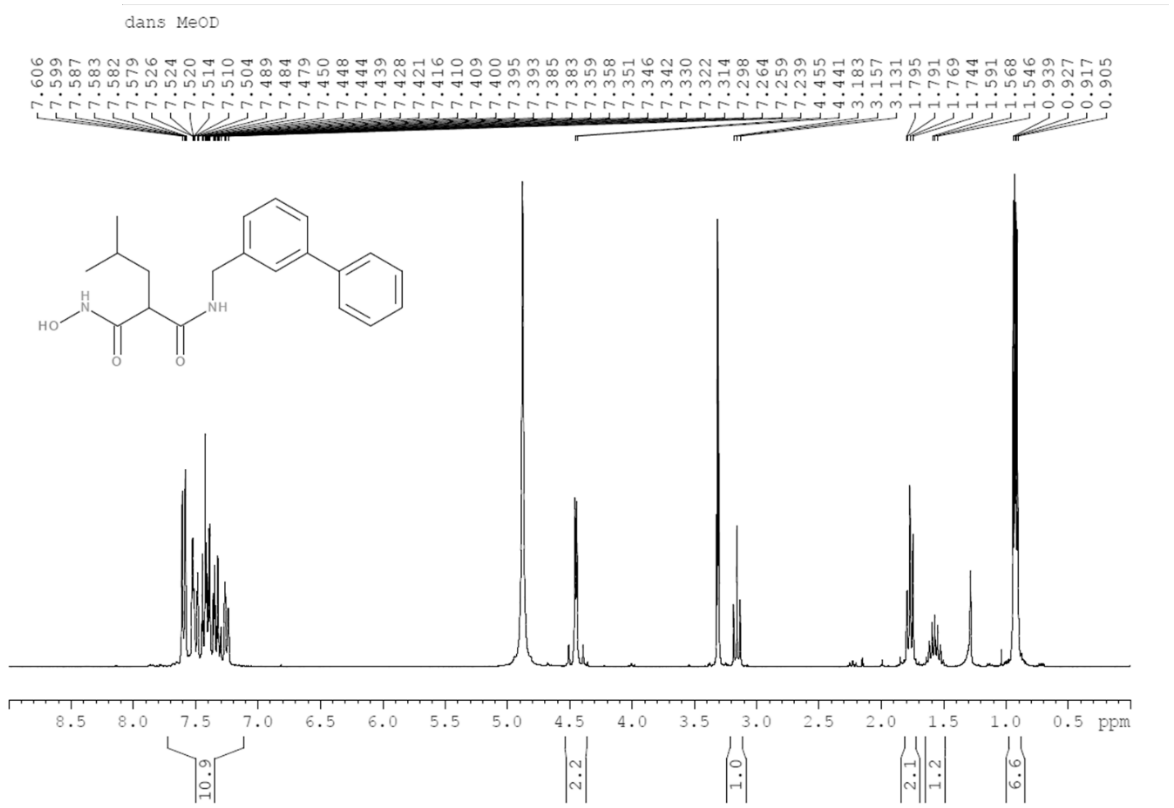

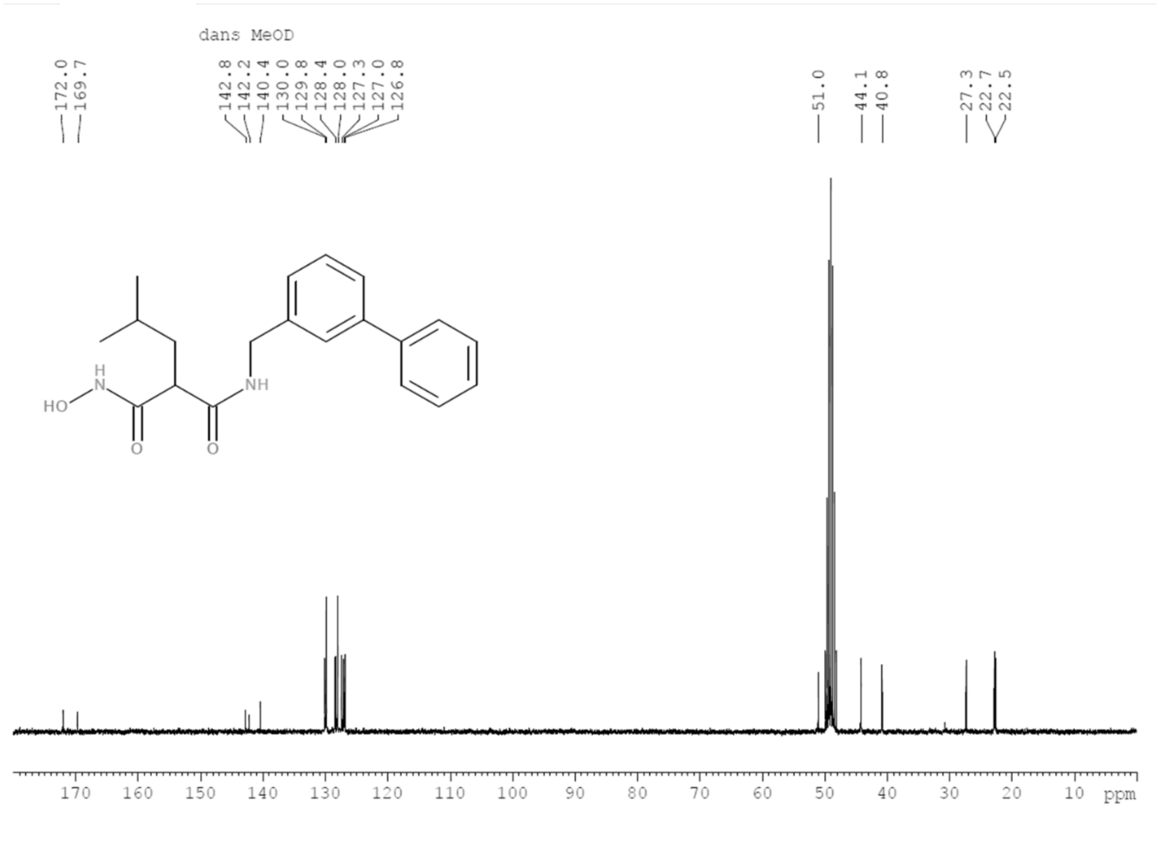

Compound 2

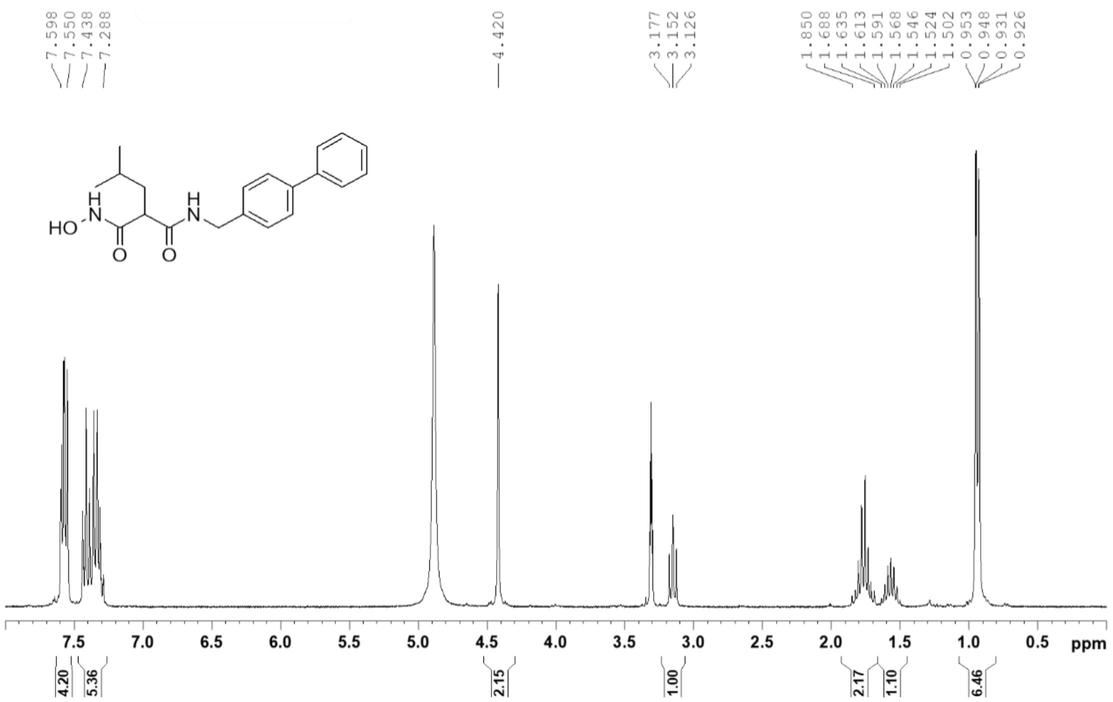

7.598
7.550
7.438
7.288
4.420
3.177
3.152
3.126
1.850
1.688
1.635
1.613
1.591
1.568
1.546
1.524
1.502
0.953
0.948
0.931
0.926
HO
N
H
O
O
N
H
ppm
4.20
5.36
2.15
1.00
2.17
1.10
6.46

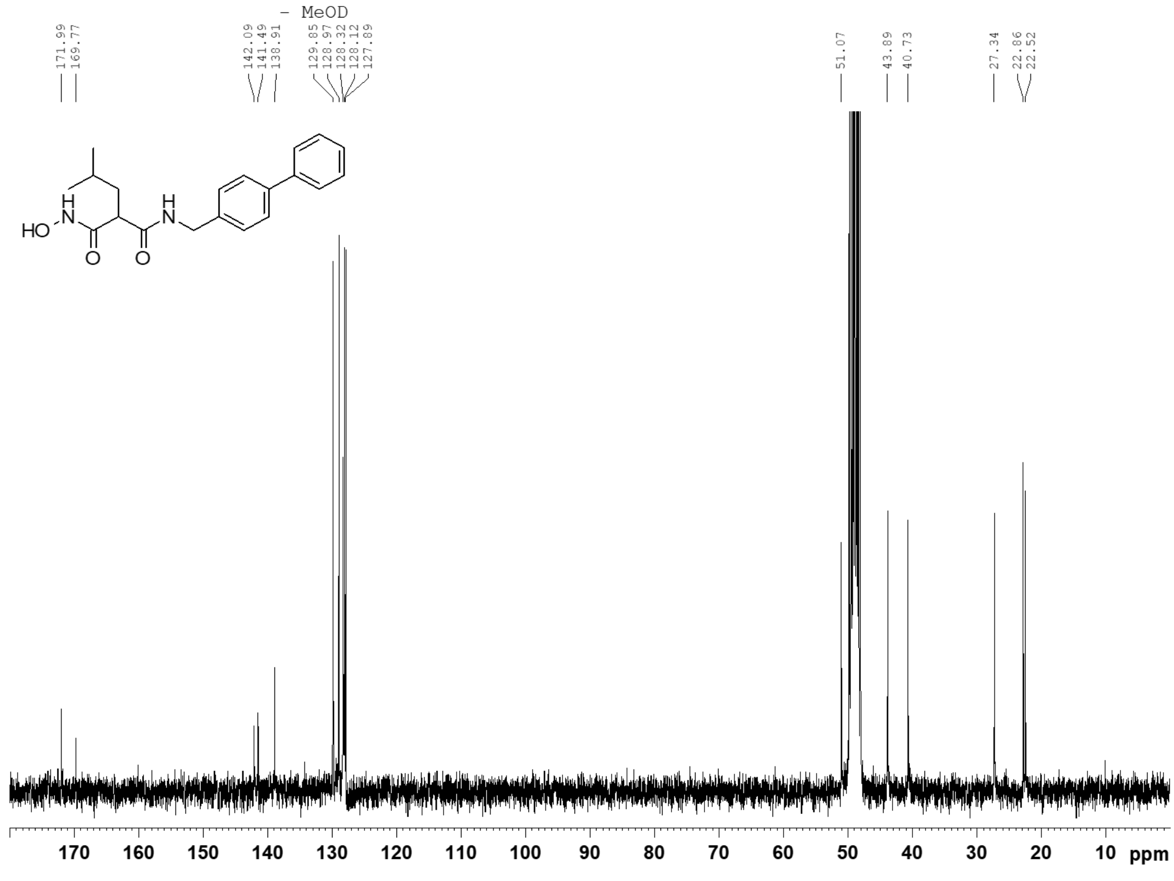

MeOD
171.99
169.77
142.09
141.49
138.91
129.85
128.97
128.32
128.12
127.89
51.07
43.89
40.73
27.34
22.86
22.52
HO
N
H
O
O
N
H
ppm

Compound 3

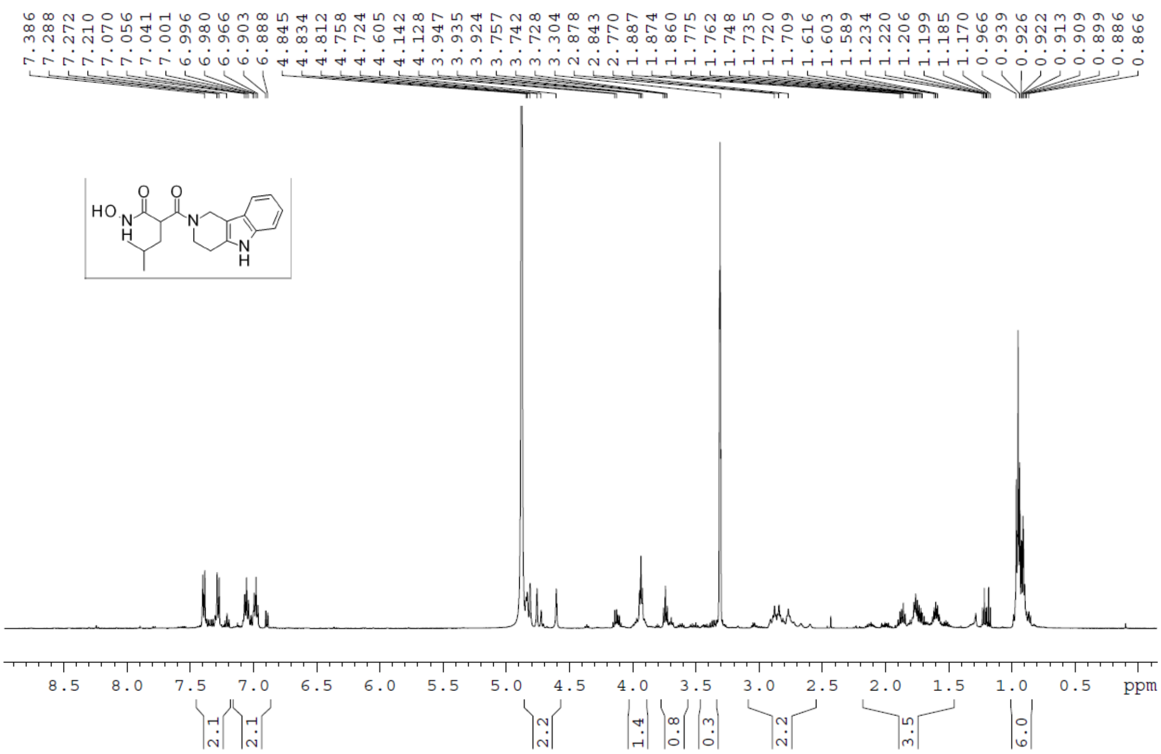
7.386 7.288 7.272 7.210 7.070 7.056 7.041 7.001 6.996 6.980 6.966 6.903 6.888 4.845 4.834 4.812 4.758 4.724 4.605 4.142 4.128 3.947 3.935 3.924 3.757 3.742 3.728 3.304 2.878 2.843 2.770 1.887 1.874 1.860 1.775 1.762 1.748 1.735 1.720 1.709 1.616 1.603 1.589 1.234 1.220 1.206 1.199 1.185 1.170 0.966 0.939 0.926 0.922 0.913 0.909 0.899 0.886 0.866
HO
N
H
O
O
N
N
H
8.5 8.0 7.5 7.0 6.5 6.0 5.5 5.0 4.5 4.0 3.5 3.0 2.5 2.0 1.5 1.0 0.5 ppm
2.1 2.1 2.2 1.4 0.8 0.3 2.2 3.5 6.0

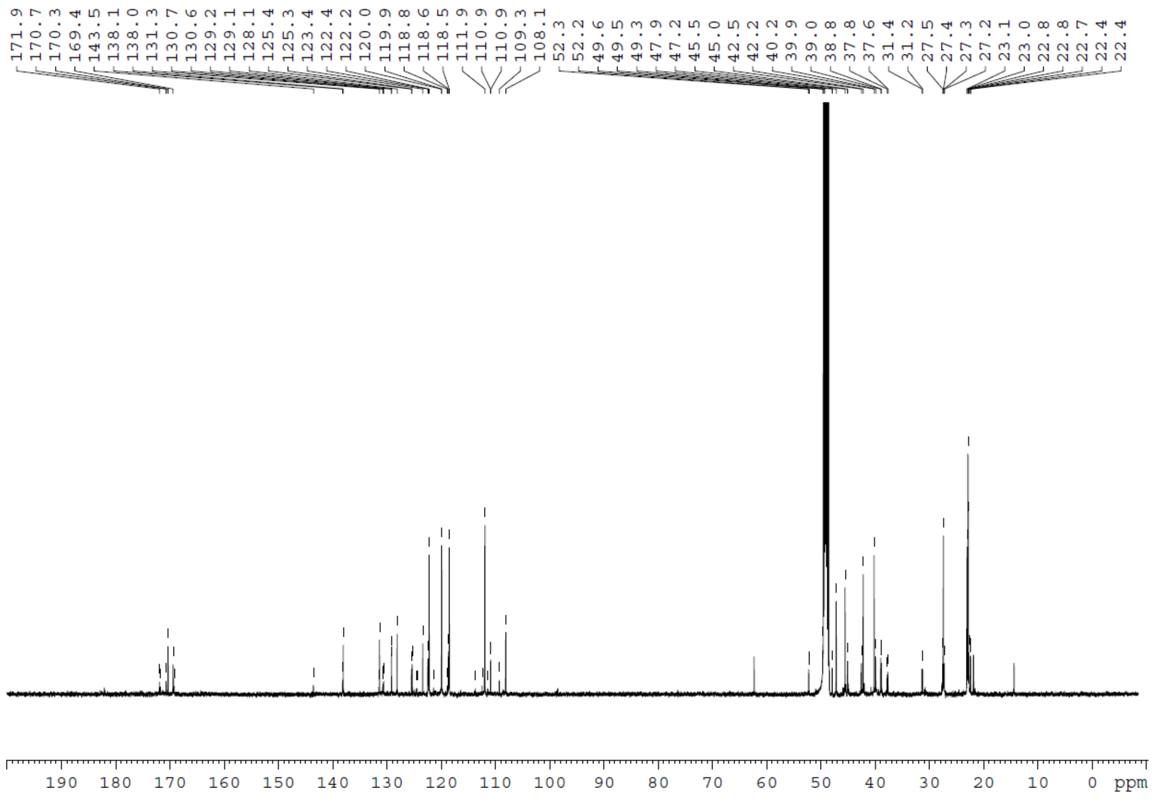
171.9 170.7 170.3 169.4 143.5 138.1 138.0 131.3 130.7 130.6 129.2 129.1 128.1 125.4 125.3 123.4 122.4 122.2 120.0 119.9 118.8 118.6 118.5 111.9 110.9 110.9 109.3 108.1 52.3 52.2 49.6 49.5 49.3 47.9 47.2 45.5 45.0 42.5 42.2 40.2 39.9 39.0 38.8 37.8 37.6 31.4 31.2 27.5 27.4 27.3 27.2 23.1 23.0 22.8 22.8 22.7 22.4 22.4
190 180 170 160 150 140 130 120 110 100 90 80 70 60 50 40 30 20 10 0 ppm

Compound 4

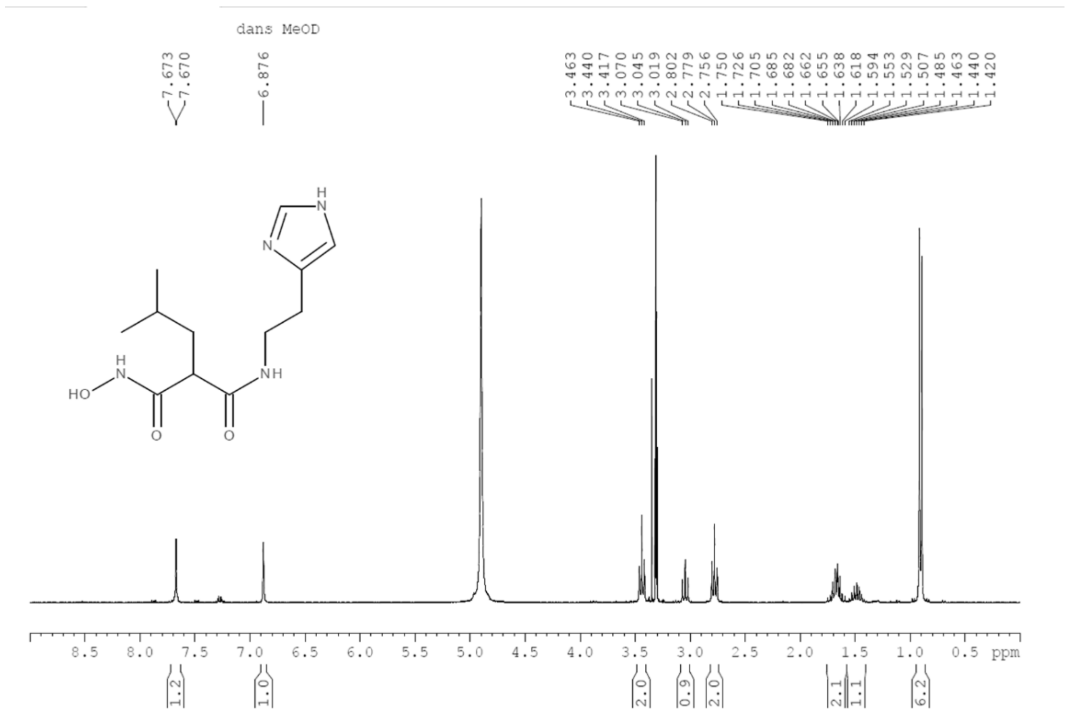
dans MeOD
7.673
7.670
6.876
3.463
3.440
3.417
3.070
3.045
3.019
2.802
2.779
2.756
1.750
1.726
1.705
1.685
1.682
1.662
1.655
1.638
1.618
1.594
1.553
1.529
1.507
1.485
1.463
1.440
1.420
HO
H
N
O
O
NH
N
H
N
8.5 8.0 7.5 7.0 6.5 6.0 5.5 5.0 4.5 4.0 3.5 3.0 2.5 2.0 1.5 1.0 0.5 ppm
1.2
1.0
2.0
0.9
2.0
2.1
1.1
6.2

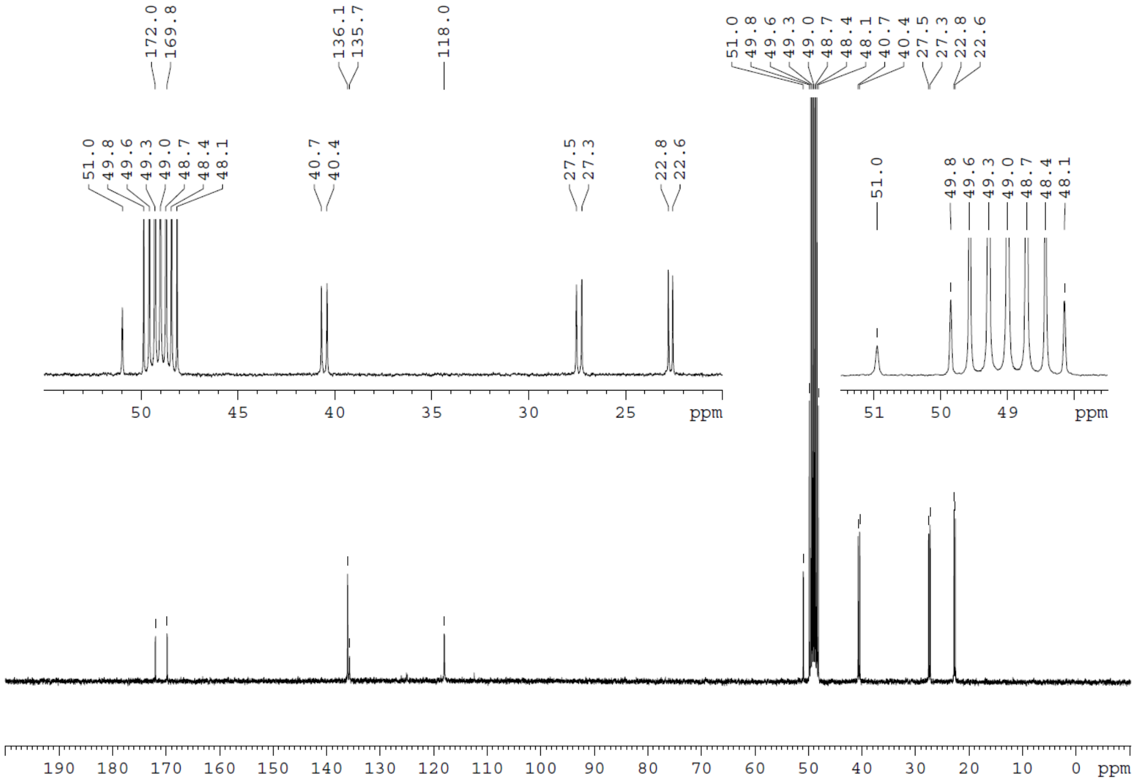
172.0
169.8
136.1
135.7
118.0
51.0
49.8
49.6
49.3
49.0
48.7
48.4
48.1
40.7
40.4
27.5
27.3
22.8
22.6
50 45 40 35 30 25 ppm
51 50 49 ppm
190 180 170 160 150 140 130 120 110 100 90 80 70 60 50 40 30 20 10 0 ppm

Compound 5

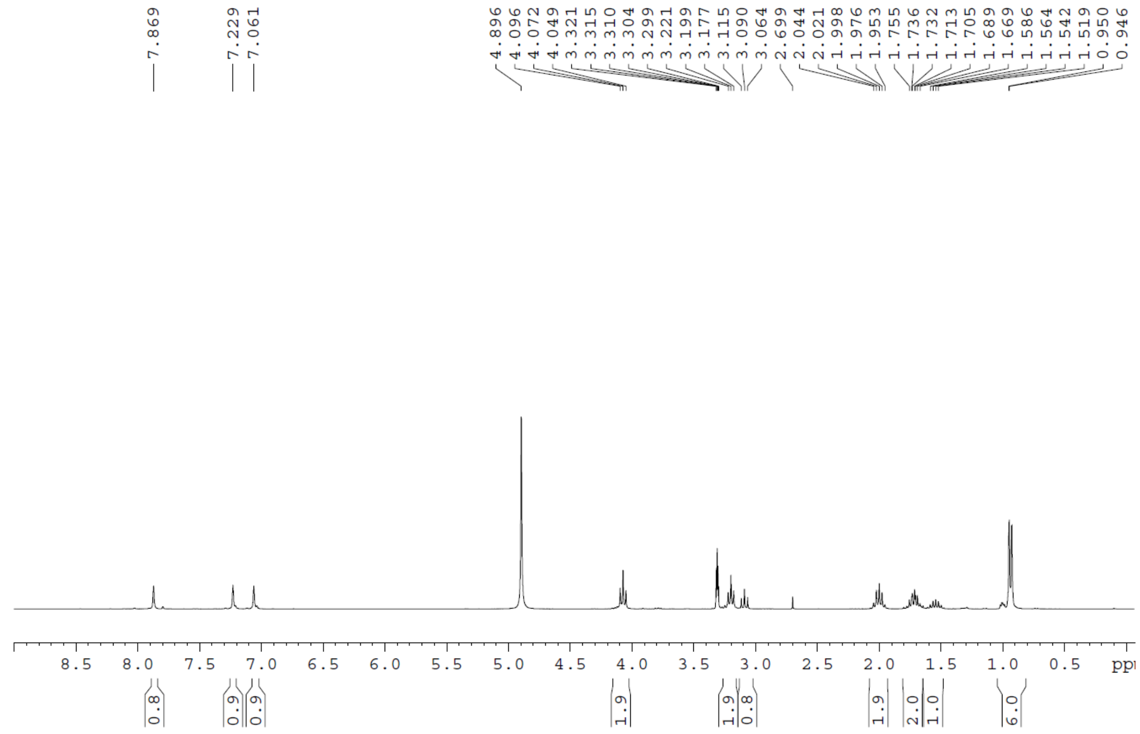

7.869
7.229
7.061
4.896
4.096
4.072
4.049
3.321
3.315
3.310
3.304
3.299
3.221
3.199
3.177
3.115
3.090
3.064
2.699
2.044
2.021
1.998
1.976
1.953
1.755
1.736
1.732
1.713
1.705
1.689
1.669
1.586
1.564
1.542
1.519
0.950
0.946
8.5 8.0 7.5 7.0 6.5 6.0 5.5 5.0 4.5 4.0 3.5 3.0 2.5 2.0 1.5 1.0 0.5 ppm
0.8
0.9
0.9
1.9
1.9
0.8
1.9
2.0
1.0
6.0

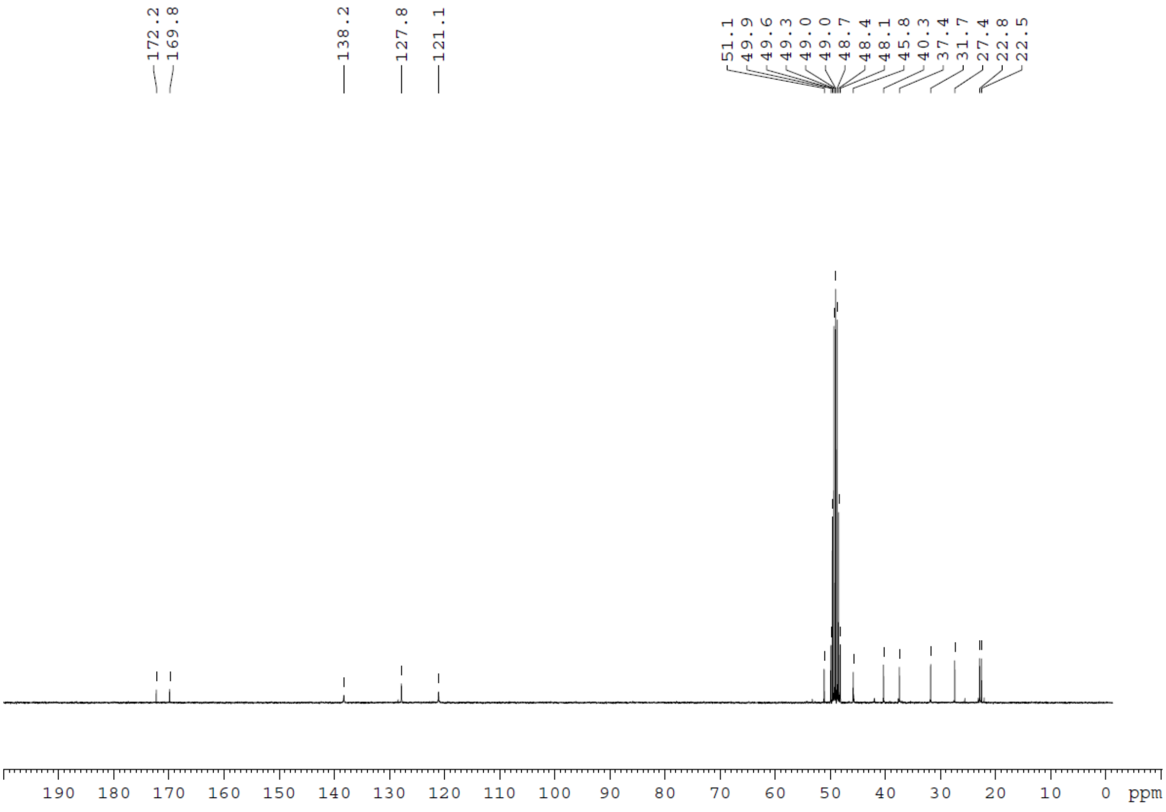

172.2
169.8
138.2
127.8
121.1
51.1
49.9
49.6
49.3
49.0
49.0
48.7
48.4
48.1
45.8
40.3
37.4
31.7
27.4
22.8
22.5
190 180 170 160 150 140 130 120 110 100 90 80 70 60 50 40 30 20 10 0 ppm

Compound 6

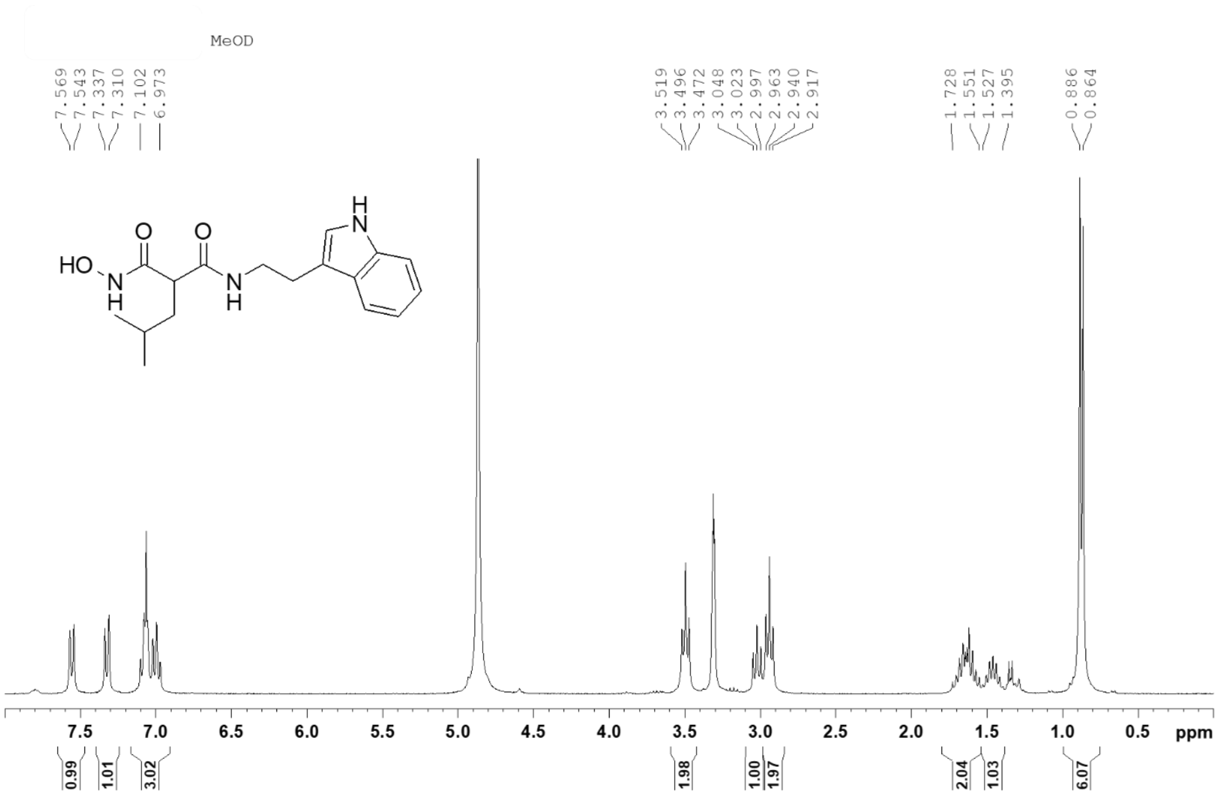
MeOD
7.569
7.543
7.337
7.310
7.102
6.973
3.519
3.496
3.472
3.048
3.023
2.997
2.963
2.940
2.917
1.728
1.551
1.527
1.395
0.886
0.864
HO
N
H
O
O
N
H
H
N
7.5
7.0
6.5
6.0
5.5
5.0
4.5
4.0
3.5
3.0
2.5
2.0
1.5
1.0
0.5
ppm
0.99
1.01
3.02
1.98
1.00
1.97
2.04
1.03
6.07

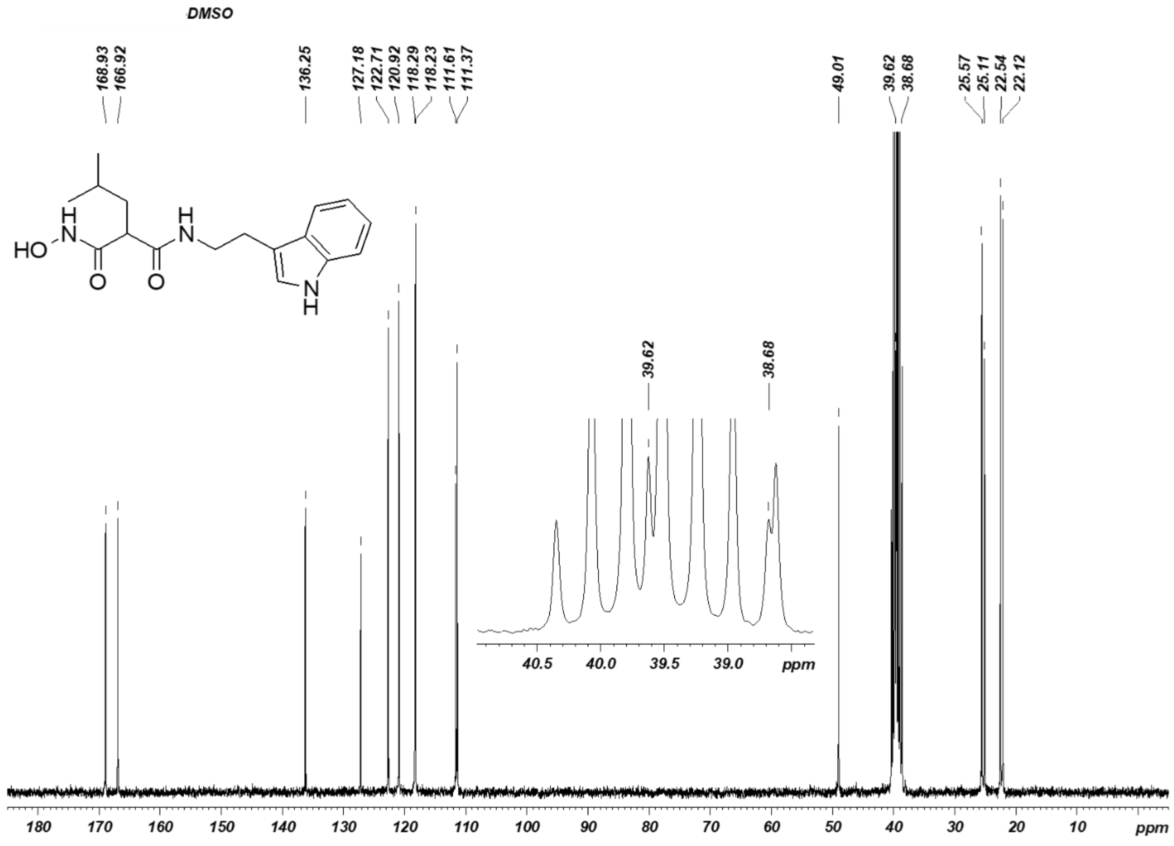
DMSO
168.93
166.92
136.25
127.18
122.71
120.92
118.29
118.23
111.61
111.37
49.01
39.62
38.68
25.57
25.11
22.54
22.12
HO
N
H
O
O
N
H
N
H
39.62
38.68
40.5
40.0
39.5
39.0
ppm
180
170
160
150
140
130
120
110
100
90
80
70
60
50
40
30
20
10
ppm

Compound 7

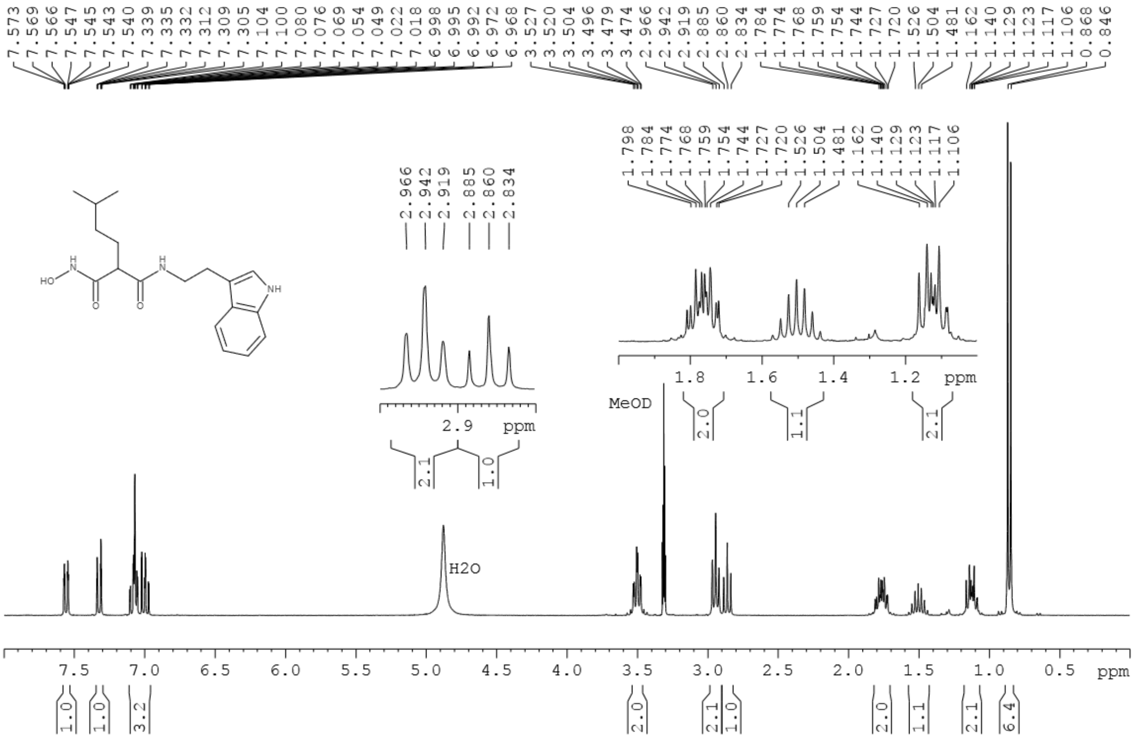
7.573 7.569 7.566 7.547 7.545 7.543 7.540 7.339 7.335 7.332 7.312 7.309 7.305 7.104 7.100 7.080 7.076 7.069 7.054 7.049 7.022 7.018 6.998 6.995 6.992 6.972 6.968 3.527 3.520 3.504 3.496 3.479 3.474 2.966 2.942 2.919 2.885 2.860 2.834 1.784 1.774 1.768 1.759 1.754 1.744 1.727 1.720 1.526 1.504 1.481 1.162 1.140 1.129 1.123 1.117 1.106 0.868 0.846
2.966 2.942 2.919 2.885 2.860 2.834
1.798 1.784 1.774 1.768 1.759 1.754 1.744 1.727 1.720 1.526 1.504 1.481 1.162 1.140 1.129 1.123 1.117 1.106
MeOD
H2O
2.9 ppm
1.8 1.6 1.4 1.2 ppm
7.5 7.0 6.5 6.0 5.5 5.0 4.5 4.0 3.5 3.0 2.5 2.0 1.5 1.0 0.5 ppm
1.0 1.0 3.2 2.0 2.1 1.0 2.0 1.1 2.1 6.4

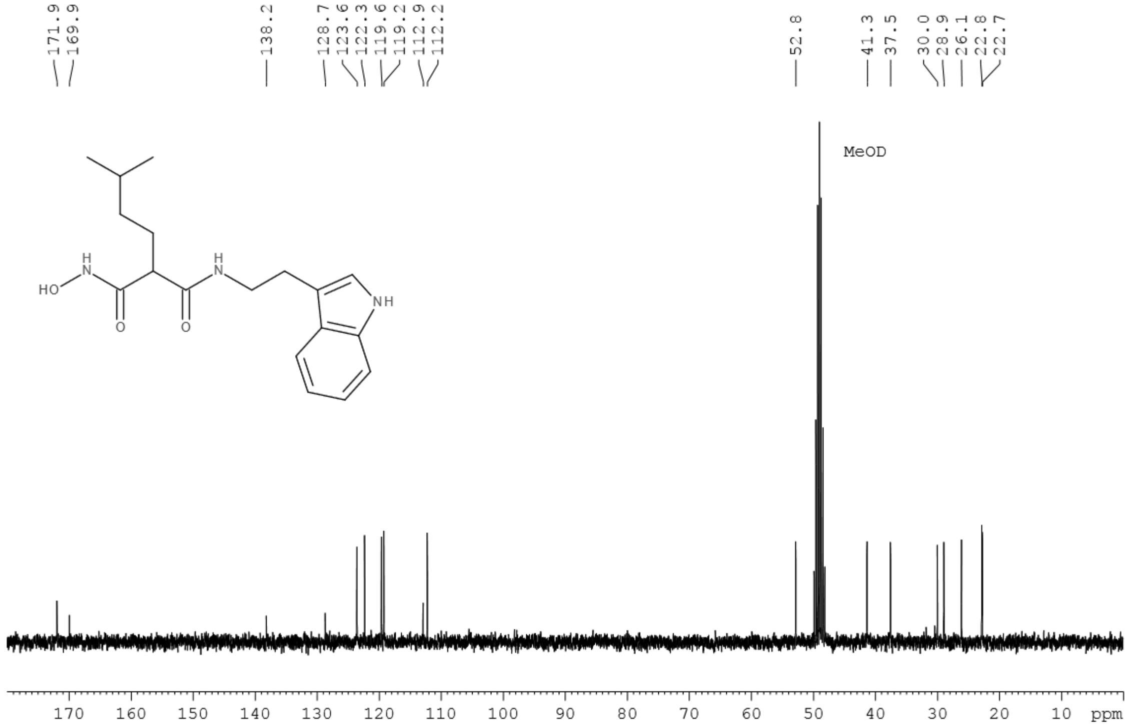
171.9 169.9 138.2 128.7 123.6 122.3 119.6 119.2 112.9 112.2 52.8 41.3 37.5 30.0 28.9 26.1 22.8 22.7
MeOD
170 160 150 140 130 120 110 100 90 80 70 60 50 40 30 20 10 ppm

Compound 8

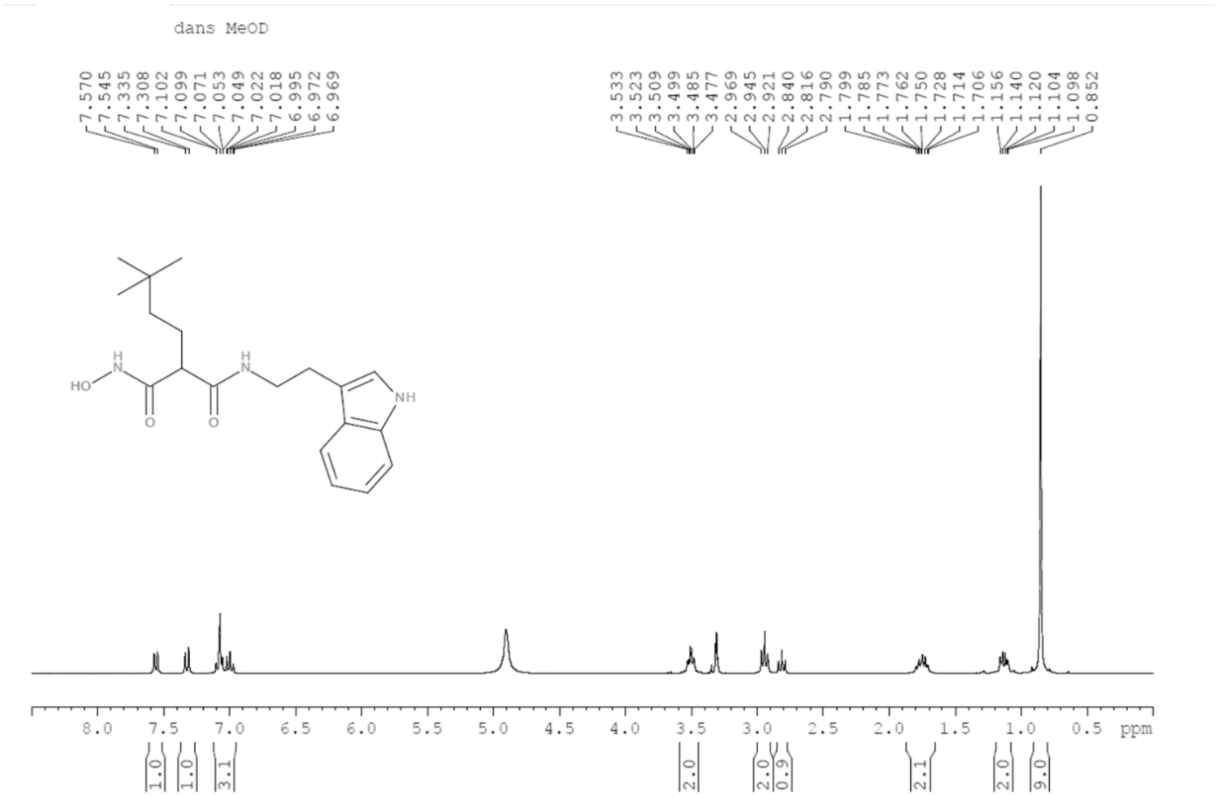
dans MeOD
7.570
7.545
7.335
7.308
7.102
7.099
7.071
7.053
7.049
7.022
7.018
6.995
6.972
6.969
3.533
3.523
3.509
3.499
3.485
3.477
2.969
2.945
2.921
2.840
2.816
2.790
1.799
1.785
1.773
1.762
1.750
1.728
1.714
1.706
1.156
1.140
1.120
1.104
1.098
0.852
HO
H
N
H
N
O
O
NH
8.0
7.5
7.0
6.5
6.0
5.5
5.0
4.5
4.0
3.5
3.0
2.5
2.0
1.5
1.0
0.5
ppm
1.0
1.0
3.1
2.0
2.0
0.9
2.1
2.0
9.0

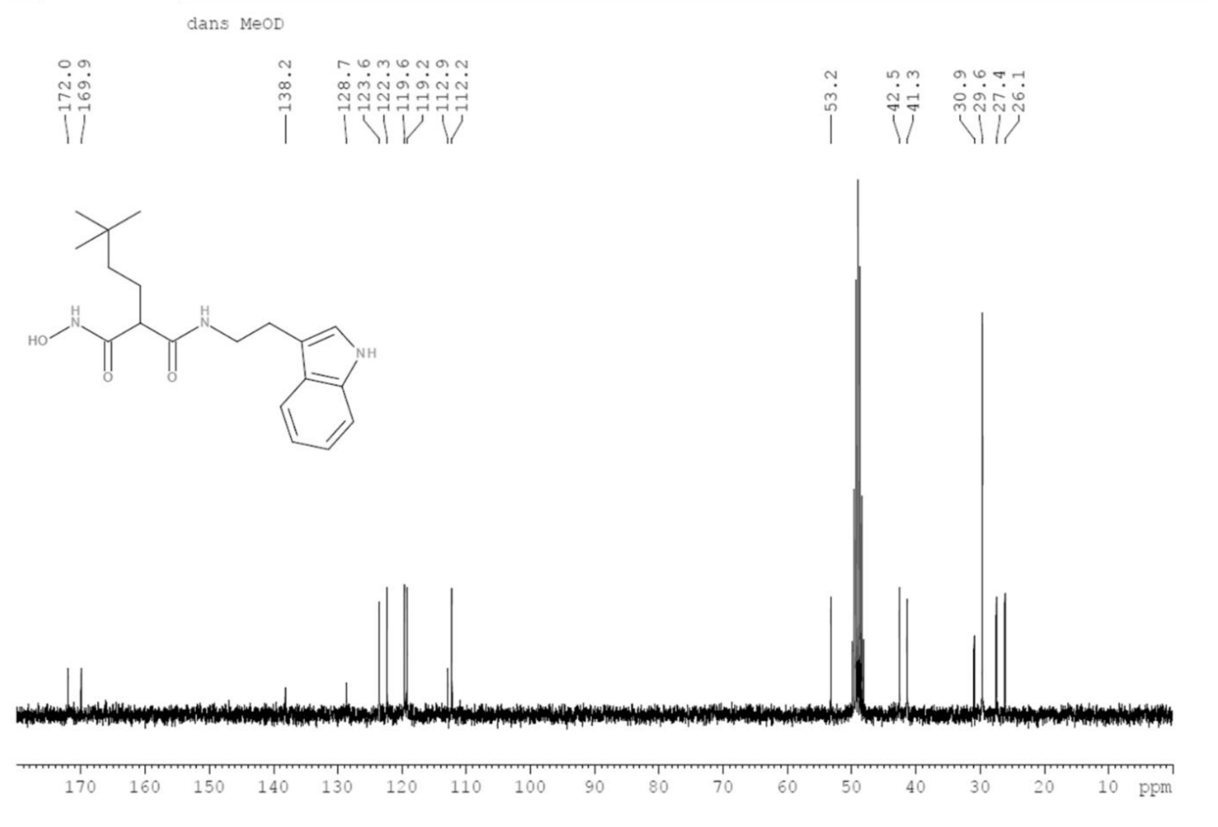
dans MeOD
172.0
169.9
138.2
128.7
123.6
122.3
119.6
119.2
112.9
112.2
53.2
42.5
41.3
30.9
29.6
27.4
26.1
HO
H
N
H
N
O
O
NH
170
160
150
140
130
120
110
100
90
80
70
60
50
40
30
20
10
ppm

Compound 9

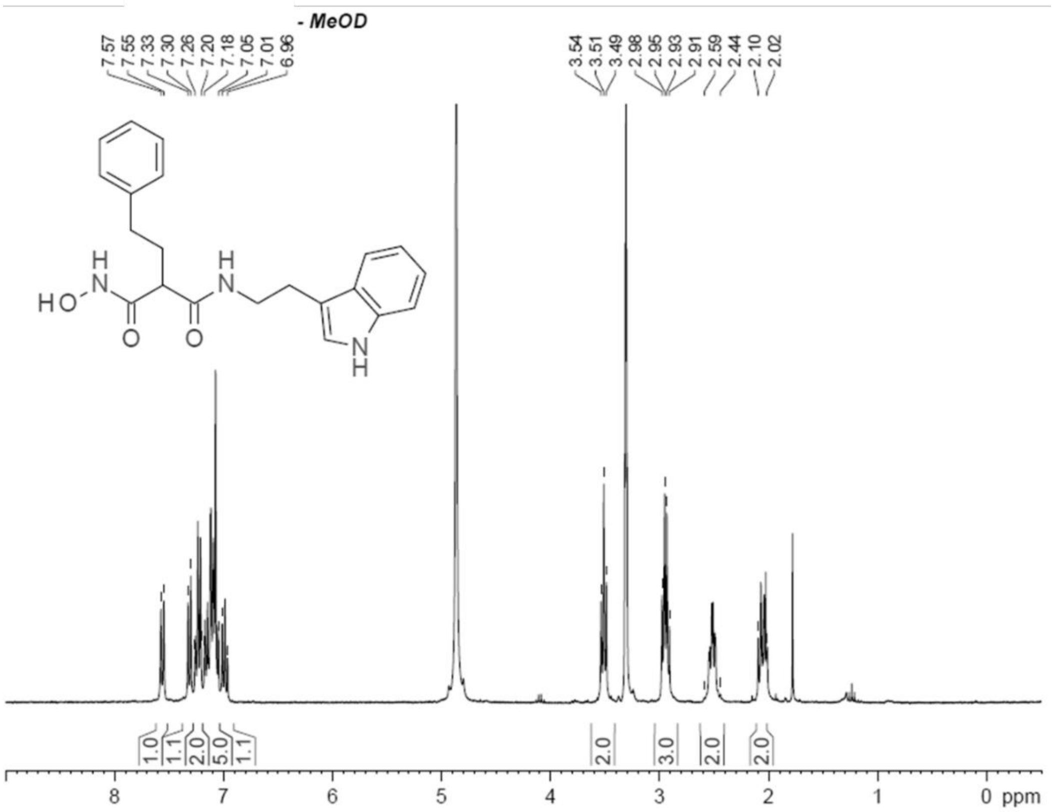
- MeOD
7.57
7.55
7.33
7.30
7.26
7.20
7.18
7.05
7.01
6.96
3.54
3.51
3.49
2.98
2.95
2.93
2.91
2.59
2.44
2.10
2.02
1.0
1.1
2.0
5.0
1.1
2.0
3.0
2.0
2.0
8
7
6
5
4
3
2
1
0 ppm

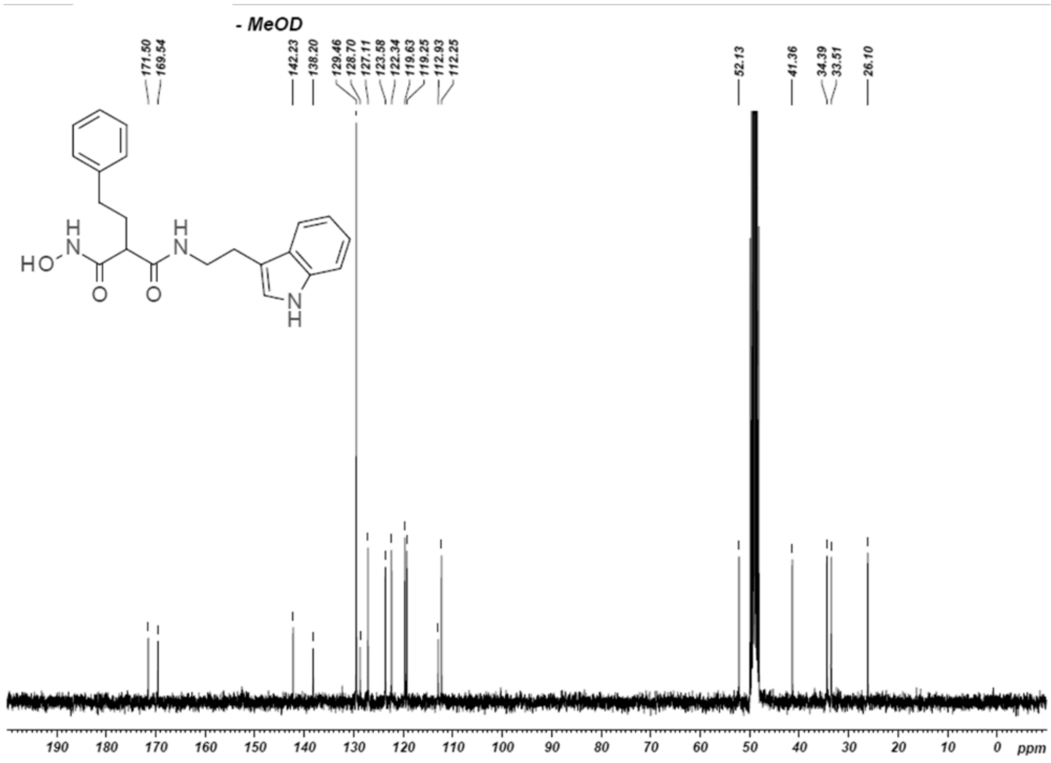
- MeOD
171.50
169.54
142.23
138.20
129.46
128.70
127.11
123.58
122.34
119.63
119.25
112.93
112.25
52.13
41.36
34.39
33.51
26.10
190
180
170
160
150
140
130
120
110
100
90
80
70
60
50
40
30
20
10
0
ppm

Compound 10

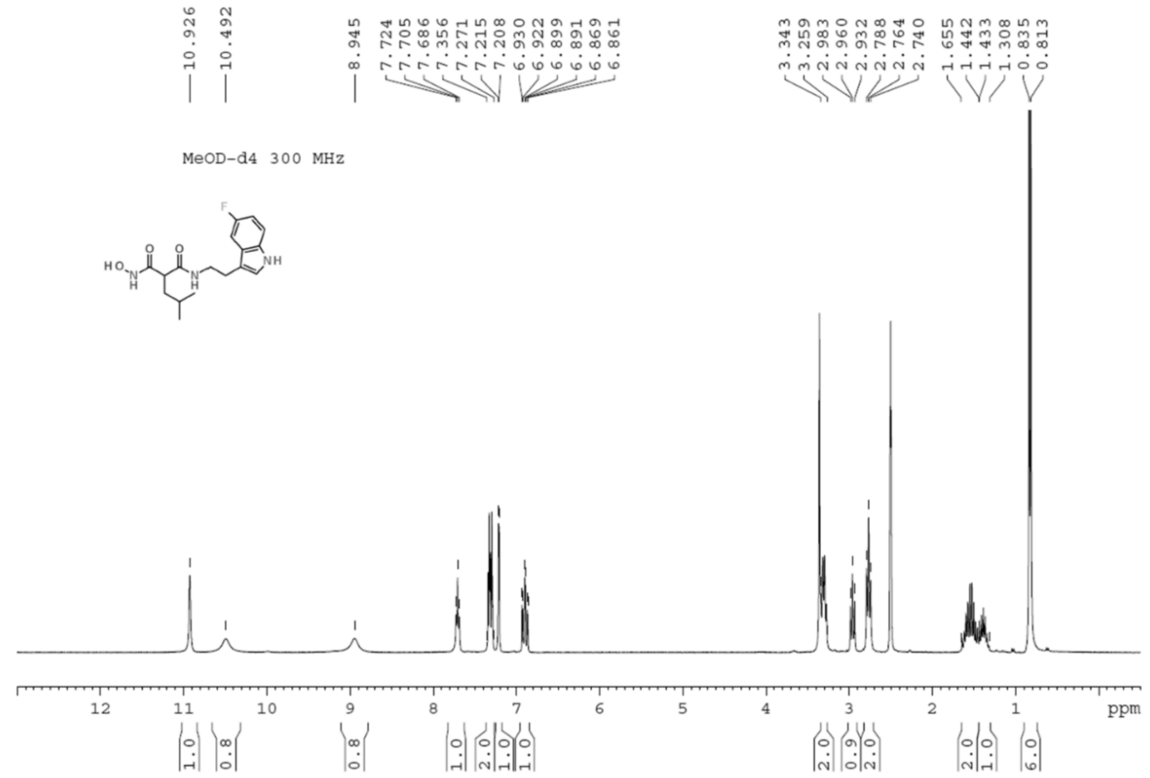
10.926
10.492
8.945
7.724
7.705
7.686
7.356
7.271
7.215
7.208
6.930
6.922
6.899
6.891
6.869
6.861
3.343
3.259
2.983
2.960
2.932
2.788
2.764
2.740
1.655
1.442
1.433
1.308
0.835
0.813
MeOD-d4 300 MHz
ppm

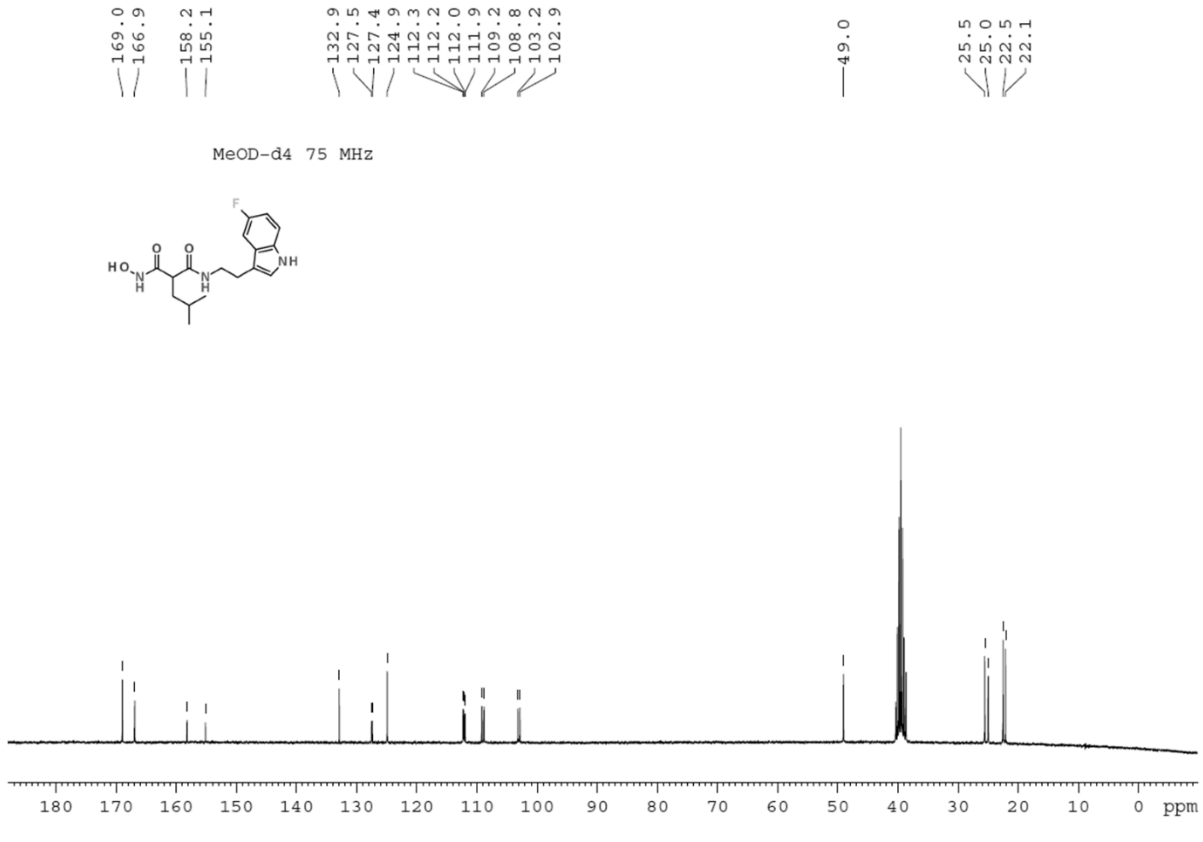
169.0
166.9
158.2
155.1
132.9
127.5
127.4
124.9
112.3
112.2
112.0
111.9
109.2
108.8
103.2
102.9
49.0
25.5
25.0
22.5
22.1
MeOD-d4 75 MHz
ppm

Compound 11

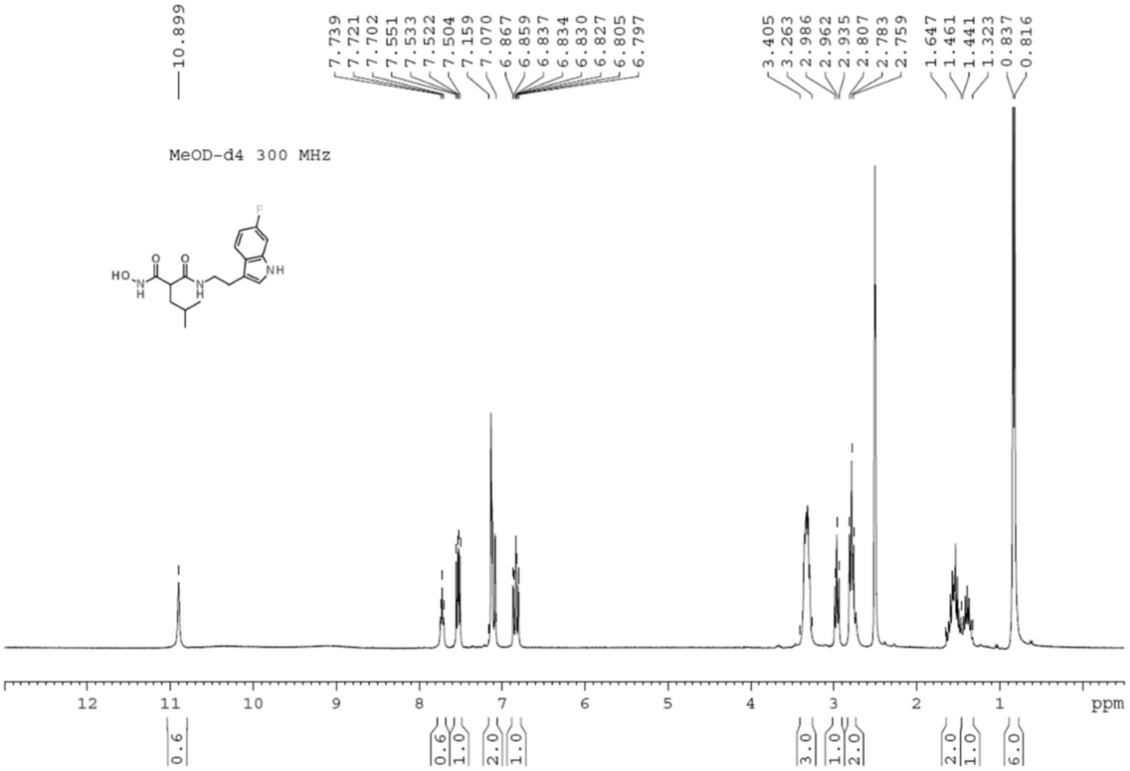

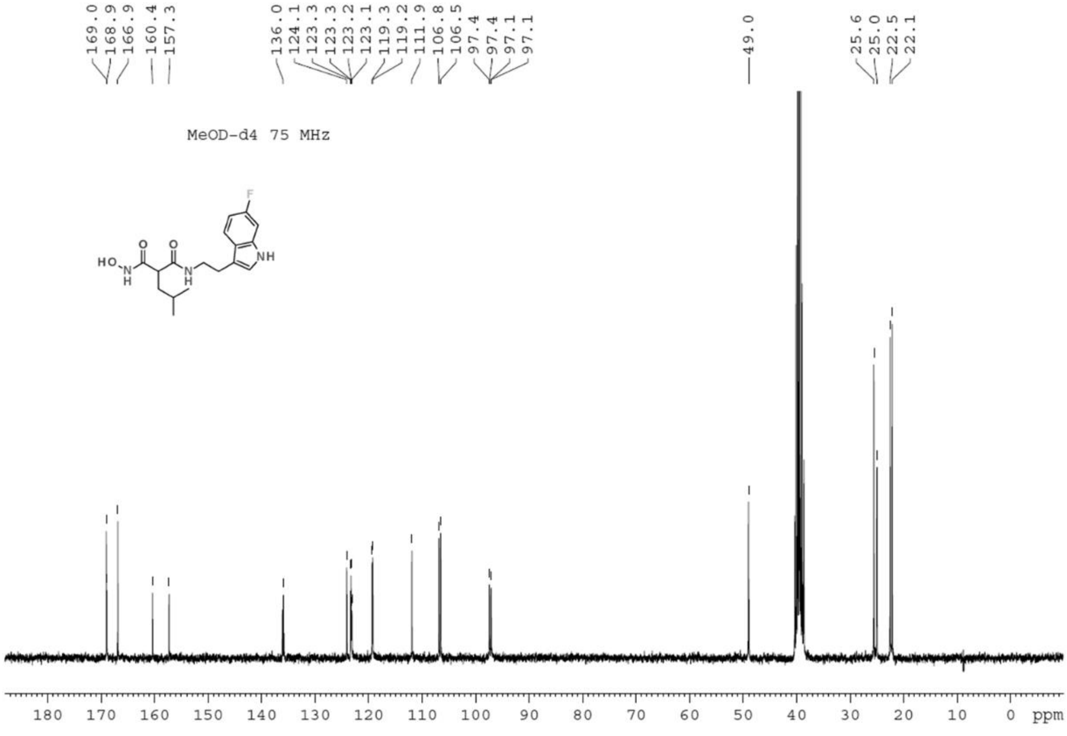

Compound 12

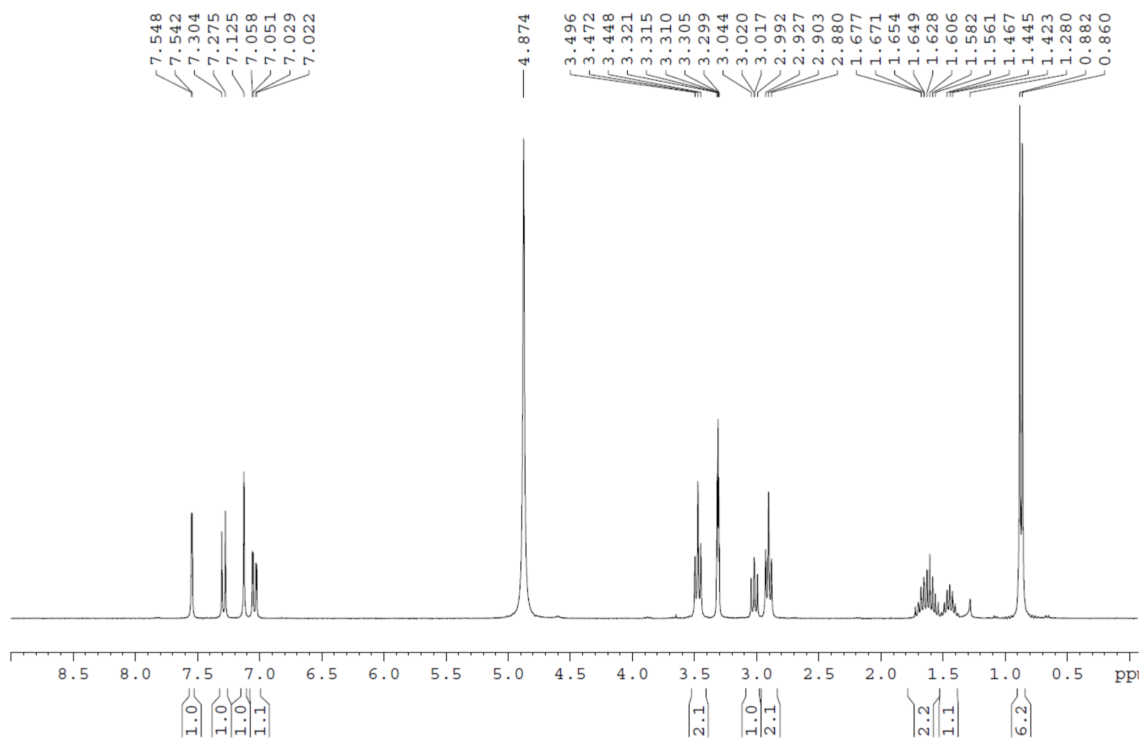
7.548
7.542
7.304
7.275
7.125
7.058
7.051
7.029
7.022
4.874
3.496
3.472
3.448
3.321
3.315
3.310
3.305
3.299
3.044
3.020
3.017
2.992
2.927
2.903
2.880
1.677
1.671
1.654
1.649
1.628
1.606
1.582
1.561
1.467
1.445
1.423
1.280
0.882
0.860
8.5 8.0 7.5 7.0 6.5 6.0 5.5 5.0 4.5 4.0 3.5 3.0 2.5 2.0 1.5 1.0 0.5 ppm
1.0
1.0
1.0
1.1
2.1
1.0
2.1
2.2
1.1
6.2

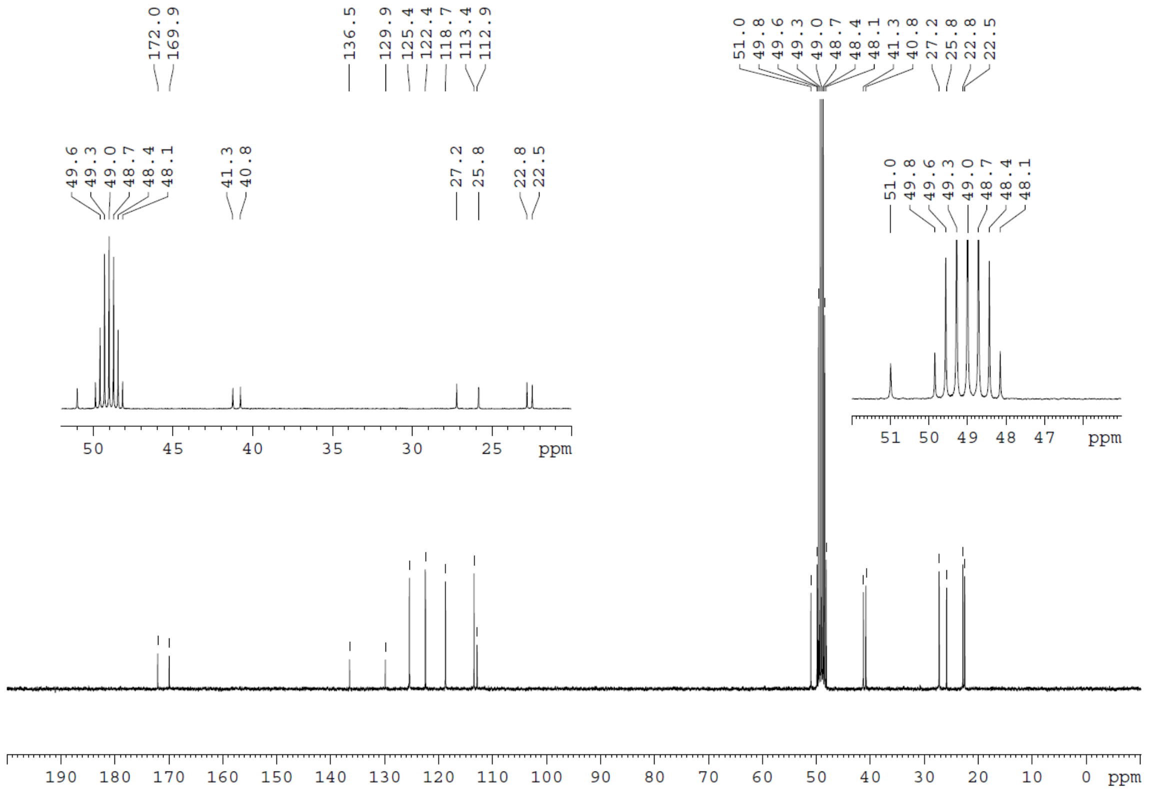
172.0
169.9
136.5
129.9
125.4
122.4
118.7
113.4
112.9
51.0
49.8
49.6
49.3
49.0
48.7
48.4
48.1
41.3
40.8
27.2
25.8
22.8
22.5
190 180 170 160 150 140 130 120 110 100 90 80 70 60 50 40 30 20 10 0 ppm
50 45 40 35 30 25 ppm
51 50 49 48 47 ppm

Compound 13

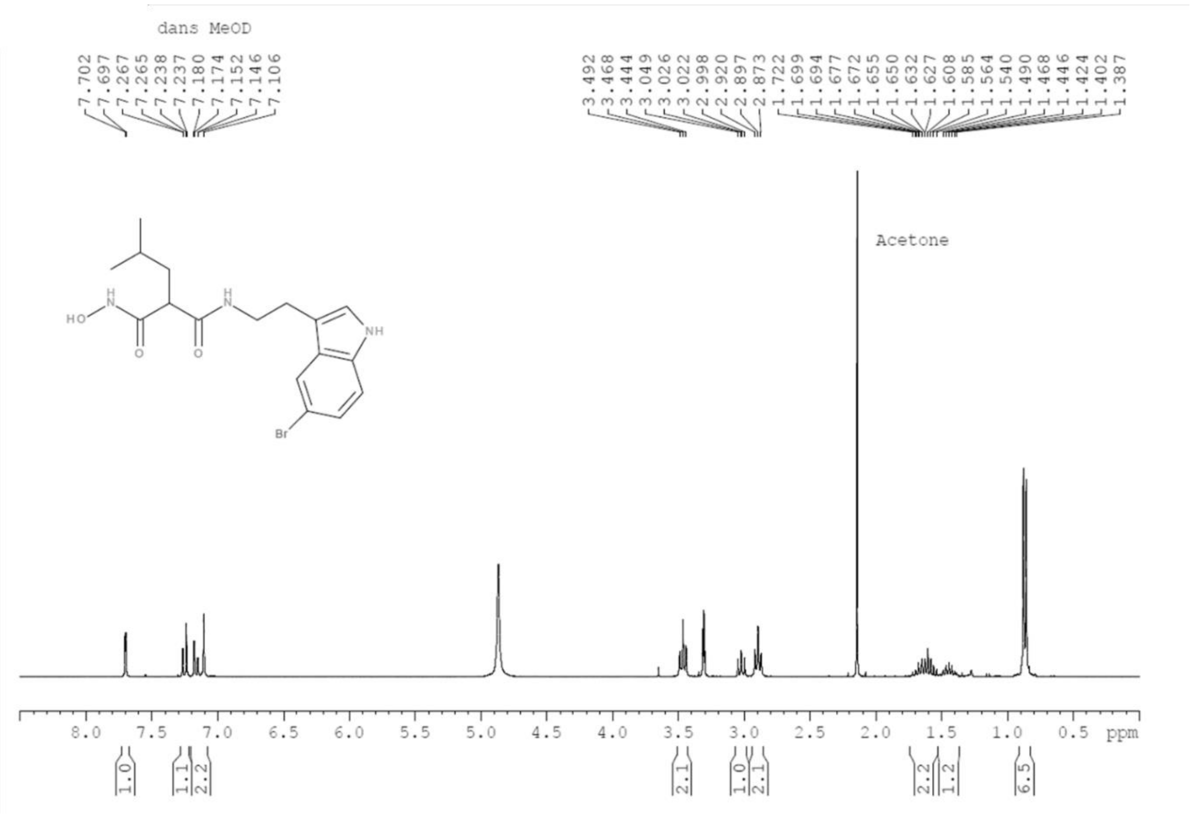
dans MeOD
7.702
7.697
7.267
7.265
7.238
7.237
7.180
7.174
7.152
7.146
7.106
3.492
3.468
3.444
3.049
3.026
3.022
2.998
2.920
2.897
2.873
1.722
1.699
1.694
1.677
1.672
1.655
1.650
1.632
1.627
1.608
1.585
1.564
1.540
1.490
1.468
1.446
1.424
1.402
1.387
Acetone
HO
NH
NH
Br
8.0 7.5 7.0 6.5 6.0 5.5 5.0 4.5 4.0 3.5 3.0 2.5 2.0 1.5 1.0 0.5 ppm
1.0
1.1
2.2
2.1
1.0
2.1
2.2
1.2
6.5

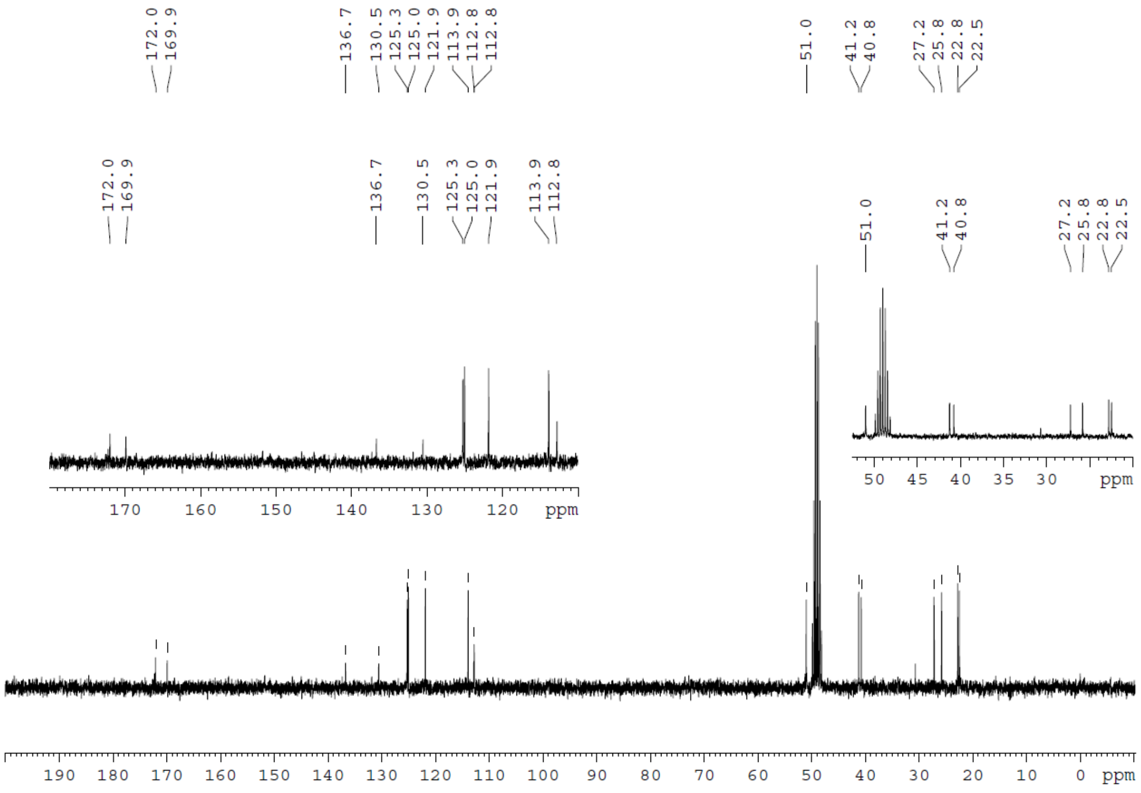
172.0
169.9
136.7
130.5
125.3
125.0
121.9
113.9
112.8
112.8
51.0
41.2
40.8
27.2
25.8
22.8
22.5
170 160 150 140 130 120 ppm
50 45 40 35 30 ppm
190 180 170 160 150 140 130 120 110 100 90 80 70 60 50 40 30 20 10 0 ppm

Compound 14

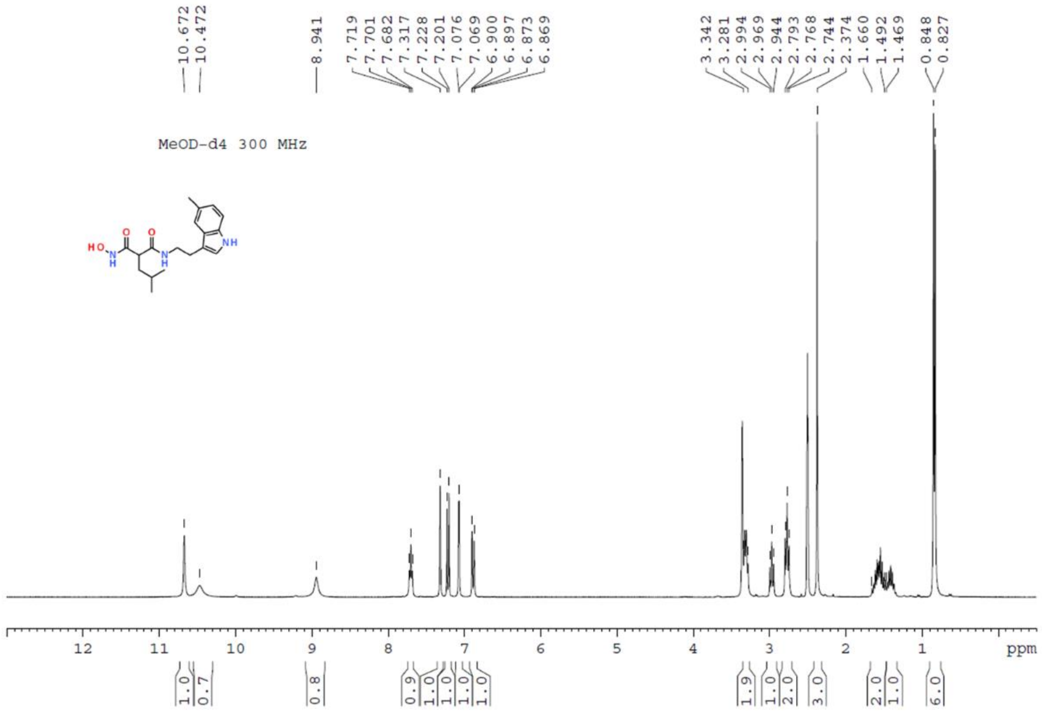

10.672
10.472
8.941
7.719
7.701
7.682
7.317
7.228
7.201
7.076
7.069
6.900
6.897
6.873
6.869
3.342
3.281
2.994
2.969
2.944
2.793
2.768
2.744
2.374
1.660
1.492
1.469
0.848
0.827
MeOD-d4 300 MHz
HO N H O O N H NH
12 11 10 9 8 7 6 5 4 3 2 1 ppm
1.0 0.7 0.8 0.9 1.0 1.0 1.0 1.0 1.0 1.9 1.0 2.0 3.0 2.0 1.0 6.0

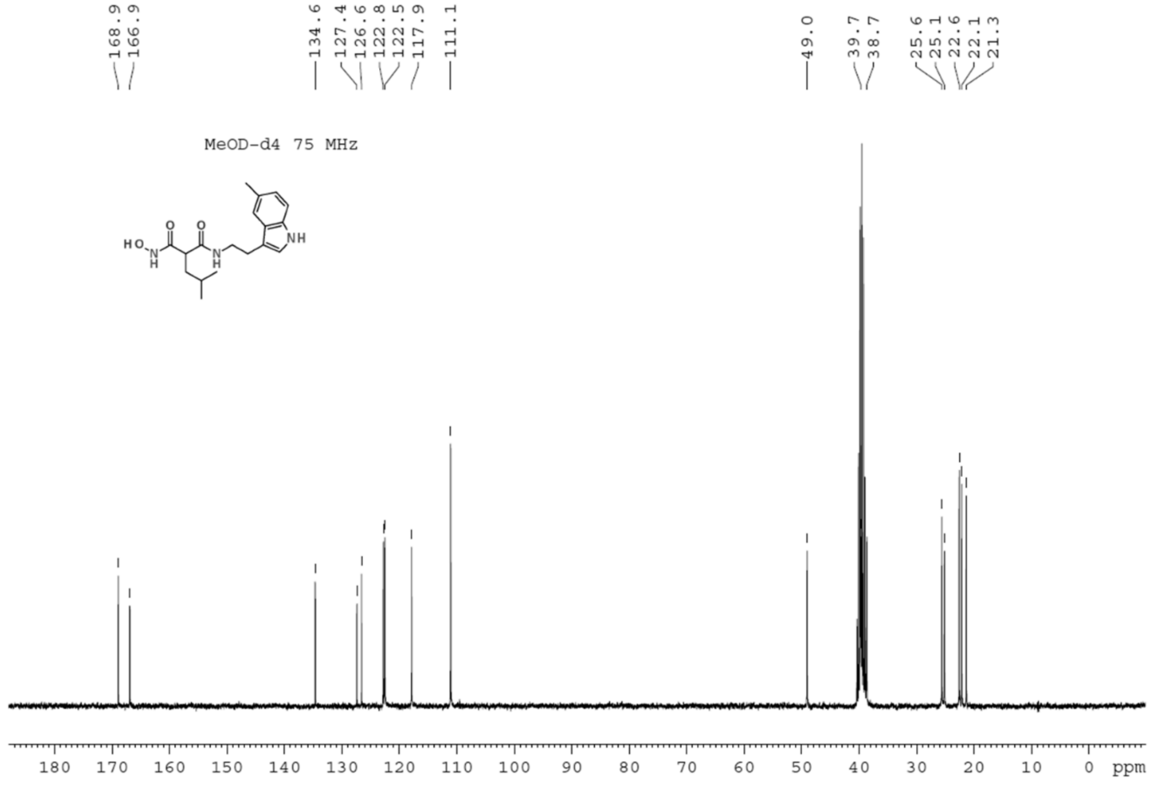

168.9
166.9
134.6
127.4
126.6
122.8
122.5
117.9
111.1
49.0
39.7
38.7
25.6
25.1
22.6
22.1
21.3
MeOD-d4 75 MHz
HO N H O O N H NH
180 170 160 150 140 130 120 110 100 90 80 70 60 50 40 30 20 10 0 ppm

Compound 15

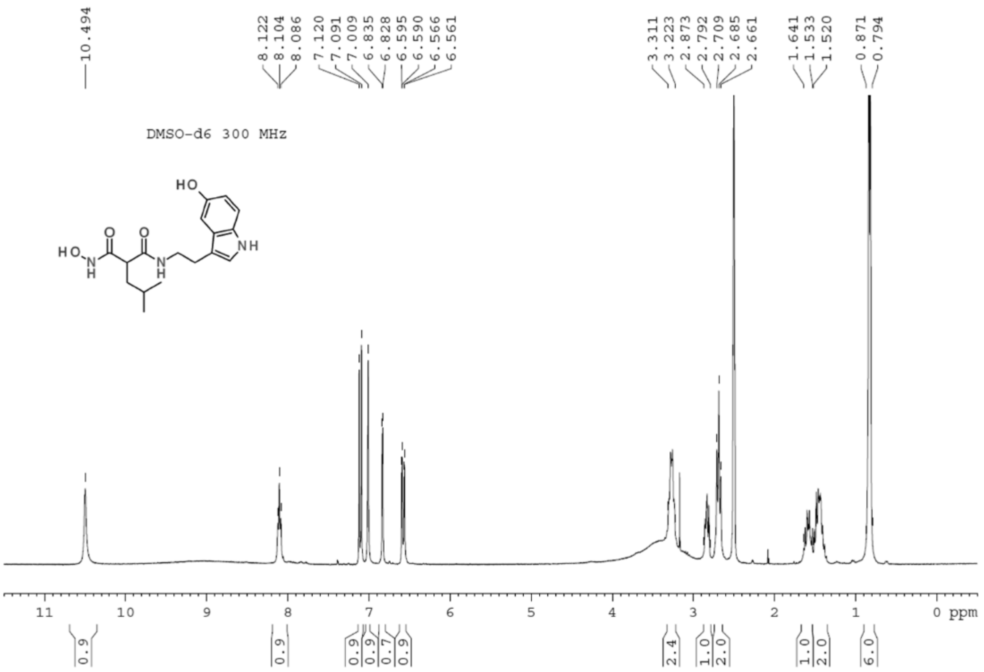

10.494
8.122
8.104
8.086
7.120
7.091
7.009
6.835
6.828
6.595
6.590
6.566
6.561
3.311
3.223
2.873
2.792
2.709
2.685
2.661
1.641
1.533
1.520
0.871
0.794
DMSO-d6 300 MHz
HO
NH
HO
N
H
N
H
O
O
11 10 9 8 7 6 5 4 3 2 1 0 ppm
0.9
0.9
0.9
0.9
0.7
0.9
2.4
1.0
2.0
1.0
2.0
6.0

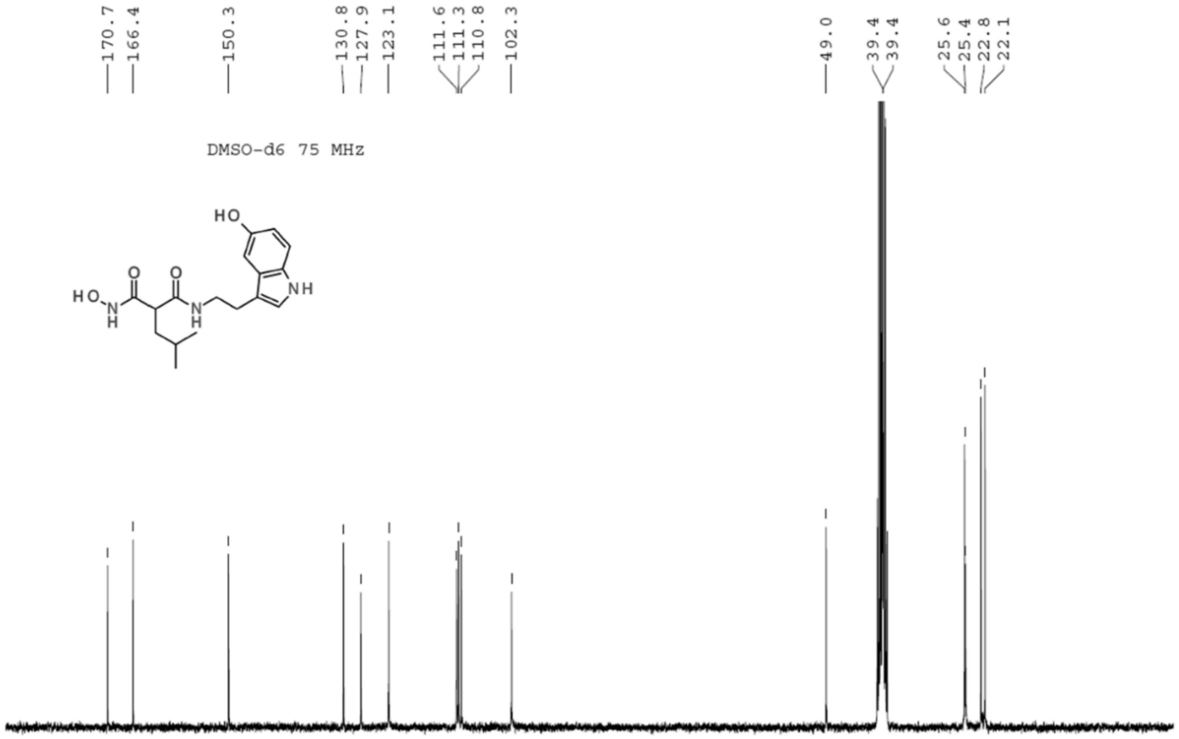

170.7
166.4
150.3
130.8
127.9
123.1
111.6
111.3
110.8
102.3
49.0
39.4
39.4
25.6
25.4
22.8
22.1
DMSO-d6 75 MHz
HO
NH
HO
N
H
N
H
O
O
180 170 160 150 140 130 120 110 100 90 80 70 60 50 40 30 20 10 0 ppm

Compound 16

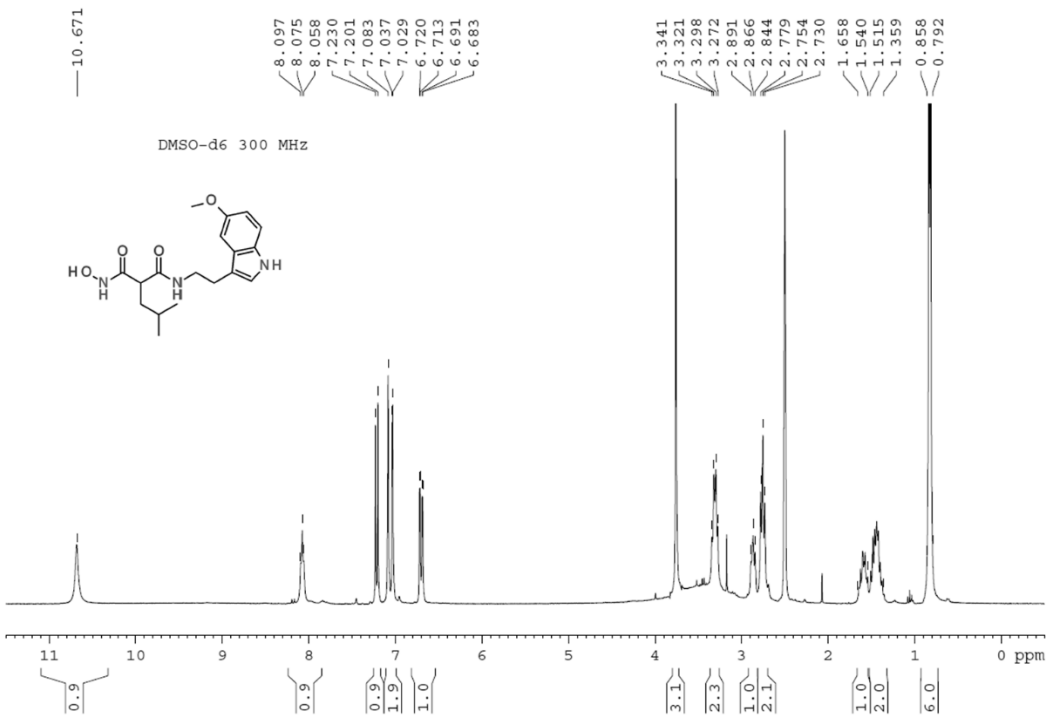
10.671
8.097 8.075 8.058 7.230 7.201 7.083 7.037 7.029 6.720 6.713 6.691 6.683
3.341 3.321 3.298 3.272 2.891 2.866 2.844 2.779 2.754 2.730 1.658 1.540 1.515 1.359 0.858 0.792
DMSO-d6 300 MHz
HO N H
O O
N H
NH
O
11 10 9 8 7 6 5 4 3 2 1 0 ppm
0.9 0.9 0.9 1.9 1.0 3.1 2.3 1.0 2.1 1.0 2.0 6.0

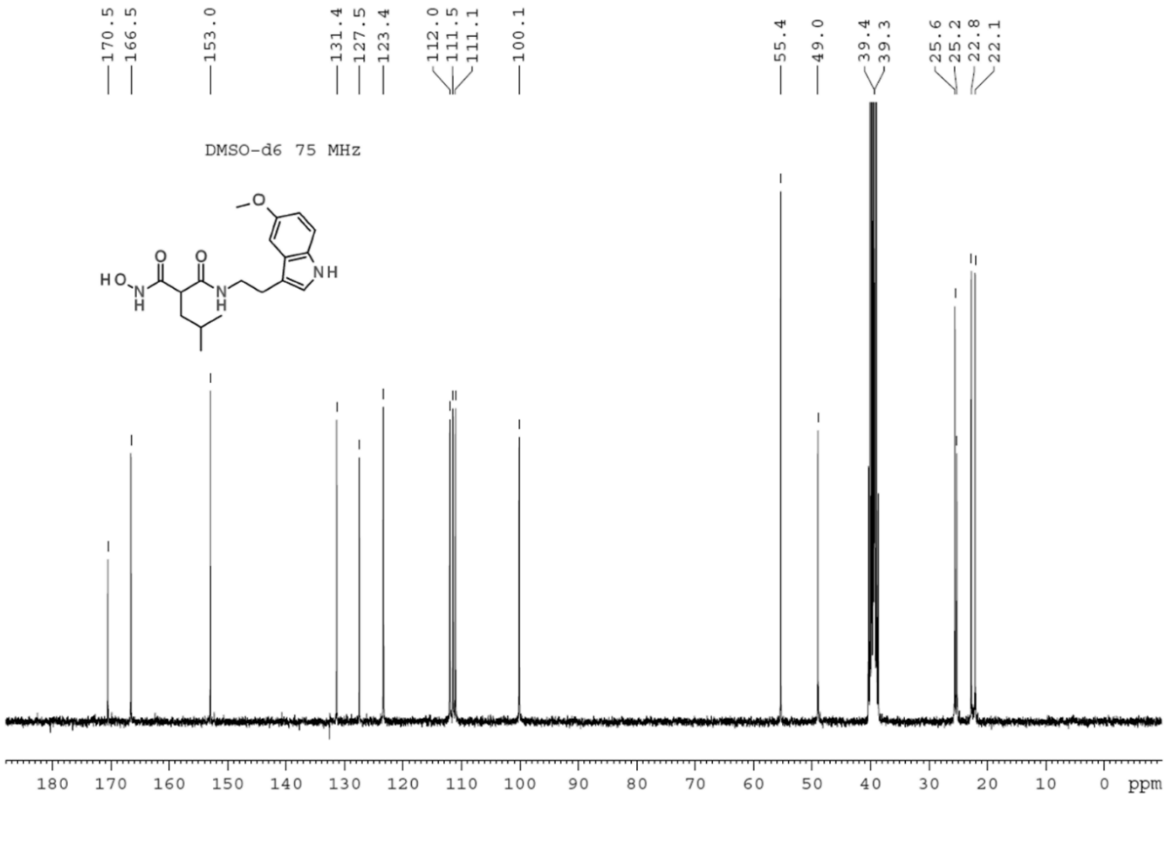
170.5 166.5 153.0 131.4 127.5 123.4 112.0 111.5 111.1 100.1 55.4 49.0 39.4 39.3 25.6 25.2 22.8 22.1
DMSO-d6 75 MHz
HO N H
O O
N H
NH
O
180 170 160 150 140 130 120 110 100 90 80 70 60 50 40 30 20 10 0 ppm

Compound 17

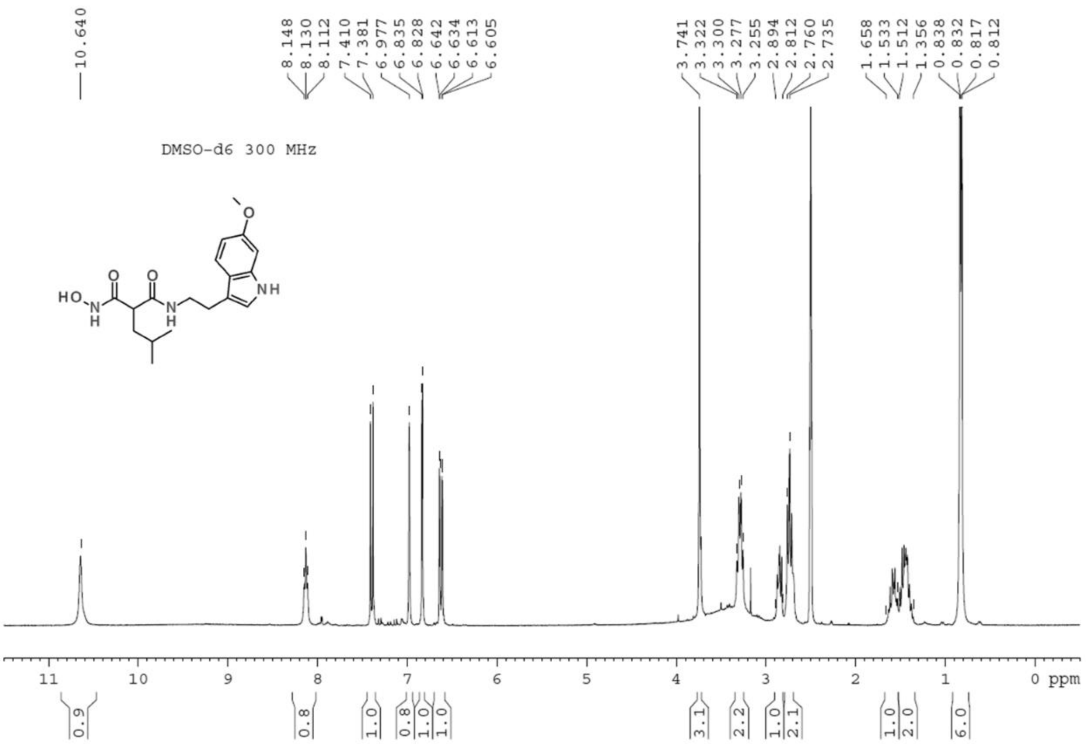
10.640
8.148
8.130
8.112
7.410
7.381
6.977
6.835
6.828
6.642
6.634
6.613
6.605
3.741
3.322
3.300
3.277
3.255
2.894
2.812
2.760
2.735
1.658
1.533
1.512
1.356
0.838
0.832
0.817
0.812
DMSO-d6 300 MHz
HO
N
H
O
O
N
H
NH
O
11 10 9 8 7 6 5 4 3 2 1 0 ppm
0.9
0.8
1.0
0.8
1.0
1.0
3.1
2.2
1.0
2.1
1.0
2.0
6.0

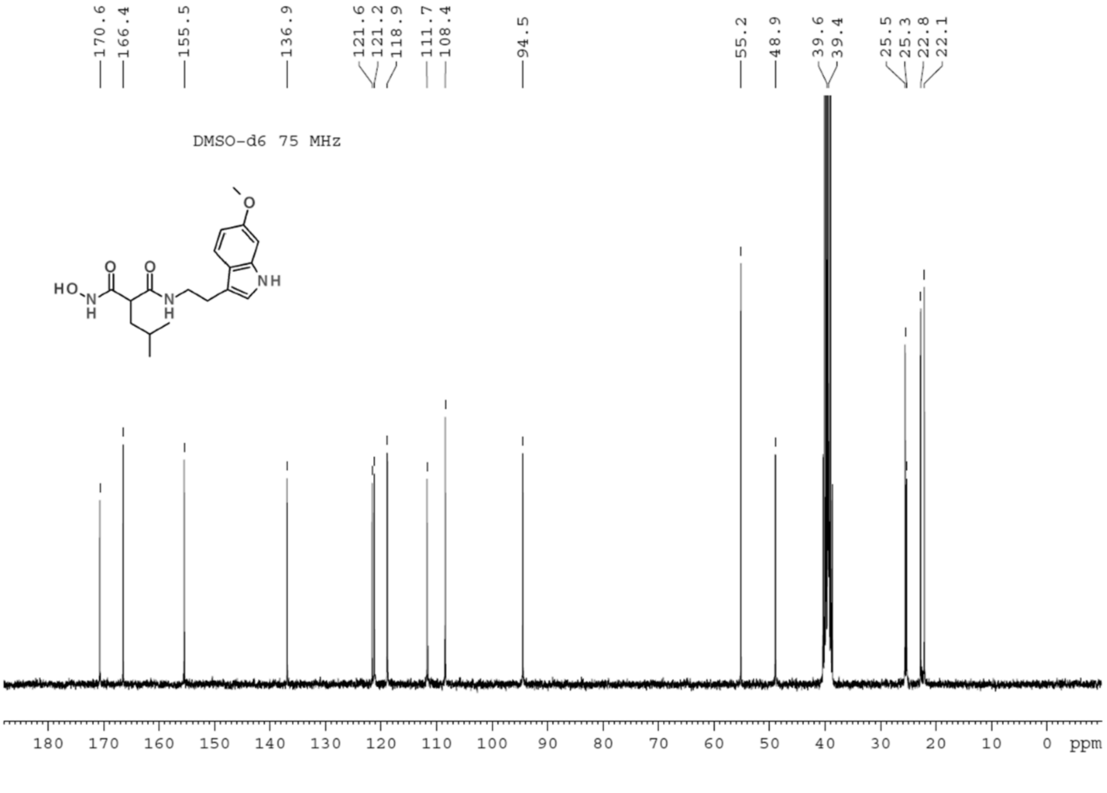
170.6
166.4
155.5
136.9
121.6
121.2
118.9
111.7
108.4
94.5
55.2
48.9
39.6
39.4
25.5
25.3
22.8
22.1
DMSO-d6 75 MHz
HO
N
H
O
O
N
H
NH
O
180 170 160 150 140 130 120 110 100 90 80 70 60 50 40 30 20 10 0 ppm

Compound 18

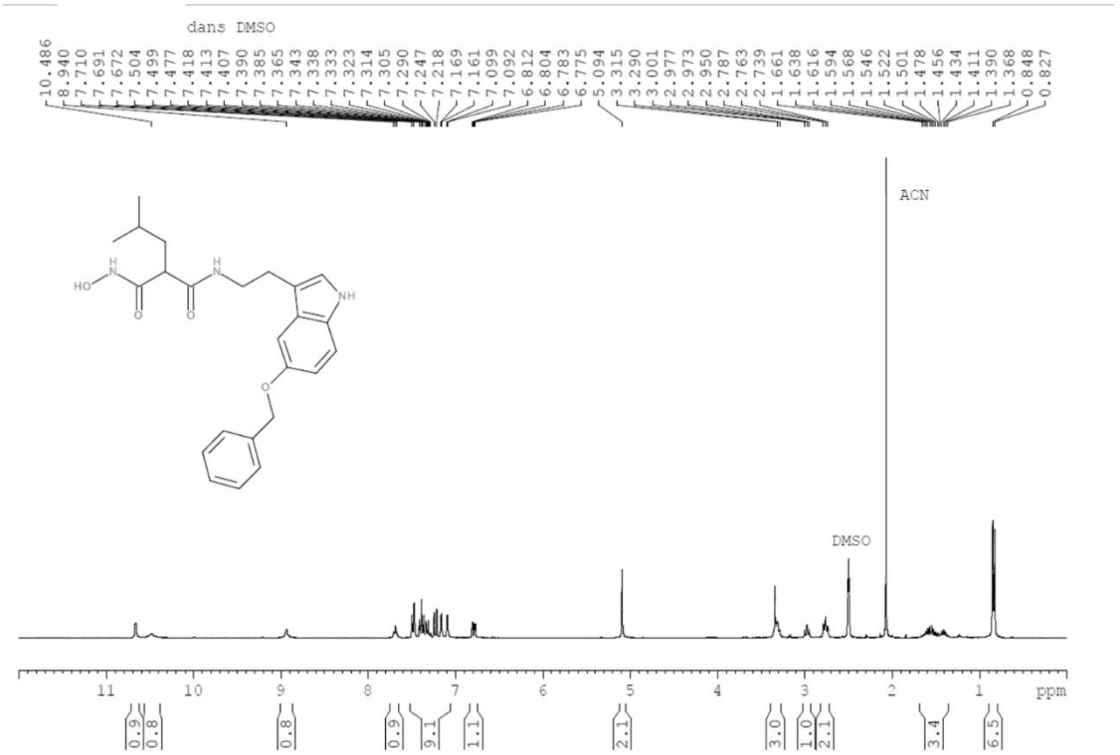
dans DMSO
10.486
8.940
7.710
7.691
7.672
7.504
7.499
7.477
7.418
7.413
7.407
7.390
7.385
7.365
7.343
7.338
7.333
7.323
7.314
7.305
7.290
7.247
7.218
7.169
7.161
7.099
7.092
6.812
6.804
6.783
6.775
5.094
3.315
3.290
3.001
2.977
2.973
2.950
2.787
2.763
2.739
1.661
1.638
1.616
1.594
1.568
1.546
1.522
1.501
1.478
1.456
1.434
1.411
1.390
1.368
0.848
0.827
ACN
HO
H
N
N
O
O
NH
O
DMSO
11
10
9
8
7
6
5
4
3
2
1
ppm
0.9
0.8
0.8
0.9
9.1
1.1
2.1
3.0
1.0
2.1
3.4
6.5

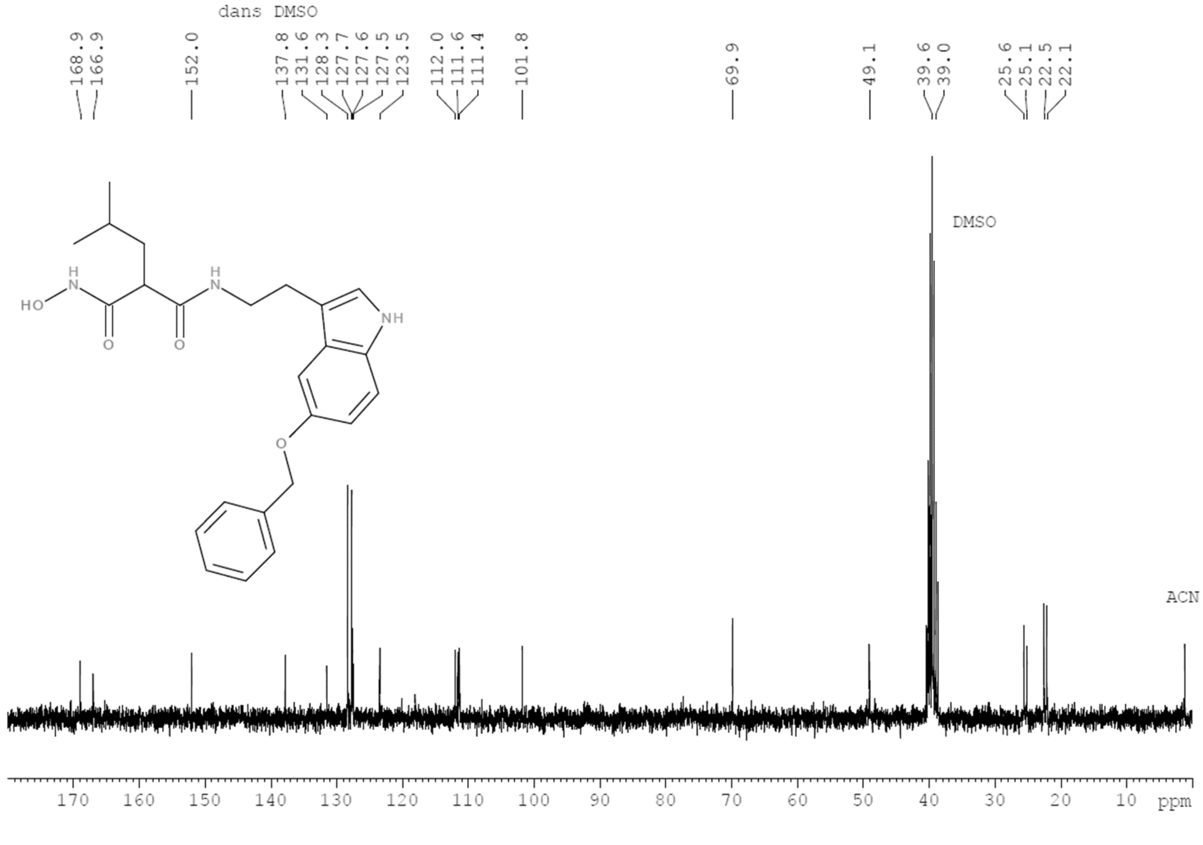
dans DMSO
168.9
166.9
152.0
137.8
131.6
128.3
127.7
127.6
127.5
123.5
112.0
111.6
111.4
101.8
69.9
49.1
39.6
39.0
25.6
25.1
22.5
22.1
DMSO
HO
H
N
N
O
O
NH
O
ACN
170
160
150
140
130
120
110
100
90
80
70
60
50
40
30
20
10
ppm

Compound 19

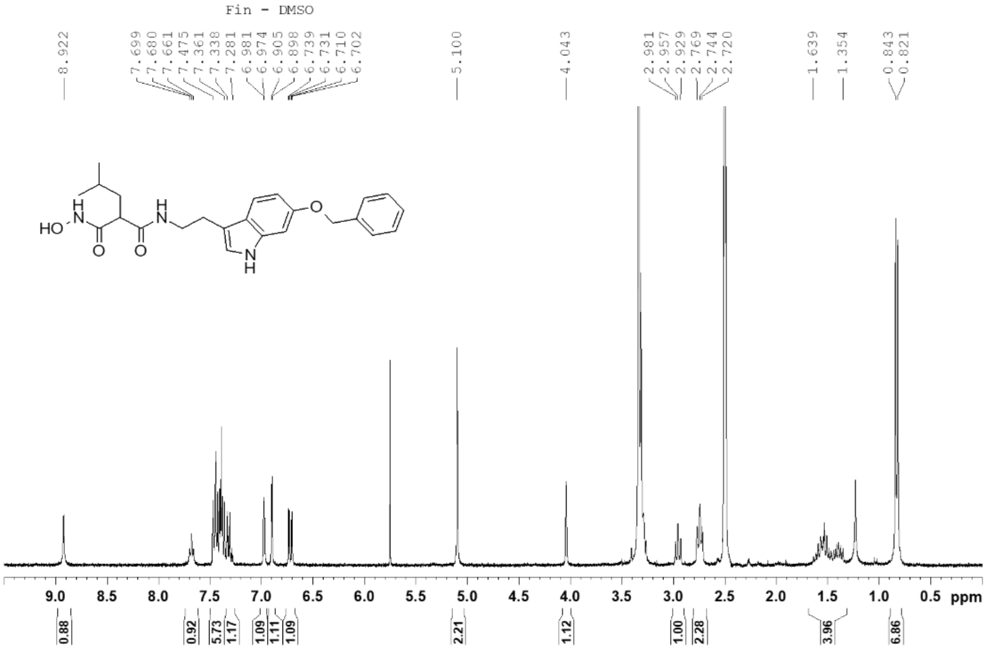
Fin - DMSO
8.922
7.699
7.680
7.661
7.475
7.361
7.338
7.281
6.981
6.974
6.905
6.898
6.739
6.731
6.710
6.702
5.100
4.043
2.981
2.957
2.929
2.769
2.744
2.720
1.639
1.354
0.843
0.821
HO
N
H
N
H
O
O
O
N
H
9.0 8.5 8.0 7.5 7.0 6.5 6.0 5.5 5.0 4.5 4.0 3.5 3.0 2.5 2.0 1.5 1.0 0.5 ppm
0.88
0.92
5.73
1.17
1.09
1.11
1.09
2.21
1.12
1.00
2.28
3.96
6.86

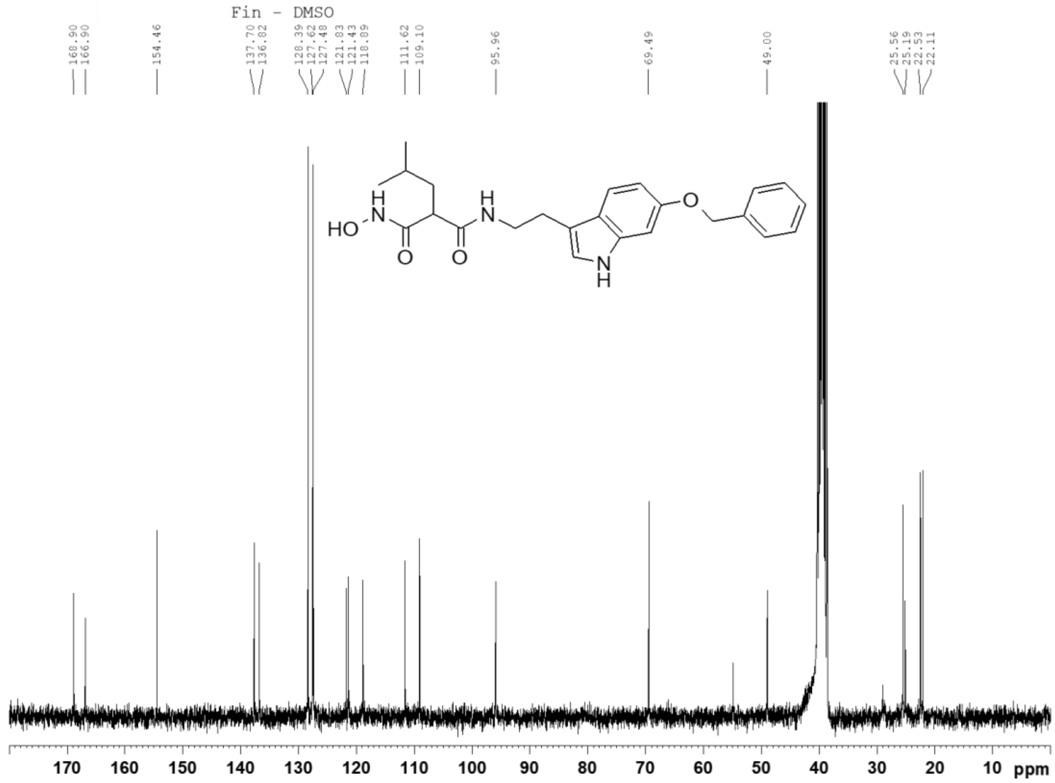
Fin - DMSO
168.90
166.90
154.46
137.70
136.82
128.39
127.62
127.48
121.83
121.43
118.89
111.62
109.10
95.96
69.49
49.00
25.56
25.19
22.53
22.11
HO
N
H
N
H
O
O
O
N
H
170 160 150 140 130 120 110 100 90 80 70 60 50 40 30 20 10 ppm

Compound 20

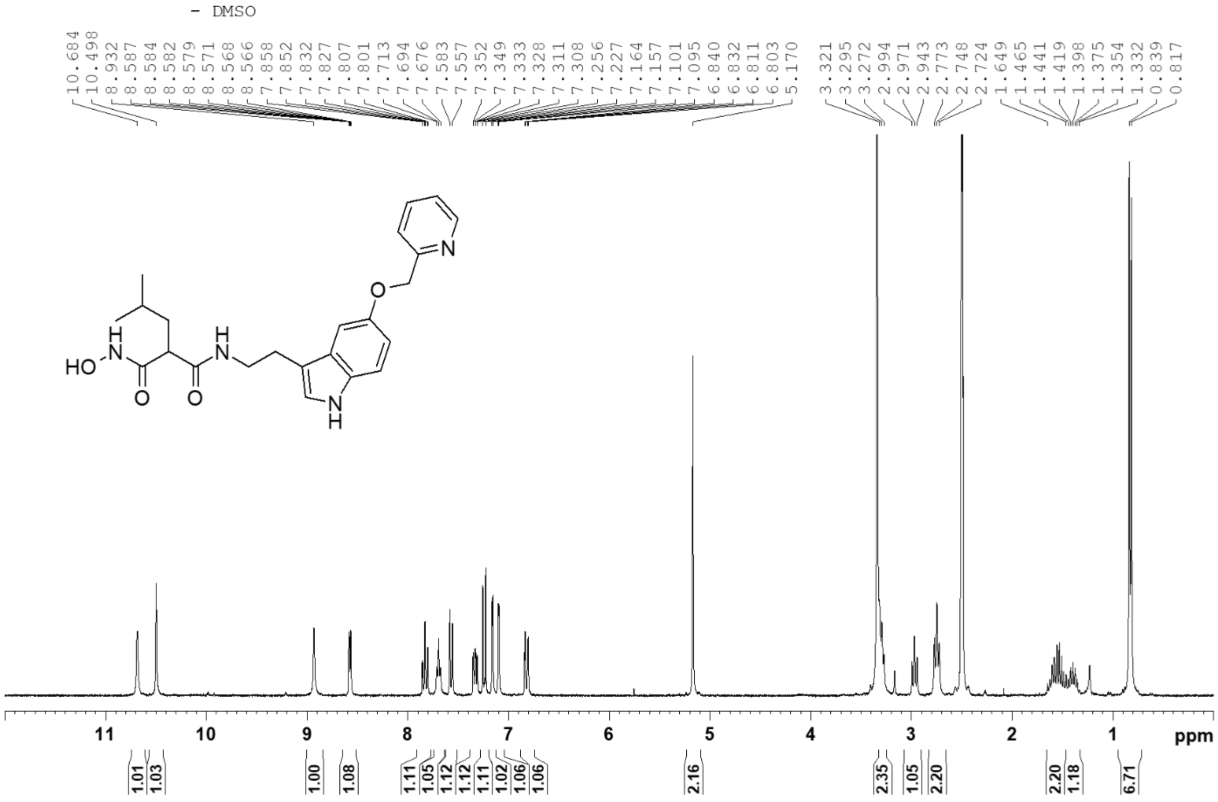

- DMSO
10.684
10.498
8.932
8.587
8.584
8.582
8.579
8.571
8.568
8.566
7.858
7.852
7.832
7.827
7.807
7.801
7.713
7.694
7.676
7.583
7.557
7.352
7.349
7.333
7.328
7.311
7.308
7.256
7.227
7.164
7.157
7.101
7.095
6.840
6.832
6.811
6.803
5.170
3.321
3.295
3.272
2.994
2.971
2.943
2.773
2.748
2.724
1.649
1.465
1.441
1.419
1.398
1.375
1.354
1.332
0.839
0.817
HO
N
H
O
O
N
H
N
H
O
N
11
10
9
8
7
6
5
4
3
2
1
ppm
1.01
1.03
1.00
1.08
1.11
1.05
1.12
1.12
1.11
1.02
1.06
1.06
2.16
2.35
1.05
2.20
2.20
1.18
6.71

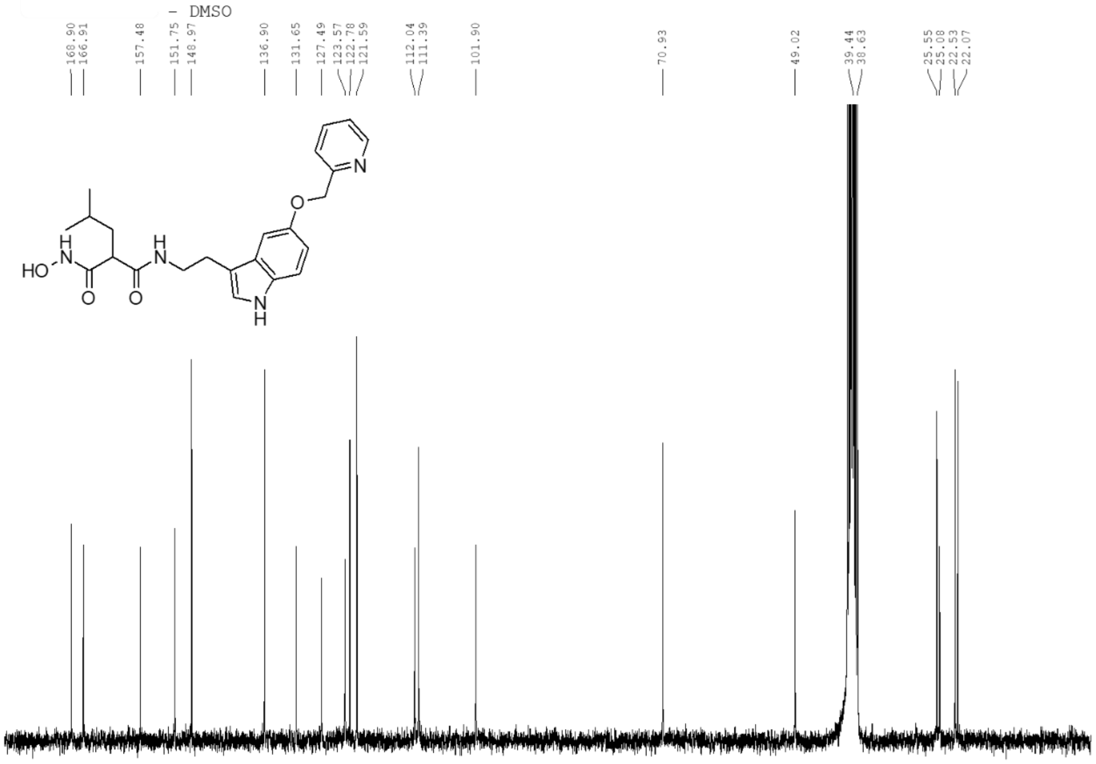

- DMSO
168.90
166.91
157.48
151.75
148.97
136.90
131.65
127.49
123.57
122.78
121.59
112.04
111.39
101.90
70.93
49.02
39.44
38.63
25.55
25.08
22.53
22.07
HO
N
H
O
O
N
H
N
H
O
N
170
160
150
140
130
120
110
100
90
80
70
60
50
40
30
20
10
ppm

Compound 21

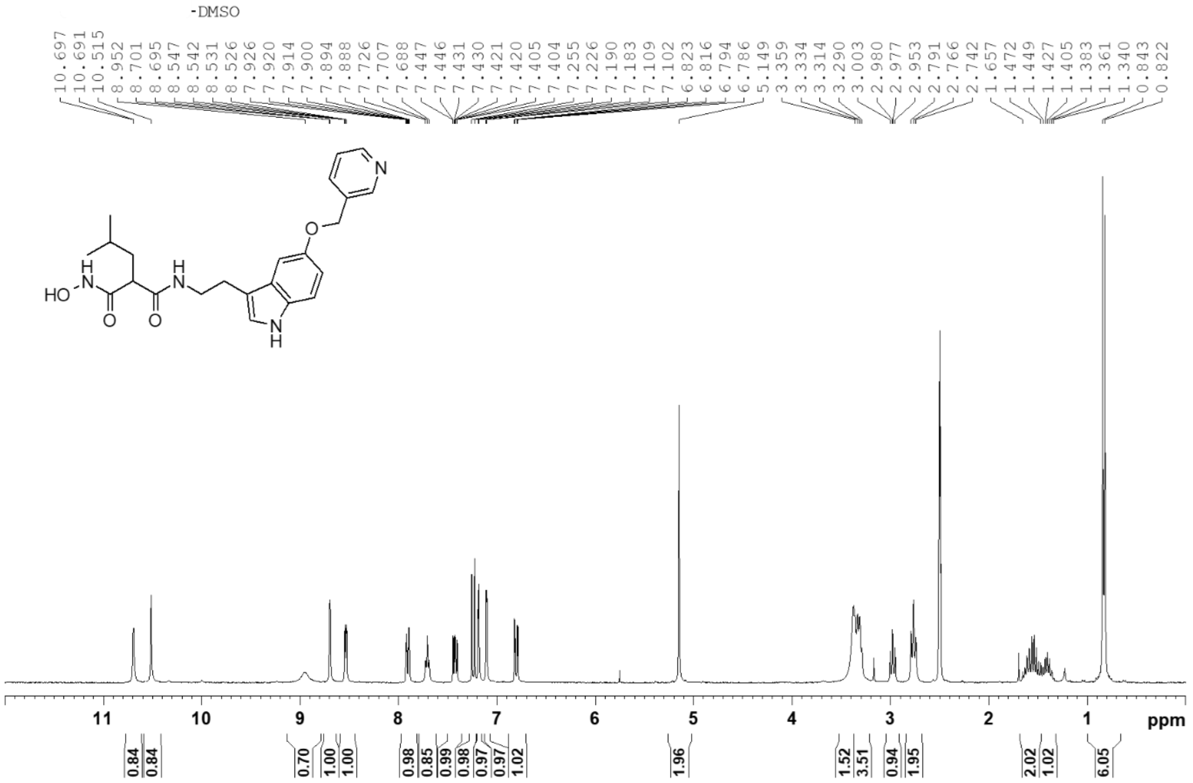
-DMSO
10.697 10.691 10.515 8.952 8.701 8.695 8.547 8.542 8.531 8.526 7.926 7.920 7.914 7.900 7.894 7.888 7.726 7.707 7.688 7.447 7.446 7.431 7.430 7.421 7.420 7.405 7.404 7.255 7.226 7.190 7.183 7.109 7.102 6.823 6.816 6.794 6.786 5.149 3.359 3.334 3.314 3.290 3.003 2.980 2.977 2.953 2.791 2.766 2.742 1.657 1.472 1.449 1.427 1.405 1.383 1.361 1.340 0.843 0.822
HO
N
H
O
O
N
H
O
N
N
H
11 10 9 8 7 6 5 4 3 2 1 ppm
0.84 0.84 0.70 1.00 1.00 0.98 0.85 0.99 0.98 0.97 0.97 1.02 1.96 1.52 3.51 0.94 1.95 2.02 1.02 6.05

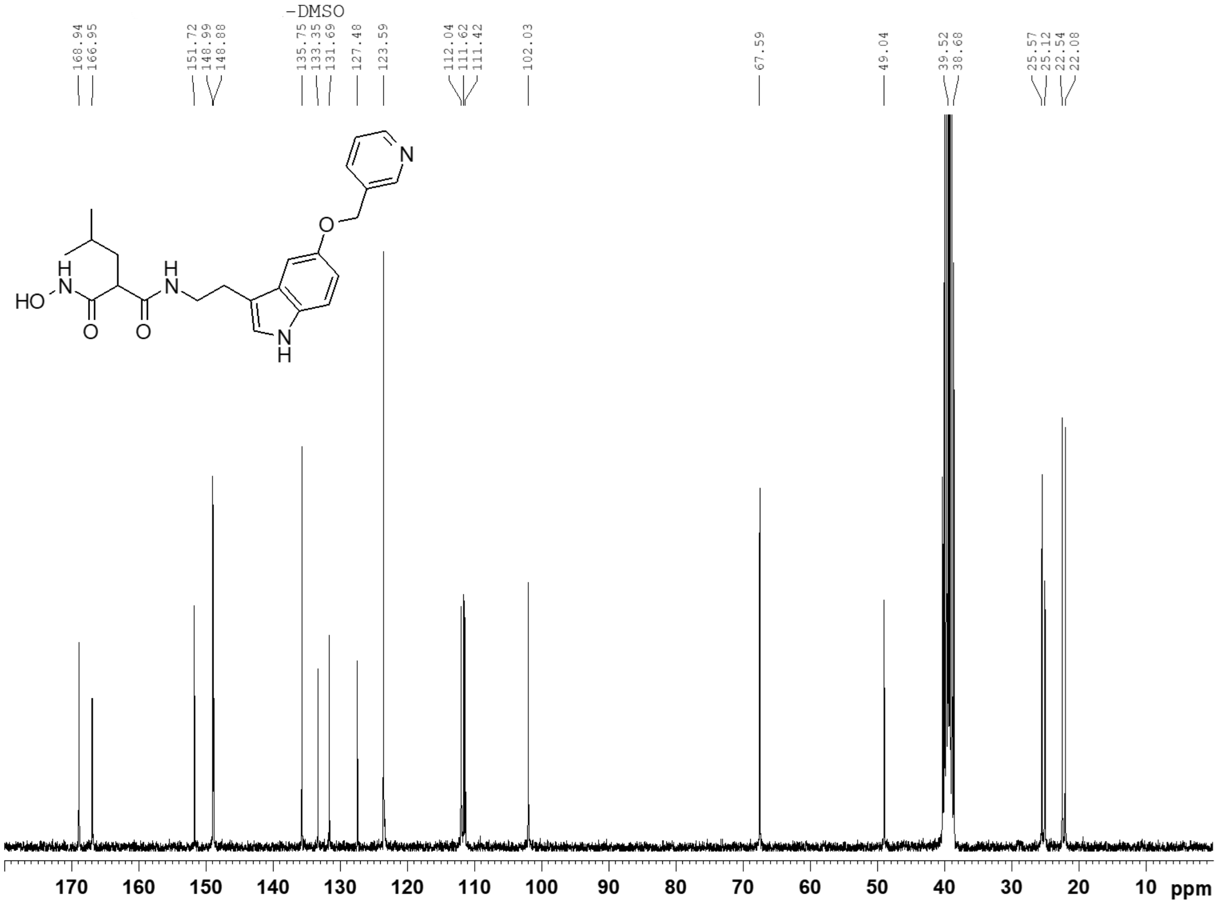
-DMSO
168.94 166.95 151.72 148.99 148.88 135.75 133.35 131.69 127.48 123.59 112.04 111.62 111.42 102.03 67.59 49.04 39.52 38.68 25.57 25.12 22.54 22.08
HO
N
H
O
O
N
H
O
N
N
H
170 160 150 140 130 120 110 100 90 80 70 60 50 40 30 20 10 ppm

Compound 22

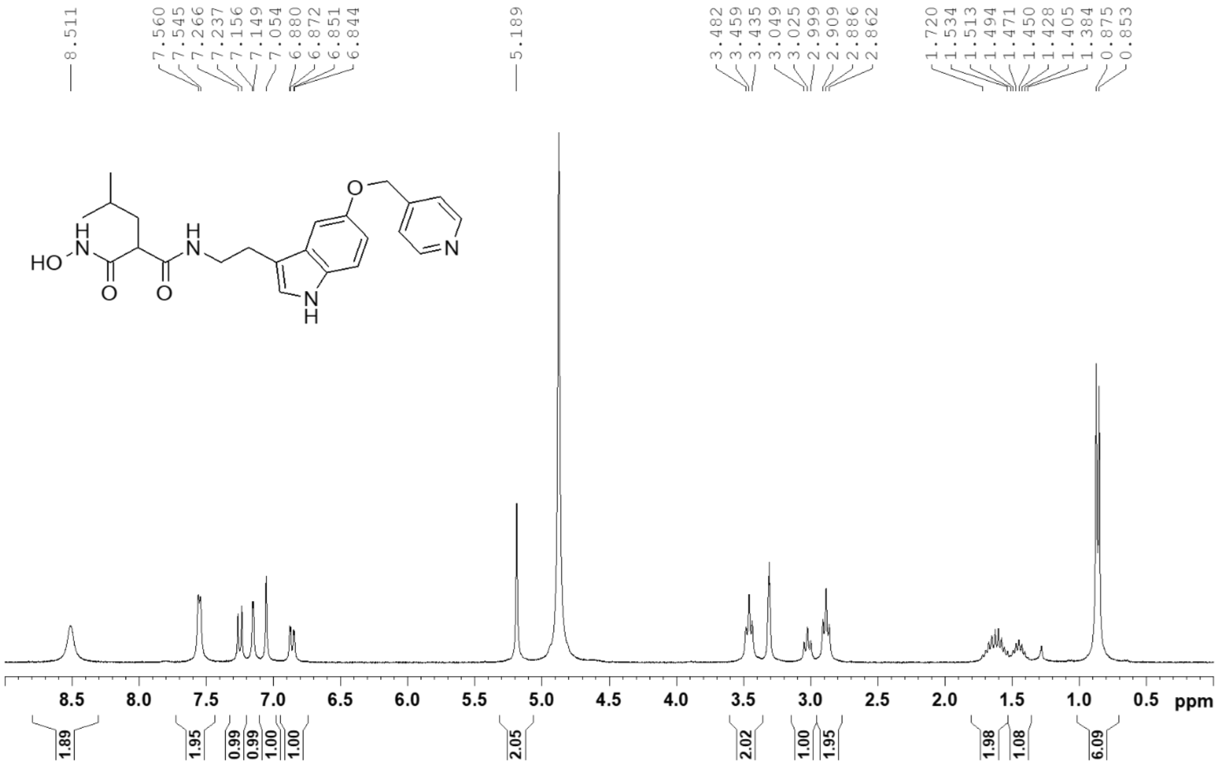
8.511
7.560 7.545 7.266 7.237 7.156 7.149 7.054 6.880 6.872 6.851 6.844
5.189
3.482 3.459 3.435 3.049 3.025 2.999 2.909 2.886 2.862
1.720 1.534 1.513 1.494 1.471 1.450 1.428 1.405 1.384 0.875 0.853
HO N H O O N H N H O N
8.5 8.0 7.5 7.0 6.5 6.0 5.5 5.0 4.5 4.0 3.5 3.0 2.5 2.0 1.5 1.0 0.5 ppm
1.89 1.95 0.99 0.99 1.00 1.00 2.05 2.02 1.00 1.95 1.98 1.08 6.09

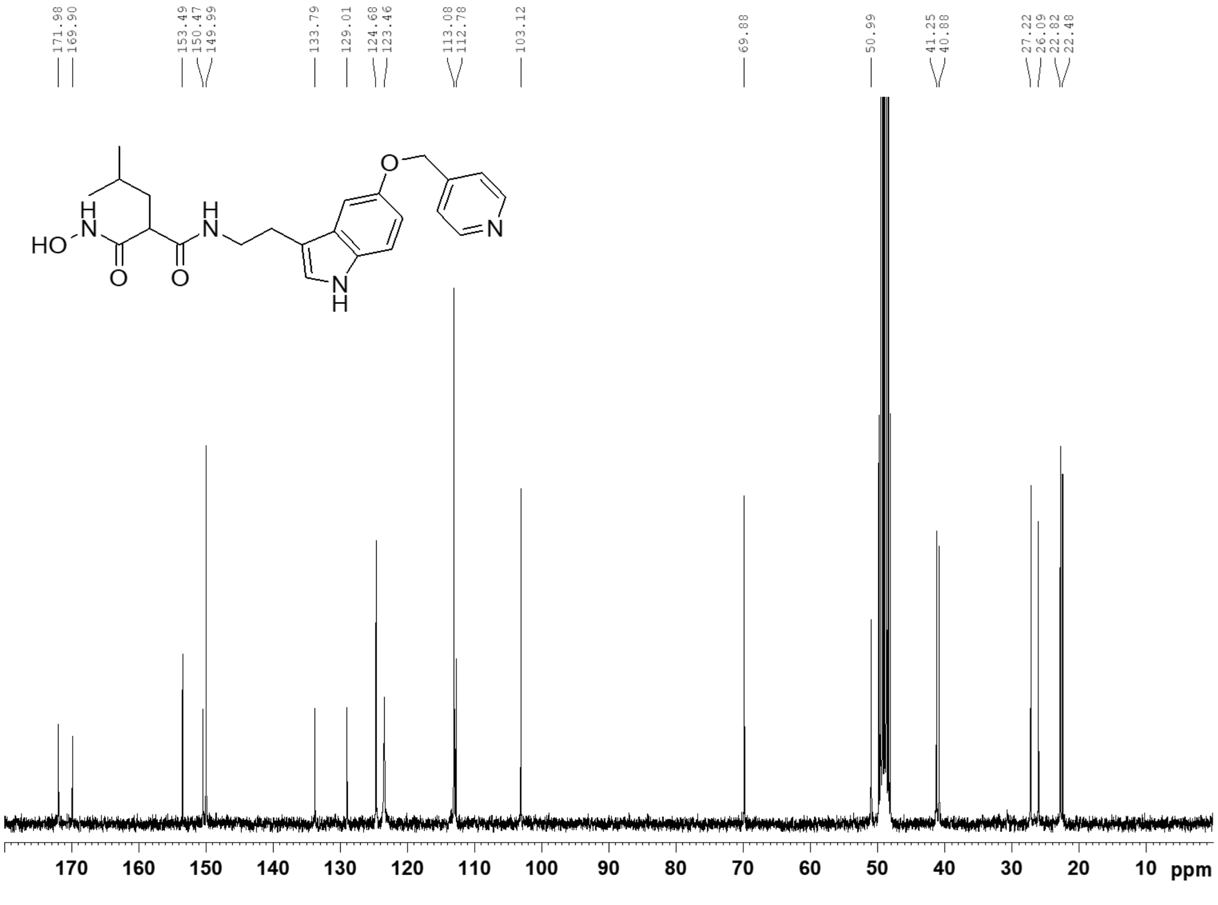
171.98 169.90 153.49 150.47 149.99 133.79 129.01 124.68 123.46 113.08 112.78 103.12 69.88 50.99 41.25 40.88 27.22 26.09 22.82 22.48
HO N H O O N H N H O N
170 160 150 140 130 120 110 100 90 80 70 60 50 40 30 20 10 ppm

Compound 23

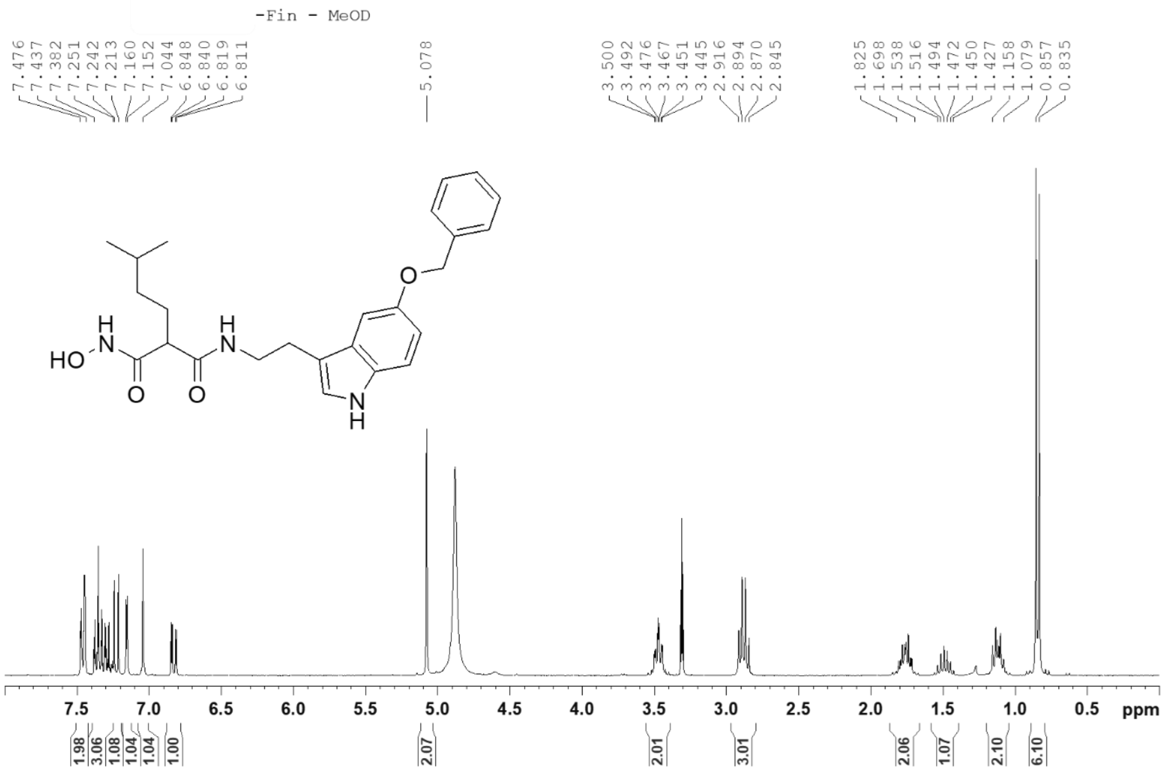
-Fin - MeOD
7.476 7.437 7.382 7.251 7.242 7.213 7.160 7.152 7.044 6.848 6.840 6.819 6.811
5.078
3.500 3.492 3.476 3.467 3.451 3.445 2.916 2.894 2.870 2.845
1.825 1.698 1.538 1.516 1.494 1.472 1.450 1.427 1.158 1.079 0.857 0.835
HO N H O O N H N H O
7.5 7.0 6.5 6.0 5.5 5.0 4.5 4.0 3.5 3.0 2.5 2.0 1.5 1.0 0.5 ppm
1.98 3.06 1.08 1.04 1.04 1.00 2.07 2.01 3.01 2.06 1.07 2.10 6.10

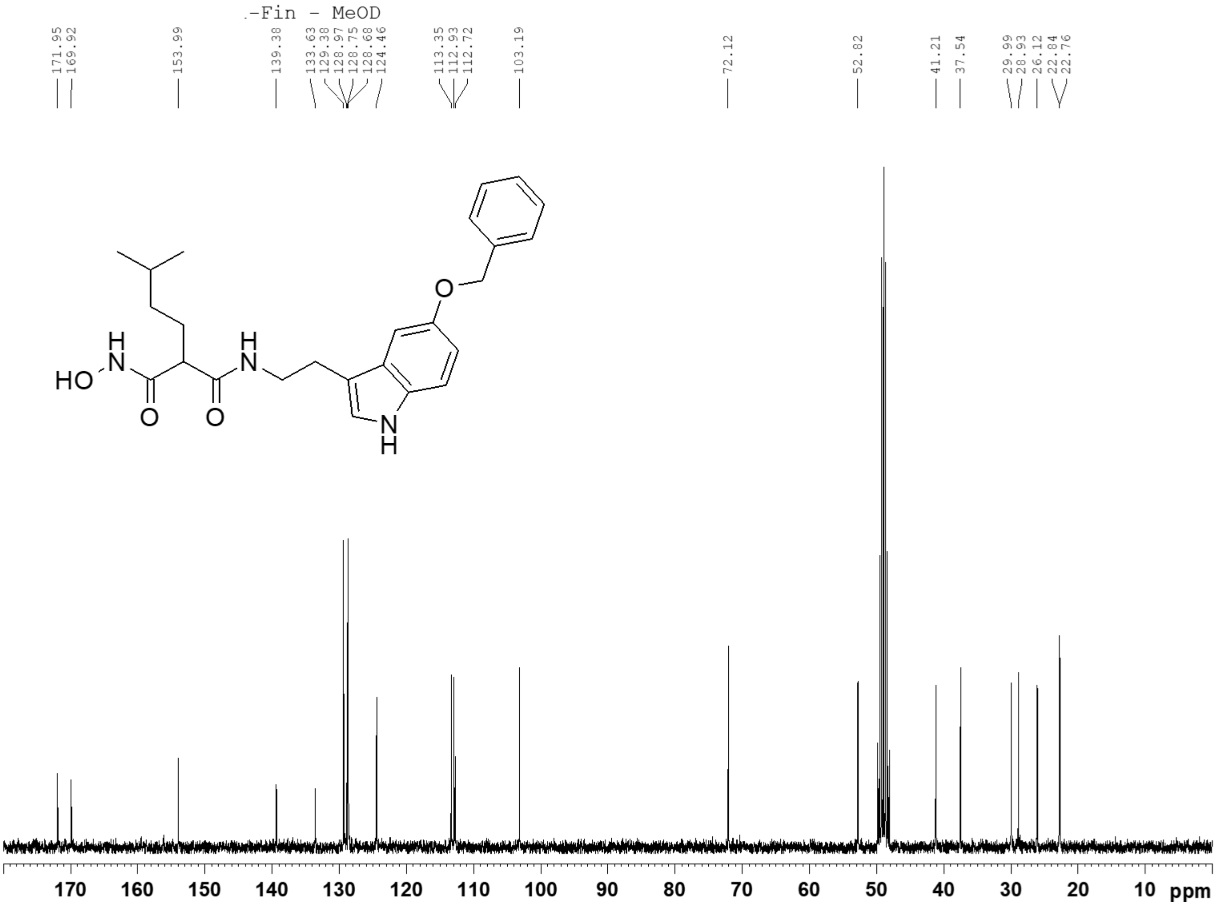
-Fin - MeOD
171.95 169.92 153.99 139.38 133.63 129.38 128.97 128.75 128.68 124.46 113.35 112.93 112.72 103.19 72.12 52.82 41.21 37.54 29.99 28.93 26.12 22.84 22.76
HO N H O O N H N H O
170 160 150 140 130 120 110 100 90 80 70 60 50 40 30 20 10 ppm

Compound 24

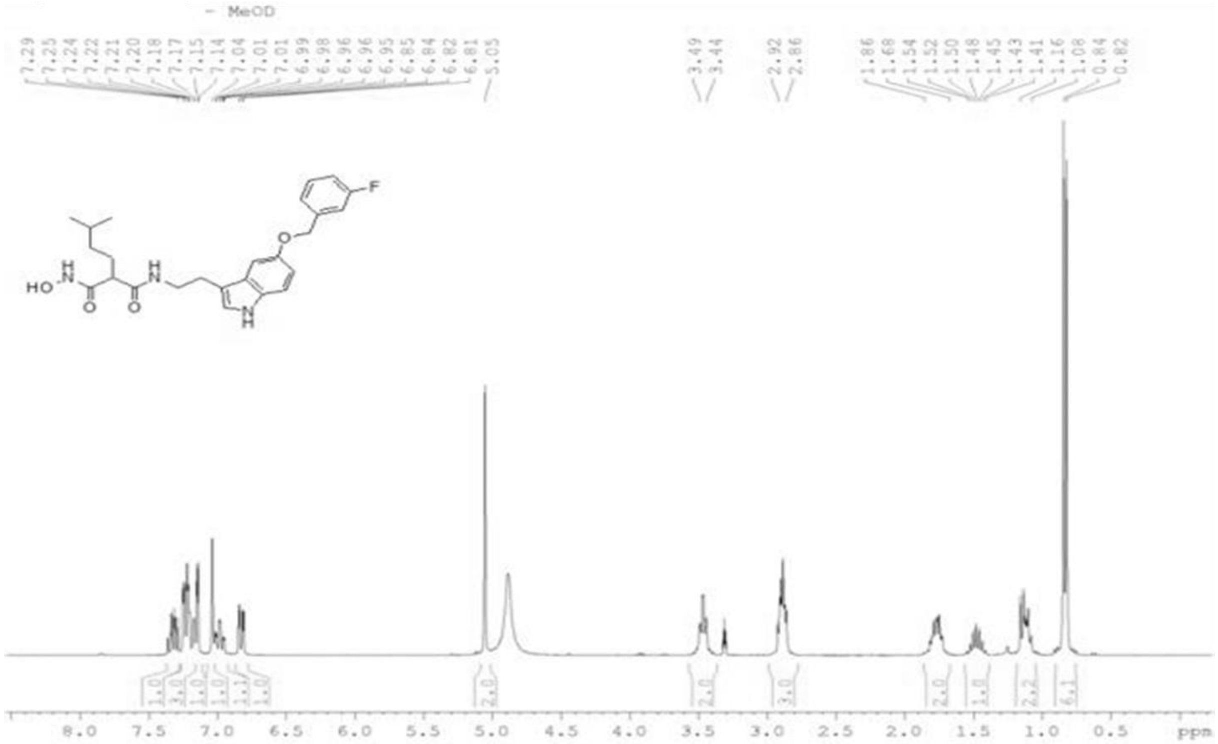
- MeOD
7.29 7.25 7.24 7.22 7.21 7.20 7.18 7.17 7.15 7.14 7.04 7.01 7.01 6.99 6.98 6.96 6.96 6.95 6.85 6.84 6.82 6.81 5.05
3.49 3.44
2.92 2.86
1.86 1.68 1.54 1.52 1.50 1.48 1.45 1.43 1.41 1.16 1.08 0.84 0.82
HO N H O O N H N H O F
8.0 7.5 7.0 6.5 6.0 5.5 5.0 4.5 4.0 3.5 3.0 2.5 2.0 1.5 1.0 0.5 ppm

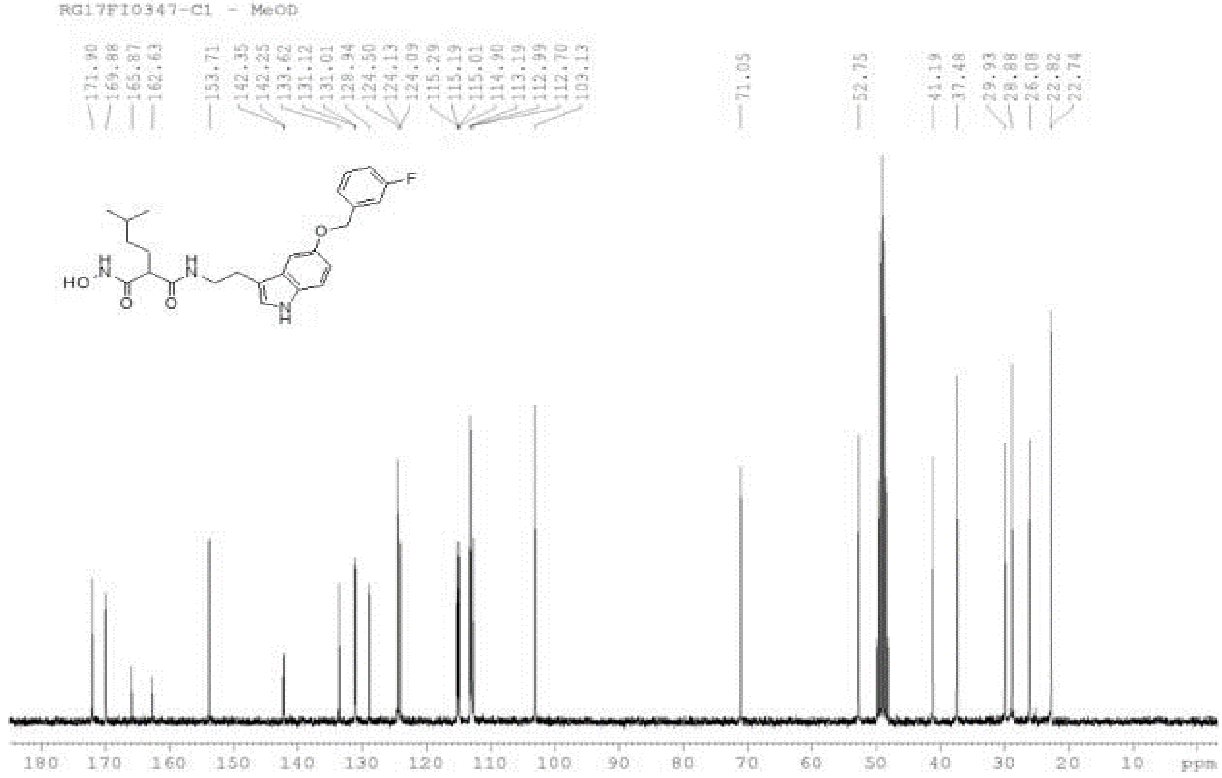
RG17FI0347-C1 - MeOD
171.90 169.88 165.87 162.63
153.71 142.35 142.25 133.62 131.12 131.01 128.94 124.50 124.13 124.09 115.29 115.19 115.01 114.90 113.19 112.99 112.70 103.13
71.05
52.75
41.19 37.48 29.93 28.88 26.08 22.82 22.74
HO N H O O N H N H O F
180 170 160 150 140 130 120 110 100 90 80 70 60 50 40 30 20 10 ppm

Compound 25

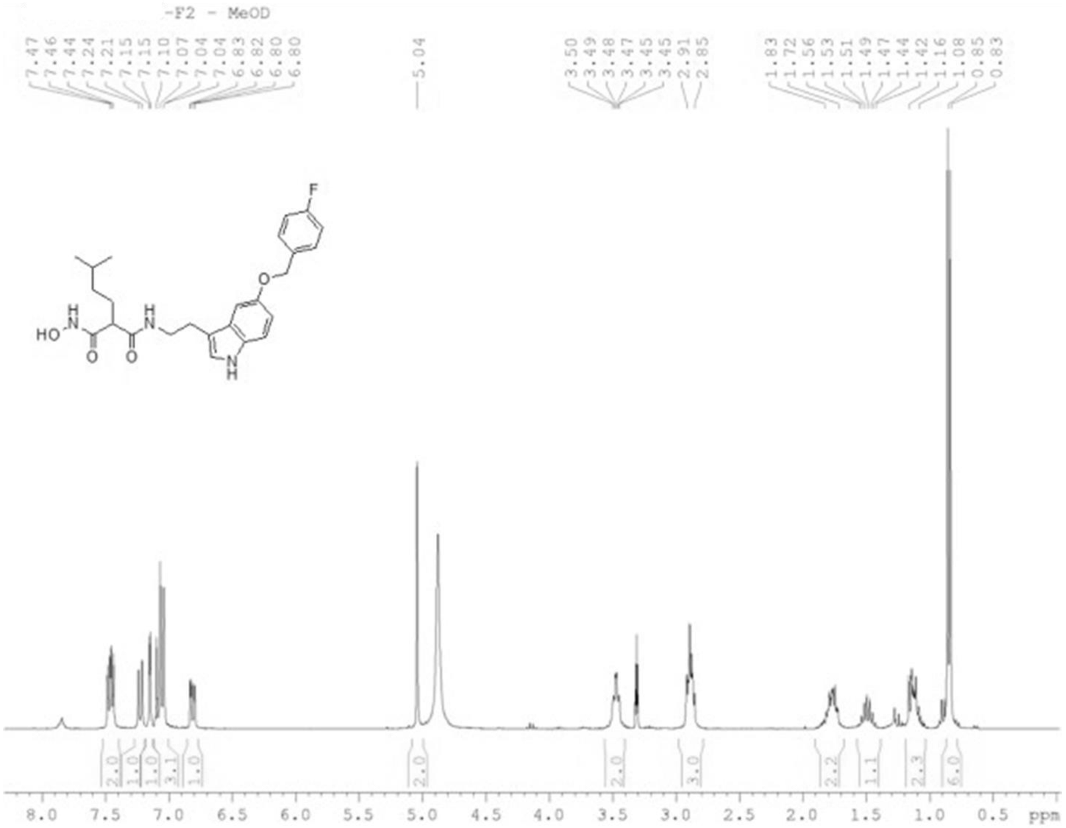
-F2 - MeOD
7.47
7.46
7.44
7.24
7.21
7.15
7.15
7.10
7.07
7.04
7.04
6.83
6.82
6.80
6.80
5.04
3.50
3.49
3.48
3.47
3.45
3.45
2.91
2.85
1.83
1.72
1.56
1.53
1.51
1.49
1.47
1.44
1.42
1.16
1.08
0.85
0.83
F
HO
H
N
O
O
H
N
O
N
H
2.0
1.0
1.0
3.1
1.0
2.0
2.0
3.0
2.2
1.1
2.3
6.0
8.0
7.5
7.0
6.5
6.0
5.5
5.0
4.5
4.0
3.5
3.0
2.5
2.0
1.5
1.0
0.5
ppm

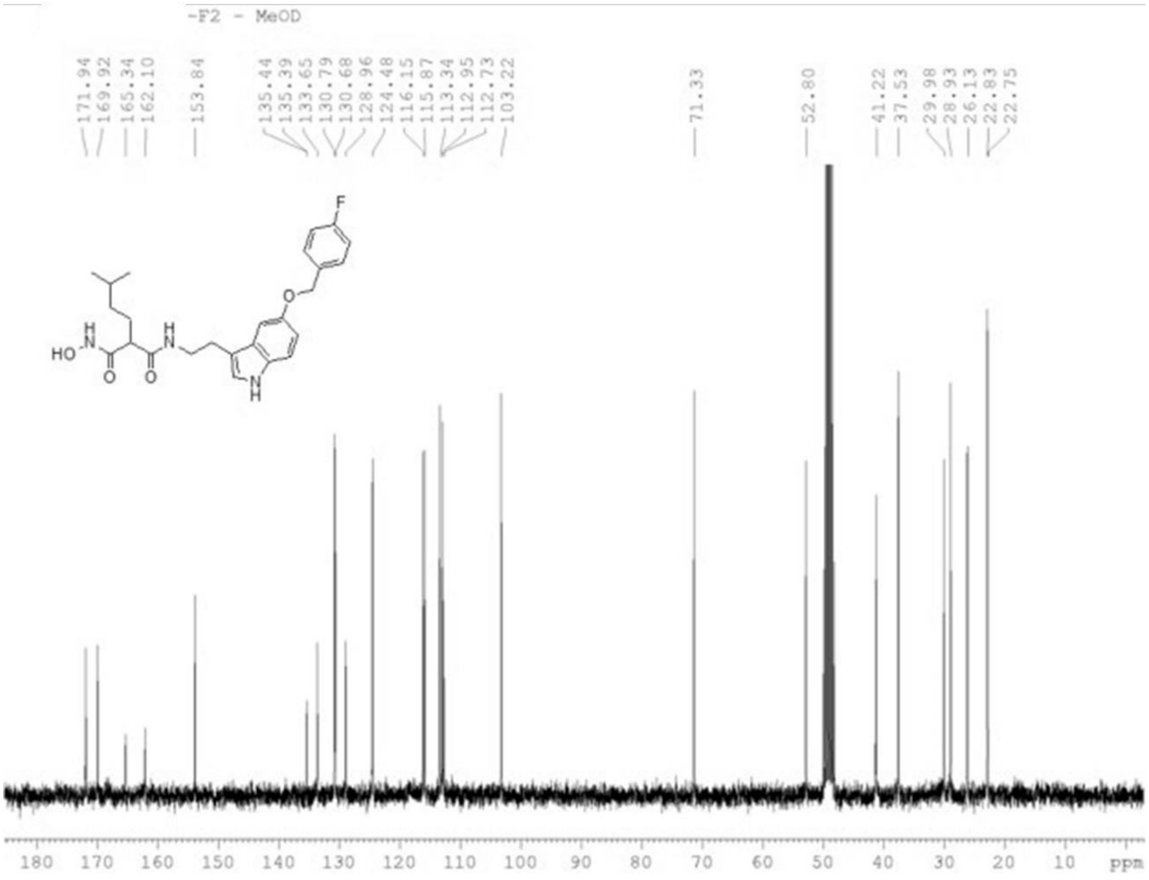
-F2 - MeOD
171.94
169.92
165.34
162.10
153.84
135.44
135.39
133.65
130.79
130.68
128.96
124.48
116.15
115.87
113.34
112.95
112.73
103.22
71.33
52.80
41.22
37.53
29.98
28.93
26.13
22.83
22.75
F
HO
H
N
O
O
H
N
O
N
H
180
170
160
150
140
130
120
110
100
90
80
70
60
50
40
30
20
10
ppm

Compound 26

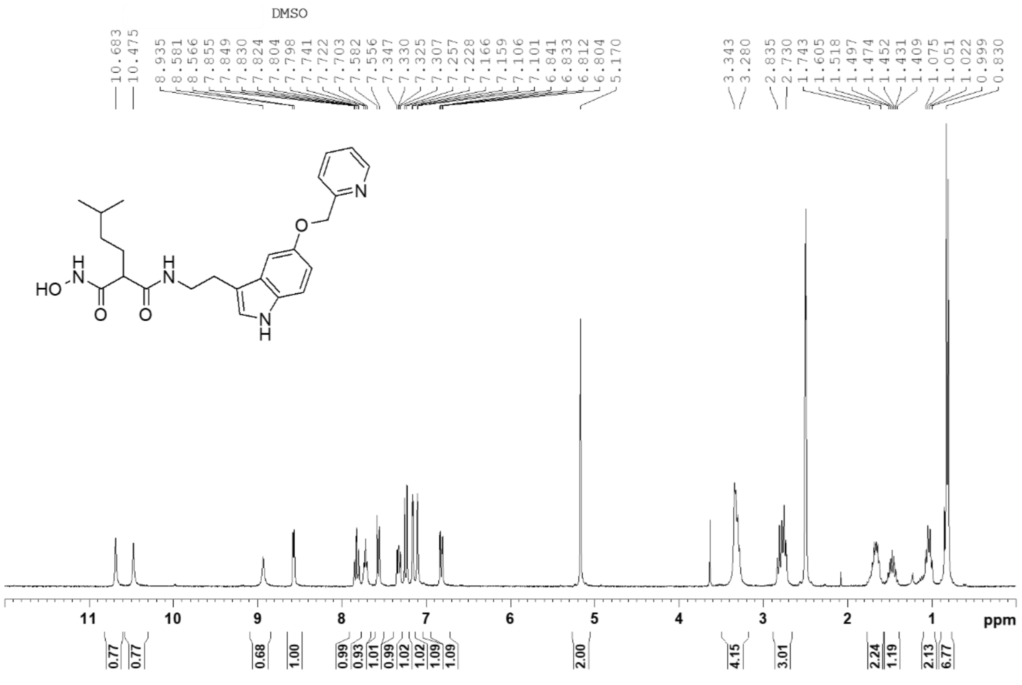

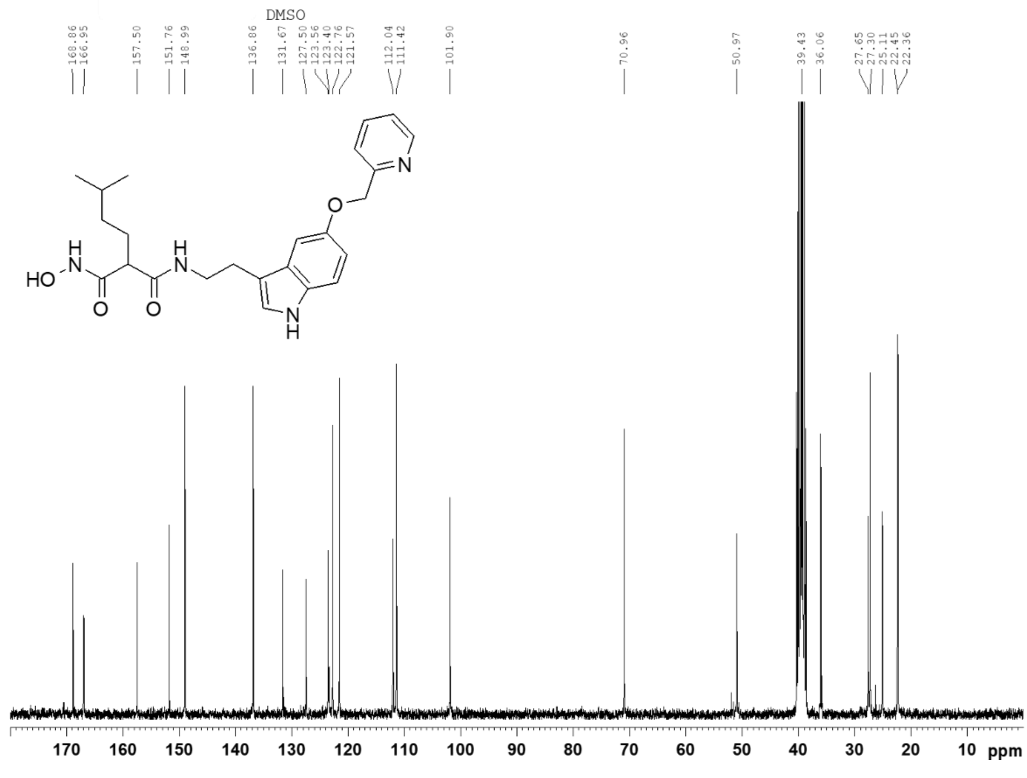

Compound 27

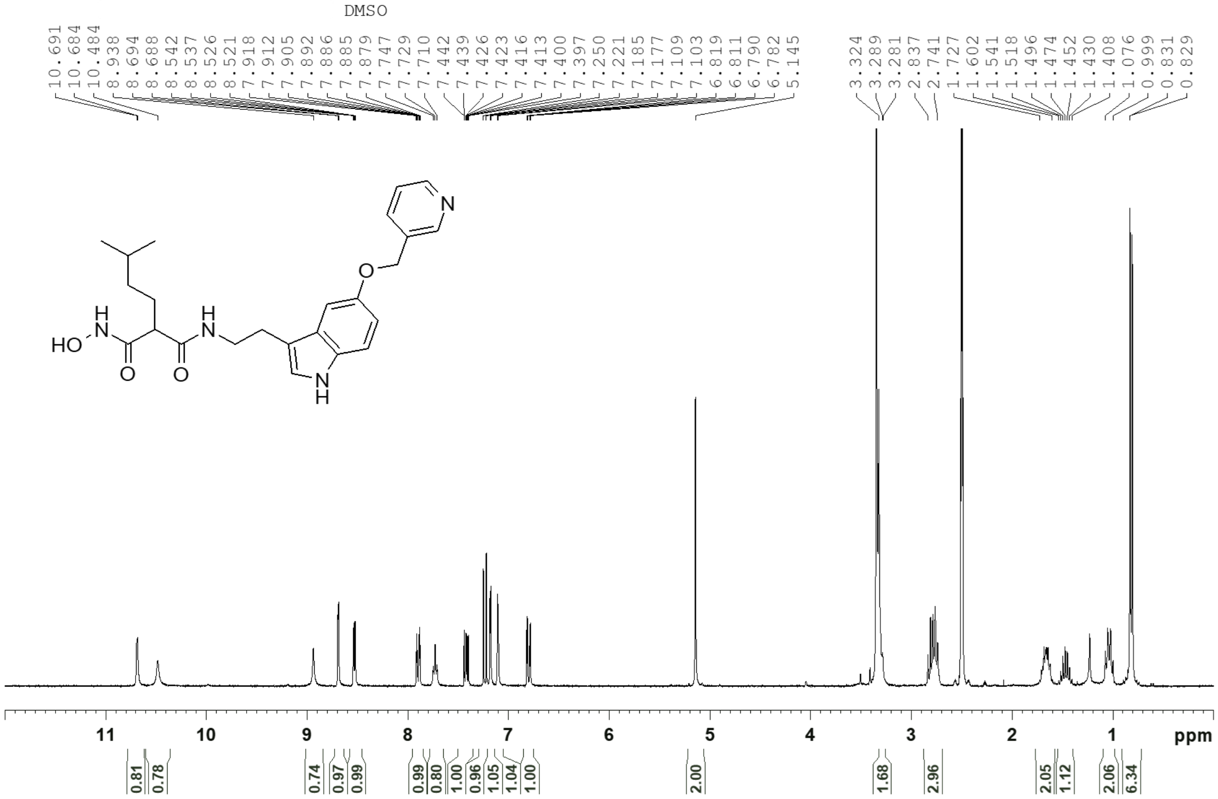

DMSO
10.691
10.684
10.484
8.938
8.694
8.688
8.542
8.537
8.526
8.521
7.918
7.912
7.905
7.892
7.886
7.885
7.879
7.747
7.729
7.710
7.442
7.439
7.426
7.423
7.416
7.413
7.400
7.397
7.250
7.221
7.185
7.177
7.109
7.103
6.819
6.811
6.790
6.782
5.145
3.324
3.289
3.281
2.837
2.741
1.727
1.602
1.541
1.518
1.496
1.474
1.452
1.430
1.408
1.076
0.999
0.831
0.829
HO
N
H
O
O
N
H
N
H
O
N
11
10
9
8
7
6
5
4
3
2
1
ppm
0.81
0.78
0.74
0.97
0.99
0.99
0.80
1.00
0.96
1.05
1.04
1.00
2.00
1.68
2.96
2.05
1.12
2.06
6.34

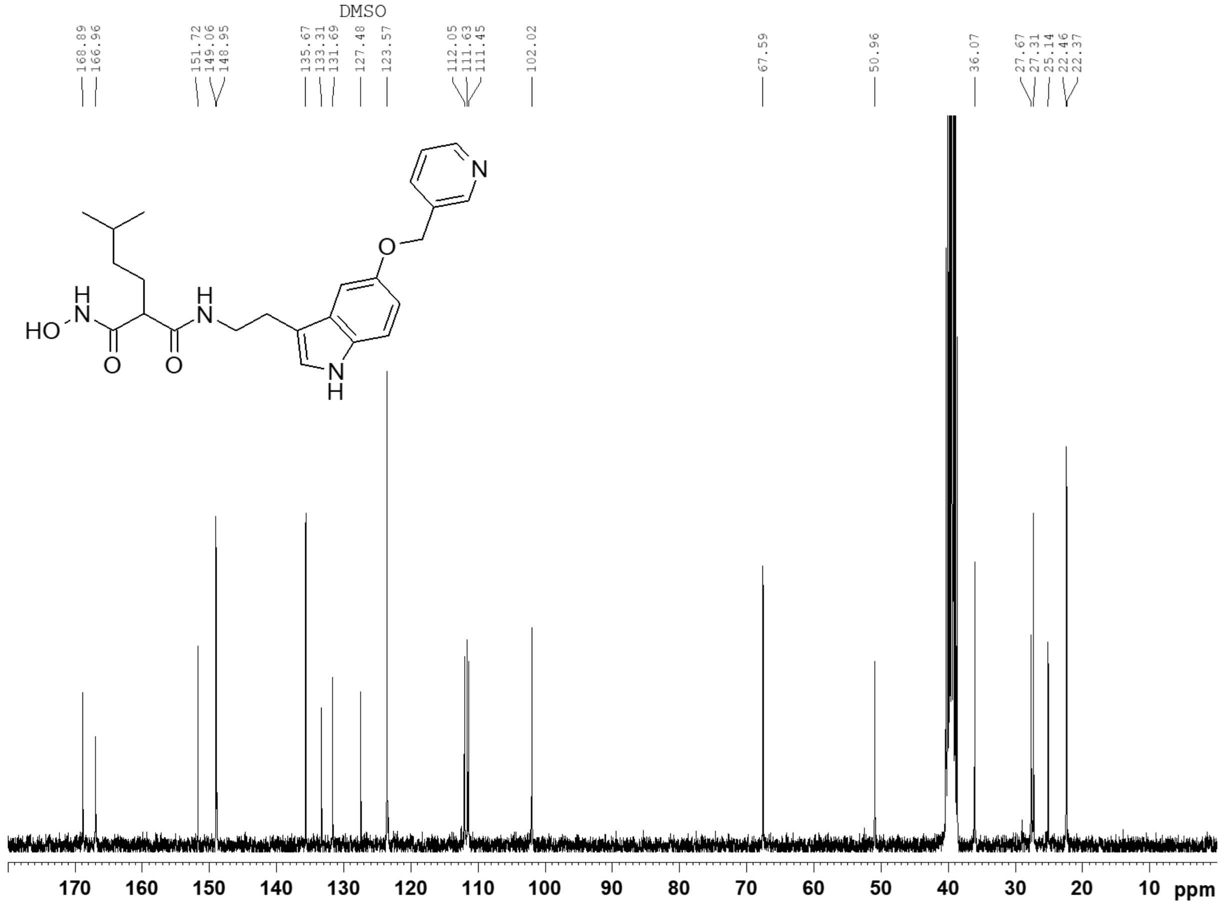

DMSO
168.89
166.96
151.72
149.06
148.95
135.67
133.31
131.69
127.48
123.57
112.05
111.63
111.45
102.02
67.59
50.96
36.07
27.67
27.31
25.14
22.46
22.37
HO
N
H
O
O
N
H
N
H
O
N
170
160
150
140
130
120
110
100
90
80
70
60
50
40
30
20
10
ppm

Compound 28

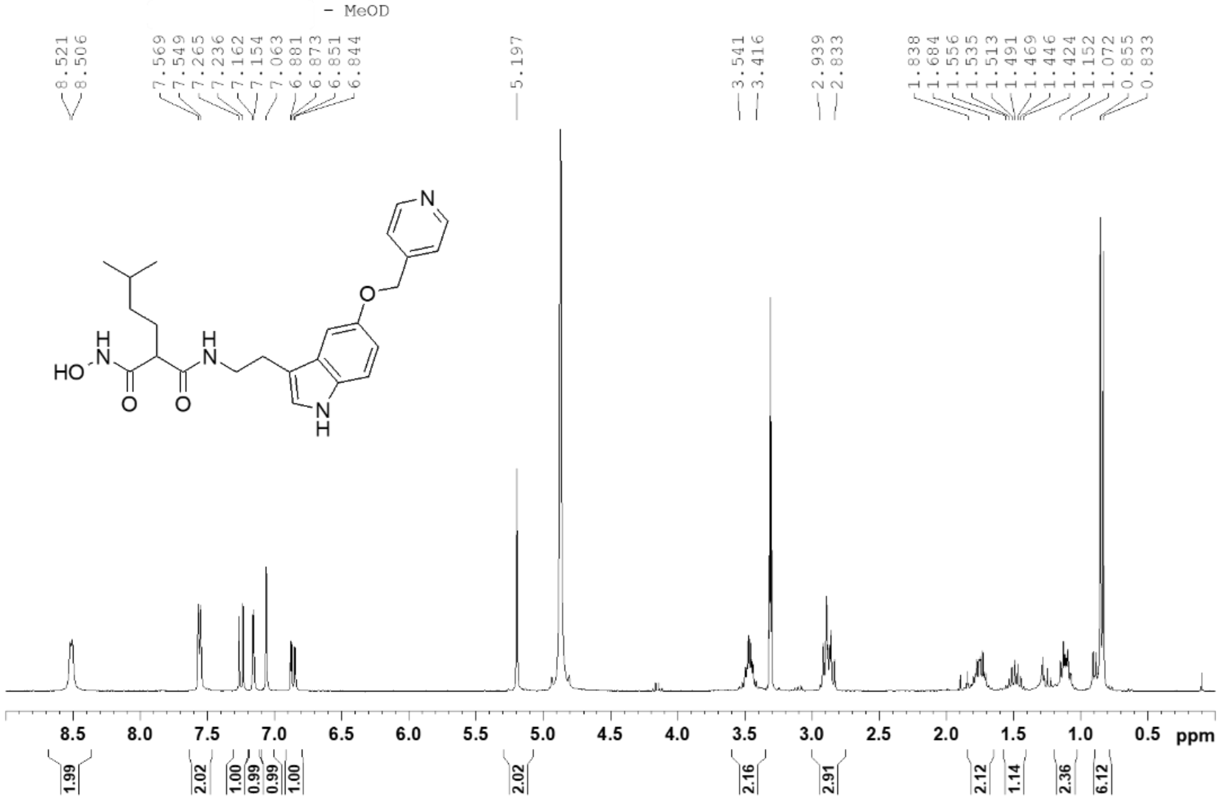
MeOD
8.521
8.506
7.569
7.549
7.265
7.236
7.162
7.154
7.063
6.881
6.873
6.851
6.844
5.197
3.541
3.416
2.939
2.833
1.838
1.684
1.556
1.535
1.513
1.491
1.469
1.446
1.424
1.152
1.072
0.855
0.833
HO
N
H
O
O
N
H
N
H
N
O
8.5 8.0 7.5 7.0 6.5 6.0 5.5 5.0 4.5 4.0 3.5 3.0 2.5 2.0 1.5 1.0 0.5 ppm
1.99
2.02
1.00
0.99
0.99
1.00
2.02
2.16
2.91
2.12
1.14
2.36
6.12

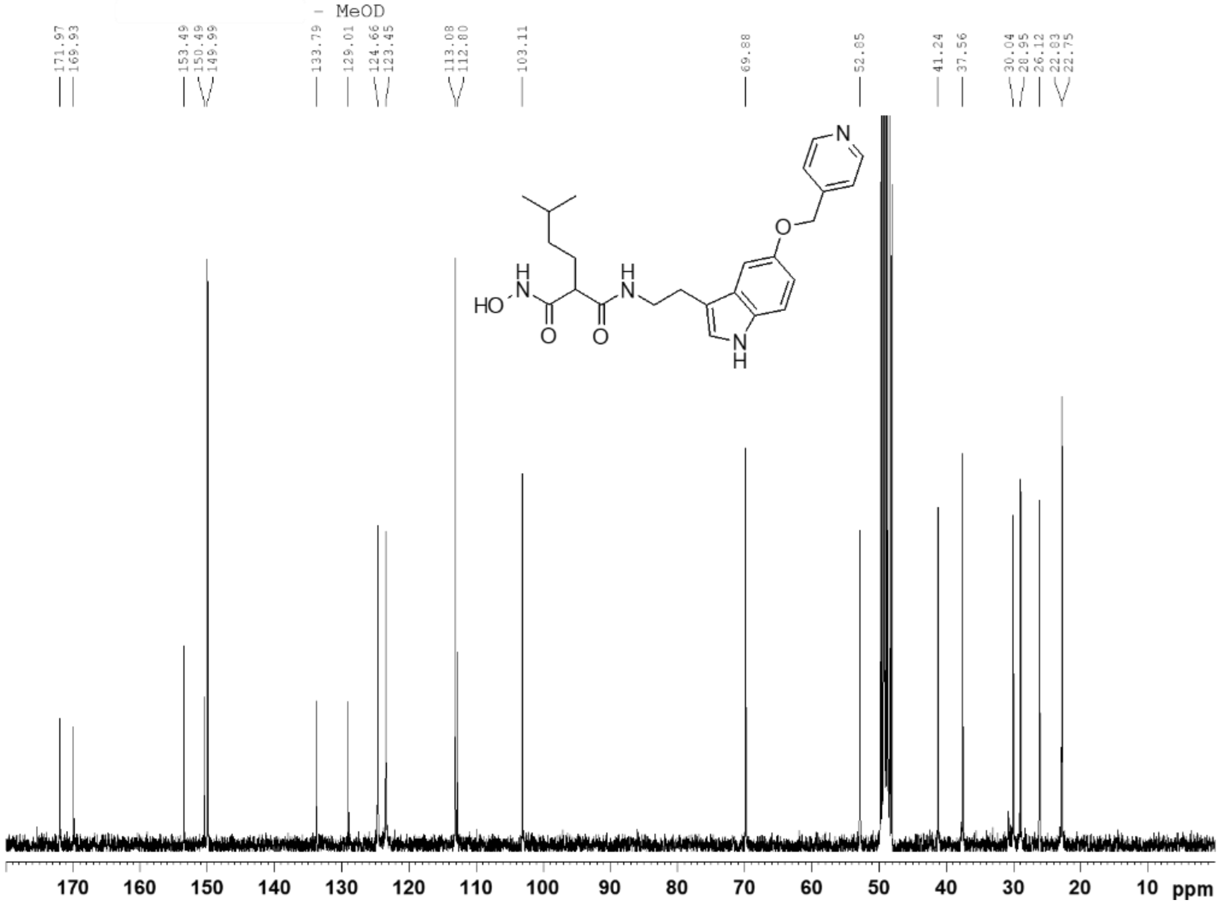
MeOD
171.97
169.93
153.49
150.49
149.99
133.79
129.01
124.66
123.45
113.08
112.80
103.11
69.88
52.85
41.24
37.56
30.04
28.95
26.12
22.83
22.75
HO
N
H
O
O
N
H
N
H
N
O
170 160 150 140 130 120 110 100 90 80 70 60 50 40 30 20 10 ppm

Compound 29

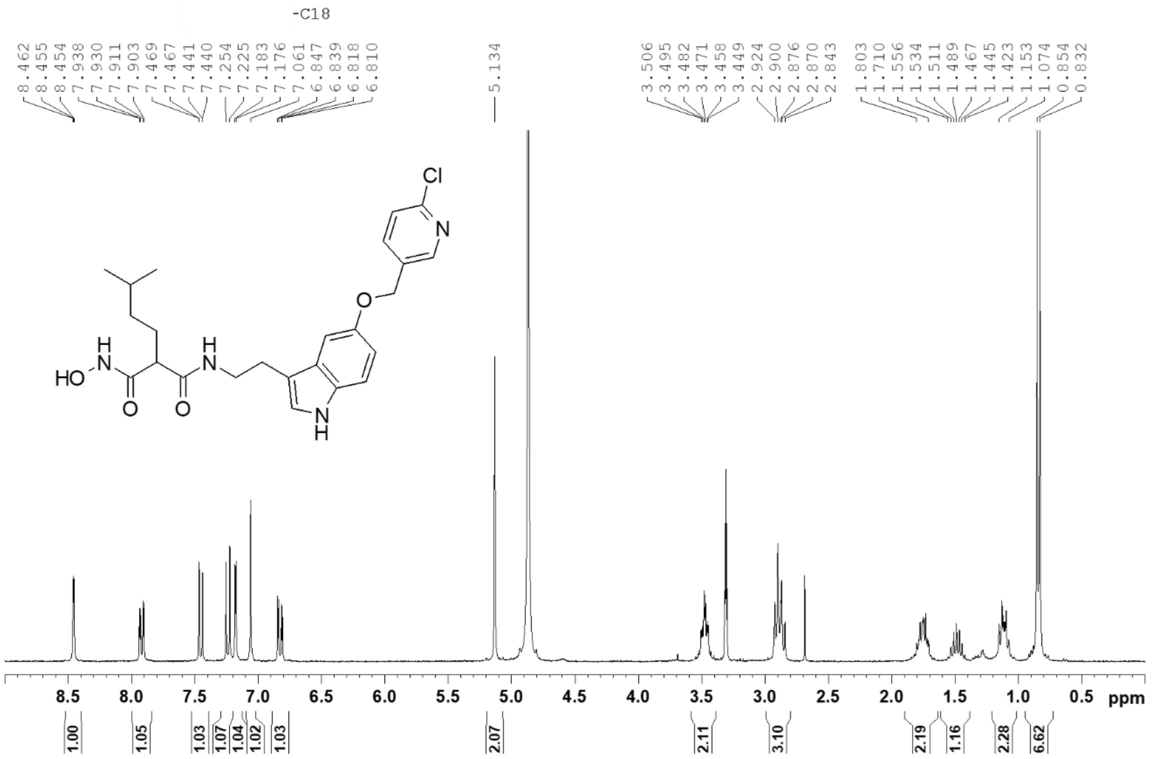

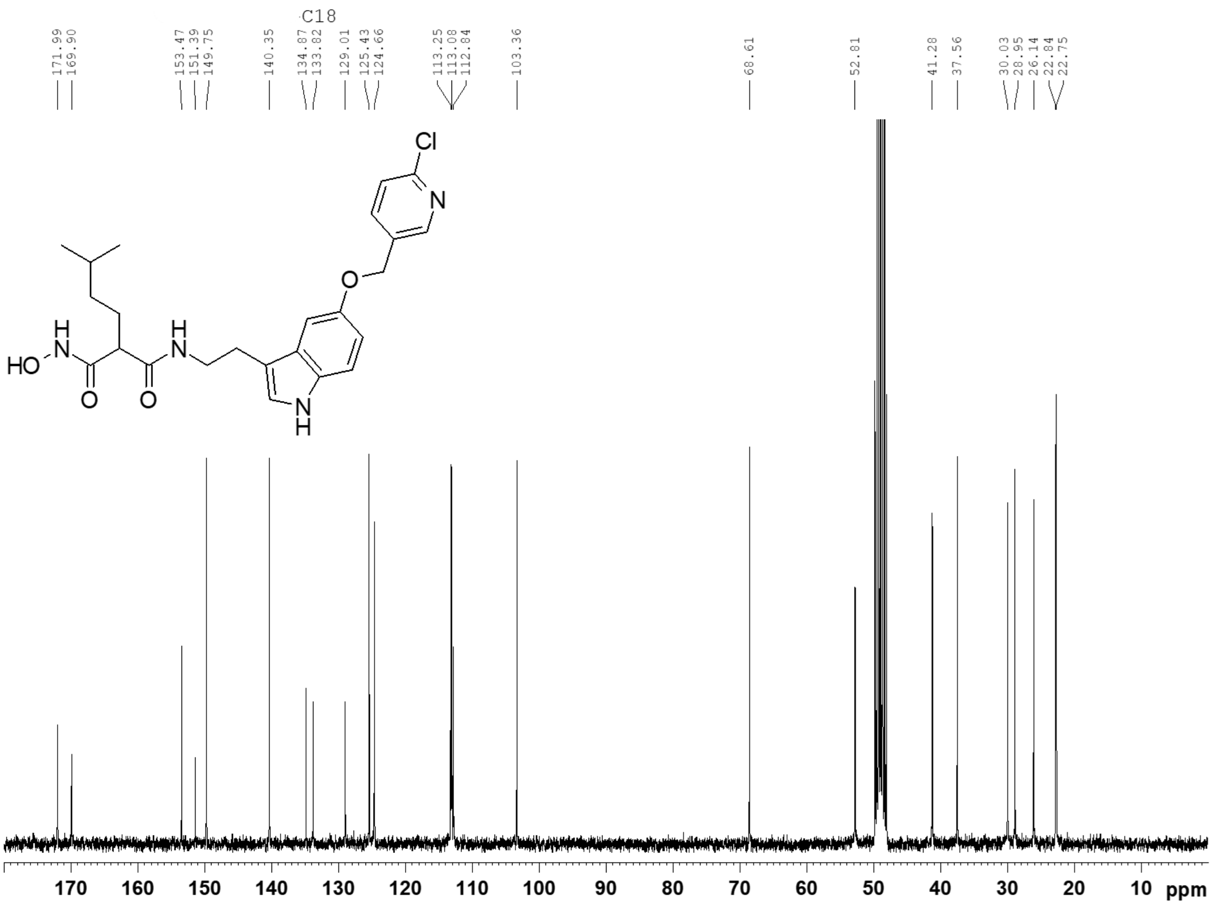

Compound 30

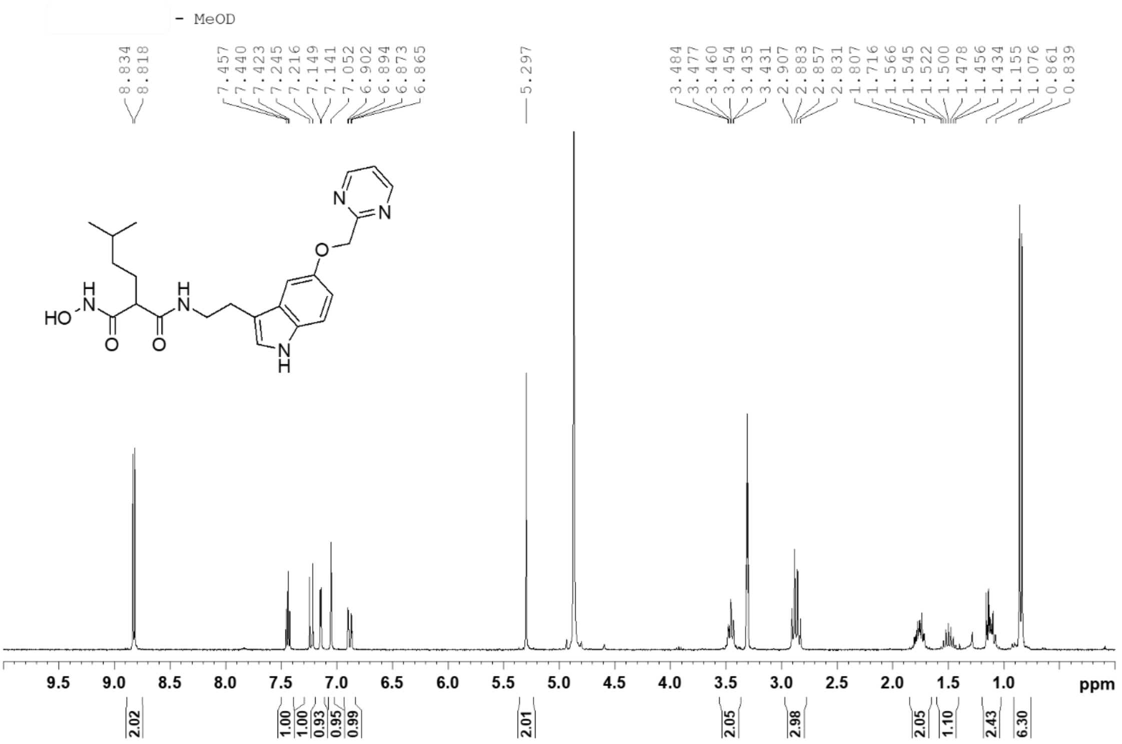
MeOD
8.834
8.818
7.457
7.440
7.423
7.245
7.216
7.149
7.141
7.052
6.902
6.894
6.873
6.865
5.297
3.484
3.477
3.460
3.454
3.435
3.431
2.907
2.883
2.857
2.831
1.807
1.716
1.566
1.545
1.522
1.500
1.478
1.456
1.434
1.155
1.076
0.861
0.839
HO
N
H
O
O
N
H
O
N
N
N
H
9.5
9.0
8.5
8.0
7.5
7.0
6.5
6.0
5.5
5.0
4.5
4.0
3.5
3.0
2.5
2.0
1.5
1.0
ppm
2.02
1.00
1.00
0.93
0.95
0.99
2.01
2.05
2.98
2.05
1.10
2.43
6.30

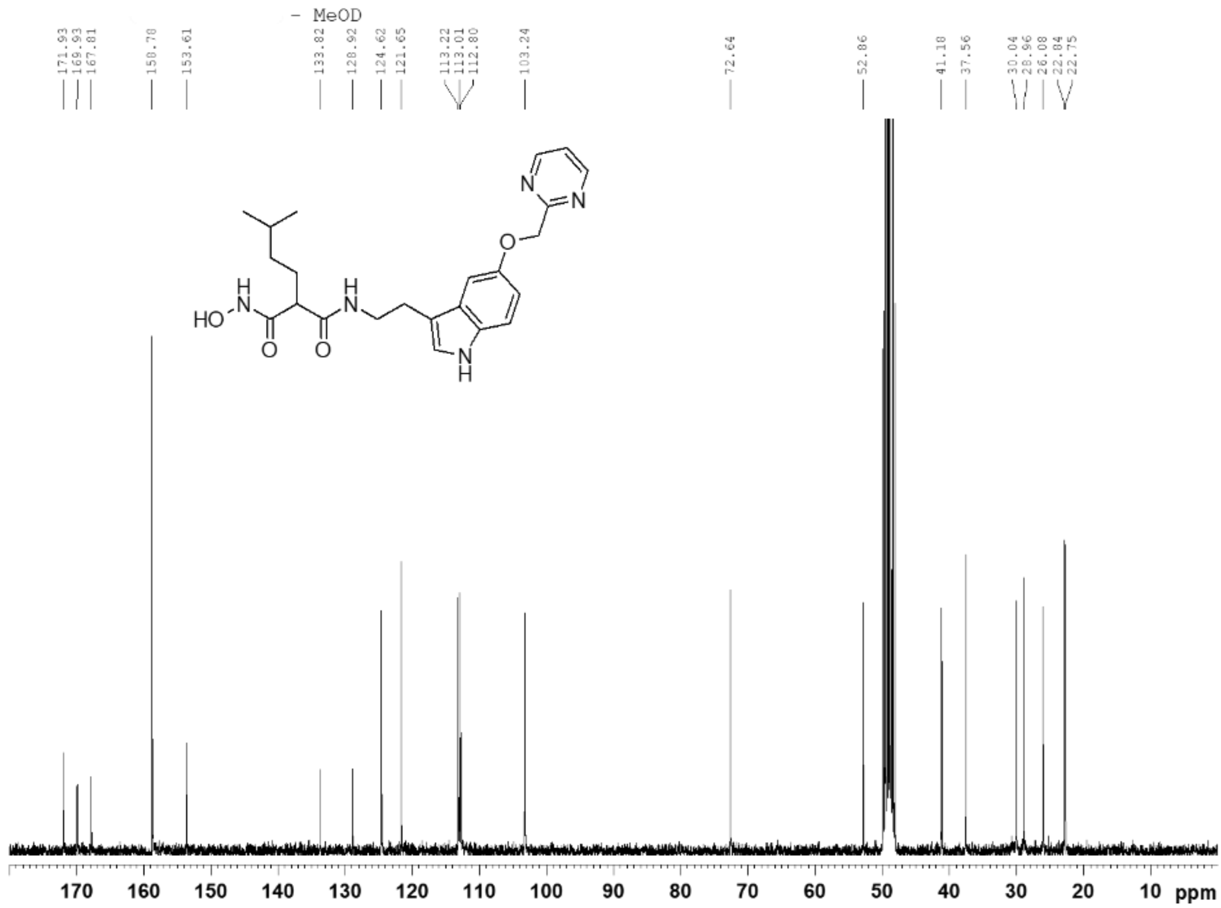
MeOD
171.93
169.93
167.81
158.78
153.61
133.82
128.92
124.62
121.65
113.22
113.01
112.80
103.24
72.64
52.86
41.18
37.56
30.04
28.96
26.08
22.84
22.75
HO
N
H
O
O
N
H
O
N
N
N
H
170
160
150
140
130
120
110
100
90
80
70
60
50
40
30
20
10
ppm

Compound 31

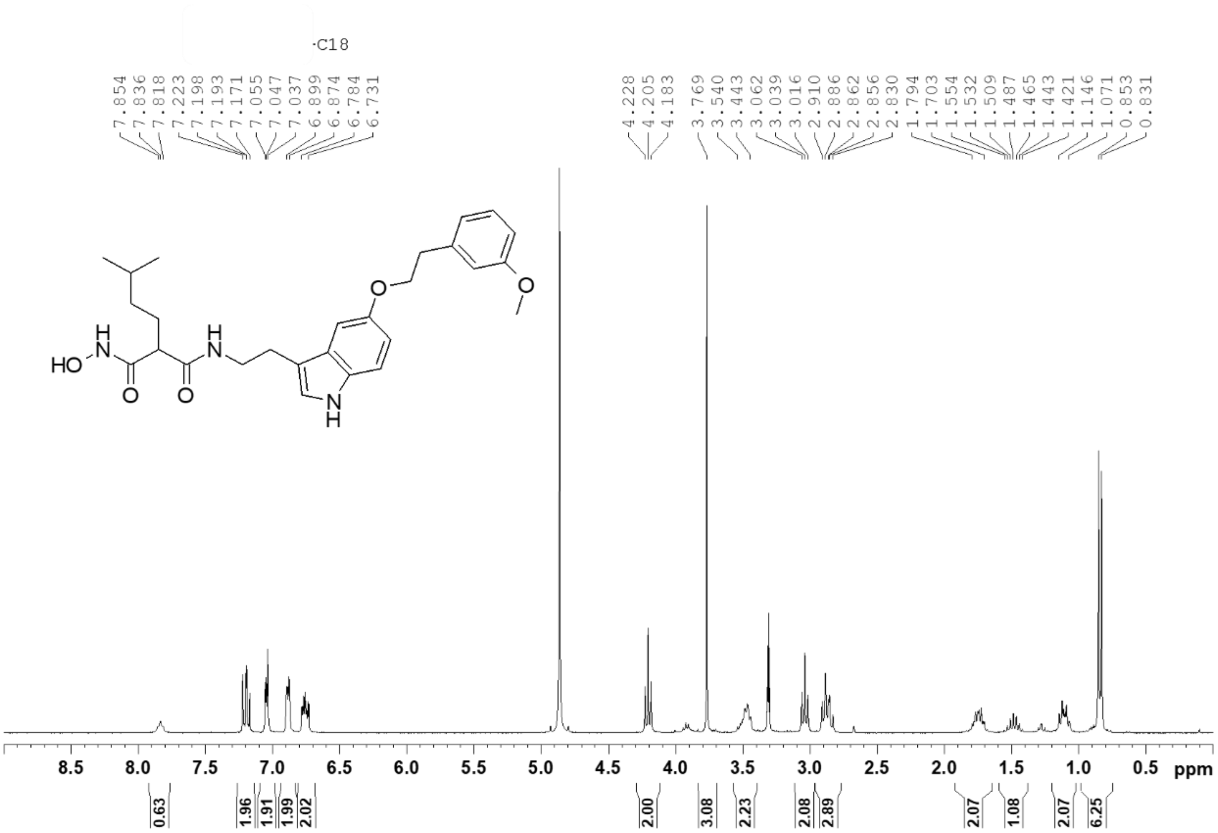

C18
7.854 7.836 7.818 7.223 7.198 7.193 7.171 7.055 7.047 7.037 6.899 6.874 6.784 6.731
4.228 4.205 4.183 3.769 3.540 3.443 3.062 3.039 3.016 2.910 2.886 2.862 2.856 2.830 1.794 1.703 1.554 1.532 1.509 1.487 1.465 1.443 1.421 1.146 1.071 0.853 0.831
HO
H
N
O
O
H
N
O
O
N
H
8.5 8.0 7.5 7.0 6.5 6.0 5.5 5.0 4.5 4.0 3.5 3.0 2.5 2.0 1.5 1.0 0.5 ppm
0.63 1.96 1.91 1.99 2.02 2.00 3.08 2.23 2.08 2.89 2.07 1.08 2.07 6.25

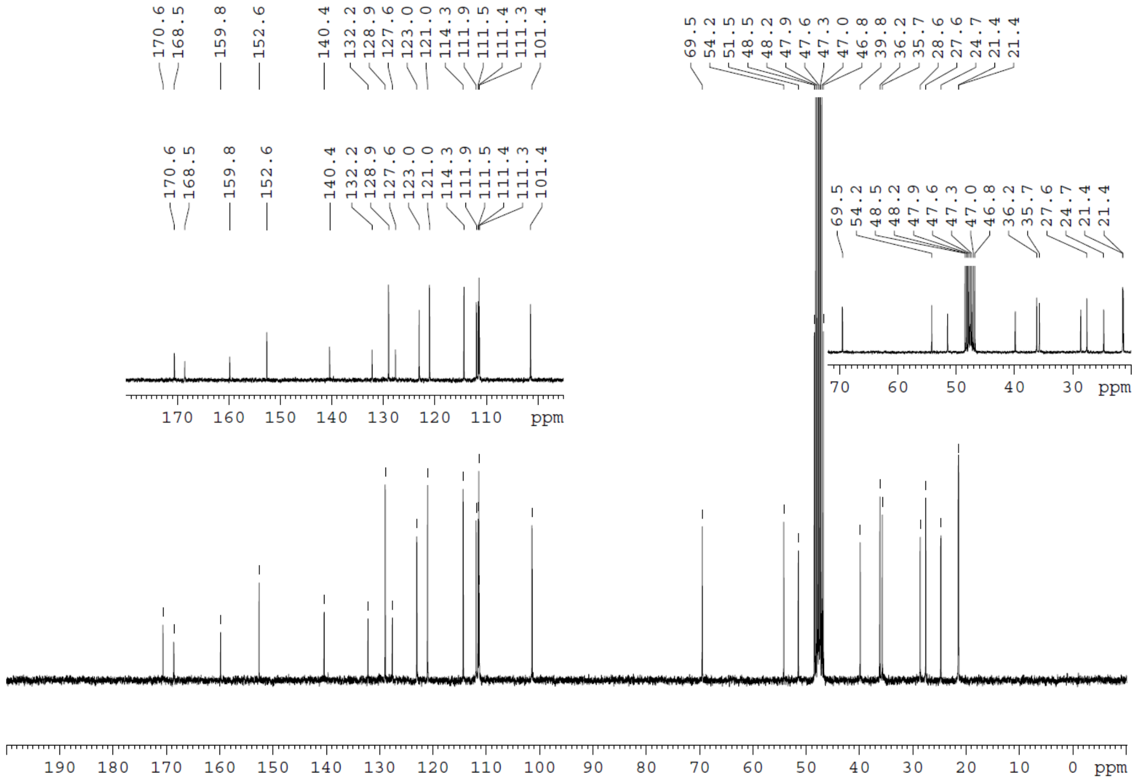

170.6 168.5 159.8 152.6 140.4 132.2 128.9 127.6 123.0 121.0 114.3 111.9 111.5 111.4 111.3 101.4
69.5 54.2 51.5 48.5 48.2 47.9 47.6 47.3 47.0 46.8 39.8 36.2 35.7 28.6 27.6 24.7 21.4 21.4
170 160 150 140 130 120 110 ppm
70 60 50 40 30 ppm
190 180 170 160 150 140 130 120 110 100 90 80 70 60 50 40 30 20 10 0 ppm

Compound 32

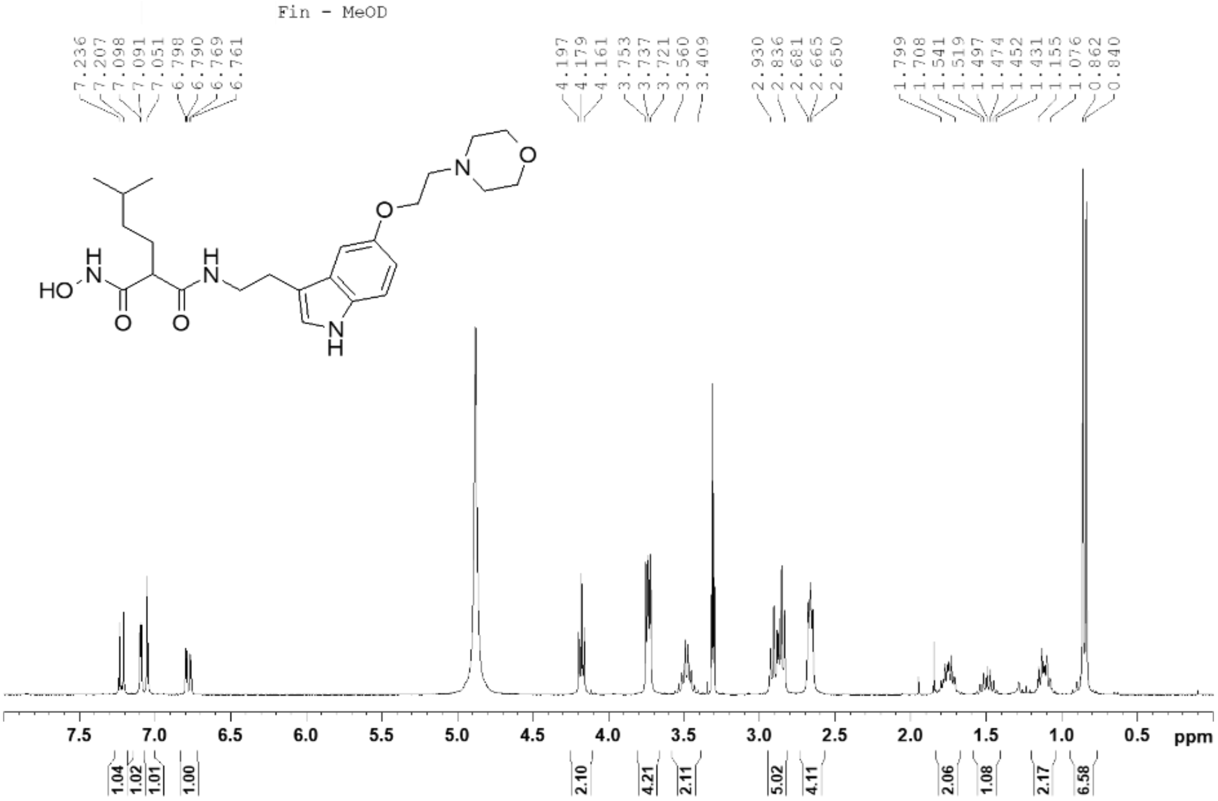

Fin - MeOD
7.236
7.207
7.098
7.091
7.051
6.798
6.790
6.769
6.761
4.197
4.179
4.161
3.753
3.737
3.721
3.560
3.409
2.930
2.836
2.681
2.665
2.650
1.799
1.708
1.541
1.519
1.497
1.474
1.452
1.431
1.155
1.076
0.862
0.840
ppm
1.04
1.02
1.01
1.00
2.10
4.21
2.11
5.02
4.11
2.06
1.08
2.17
6.58

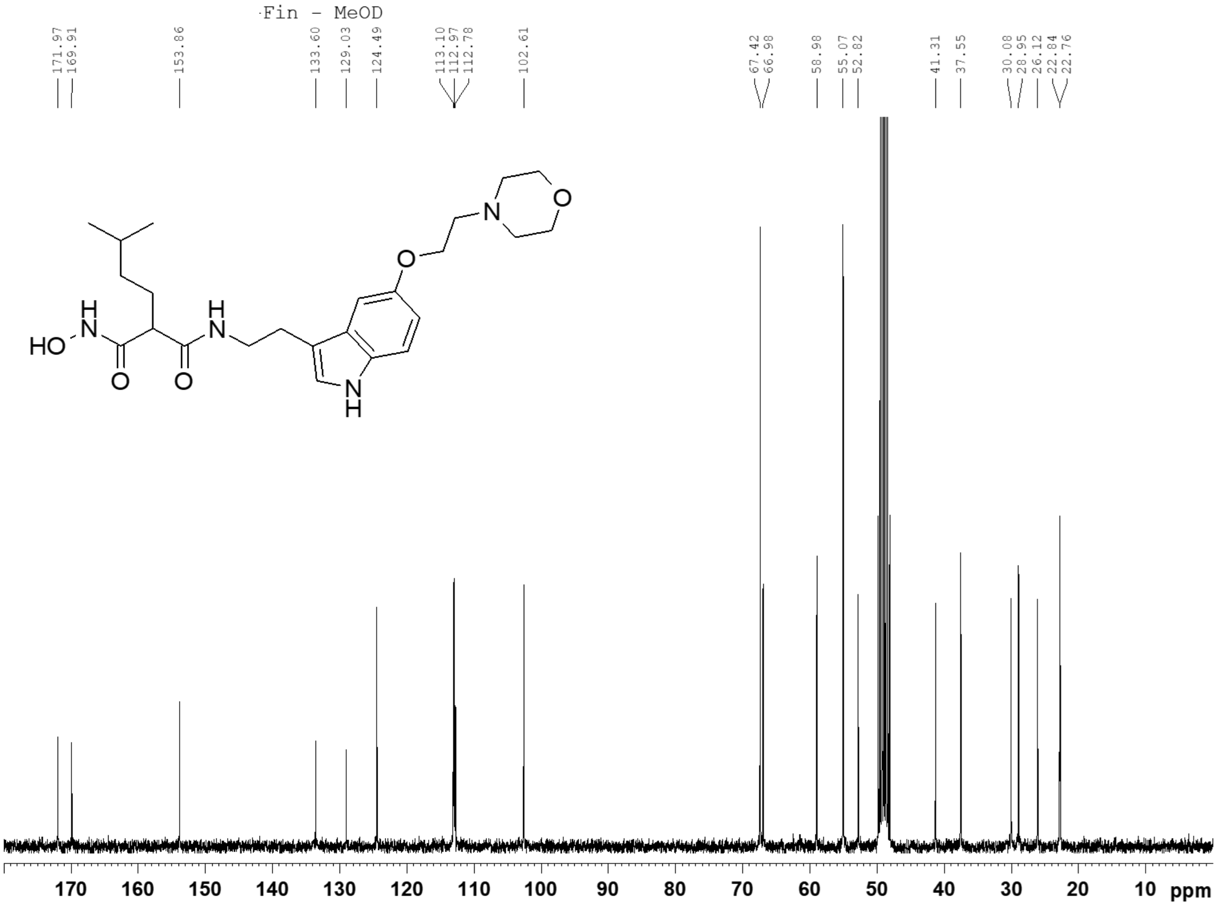

Fin - MeOD
171.97
169.91
153.86
133.60
129.03
124.49
113.10
112.97
112.78
102.61
67.42
66.98
58.98
55.07
52.82
41.31
37.55
30.08
28.95
26.12
22.84
22.76
ppm

Compound 33

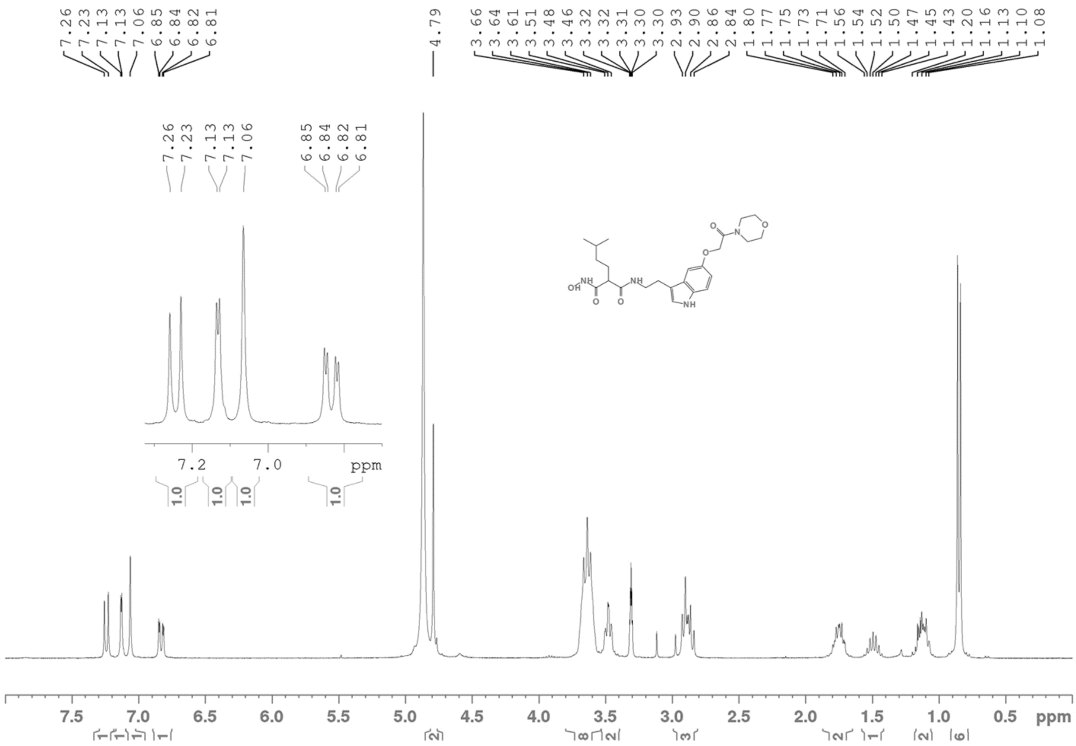

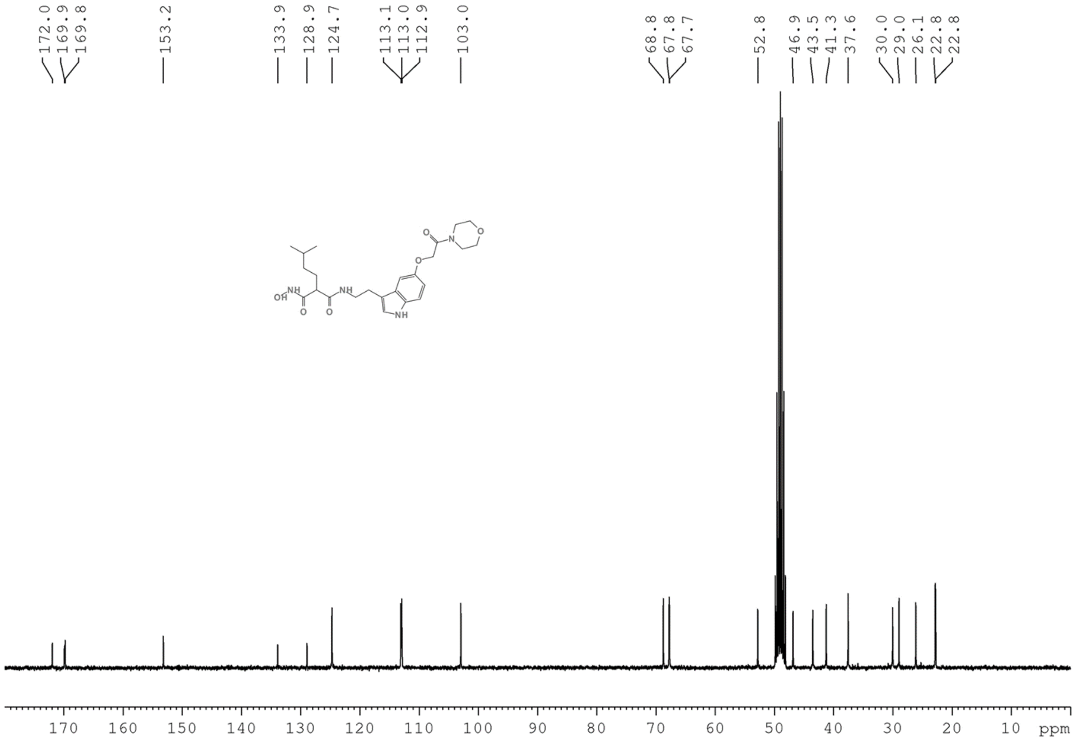

Compound 34

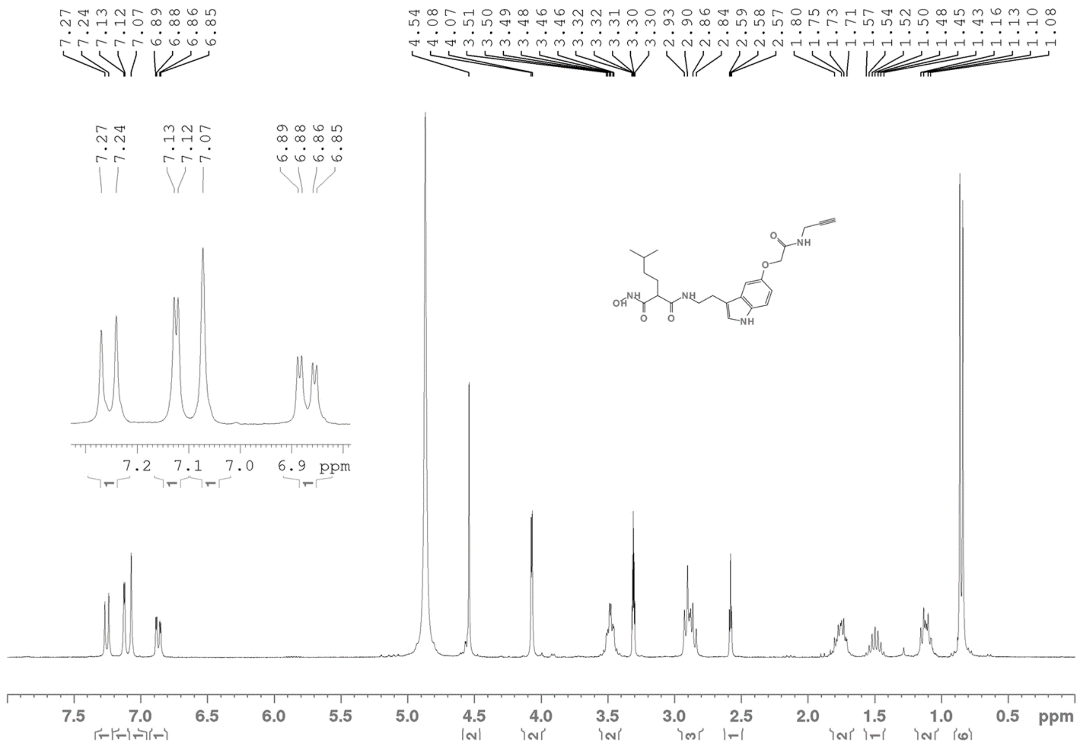
7.27 7.24 7.13 7.12 7.07 6.89 6.88 6.86 6.85
4.54 4.08 4.07 3.51 3.50 3.49 3.48 3.46 3.46 3.32 3.32 3.31 3.30 3.30 2.93 2.90 2.86 2.84 2.59 2.58 2.57 1.80 1.75 1.73 1.71 1.57 1.54 1.52 1.50 1.48 1.45 1.43 1.16 1.13 1.10 1.08
7.2 7.1 7.0 6.9 ppm
NH
OH
O
7.5 7.0 6.5 6.0 5.5 5.0 4.5 4.0 3.5 3.0 2.5 2.0 1.5 1.0 0.5 ppm

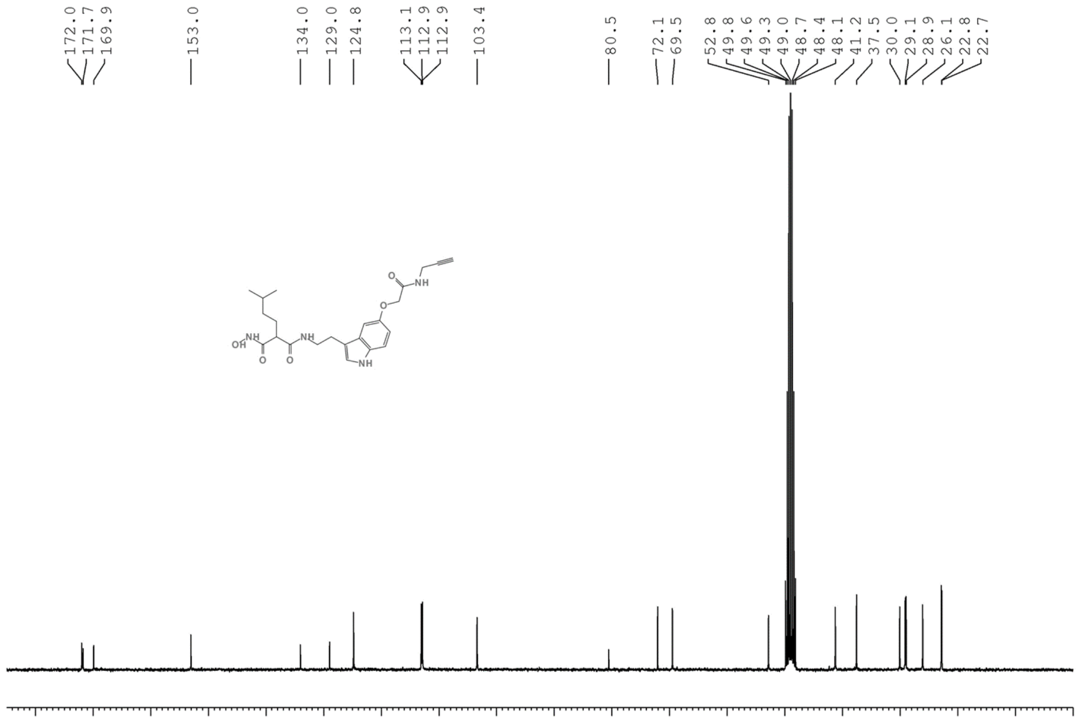
172.0 171.7 169.9 153.0 134.0 129.0 124.8 113.1 112.9 112.9 103.4 80.5 72.1 69.5 52.8 49.8 49.6 49.3 49.0 48.7 48.4 48.1 41.2 37.5 30.0 29.1 28.9 26.1 22.8 22.7
180 170 160 150 140 130 120 110 100 90 80 70 60 50 40 30 20 10 ppm

Compound 35

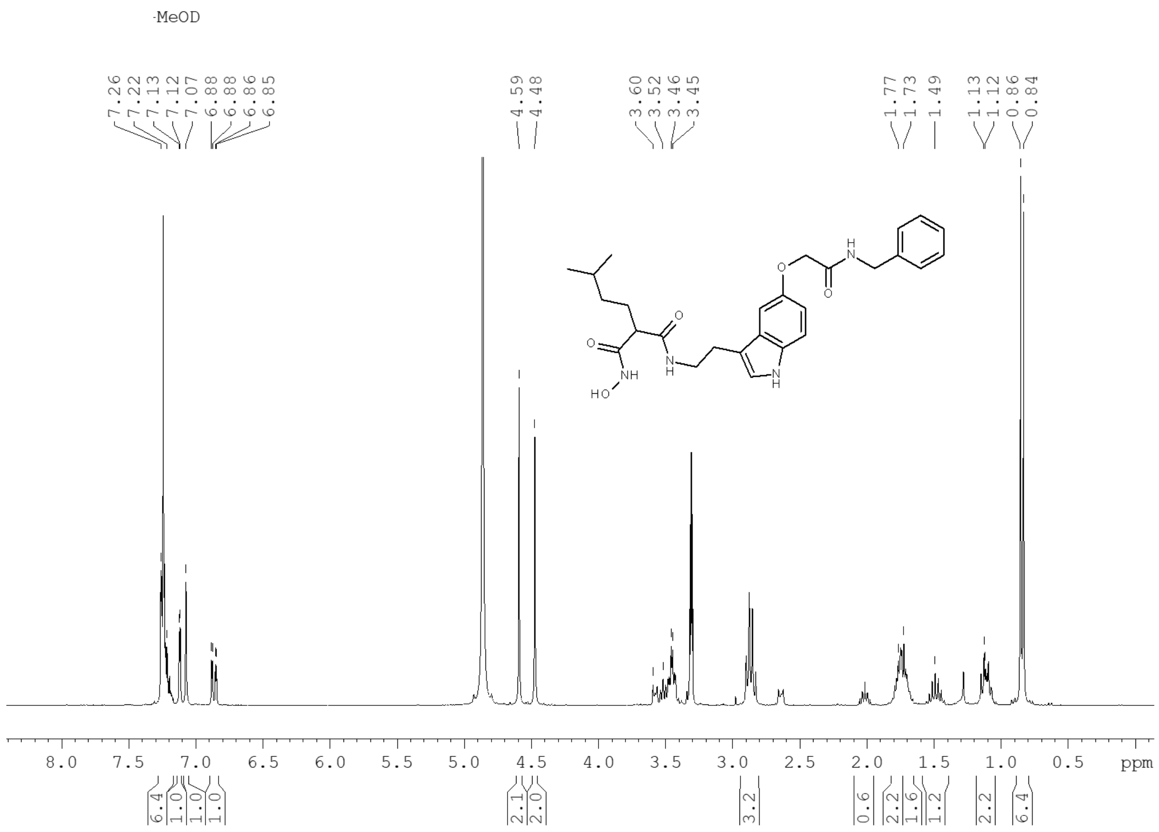

MeOD
7.26
7.22
7.13
7.12
7.07
6.88
6.88
6.86
6.85
4.59
4.48
3.60
3.52
3.46
3.45
1.77
1.73
1.49
1.13
1.12
0.86
0.84
8.0 7.5 7.0 6.5 6.0 5.5 5.0 4.5 4.0 3.5 3.0 2.5 2.0 1.5 1.0 0.5 ppm
6.4 1.0 1.0 1.0 2.1 2.0 3.2 0.6 2.2 1.6 1.2 2.2 6.4

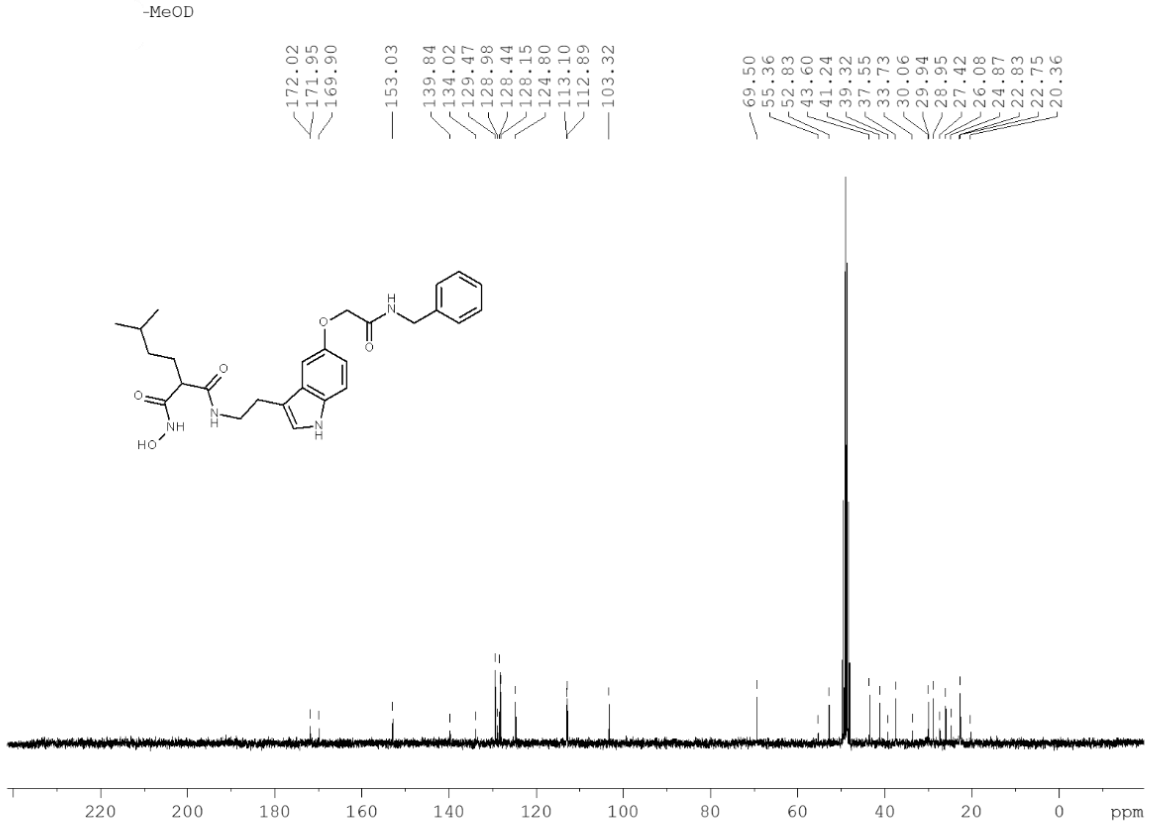

MeOD
172.02
171.95
169.90
153.03
139.84
134.02
129.47
128.98
128.44
128.15
124.80
113.10
112.89
103.32
69.50
55.36
52.83
43.60
41.24
39.32
37.55
33.73
30.06
29.94
28.95
27.42
26.08
24.87
22.83
22.75
20.36
220 200 180 160 140 120 100 80 60 40 20 0 ppm

DEPT-135 spectrum of compound 35

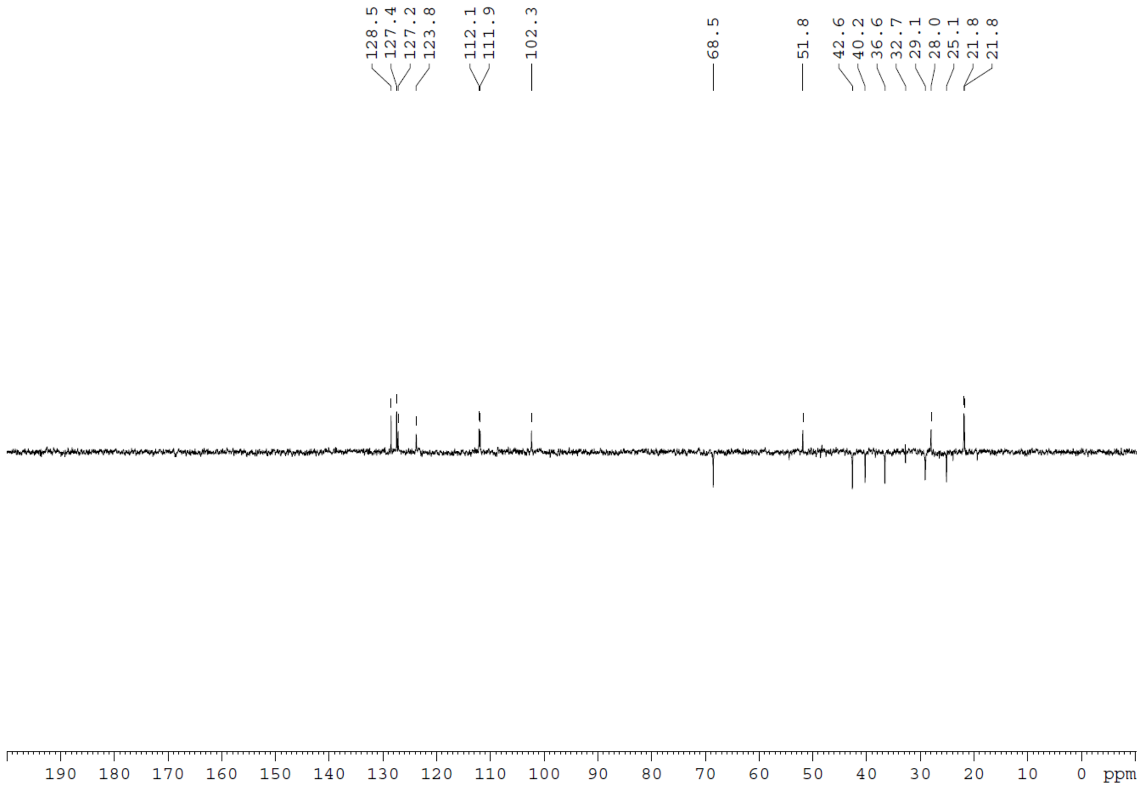

Compound 36

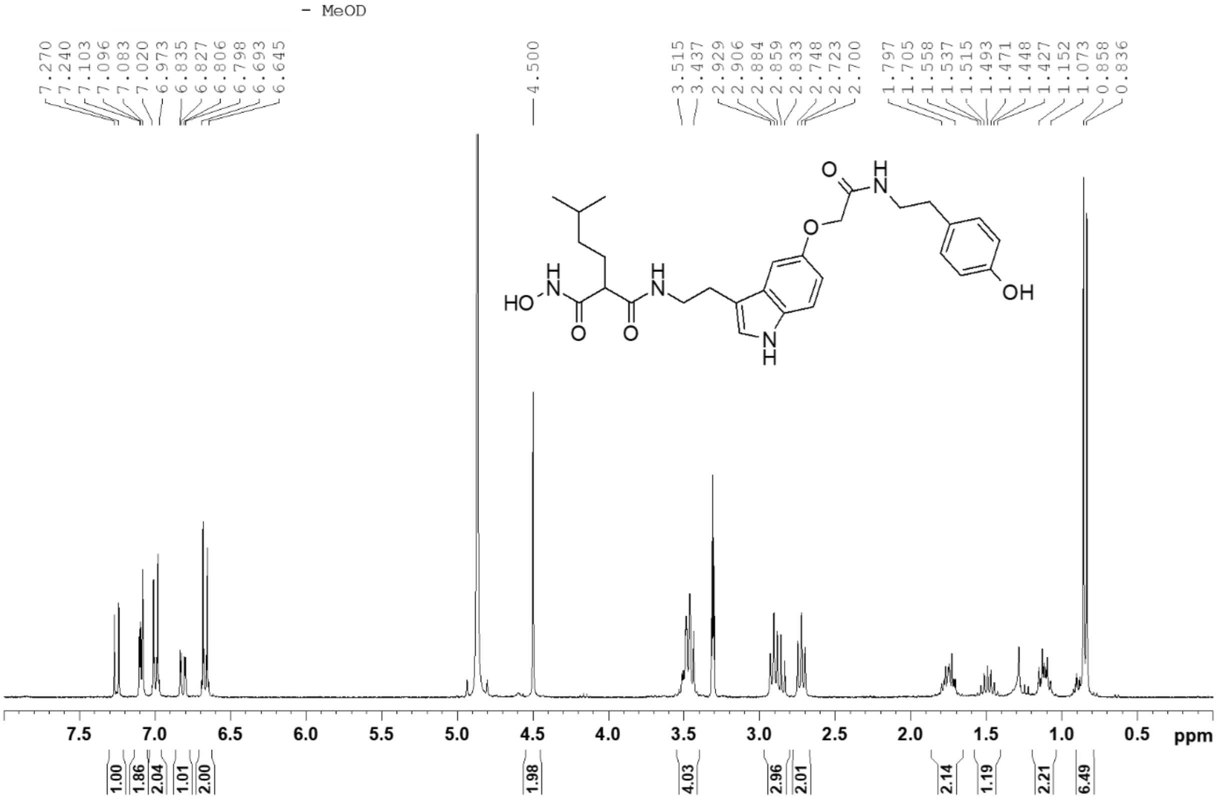

MeOD
7.270
7.240
7.096
7.083
7.020
6.973
6.835
6.827
6.806
6.798
6.693
6.645
4.500
3.515
3.437
2.929
2.906
2.884
2.859
2.833
2.748
2.723
2.700
1.797
1.705
1.558
1.537
1.515
1.493
1.471
1.448
1.427
1.152
1.073
0.858
0.836
HO
N
H
O
O
N
H
O
O
N
H
OH
N
H
7.5 7.0 6.5 6.0 5.5 5.0 4.5 4.0 3.5 3.0 2.5 2.0 1.5 1.0 0.5 ppm
1.00
1.86
2.04
1.01
2.00
1.98
4.03
2.96
2.01
2.14
1.19
2.21
6.49

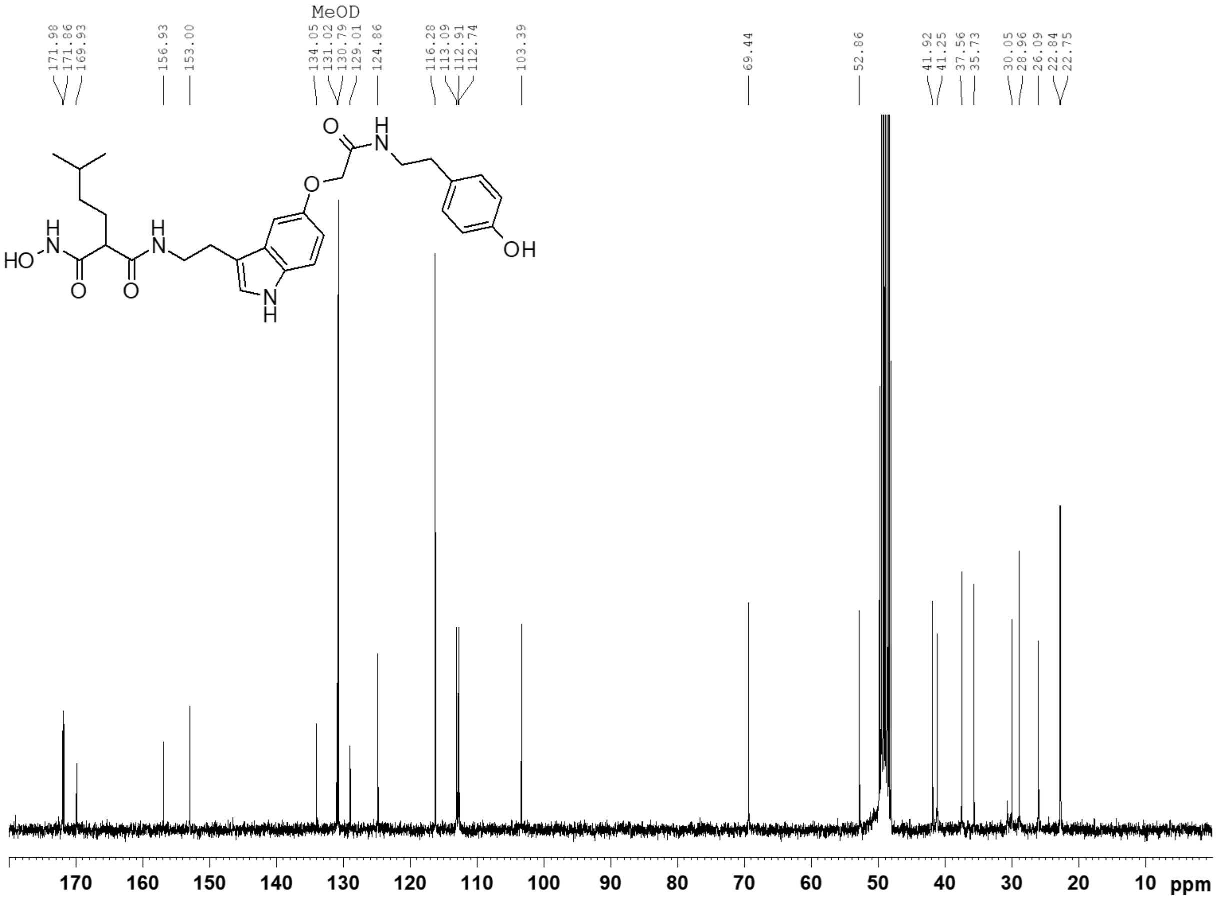

MeOD
171.98
171.86
169.93
156.93
153.00
134.05
131.02
130.79
129.01
124.86
116.28
113.09
112.91
112.74
103.39
69.44
52.86
41.92
41.25
37.56
35.73
30.05
28.96
26.09
22.84
22.75
170 160 150 140 130 120 110 100 90 80 70 60 50 40 30 20 10 ppm

Compound 37

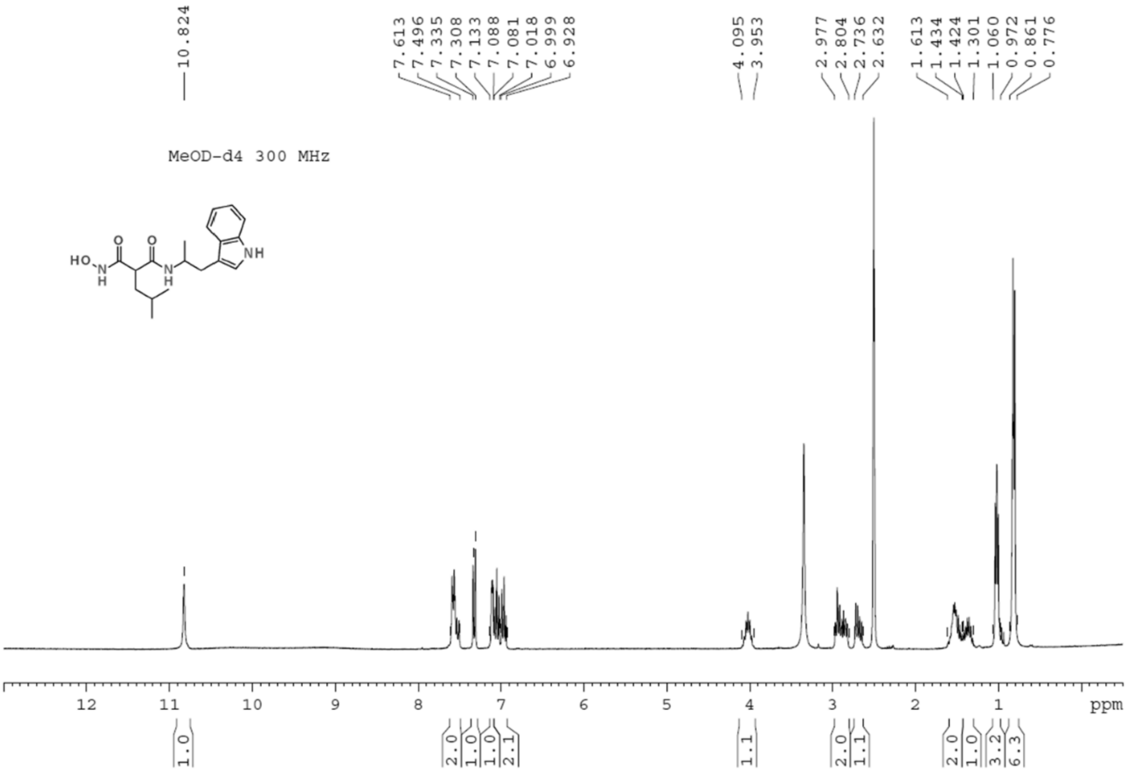
10.824
7.613
7.496
7.335
7.308
7.133
7.088
7.081
7.018
6.999
6.928
4.095
3.953
2.977
2.804
2.736
2.632
1.613
1.434
1.424
1.301
1.060
0.972
0.861
0.776
MeOD-d4 300 MHz
HO
N
H
O
O
N
H
NH
12 11 10 9 8 7 6 5 4 3 2 1 ppm
1.0
2.0
1.0
1.0
2.1
1.1
2.0
1.1
2.0
1.0
3.2
6.3

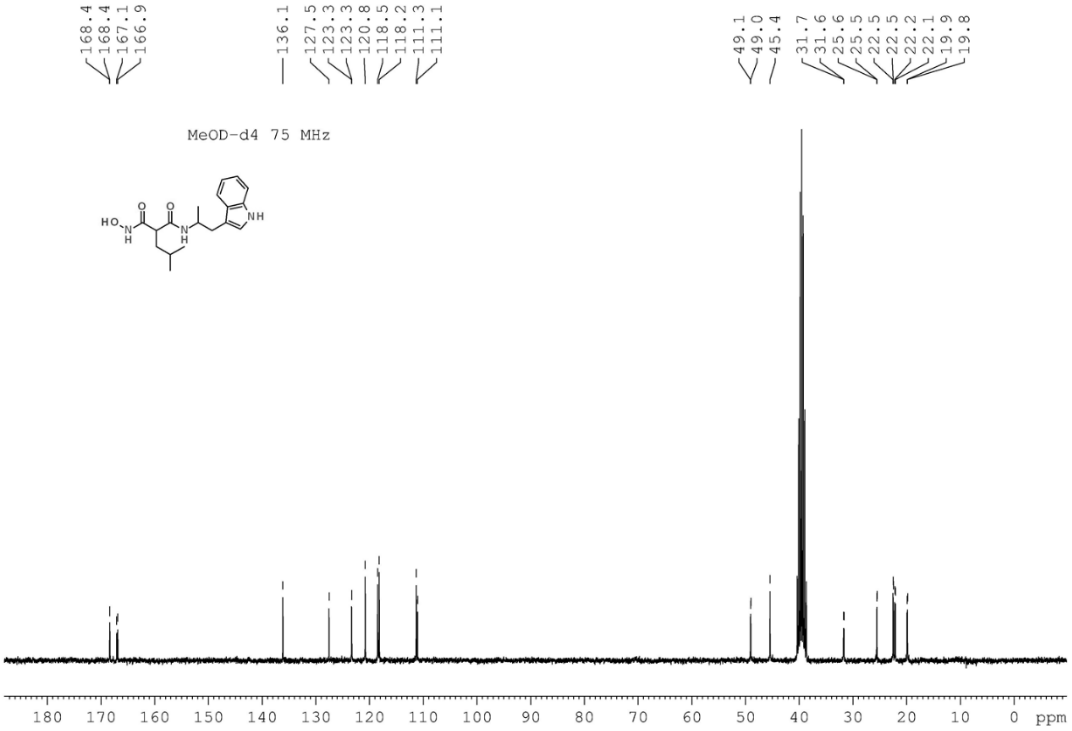
168.4
168.4
167.1
166.9
136.1
127.5
123.3
123.3
120.8
118.5
118.2
111.3
111.1
49.1
49.0
45.4
31.7
31.6
25.6
25.5
22.5
22.5
22.2
22.1
19.9
19.8
MeOD-d4 75 MHz
HO
N
H
O
O
N
H
NH
180 170 160 150 140 130 120 110 100 90 80 70 60 50 40 30 20 10 0 ppm

Compound 38

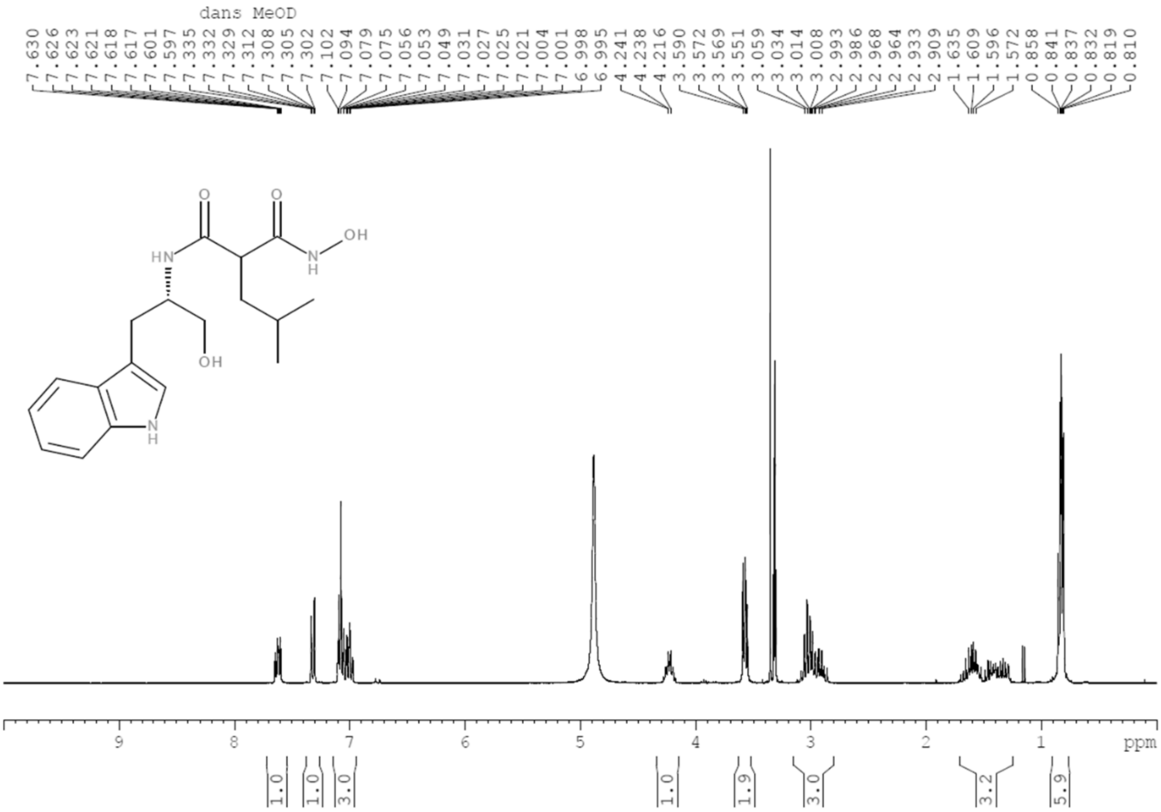
dans MeOD
7.630
7.626
7.623
7.621
7.618
7.617
7.601
7.597
7.335
7.332
7.329
7.312
7.308
7.305
7.302
7.102
7.094
7.079
7.075
7.056
7.053
7.049
7.031
7.027
7.025
7.021
7.004
7.001
6.998
6.995
4.241
4.238
4.216
3.590
3.572
3.569
3.551
3.059
3.034
3.014
3.008
2.993
2.986
2.968
2.964
2.933
2.909
1.635
1.609
1.596
1.572
0.858
0.841
0.837
0.832
0.819
0.810
O
O
HN
N
OH
H
OH
N
H
9
8
7
6
5
4
3
2
1
ppm
1.0
1.0
3.0
1.0
1.9
3.0
3.2
5.9

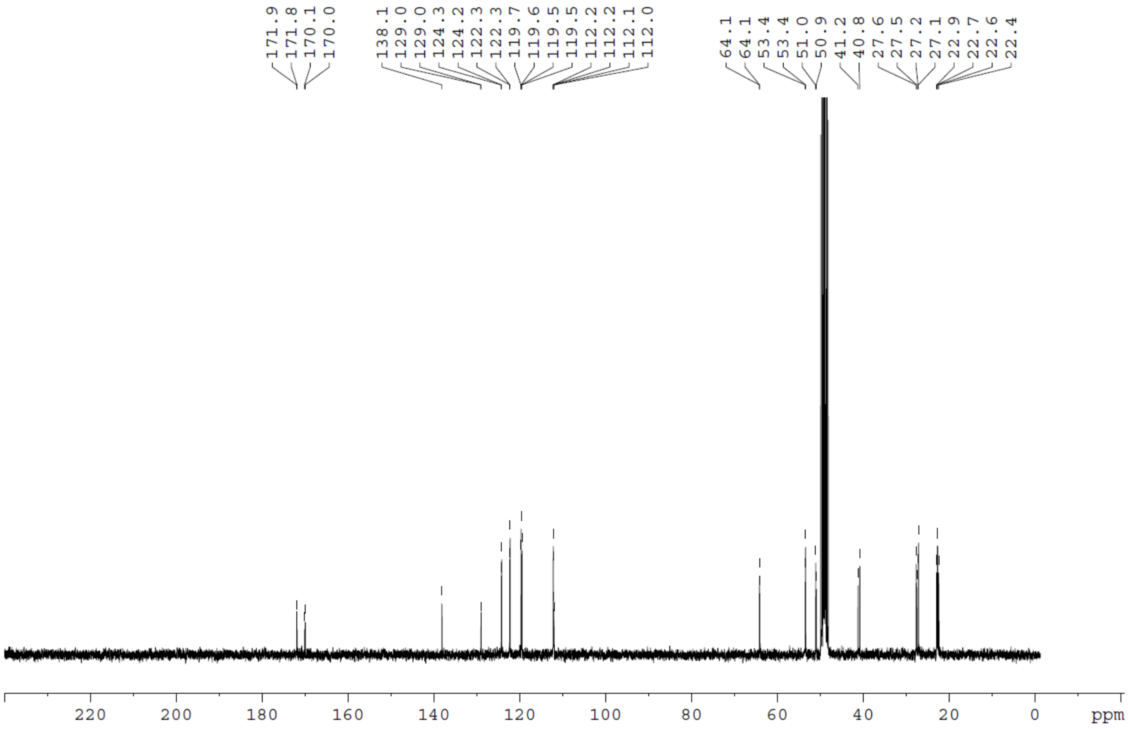
171.9
171.8
170.1
170.0
138.1
129.0
129.0
124.3
124.2
122.3
122.3
119.7
119.6
119.5
119.5
112.2
112.2
112.1
112.0
64.1
64.1
53.4
53.4
51.0
50.9
41.2
40.8
27.6
27.5
27.2
27.1
22.9
22.7
22.6
22.4
220
200
180
160
140
120
100
80
60
40
20
0
ppm

Compound 39

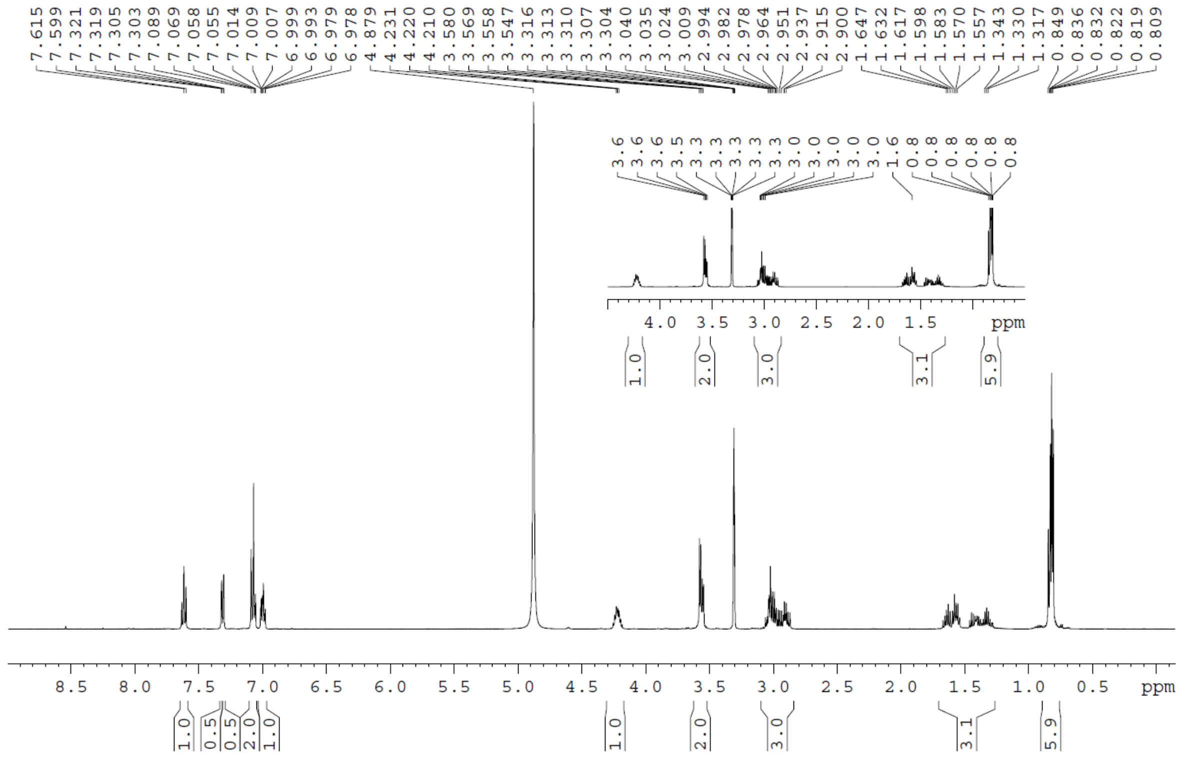
7.615
7.599
7.321
7.319
7.305
7.303
7.089
7.069
7.058
7.055
7.014
7.009
7.007
6.999
6.993
6.979
6.978
4.879
4.231
4.220
4.210
3.580
3.569
3.558
3.547
3.316
3.313
3.310
3.307
3.304
3.040
3.035
3.024
3.009
2.994
2.982
2.978
2.964
2.951
2.937
2.915
2.900
1.647
1.632
1.617
1.598
1.583
1.570
1.557
1.343
1.330
1.317
0.849
0.836
0.832
0.822
0.819
0.809
ppm

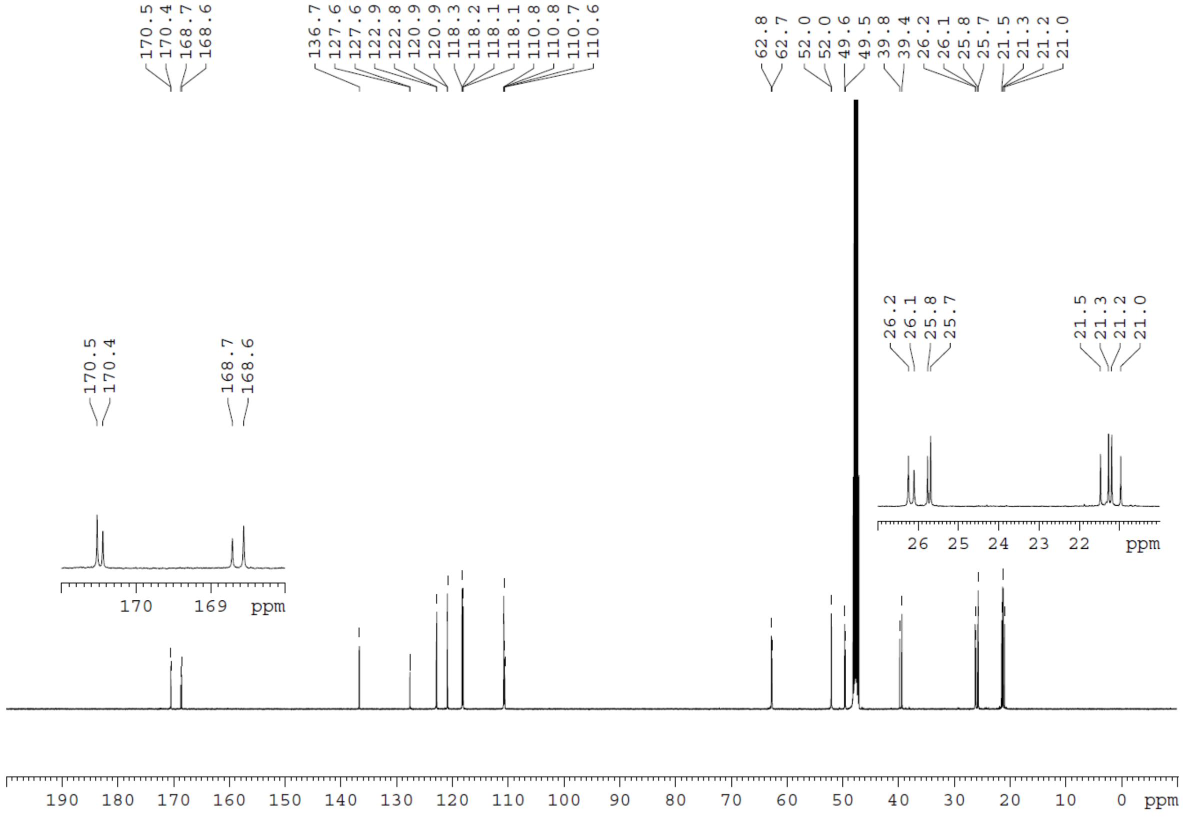
170.5
170.4
168.7
168.6
136.7
127.6
122.9
122.8
120.9
120.9
118.3
118.2
118.1
118.1
110.8
110.8
110.7
110.6
62.8
62.7
52.0
52.0
49.6
49.5
39.8
39.4
26.2
26.1
25.8
25.7
21.5
21.3
21.2
21.0
ppm

Compound 40

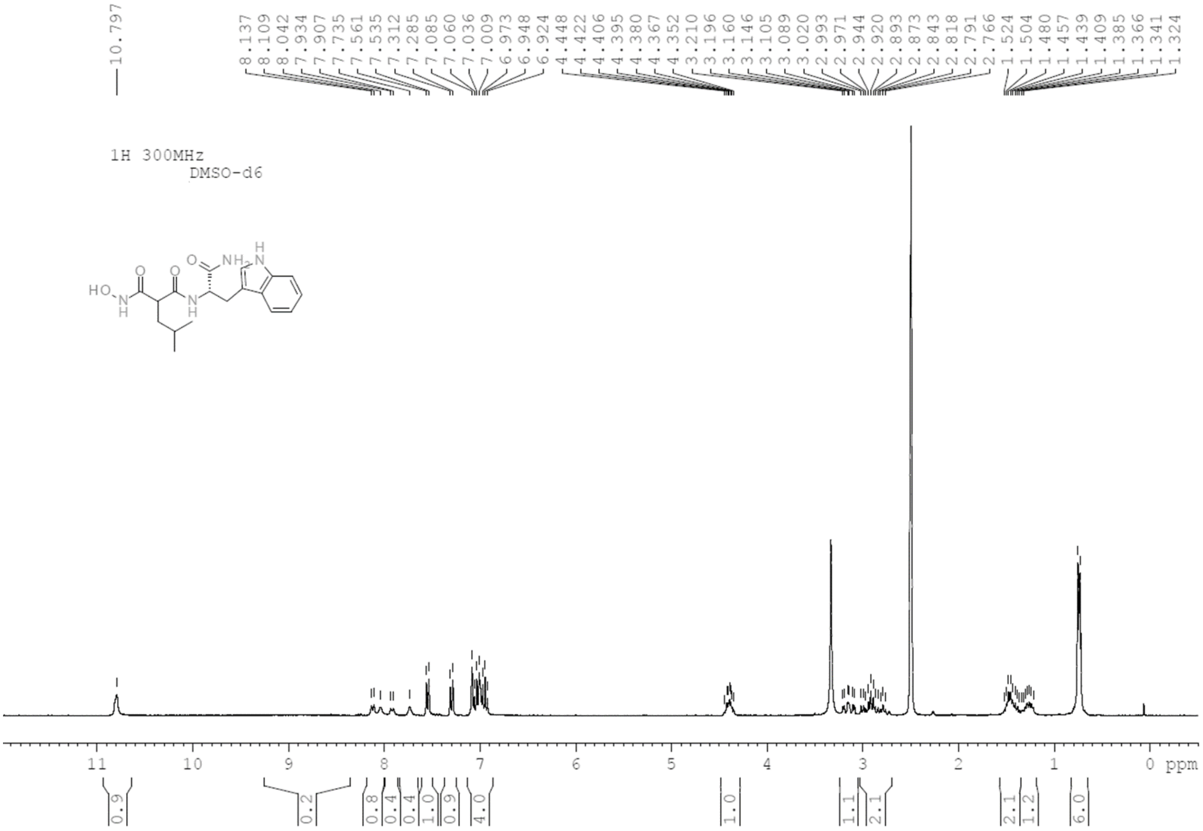

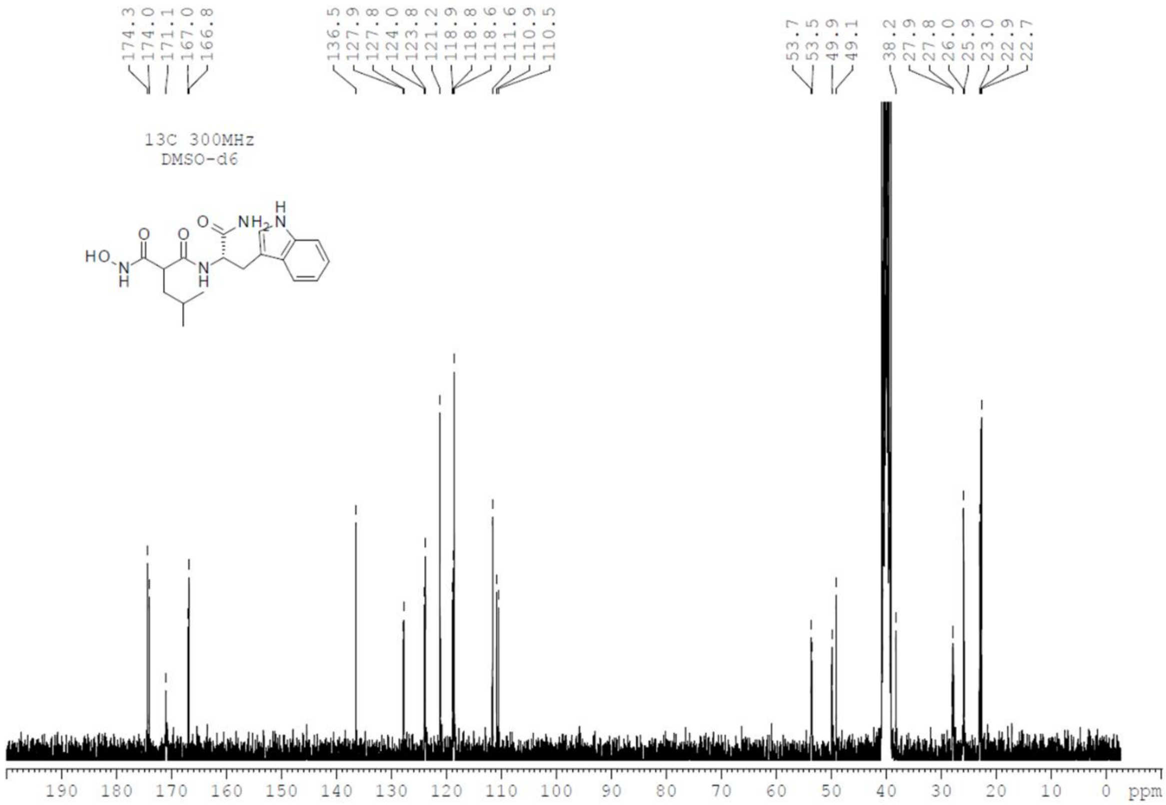

Compound 41

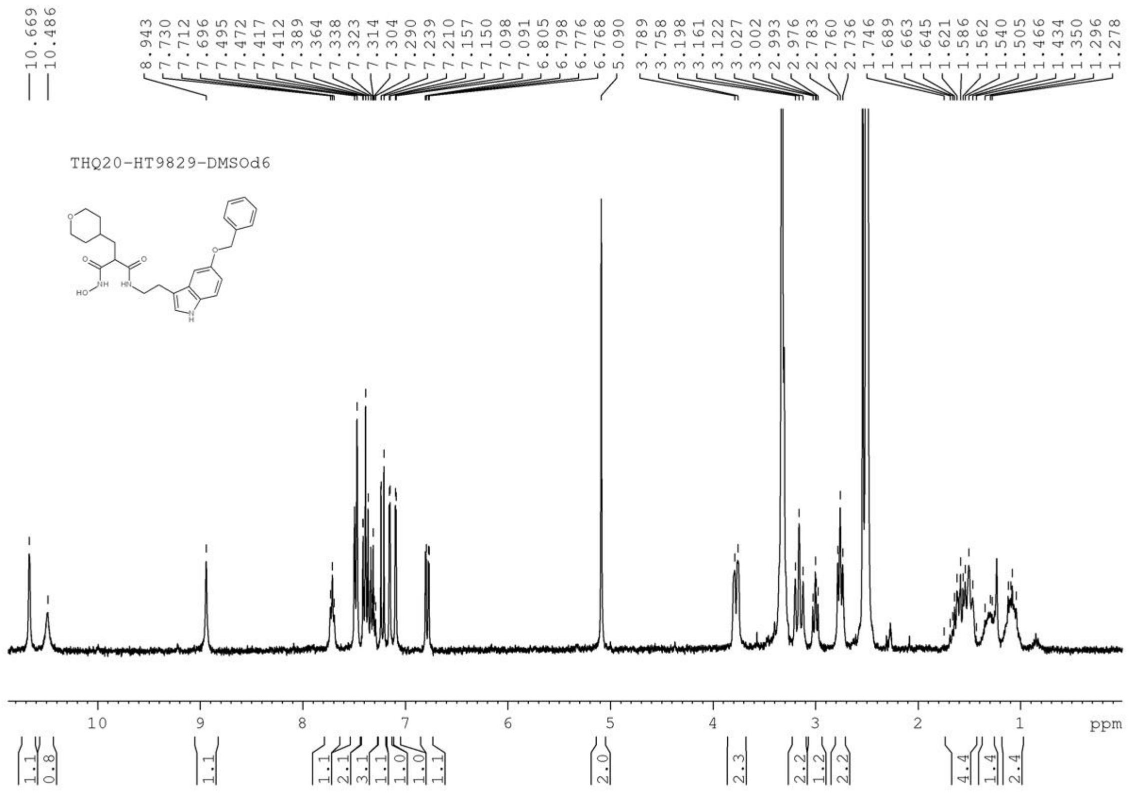
THQ20-HT9829-DMSOd6
10.669
10.486
8.943
7.730
7.712
7.696
7.495
7.472
7.417
7.412
7.389
7.364
7.338
7.323
7.314
7.304
7.290
7.239
7.210
7.157
7.150
7.098
7.091
6.805
6.798
6.776
6.768
5.090
3.789
3.758
3.198
3.161
3.122
3.027
3.002
2.993
2.976
2.783
2.760
2.736
1.746
1.689
1.663
1.645
1.621
1.586
1.562
1.540
1.505
1.466
1.434
1.350
1.296
1.278
ppm
1.1
0.8
1.1
1.1
2.1
3.1
1.1
1.0
1.0
1.1
2.0
2.3
2.2
1.2
2.2
4.4
1.4
2.4

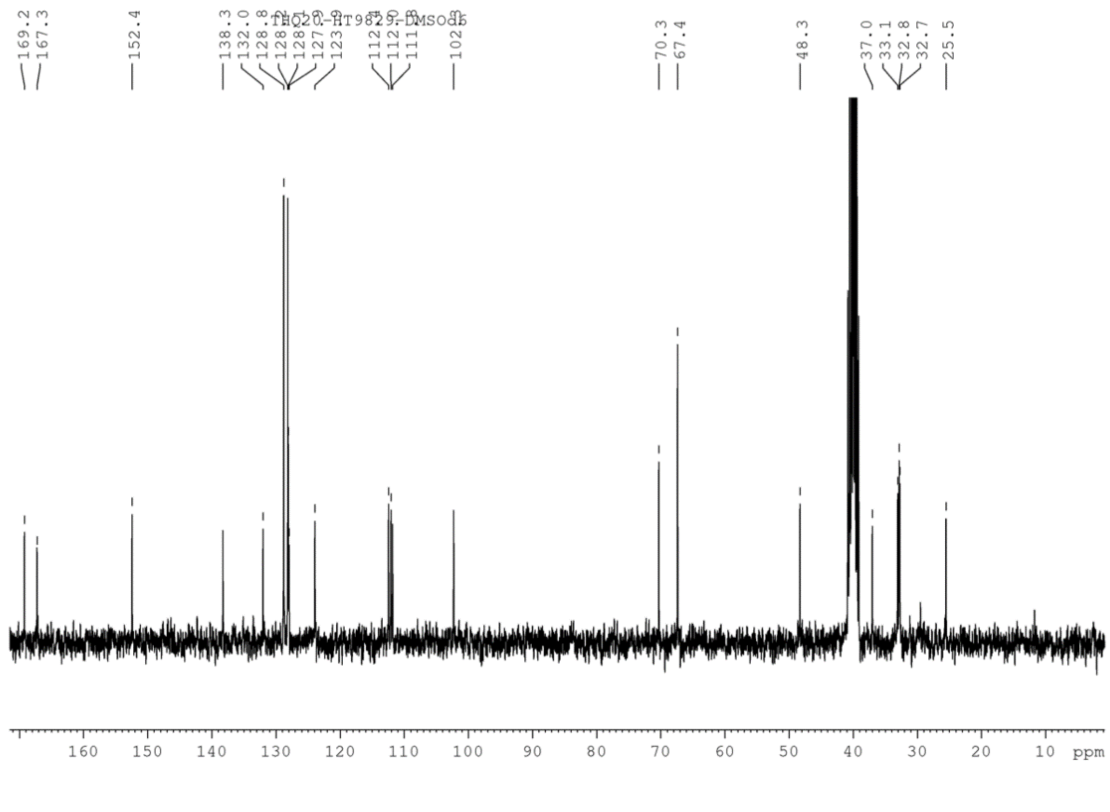
THQ20-HT9829-DMSOd6
169.2
167.3
152.4
138.3
132.0
128.8
128
127
123
112
112
111
102
70.3
67.4
48.3
37.0
33.1
32.8
32.7
25.5
ppm

Compound 42

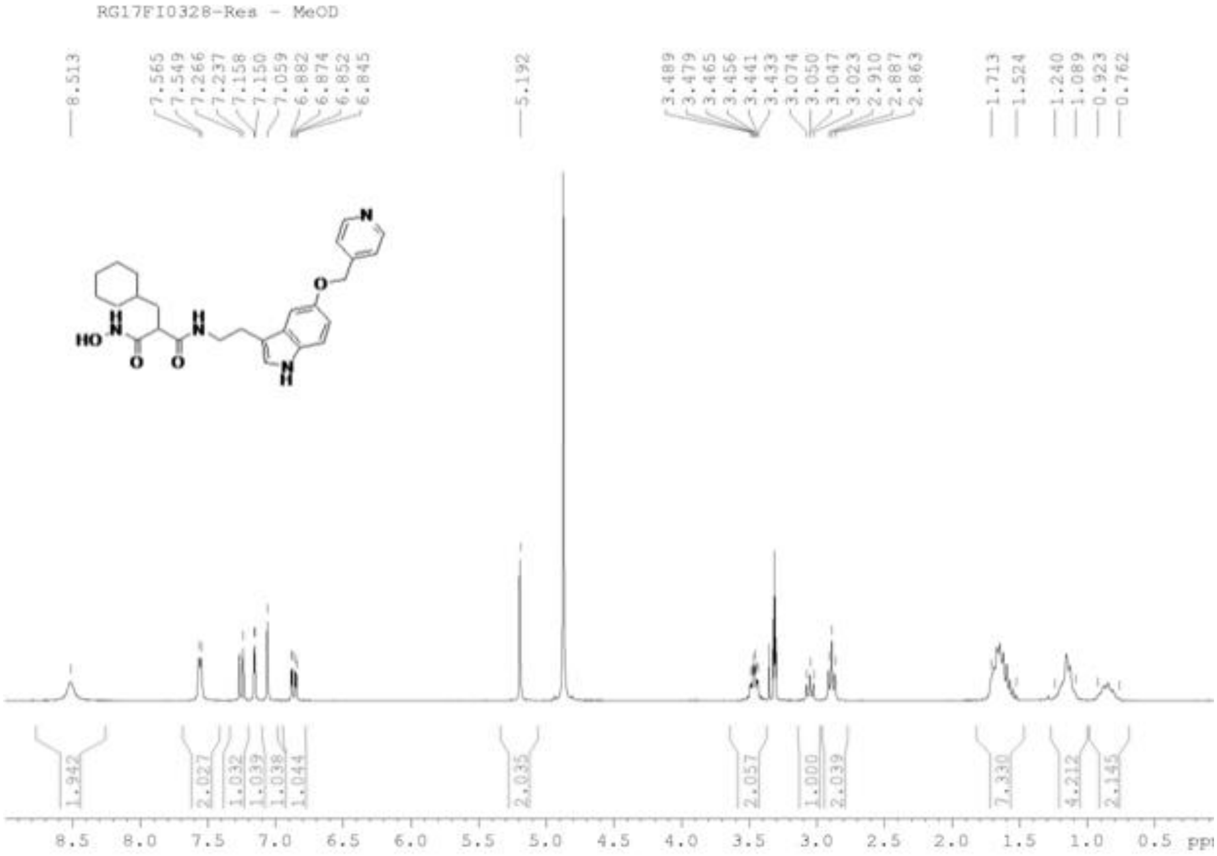
RG17FI0328-Res - MeOD
8.513
7.565 7.549 7.266 7.237 7.158 7.150 7.059 6.882 6.874 6.852 6.845
5.192
3.489 3.479 3.465 3.456 3.441 3.433 3.074 3.050 3.047 3.023 2.910 2.887 2.863
1.713 1.524
1.240 1.089 0.923 0.762
HO
N
H
O
O
N
H
N
H
O
N
1.942 2.027 1.032 1.039 1.038 1.044 2.035 2.057 1.000 2.039 7.330 4.212 2.145
8.5 8.0 7.5 7.0 6.5 6.0 5.5 5.0 4.5 4.0 3.5 3.0 2.5 2.0 1.5 1.0 0.5 ppm

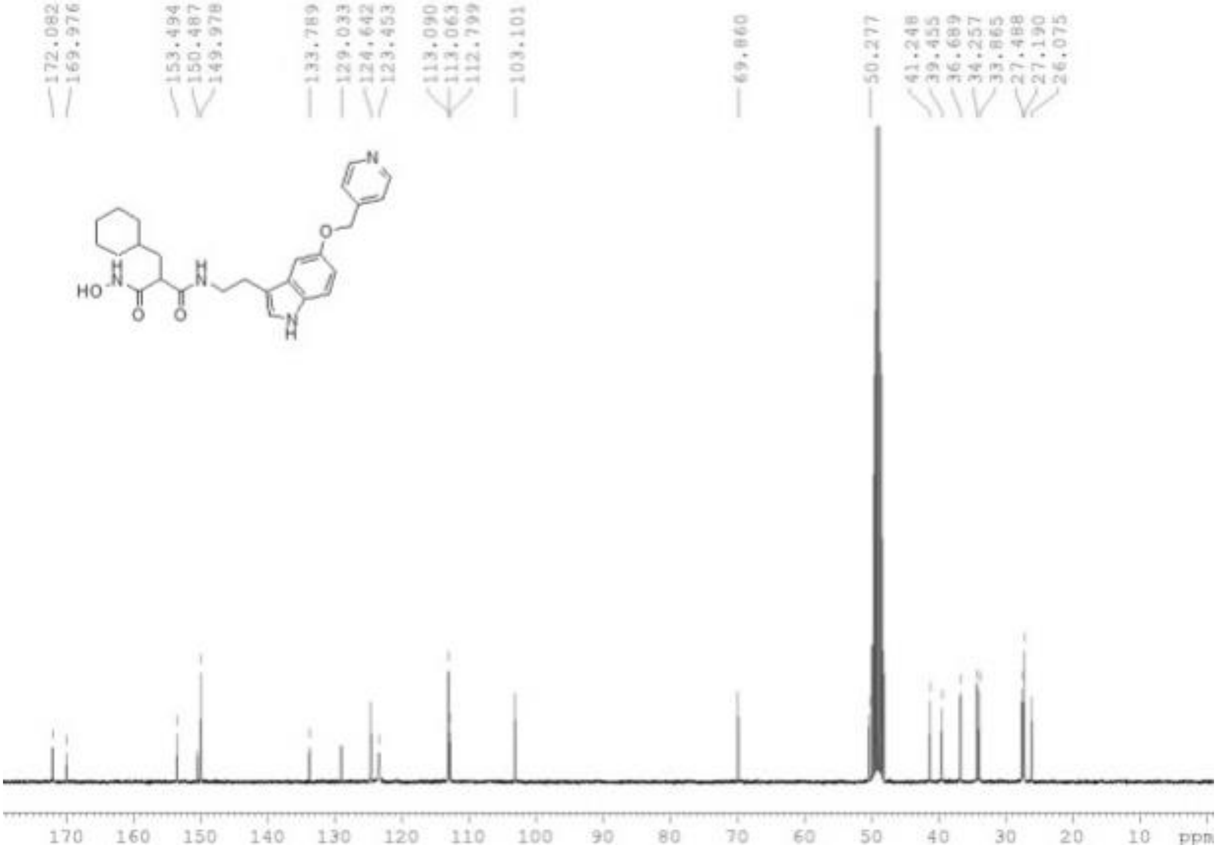
172.082 169.976
153.494 150.487 149.978
133.789 129.033 124.642 123.453
113.090 113.063 112.799
103.101
69.860
50.277
41.248 39.455 36.689 34.257 33.865 27.488 27.190 26.075
HO
N
H
O
O
N
H
N
H
O
N
170 160 150 140 130 120 110 100 90 80 70 60 50 40 30 20 10 ppm

Compound 43

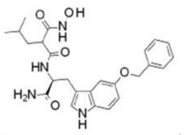

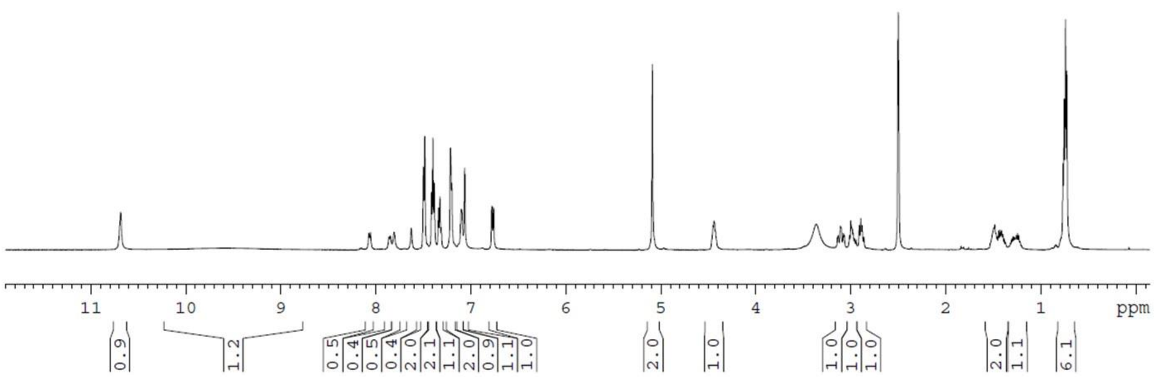

8.072
8.056
7.846
7.806
7.626
7.499
7.484
7.413
7.398
7.383
7.339
7.325
7.310
7.213
7.199
7.100
7.094
7.064
6.779
6.777
6.762
6.760
5.088
4.448
4.439
4.433
4.424
3.364
3.131
3.108
3.101
3.078
3.068
3.014
2.999
2.991
2.985
2.975
2.910
2.893
2.880
2.862
2.500
1.496
1.484
1.475
1.443
1.428
1.412
1.398
1.293
1.258
1.245
1.231
0.766
0.753
0.739
0.726
11 10 9 8 7 6 5 4 3 2 1 ppm
0.9
1.2
0.5
0.4
0.5
0.4
2.0
2.1
1.1
2.0
0.9
1.1
1.0
2.0
1.0
1.0
1.0
1.0
2.0
1.1
6.1

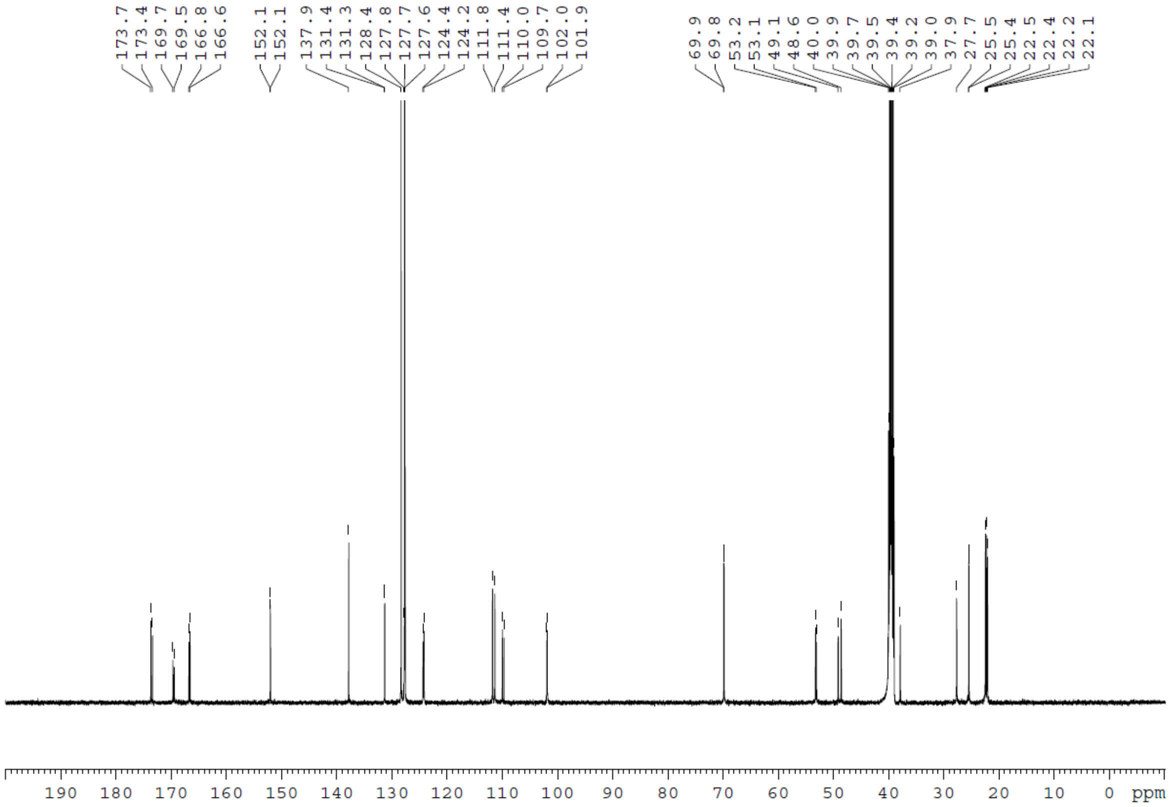

173.7
173.4
169.7
169.5
166.8
166.6
152.1
152.1
137.9
131.4
131.3
128.4
127.8
127.7
127.6
124.4
124.2
111.8
111.4
110.0
109.7
102.0
101.9
69.9
69.8
53.2
53.1
49.1
48.6
40.0
39.9
39.7
39.5
39.4
39.2
39.0
37.9
27.7
25.5
25.4
22.5
22.4
22.2
22.1
190 180 170 160 150 140 130 120 110 100 90 80 70 60 50 40 30 20 10 0 ppm

Compound 44

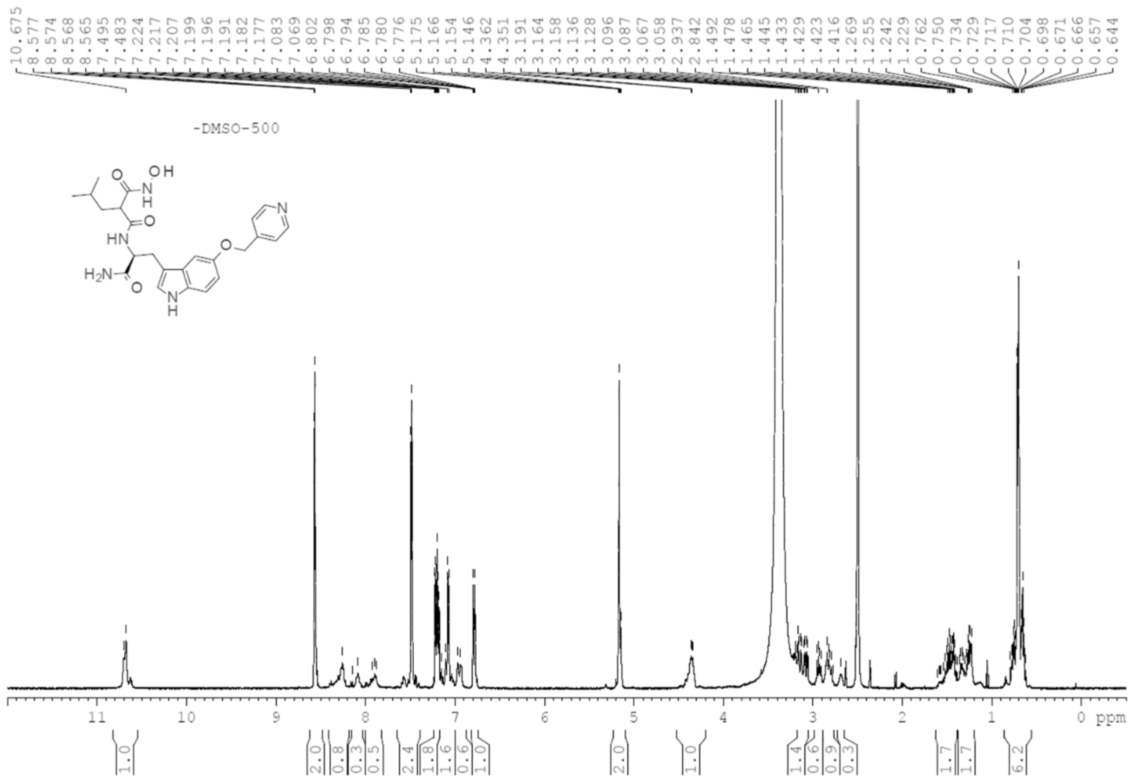
10.675 8.577 8.574 8.568 8.565 7.495 7.483 7.224 7.217 7.207 7.199 7.196 7.191 7.182 7.177 7.083 7.069 6.802 6.798 6.794 6.785 6.780 6.776 5.175 5.166 5.154 5.146 4.362 4.351 3.191 3.164 3.158 3.136 3.128 3.096 3.087 3.067 3.058 2.937 2.842 1.492 1.478 1.465 1.445 1.433 1.429 1.423 1.416 1.269 1.255 1.242 1.229 0.762 0.750 0.734 0.729 0.717 0.710 0.704 0.698 0.671 0.666 0.657 0.644
-DMSO-500
OH
N
H
O
O
HN
H2N
O
N
O
N
H
ppm
1.0
2.0 0.8 0.3 0.5
2.4 1.8 1.6 0.6 1.0
2.0
1.0
1.4 0.6 0.9 0.3
1.7 1.7
6.2

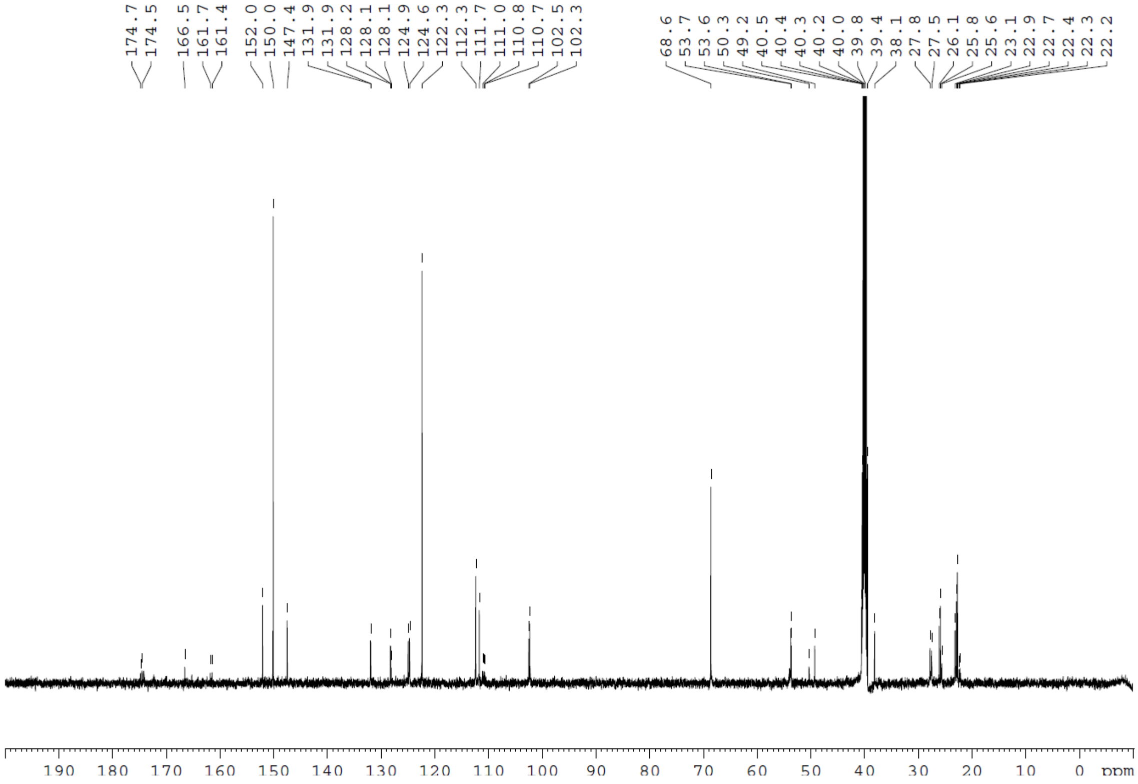
174.7 174.5 166.5 161.7 161.4 152.0 150.0 147.4 131.9 131.9 128.2 128.1 128.1 124.9 124.6 122.3 112.3 111.7 111.0 110.8 110.7 102.5 102.3
68.6 53.7 53.6 50.3 49.2 40.5 40.4 40.3 40.2 40.0 39.8 39.4 38.1 27.8 27.5 26.1 25.8 25.6 23.1 22.9 22.7 22.4 22.3 22.2
ppm
